# A combination screening to identify enhancers of *para*-aminosalicylic acid against *Mycobacterium tuberculosis*

**DOI:** 10.1038/s41598-022-08209-w

**Published:** 2022-04-04

**Authors:** Jinyeong Heo, Dahae Koh, Minjeong Woo, Doyoon Kwon, Virgínia Carla de Almeida Falcão, Connor Wood, Honggun Lee, Kideok Kim, Inhee Choi, Jichan Jang, Priscille Brodin, David Shum, Vincent Delorme

**Affiliations:** 1 Screening Discovery Platform, Institut Pasteur Korea, Seongnam, Gyeonggi 13488 Republic of Korea; 2 Tuberculosis Research Laboratory, Institut Pasteur Korea, Seongnam, Gyeonggi 13488 Republic of Korea; 3 Medicinal Chemistry Platform, Institut Pasteur Korea, Seongnam, Gyeonggi 13488 Republic of Korea; 4 Molecular Mechanisms of Antibiotics, Division of Life Science, Research Institute of Life Science, Department of Bio & Medical Big Data (BK21 Four Program), Gyeongsang National University, Jinju, 52828 Republic of Korea; 5 University of Lille, CNRS, INSERM, CHU Lille, Institut Pasteur de Lille, U1019 - UMR 9017 - CIIL - Center for Infection and Immunity of Lille, Lille, France

**Keywords:** Phenotypic screening, High-throughput screening, Tuberculosis

## Abstract

*Para*-aminosalicylic acid (PAS) is an antibiotic that was largely used for the multi-therapy of tuberculosis in the twentieth century. To try to overcome the inconvenience of its low efficacy and poor tolerance, we searched for novel chemical entities able to synergize with PAS using a combination screening against growing axenic *Mycobacterium tuberculosis*. The screening was performed at a sub-inhibitory concentration of PAS on a library of about 100,000 small molecules. Selected hit compounds were analyzed by dose–response and further probed with an intracellular macrophage assay. Scaffolds with potential additive effect with PAS are reported, opening interesting prospects for mechanism of action studies. We also report here evidence of a yet unknown bio-activation mechanism, involving activation of pyrido[1,2-a]pyrimidin-4-one (PP) derivatives through the Rv3087 protein.

## Introduction

**Figure 1:**
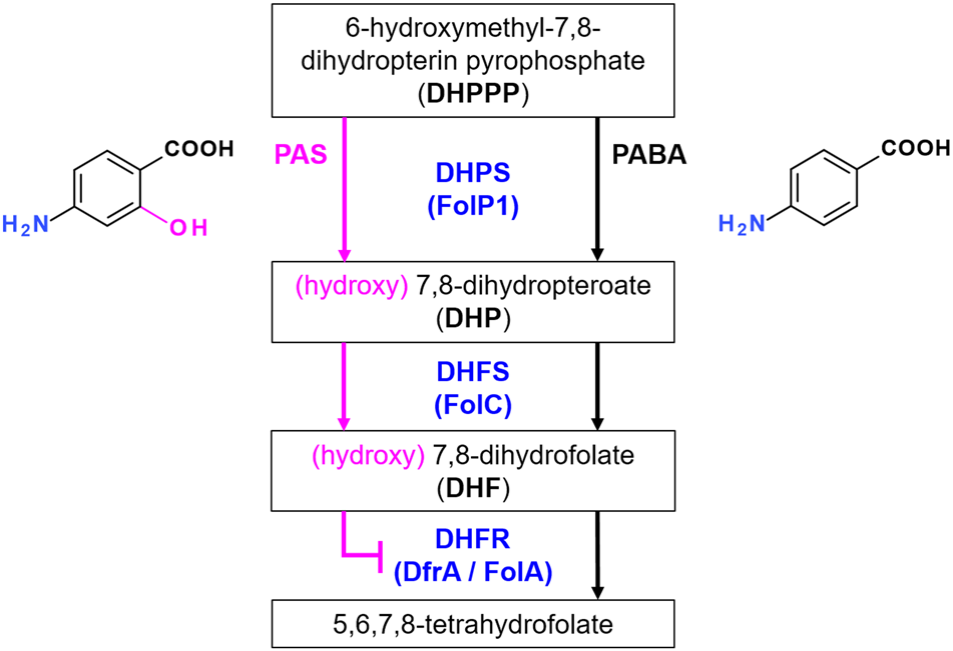
Simplified representation of the folate synthesis pathway in *M. tuberculosis* and the proposed mechanism of action for *para*-aminosalicylic acid (PAS). Incorporation of PAS instead of *para*-aminobenzoic acid (PABA) in the pathway, through the action of FolP1 (dihydropteroate synthase, DHPS), lead to the formation of an hydroxylated 7,8-dihydropteroate (DHP) intermediate, itself converted into an anti-folate metabolite by FolC (dihydrofolate synthase, DHFS), resulting in downstream inhibition of FolA (dihydrofolate reductase, DHFR).

The treatment of tuberculosis (TB) using antibiotics started in the 1940s with streptomycin^1^ and *para*-aminosalicylic acid (PAS)^2^. Although clinically effective the high dosages required for PAS (12 g daily for adults), the side effects associated with its usage and its somehow moderate bacteriostatic activity compared to other oral drugs like isoniazid and ethambutol led to its removal from the first line treatment of TB^3^. Despite this, PAS is still in use currently for the treatment of drug-resistant TB infections and is listed on the World Health Organization's List of Essential Medicines^4^. The mechanism of action of PAS remained elusive for more than 50 years, but strong evidence gathered by several groups has now clearly established a pro-drug scenario^5–7^. In this model, PAS is incorporated competitively with *para*-aminobenzoic acid (PABA) in the folate synthesis pathway by dihydropteroate synthase (DHPS, FolP1) leading to downstream inhibition of dihydrofolate reductase (DHFR, DfrA or FolA) and arrest of bacterial growth (Fig. 1). It has also been reported recently that the active metabolite of PAS (obtained after action of FolC) could inhibit the Flavin-dependent thymidylate synthase (ThyX) located downstream in the folate cycle^8,9^. Because PAS and PABA share similar affinity for FolP1^5,7^, any mechanism leading to a depletion of the PABA pool could favor the synthesis of the anti-folate intermediates, resulting in a more efficient inhibition of the folate synthesis^10^. A reduction in the pool of 6-hydroxymethyl-7,8-dihydropterin pyrophosphate (DHPPP, the substrate of FolP1) could also lead to the decreased formation of folate, further exacerbating the effect of PAS. Conversely, compounds affecting folate precursor biosynthesis could also antagonize with PAS, as observed for methionine^11^. In summary, any compound leading to alteration of the PABA or DHPPP pool could potentially generate a response (increased or decreased resistance) when used in combination with PAS, while showing no or minor effects on bacterial growth when used alone. In order to test this hypothesis and probe for compounds showing additivity, synergy or antagonism with PAS, we gathered a library of commercially available small molecules and tested them alone and in combination with a sub-inhibitory concentration of PAS. We report here the results of this screening and highlight a few scaffolds that may prove useful for the study of the folate pathway in mycobacteria.

## Results

### Assay design and primary screening

**Figure 2:**
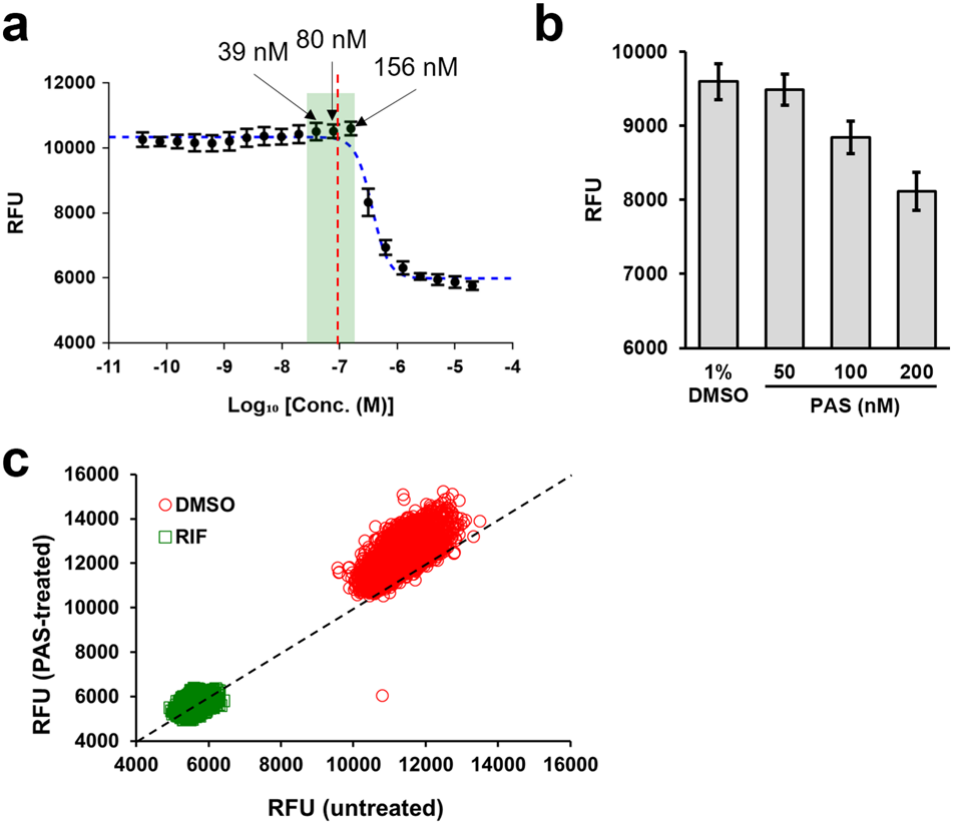
Screening optimization and validation. (**a**) Dose–response curve of PAS against H37Rv-GFP and selection of the highest, non-inhibitory concentration for the screening. The curve contained 20 doses, starting from 20 μM with twofold dilution between each dose and was repeated 4 times with 3 replicates each time. All values were pooled and average ± SD are shown on the graph (n = 12). The acceptable range of concentration for PAS is indicated in green, the 3 corresponding concentrations from the dose–response curve are indicated. The red, dashed line indicates the position of the 100 nM concentration. The best fit for the dose–response (using a sigmoidal dose–response model, 4 parameters) is indicated as a blue dashed line. (**b**) Confirmation of the effect of PAS on the growth of H37Rv-GFP, at three selected concentrations. Experiment was repeated 3 times and the average ± SD for a representative result are shown (n = 12). (**c**) Aggregated raw fluorescence values (RFU) for the positive (1 μg/mL rifampicin, RIF) and negative (1% DMSO) controls, for each of the two screening sets (PAS-treated, untreated). The black dashed line indicates equal RFU values for both sets (Y = X).

**Figure 3:**
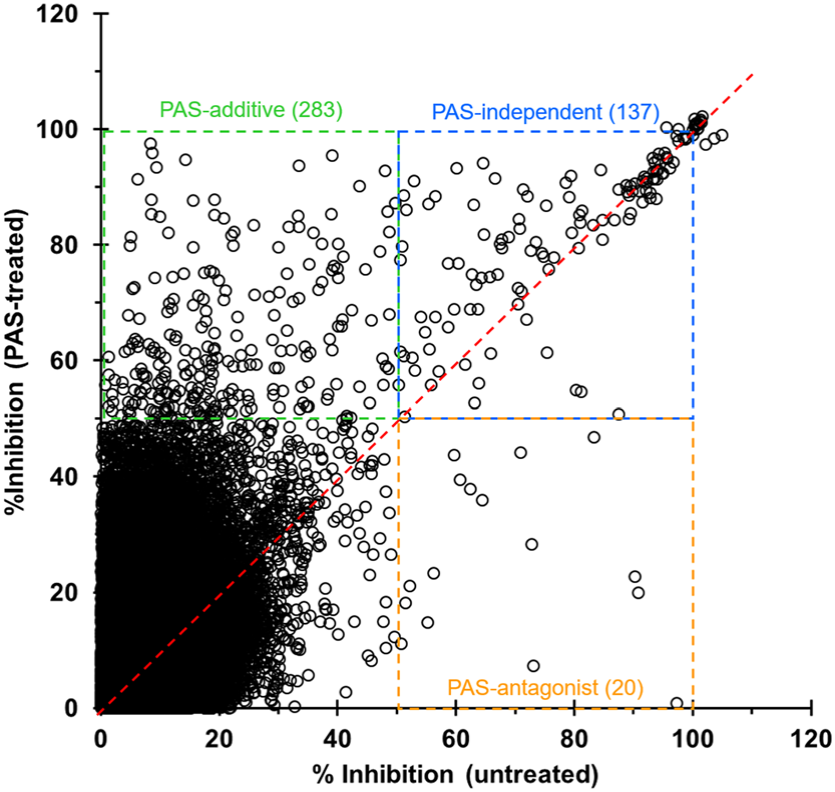
Distribution of the compound inhibition data for both screening sets (PAS-treated, untreated). Hits were selected at an inhibition threshold of 50% and grouped into 3 categories, as shown with dashed boxes on the graph: PAS-additive (top-left), PAS-independent (top-right) and PAS-antagonist (bottom-right). The number of hits in each box is indicated. The red-dashed line indicates equal percentage of inhibition for both sets (Y = X).

For this combination screening, a key requirement was to work with a concentration of PAS that was high enough to provide a combination effect and yet still below the inhibitory dose, to allow the bacteria to replicate. Based on the dose–response curves obtained for PAS against our H37Rv-GFP reporter strain (a *Mycobacterium tuberculosis* H37Rv strain constitutively expressing a green fluorescent protein), concentrations of 40 to 150 nM appeared to be suitable for this purpose (Fig. 2a). Considering that higher concentrations would lead to visible effects on the bacterial growth and lower concentrations would be at risk of producing insufficient combination effect with the compound library, the 100 nM concentration was selected and validated after repeated measures (Fig. 2b). The entire library of compounds was then screened in two distinct sets (PAS-treated and untreated) to identify potential combination effects. Aggregation of the raw fluorescence values for the controls of each set indicated that the selected concentration of PAS (100 nM) had no impact on the bacterial growth, as initially desired (Fig. 2c). The average Z’ score for all the plate was 0.70 (Untreated set 0.72; PAS-treated set 0.68), indicating a good and reproducible assay. Data for each set were normalized as percentage of inhibition using the corresponding average values of the positive and negative controls, calculated on a plate-by-plate basis. Based on the distribution of the percentage of inhibition for all the compounds (Fig. S1), we decided to select hits at a cut-off of 50% inhibition. Hits were grouped into 3 categories: (i) PAS-additive, compounds that were selected as hits only in presence of PAS; (ii) PAS-independent, compounds that were selected as hits in both sets; (iii) PAS-antagonist, compounds that were selected only in the absence of PAS (Fig. 3). A total of 440 unique compounds were selected using this threshold.

### Secondary screening and hit analysis

**Figure 4:**
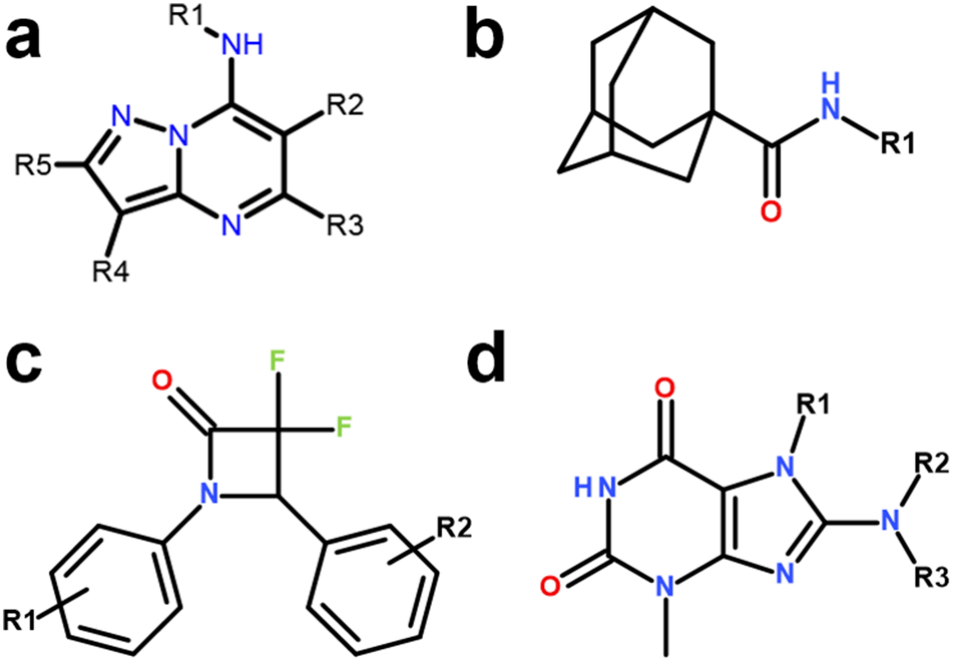
Structure of the main hit clusters identified. (**a**) Aminopyrazolo[1,5-*a*]pyrimidine (APP). (**b**) Adamantyl amide (AA). (**c**) 3,3-difluoro-2-azetidinone (DFA). (**d**) purine-like (PL) compounds.

Among the 440 hits, 337 compounds were selected for validation by cherry-picking and dose–response curve. Each hit was validated using a 10 points dose–response starting at 25 μM and performed in two sets (PAS-treated and untreated), in conditions identical to that used for the primary screening. Based on the results, compounds were labelled as *Additive*, *Antagonist*, *Independent* or *Inactive*, as described in the materials and methods section. Out of the 337 compounds tested, 112 were labelled as *Inactive*, 87 as *Independent*, 3 as *Antagonist* and 135 as *Additive* (Fig. S1). A similarity analysis on the 225 validated hits was used to identify clusters of active molecules, revealing 4 main groups, each presenting at least 6 active molecules (Figs. S2, S3). Among these was a group of 15 derivatives related to the aminopyrazolo[1,5-a]pyrimidine (APP) scaffold (Fig. 4a and Fig. S4), which has been reported and explored earlier^12^. We found that most of these derivatives (12/15) showed good additivity with PAS, with ln(FC) values up to 1.54 (up to 4.7 times more potent in presence of PAS, Table 1). The mechanism of action of these APP compounds has not been described yet, but the additivity values observed here seems to indicate a possible involvement with the folate pathway, although more experiments would be required to confirm this point.

**Table 1:** Activity profile of the aminopyrazolo[1,5-*a*]pyrimidines (APP) against H37Rv-GFP, in presence or absence of PAS.

Compound	IC_50_ (μM)^a^		Ln(FC)	Category
	Untreated	PAS-treated		
APP_1	1.6 (1.4–1.7)	0.82 (0.69–0.97)	0.67	Additive
APP_2	2.6 (2.4–2.9)	0.95 (0.84–1.1)	1.01	Additive
APP_3	2.5 (2.1–3.0)	1.5 (1.4–1.7)	0.51	Additive
APP_4	1.3 (1.2–1.4)	0.57 (0.54–0.61)	0.82	Additive
APP_5	3.1 (2.1–4.5)	1.2 (1.1–1.3)	0.95	Additive
APP_6	1.7 (1.4–2.1)	0.87 (0.83–0.92)	0.67	Additive
APP_7	1.3 (1.2–1.4)	0.74 (0.69–0.79)	0.56	Additive
APP_8	2.8 (2.6–3.0)	0.73 (0.63–0.85)	1.34	Additive
APP_9	2.4 (2.3–2.6)	1.2 (1.1–1.3)	0.69	Additive
APP_10	3.0 (2.8–3.3)	1.9 (1.8–2.0)	0.46	Independent
APP_11	2.0 (1.9–2.2)	1.1 (1.0–1.2)	0.60	Additive
APP_12	1.5 (1.4–1.7)	0.80 (0.74–0.87)	0.63	Additive
APP_13	11.2 (7.9–15.8)	2.4 (2.1–2.6)	1.54	Additive
APP_14	2.4 (2.3–2.6)	0.80 (0.71–0.89)	1.10	Additive
APP_15	1.6 (1.4–2.0)	1.3 (1.2–1.3)	0.21	Independent

^a^Values are best-fit for a duplicated dose–response. The 95% CI is indicated in parenthesis.

Another cluster identified was the adamantyl amide scaffold, for which 11 molecules were present (Fig. 4b and Fig. S5). Similar compounds have been reported earlier and were linked to inhibition of MmpL3, including the adamantyl amine SQ109 (reviewed in^13^). Interestingly, all the 11 compounds showed good additivity with PAS, with up to 4.3 times increase in potency, while 6 compounds showed a measurable activity only in the presence of PAS (Table 2). Modulation of *mmpL3* expression has been shown to induce changes in cellular permeability^14^, suggesting that the effect seen here could be mediated by an increased penetration of PAS through the membrane rather than a combination within the folate pathway.

**Table 2:** Activity profile of the adamantyl amides (AA) against H37Rv-GFP, in presence or absence of PAS.

Compound	IC_50_ (μM)^a^		Ln(FC)	Category
	Untreated	PAS-treated		
AA_1	NA	2.8 (2.4–3.2)	–	Additive
AA_2	11.4 (no plateau)	5.4 (3.2–8.9)	0.70	Additive
AA_3	17.1 (no plateau)	9.2 (6.7–1.3)	0.62	Additive
AA_4	NA	8.8 (7.0–11.1)	–	Additive
AA_5	4.5 (3.1–5.1)	1.8 (1.4–2.3)	0.92	Additive
AA_6	3.9 (3.2–4.9)	0.91 (0.70–1.2)	1.46	Additive
AA_7	9.0 (6.7–9.4)	3.7 (3.0–4.6)	0.89	Additive
AA_8	NA	7.0 (5.6–8.9)	–	Additive
AA_9	NA	20.8 (no plateau)	–	Additive
AA_10	NA	5.9 (4.5–7.6)	–	Additive
AA_11	NA	11.8 (no plateau)	–	Additive

^a^Values are best-fit for a duplicated dose–response. The 95% CI is indicated in parenthesis, except for cases where it was very wide (no plateau for the bottom value of the curve). NA: not active or IC_50_ > 25 μM.

A group of 4 derivatives bearing a 4,5-dichloro-2*H*-pyridazin-3-one (DCP) moiety was also noted (Figs. S2, S3). Careful examination of the hit list actually revealed that a total of 8 derivatives shared this DCP moiety (Fig. S6). The wide biological effects of compounds containing pyridazinones has been highlighted in the past^15^, but, to our knowledge, such compounds have not been reported for anti-tubercular activities so far. The two most potent derivatives (DCP_3, DCP_7) were found to be PAS-independent, which was confirmed during the secondary screening, as well as by dose–response assay with the resynthesized compounds (Fig. S6). Both compounds showed attractive IC_50_ values of 2 to 5 μM against H37Rv-GFP, were not cytotoxic to macrophages at 50 μM and were also found to be active in macrophages with IC_50_ values below 10 μM (Fig. S7), indicating potential value for further exploration.

Of lower interest was a cluster of 4 compounds related to the 8-hydroxyquinoline (8-HQ) family, which were already reported and studied in the past, in particular for their property of metal chelation^16,17^. These 4 derivatives were actually ester forms of 8-HQ. They showed IC_50_ values of 2–7 μM during the secondary screening and were found to be PAS-insensitive (Fig. S8). Additionally, a cluster of 4 compounds presenting a naphtho[2,3-b]furan-4,9-dione (NFD) core structure with a carboxamide linker was observed. Similar compounds were reported for their growth inhibitory effects against *Trypanosoma cruzi* epimastigotes^18^. We could not find direct reports of anti-tubercular activity, but related 1,4-naphtoquinones were previously reported^16^. The activity of the NFD compounds was confirmed by dose–response (secondary screening) and found to be PAS-insensitive as well, with more modest IC_50_ values however, ranging from 10 to 20 μM (Fig. S9).

A small cluster of 3 molecules from the pyrido[1,2-a]pyrimidin-4-one (PP) family was also observed (Fig. S10). Similar compounds were previously identified as inhibitors of H37Rv-GFP replication in macrophages^19^, leading to structure–activity relationship studies and a patent application^20^. Despite promising in vitro properties (bactericidal activity, sub-micromolar activity against drug-resistant isolates), the compounds were found unfit for clinical development due to poor solubility and low metabolic stability. Here, we tried to advance the understanding of the mechanism of action for these compounds by generating spontaneous resistant mutants. Whole genome sequencing studies revealed mutations in the *rv3087* gene, encoding a putative triacylglycerol synthase (Fig. S11). In multiple cases, these mutations likely led to loss of protein function and strong resistance, indicating a possible bio-activation mechanism. Accordingly, two H37Rv Rv3087-KO strains constructed by disruption of the *rv3087* gene by insertion of a hygromycin resistance cassette by allelic replacement were viable and acquired resistance to PP compounds (Figs. S11, S12). Overexpression of Rv3087 in the resistant mutants also restored the susceptibility to a level similar to that seen for the wild-type (Fig. S12). These results reinforced the idea that Rv3087 could be acting as a bio-activator for PP compounds, presumably via alkylation of the free alcohol position.

Finally, of additional interest from the screening campaign conducted here was a group of 6 derivatives sharing a 3,3-difluoro-2-azetidinone moiety, resembling beta-lactams (DFA, Fig. 4c and Fig. S13). However, these compounds were active only when used in combination with PAS (Table 3) and their activity remained relatively low (IC_50_ > 5 μM). A last group of 6 derivatives with a structure related to that of purine, more specifically theobromine, was highlighted by the similarity analysis (Fig. 4d and Fig. S14). All 6 purine-like (PL) compounds were also found to be more active in presence of PAS, with 4 of them showing visible activity only in the presence of PAS (Table 4). The similitude between these PL compounds and the pterin-based substrates and intermediates present in the folate pathway is interesting and may hint at a potential combination effect with the PAS.

**Table 3:** Activity profile of the 3,3-difluoro-2-azetidinone (DFA) against H37Rv-GFP, in presence or absence of PAS.

Compound	IC_50_ (μM)^a^		Ln(FC)	Category
	Untreated	PAS-treated		
DFA_1	NA	7.5 (3.6–16.0)	–	Additive
DFA_2	NA	6.4 (5.5–7.3)	–	Additive
DFA_3	NA	8.0 (6.7–9.5)	–	Additive
DFA_4	NA	14.4 (no plateau)	–	Additive
DFA_5	NA	4.8 (2.8–8.3)	–	Additive
DFA_6	NA	9.5 (7.7–11.8)	–	Additive

^a^Values are best-fit for a duplicated dose–response. The 95% CI is indicated in parenthesis, except for cases where it was very wide (no plateau for the bottom value of the curve). NA: not active or IC_50_ > 25 μM.

**Table 4:** Activity profile of the purine-like (PL) derivatives against H37Rv-GFP, in presence or absence of PAS.

Compound	IC_50_ (μM)^a^		Ln(FC)	Category
	Untreated	PAS-treated		
PL_1	NA	7.6 (6.4–9.0)	–	Additive
PL_2	NA	3.5 (3.2–4.0)	–	Additive
PL_3	NA	10.3 (no plateau)	–	Additive
PL_4	8.7 (5.9–12.8)	4.8 (4.3–5.4)	0.59	Additive
PL_5	NA	23.2 (no plateau)	–	Additive
PL_6	8.4 (5.5–12.7)	4.8 (4.2–5.5)	0.56	Additive

^a^Values are best-fit for a duplicated dose–response. The 95% CI is indicated in parenthesis, except for cases where it was very wide (no plateau for the bottom value of the curve). NA: not active or IC_50_ > 25 μM.

### Re-synthesis and confirmation

To allow mechanistic studies and further biological evaluation of the DFA and PL compounds, we decided to re-synthesize and confirm the activity profile of these derivatives. All DFA and PL compounds were obtained in two steps with overall (non-optimized) yields of 10–30% and purities above 95%, as confirmed by HPLC and LC–MS analyses (See Appendix for all NMR, HPLC and LC–MS data). All 12 compounds were tested again in dose–response for their activity against H37Rv-GFP, in a protocol identical to that of the screening (untreated, PAS-treated). Results obtained were mostly consistent with the results of the secondary screening, indicating that both families were indeed dependent of PAS to show some inhibitory activity (Table 5). In several cases however, IC_50_ values found for the re-synthesized compounds were lower than that of the screening library counterpart. For the PL scaffold, the freshly synthesized PL_4 and PL_6 derivatives were found to be PAS-independent, an interesting result given their structural differences compared to the other PL derivatives (3 fused cycles instead of 2).

**Table 5:** Activity profile of the re-synthesized DFA and PL derivatives against H37Rv-GFP, in presence or absence of PAS.

Compound	IC_50_ (μM)^a^		Ln(FC)	Category
	Untreated	PAS-treated		
DFA_1	NA	5.5 ± 0.35	–	Additive
DFA_2	NA	6.8 ± 2.5	–	Additive
DFA_3	NA	4.6 ± 0.30	–	Additive
DFA_4	NA	5.3 ± 2.2	–	Additive
DFA_5	NA	5.9 ± 0.90	–	Additive
DFA_6	NA	7.5 ± 3.2	–	Additive
PL_1	NA	5.5 ± 3.2	–	Additive
PL_2	2.8 ± 1.1	1.2 ± 0.50	0.85	Additive
PL_3	8.7 ± 2.7	3.2 ± 1.2	1.00	Additive
PL_4	3.8 ± 1.9	3.9 ± 0.68	− 0.03	Independent
PL_5	10.4 ± 0.72	5.4 ± 3.3	0.66	Additive
PL_6	3.7 ± 1.5	3.5 ± 0.79	0.06	Independent

^a^Values are average ± SD for three independent experiments, each conducted with a duplicated dose–response. NA: not active or IC_50_ > 25 μM.

Despite slight toxicity against murine macrophages (Raw264.7) at concentrations above 10 μM (Fig. S15), PL_4 and PL_6 derivatives were tested against H37Rv-GFP replicating in murine macrophages and found to be active, with IC_50_ values of 1 to 2 μM (Figs. S16, S17), while all the other derivatives (including the 6 DFA compounds) were inactive in the macrophage assay. We noted however that compound PL_6 presented more obvious signs of cytotoxicity in these conditions (5 days incubation) as compared to the 2-day cytotoxicity experiment (Fig. S16). Additionally, the activity of PL_4 and PL_6 in macrophages was not affected by PAS (Fig. S17), neither was the intracellular activity of the DFA derivatives. This was somewhat expected given the lack of activity for PAS in this intracellular model: Rand et al. reported no effects on bacterial CFU counts at concentrations up to 1 mM^21^ and we confirmed here using our assay that PAS was unable to eliminate intracellular bacteria for concentrations up to 20 μM (Fig S18). Penetration issues for PAS, DFA or both might be to blame for the absence of activity seen for the combination.

**Figure 5:**
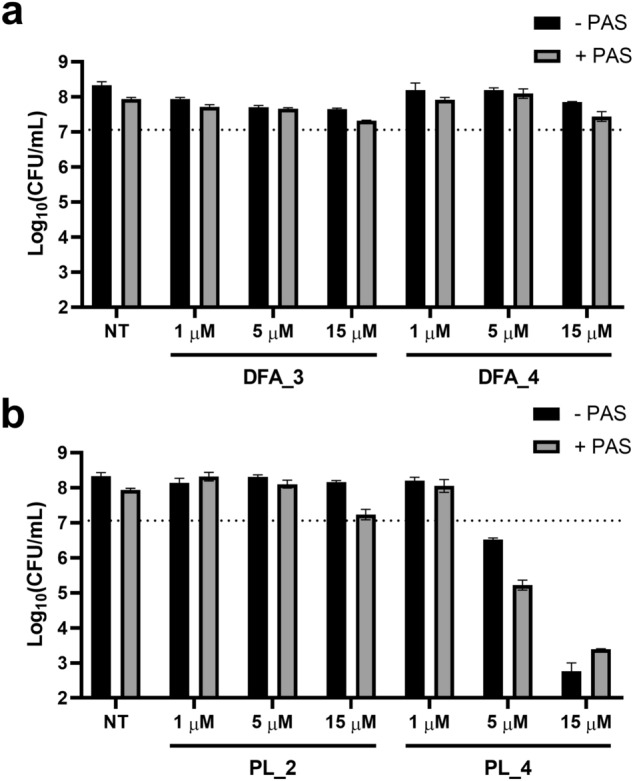
Evaluation of DFA and PL additivity with PAS using a colony-forming unit (CFU) assay. H37Rv wild-type bacteria were incubated with (**a**) DFA and (**b**) PL compounds at various concentrations, in presence or absence of PAS (100 nM). Aliquots were withdrawn at regular interval and plated on solid agar for CFU enumeration. Data shown here are for 7 days of incubation (see Fig. S18 for additional data at day 2) and represent average ± SD for plating made in duplicate. The horizontal dotted line indicates the CFU counts obtained at day 0 (7.06 ± 0.3).

To further confirm the previous screening results, we conducted a time-kill assay based on colony–forming units (CFU) enumeration for PL and DFA compounds. Bacteria were incubated with the compounds alone or in combination with PAS and aliquots taken at 2 and 7 days for CFU plating. For the two DFA compounds selected (DFA_3 and DFA_4), results indicated small gains in CFU reduction after 7 days of incubation when the PAS combination was used with the highest dose (15 μM), but the combination remained bacteriostatic (Fig. 5a and Fig. S19). Accordingly, we also noted that the colonies took more time to develop for these compounds, as compared to the controls, which could explain the more visible differences seen in liquid broth over 5 days of incubation. As a way to confirm this fact, DFA_3 and DFA_4 were combined with other antibiotics and the combinations evaluated against planktonic cultures of H37Rv, over periods of 5 days. When used at 10 μM, DFA compounds showed slight reduction of the IC_50_ values for both isoniazid and rifampicin (Fig. S20), further indicating that DFA compounds may exert a slight bacteriostatic effect and that this effect may not be specific to PAS combinations only.

For the PL compounds, PL_2 and PL_4 were chosen as representative of the potential PAS-dependent and PAS-independent sub-group, respectively. Results of the time-kill experiments indicated that PL_2 was indeed dependent of PAS to show some activity at the highest dose, although the combination remained bacteriostatic (Fig. 5b and Fig. S19). For PL_4 however a clear bactericidal activity was seen, but PAS added little to the compound activity, as expected from our previous data.

## Discussion

In this work we explored the possibility to identify compounds potentiating or synergizing with PAS using a combination screening strategy. This type of approach is not new and has been successfully used in the TB field already, to identify synergistic drug combinations^22^ or potent boosters of ethionamide activity^23^ for example. To our knowledge this is the first attempt to do so by focusing on the folate pathway, an attractive yet underexplored vulnerability in *Mycobacterium tuberculosis*^24^. From an initial library of about 100,000 small molecules, we identified 440 unique hits (0.44%) when combining results from both screening sets (untreated and PAS-treated). We chose to conduct a secondary screening by cherry-picking to save time, but some compounds were available only in limited quantities and were excluded (103 compounds). Despite a high rate of false positive (112, 33% of the retested hits), presumably due to the fact that the primary screening was not performed in duplicates, we were able to validate compounds with both PAS-additive and PAS-independent properties.

Among the validated compounds, 135 were presenting potential additive effects with PAS (31% of total hits, Fig. S1) and the results obtained with the newly synthesized compounds showed good consensus with results from the secondary screening. This indicated that our compound classification into PAS-additive and PAS-independent categories based on the secondary screening data was relatively precise, although preliminary. Strikingly, no synergistic compounds were found so far and only mild additivity was observed. We have not yet confirmed all of the PAS-additive hits by dose–response or following chemical re-synthesis however, so we hope to be able to find better additivity profile in this remaining pool of compounds. In the meantime, we highlighted here few scaffolds of interest for which our data showed an additive effect with PAS. The purine-like (PL) compounds in particular (Fig. 4d), given their molecular structures, have a higher likelihood of being directly involved in the folate pathway, opening interesting avenues for the study of their mechanism of action.

Analysis of hit clusters from the untreated set did not reveal many new families of anti-tubercular compounds. The aminopyrazolo[1,5-a]pyrimidine (APP) and adamantyl amide (AA), the two most populated clusters, were indeed already reported^12,13^. This was also the case for the 8-hydroxyquinoline (8-HQ) and pyrido[1,2-a]pyrimidin-4-one (PP) derivatives^16,20^. Only the 4,5-dichloro-2*H*-pyridazin-3-one (DCP) scaffold seems to have escaped the meticulous investigations conducted previously. We found that two derivatives in particular, DCP_3 and DCP_7, could present interesting value for further development in hit-to-lead program (Fig. S6, S7). Interestingly however, data from the PAS-treated set allowed us to identify additional hits that would not have been considered otherwise, namely the purine-like (PL) and 3,3-difluoro-2-azetidinone (DFA) compounds. This led to the confirmation of both PL_4 and PL_6 compounds as potential starting points for further investigations (Figs. S16, S17). Despite a low attractiveness of the other derivatives for further drug development perspective at this stage (lack of activity in infected macrophage assay), these compounds may still prove interesting from a mechanistic point of view. In particular, we showed here that PL_2 compound had bacteriostatic effects when combined with PAS, indicating that PAS-additivity may be explored further for this particular sub-group of PL derivatives. Also, we found that DFA compounds could benefit PAS and other antibiotics when used in combination, although the gains were minor. Further work would be needed to explore this in more details and identify additional derivatives with more pronounced additivity. Altogether the discovery of these compounds highlight the relevance of conducting such combination screening approach to unravel new chemical entities of interest, even among already analyzed chemical libraries.

Lastly, although this falls a bit outside the main scope of this work, we also report here additional information regarding the mechanism of action of the PP derivatives, with evidence that alteration in Rv3087 is strongly associated with resistance. Mutational experiments clearly indicated non-essentiality of the *rv3087* gene for bacterial growth, as predicted by multiple transposon mutagenesis experiments^25–28^. Absence of the gene or a functional copy of it led to 70 to 100-fold resistance as compared to the wild-type. Based on the protein annotation as a possible triacylglycerol synthase^29^, we propose that the compounds are activated through acylation of the free alcohol, but have been unable to confirm this so far using the recombinant protein. We hope to be able to publish more evidence when the currently on-going experiments involving additional PP derivatives are completed.

## Methods

### Compounds, reformatting and dilution

DMSO, rifampicin and PAS (catalog number A79604; lot number BCBS7659V) were purchased from Sigma-Aldrich Korea (Yongin, South Korea). Screening compounds were selected from commercial vendor libraries, based on novelty, drug likeness and absence of redundancy with compounds already tested internally in our laboratory^30^. A total of 104,783 small molecules were assembled, including 51,406 compounds from Enamine and 53,377 compounds from various suppliers: ChemDiv (12,270), ChemBridge (11,709), Life Chemicals (7917), Vitas-M (5000), Princeton BioMolecular Research (4751), InterBioScreen (3436), Otava Chemicals (3366), Thermo Fisher Scientific (Maybridge collection, 2951) and Specs (1977). Compounds were received plated in 96-well plates (mother plates); powders were resuspended in DMSO to prepare stock solution at 10 mM, where required. Compounds were reformatted in 384-well intermediate plates at a concentration of 2 mM in DMSO. All plates were sealed and stored frozen at − 20 °C until use. The day of the screening, intermediate plates were thawed and 0.5 μL of compounds dispensed in 384-well assay plates (catalog number 781091; Greiner Bio-One) using a high precision automatic dispenser (Analytic Jena). Plates were filled with 10 μL of the bacterial culture medium (as described below) prior to compound distribution. Each plate was prepared as duplicates; the first set was used to assess the activity of the compound alone, the second set to assess the activity of the compound in combination with PAS.

### Bacterial culture

*Mycobacterium tuberculosis* strain H37Rv (ATCC27294), hereafter referred to as H37Rv, was transformed with an integrative plasmid (pNIP48) bearing the coding sequence of a green fluorescent protein (GFP) in frame with a strong mycobacterial constitutive promoter (pBlaF), as previously described^19^. Positive transformants were selected using hygromycin (50 µg/mL, Invitrogen) and a single clone propagated to generate a stable GFP-expressing strain referred to as H37Rv-GFP. The reporter strain and other bacterial strains were grown in 7H9 medium (Invitrogen, Thermo Fisher Scientific) supplemented with 10% OADC (BD Biosciences), 0.05% Tween (Sigma-Aldrich), 0.5% glycerol (Sigma-Aldrich) and 50 µg/mL hygromycin B when necessary. Bacteria were grown for 14 days at 37 °C, 5% CO_2_ in ventilated Erlenmeyer flasks without shaking. Once a week, bacteria were diluted at OD_600 nm_ = 0.1 using fresh medium. This protocol allowed us to collect homogenous, planktonic cultures of bacteria, without aggregates.

### Primary screening

Due to the large volumes of cultures required for the screening, 2-week old bacterial cultures of H37Rv-GFP were directly diluted at OD_600 nm_ = 0.03 using fresh medium, containing 0.125 μM of PAS where required. Bacteria were distributed in 384-well assay plates (40 μL) using an automated dispenser (Wellmate, Thermo Fisher). As indicated above, assay plates contained the compounds diluted at 100 μM in 10 μL 7H9 complete medium. Under these conditions, the final concentration of PAS in the well was 0.1 μM and the compounds were tested at a final concentration of 20 μM. Each plate contained 32 wells for the positive control (1 μg/mL rifampicin) and 32 wells for the negative control (1% DMSO). Plates were incubated for 5 days at 37 °C, 5% CO_2_ and the relative fluorescence units (RFU) were recorded using a plate reader (Victor3, Perkin Elmer) equipped with filter sets centered at excitation (Ex.) 488 nm and emission (Em.) 535 nm. Compound RFU values were normalized with that of the aggregated positive and negative controls of the corresponding plates (plate-by-plate normalization). Assay quality was evaluated for each plate using the Z’ indicator^31^. Plates with a Z’ value below 0.5 were rejected and retested (1 plate was retested).

### Secondary screening

The confirmation of compound activity was done by dose–response (two-fold dilutions, 10 points) using the same extracellular assay as described above, where H37Rv-GFP bacteria were directly diluted and transferred to the assay plate, *i.e.* 40 μL of bacterial suspension were added to 10 μL of medium containing the compounds. Each dose–response was performed in duplicate and the data analyzed together. Fluorescence levels (RFU) were recorded using the same protocol as described above. Concentrations required to inhibit bacterial growth by 50% (IC_50_) were determined by least-square regression, fitting the data against a sigmoidal dose–response model (4 parameters), using GraphPad Prism version 6.0.2 for Windows, GraphPad Software, San Diego, California USA, www.graphpad.com. The 95% confidence interval (95% CI), corresponding to the estimated interval that has a 95% probability of containing values from repeated measurements, is reported. The calculated IC_50_ were compared between both sets using a fold change indicator calculated as below:

$$\mathrm{ln}\left(\mathrm{FC}\right)= \mathrm{ln}\frac{{IC}_{50}^{untreated}}{{IC}_{50}^{PAS-treated}}$$

Compounds were grouped in the *Additive* or *Antagonist* category when ln(FC) was greater than 0.5 or lower than − 0.5, respectively. Compounds falling in between were grouped in the *Independent* category. Compounds for which there was less than 50% reduction of bacterial growth at the highest dose (25 μM) were considered inactive. Using this definition, compounds that were inactive without PAS but showed activity with PAS were labelled as *Additive*. Compounds inactive with PAS but active alone were labelled as *Antagonist*. Compounds inactive in both sets were grouped in the *Inactive* category. Compound clustering and Structure Activity Landscape Index (SALI) plots were obtained with DataWarrior v5.2.1^32,33^, using the FragFp descriptor as similarity criterion. For re-synthesized compounds, the dose-responses were performed in duplicates in at least two independent experiments and the IC_50_ values indicated are the average ± SD for all these experiments.

### Macrophage culture and infection assay

Raw 264.7 murine macrophages were grown at 37 °C, 5% CO_2_ in RPMI 1640 medium (Welgene) supplemented with 10% heat-inactivated fetal bovine serum (FBS), referred to as RPMI-FBS medium. Cells were passaged every 2 or 3 days (70% confluence) and used between passages 2 and 9. The day of the infection, macrophage cells were treated with 1 × Versene (Gibco) for 10 min at 37 °C, gently detached using a cell-scraper, centrifuged at 300×*g* for 5 min and resuspended in fresh RPMI-FBS medium. Cells were enumerated using a Thoma cell counting chamber and suspensions at 1 × 10^6^ cells/mL were prepared. H37Rv-GFP Bacteria were harvested by centrifugation at 6000 × *g* for 10 min, washed twice with PBS and resuspended in RPMI-FBS at OD_600 nm_ = 0.2, which corresponded to a bacterial concentration of 2 × 10^7^/mL, as confirmed previously by CFU counting. One volume of bacterial suspension was mixed with one volume of cell to yield a multiplicity of infection (MOI) of 20 bacteria per cell and the mixture was incubated in an Erlenmeyer flask for 2 h at 37 °C with mild shaking (100 rpm). Infected cells were washed twice with fresh RPMI-FBS medium and plated at 20,000 cells/well in 384-well assay plates (catalog number 781091; Greiner Bio-One) containing two-fold dilutions of compounds in PBS (10 µL/well). After 5 days incubation at 37 °C, 5% CO_2_, Hoechst 33342 (Invitrogen) was added to reach a final concentration of 5 µM and plates were incubated for 20 min at 37 °C, 5% CO_2_ before imaging by fluorescence microscopy using a 20X objective (Operetta, Perkin Elmer). GFP bacteria were detected using a 488 nm laser excitation and cell nuclei using a 405 nm laser excitation. Three fields were recorded for each well and images were analyzed using the Columbus system (Perkin Elmer), to quantify the number of cells, the total surface of bacteria (as a number of pixels), the average surface of bacteria per infected cells and the ratio of infected cells. Values per well were average of the values obtained per field. IC_50_ values were determined for all 4 parameters by fitting the data against a sigmoidal dose–response model (4 parameters) by least-square regression. Final values were average of the IC_50_ values found for the 4 parameters, excluding values when the fit was not biologically relevant. Dose-responses were performed in duplicate and each experiment repeated at least twice.

### Colony forming units (CFU) assay

A 2-week old bacterial culture of H37Rv wild-type (WT) was diluted to reach an OD_600 nm_ = 0.1 (~ 10^7^ bacteria/mL) using fresh medium. Equal volumes (1 mL) of the same bacterial suspension were dispensed in 24-well plates containing the compound dilutions and the plates incubated at 37 °C, 5% CO_2_. At the corresponding time points, an aliquot of 100 μL was withdrawn from each well and serial tenfold dilutions were prepared in PBS (7 dilutions). For each dilution, a total volume of 10 μL was plated in several drops on solid 7H11 medium containing 0.5% glycerol and 10% OADC. All the plating were done in duplicate and the plates incubated at 37 °C, 5% CO_2_. Colonies were enumerated from 21 days and the counts regularly updated for up to 35 days of incubation. Counts were averaged for each condition and the results expressed in log_10_(CFU/mL), referring to the undiluted sample.

### Spontaneous resistant mutant selection and DNA extraction

Spontaneous resistant mutants to the pyrido[1,2-a]pyrimidin-4-one derivatives (PP_1 and PP_2) were raised by plating exponentially growing cultures of H37Rv wild-type (OD_600 nm_ ~ 1.0) on 7H11 agar containing 10% OADC, 0.5% glycerol and up to 20 µg/mL of PP_1 or PP_2. Plates were incubated 2 to 3 weeks and small individual colonies were repicked on fresh complete 7H11 agar containing 20 µg/mL PP_1 or PP_2 for 1 to 2 weeks. These large colonies were scrapped in 5 mL of 7H9 complete medium and cultured without antibiotic as described above to reach volumes of 50 mL at OD_600 nm_ ~ 1.0. Glycerol stocks were prepared by adding glycerol directly to culture aliquots (15% final) and freezing at − 80 °C. The rest of the bacteria were used for extraction of genomic DNA using the phenol–chloroform method. Briefly, bacteria were washed twice with water and resuspended in ice-cold TE buffer (500 μL for 10 mL culture at OD_600 nm_ ~ 1.0), lysed by bead beating for 10 min in presence of 100 μm silica beads and the clarified supernatant mixed with 500 μL of saturated Phenol/Chloroform/Isoamylalcohol (25/24/1) (Sigma-Aldrich). After centrifugation at 13,000 × *g* for 20 min, the aqueous (top) phase was collected, buffered with 50 μL of 3 M sodium acetate at pH 5.2, and mixed with 1.1 mL of absolute ethanol (Sigma-Aldrich, Korea). The genomic DNA was pelleted by centrifugation at 13,000 × *g* for 20 min, washed with 500 μL of 75% ethanol, dried at RT and stored at − 20 °C after resuspension in 100 μL of TE buffer.

### Construction of Rv3087 KO and over-expression strains

Rv3087 KO strains were obtained by allelic replacement using the method described by van Kessel and Hatfull^34^. Briefly, sequences of about 500 bp upstream (− 418 to 88 bp) and downstream (1323 to + 438 bp) of *rv3087* gene (1420 bp) were amplified by PCR from H37Rv genome and cloned in a pJSC backbone plasmid, on each side of the hygromycin resistance cassette. The plasmid was linearized by restriction and the substrate (3 kb) purified and electroporated in H37Rv bacteria previously transformed with the pJV53 plasmid and induced with acetamide for expression of the recombination proteins. Single colonies were isolated on agar containing hygromycin and verified by PCR to identify positive clones.

For the overexpression of Rv3087, the pVV16 plasmid was used as a backbone and the full-length *rv3087* gene cloned in frame of the strong Hsp60 constitutive promoter. Electro-competent bacterial cells were transformed with the pVV16::Rv3087 plasmid and selected on 7H11 agar medium containing kanamycin.

### Compound activity assessment using optical density and resazurin reduction assay

To test the activity of compounds against wild-type bacteria or mutants lacking GFP-reporter expression, bacterial growth after 5 days of incubation at 37 °C, 5% CO_2_ was determined by measuring the optical density (OD) at 600 nm directly from the assay plate (prepared as described above), using a plate reader (Victor3, Perkin Elmer). Subsequently, a sterile-filtered solution of 0.01% resazurin in PBS was prepared immediately before use and 10 μL were added to each well of the 384-well assay plate. The fluorescence of resorufin was recorded after 24 h incubation at 37 °C, 5% CO_2_, using filters Ex. 535 nm and Em. 590 nm. IC_50_ values were estimated by non-linear regression as described above.

### Chemistry

Synthesis of the pyrido[1,2-a]pyrimidin-4-one derivatives (PP_1 and PP_2) was performed as described in the related patent^20^. All other chemical synthesis were performed by GVK Biosciences, India. Reagents and solvents obtained from commercial suppliers were used without purification or drying unless otherwise noted. ^1^H NMR spectra were recorded at 300 MHz or 400 MHz (Bruker). TMS was used as an internal standard. Purity were determined by LC–MS analyses (Acquity, Waters) using a UPLC BEH C-18 (2.1 × 50 mm, 1.7 μm) column heated at 35 °C, with mobile phase A: 0.05% TFA in water; mobile phase-B: 0.05% TFA in acetonitrile (ACN); gradient (T/% B): 0/30, 3.5/90, 5/90, 5.1/30. The flow rate was 0.4 mL/min and compounds were diluted in ACN:water (70:30 v/v) prior to analysis. All analytical data (NMR spectra, HPLC and LC–MS data) were gathered as a single Appendix available online as supplementary information.

#### General procedure for the synthesis of 3,3-difluoro-2-azetidinone (DFA) derivatives (3a–3f)


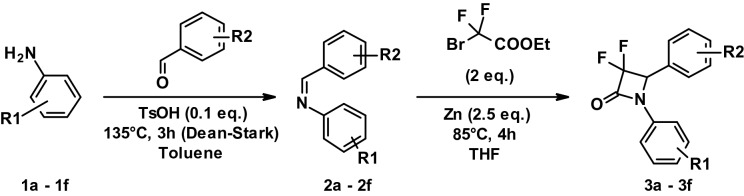


To a stirred solution of **1a**–**f** (1.2 eq.) and the corresponding aldehyde (8.058 mmol) in toluene, 0.1 eq. of *para*-toluenesulfonic acid (TsOH) was added and the mixture refluxed at 135 °C for 3 h using a Dean-Stark apparatus. The reaction was followed by thin-layer chromatography using 10% ethyl acetate (EtOAc) in petroleum ether (PE) as eluent and stopped when all the aldehyde was consumed. The reaction mixture was cooled down and concentrated under vacuum. The desired product was purified by column chromatography using silica gel (100–200 mesh) and eluted using 5% EtOAc in PE. Pure fractions were concentrated under vacuum to afford **2a**–**f** as off-white solids (yields 28–41%).

To a stirred solution of Zinc (2.5 eq.) in THF (30 mL) was added **2a**–**f** (2.072 mmol) and ethyl bromodifluoro acetate (2 eq.) in THF (20 mL). The mixture was refluxed at 85 °C for 4 h and the reaction progress followed by thin-layer chromatography using 10% EtOAc in PE as eluent. The reaction mixture was cooled down and concentrated under vacuum. The desired product was purified by column chromatography using silica gel (100–200 mesh) and eluted using 5% EtOAc in PE. Pure fractions were concentrated under vacuum to afford **3a**-**f** as off-white solids (yields 28–61%).

#### General procedure for the synthesis of purine-like (PL) derivatives (6a–6f)


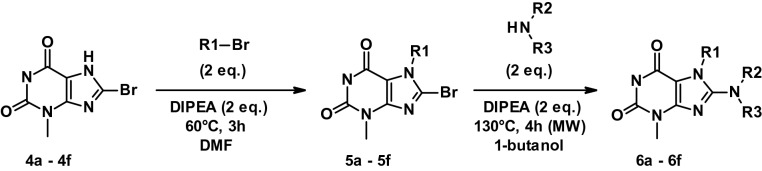


To a stirred solution of **4a**-**f** (2.040 mmol) and N, N-diisopropylethylamine (DIPEA, 2 eq.) in dimethylformamide (DMF, 25 mL) was added the corresponding brominated alkane or ketone (2 eq.) and the reaction mixture heated at 60 °C for 3 h. The reaction was followed by thin-layer chromatography using 10% EtOAc in PE as eluent and stopped when all the starting material (**4a**-**f**) was consumed. The reaction mixture was cooled down and concentrated under vacuum. The desired product was purified by column chromatography with silica gel (100–200 mesh) and eluted using 5% EtOAc in PE. Pure fractions were concentrated under vacuum to afford **5a**–**f** as off-white solids (yields 53–77%).

To a stirred solution of **5a**-**f** (0.796 mmol) and DIPEA (2 eq.) in 1-butanol (20 mL) was added the corresponding amine (2 eq.) and the mixture microwave irradiated at 130 °C for 4 h. The reaction was followed by thin-layer chromatography using 50% EtOAc in PE as eluent and stopped when all the starting material (**5a**–**f**) was consumed. The reaction mixture was cooled down and concentrated under vacuum. The desired product was purified by column chromatography with silica gel (100–200 mesh) and eluted using 30% EtOAc in PE. Pure fractions were concentrated under vacuum to afford **6a**-**f** as off-white solids (yields 24–88%).

#### Preparation of the amine intermediate for synthesis of PL_4


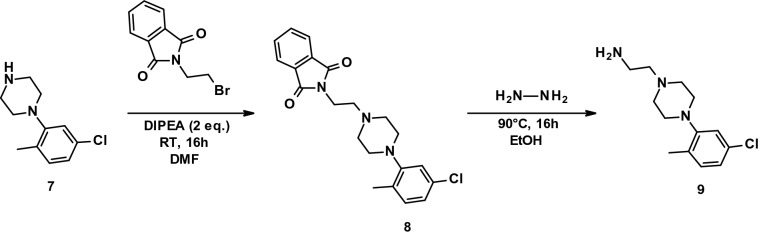


To a stirred solution of N-(2-Bromoethyl)phthalimide (0.6 g, 2.36 mmol) in DMF (12 mL) was added DIPEA (0.82 mL, 2 eq.) and the reaction mixture was stirred at RT for 10 min before addition of **7** (1.5 eq.). The reaction was further stirred at RT for 16 h. The reaction was followed by thin-layer chromatography using 20% EtOAc in hexane as eluent, until depletion of the starting material (Rf: 0.3, UV active). After completion, ice cold water (80 mL) was added and the mixture extracted with EtOAc (2 × 50 mL). Combined organic layers were dried over Na_2_SO_4_ and under vacuum. The desired product was purified by column chromatography with silica gel (100–200 mesh) and eluted using 10% EtOAc in PE. Pure fractions were combined and dried under vacuum to afford **8** as a yellow solid (yield 66%).

To a stirred solution of **8** (0.5 g, 1.3 mmol) in ethanol (EtOH, 7 mL) was added hydrazine hydrate (3 mL) at RT and the reaction mixture was stirred for 16 h at 90 °C. The reaction was followed by thin-layer chromatography using 10% methanol in dichlroromethane (DCM) as eluent and stopped after depletion of **8** (Rf: 0.3, UV active). The ethanol was distilled off completely under vacuum and the obtained solid washed with water (20 mL) to recover the water-soluble product in the aqueous layer. The aqueous layer was distilled off completely, co-distilled with ACN (10 mL) and dried under vacuum to afford **9** as an off-white solid (yield 90%).

#### Procedure for the preparation of DCP_3 (13)


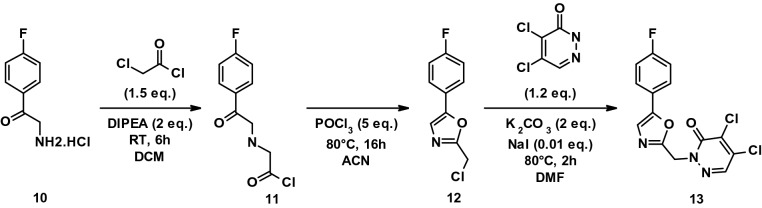


To a stirred solution of **10** (1.6 g, 8.438 mmol) in DCM (10 mL) cooled at 0 °C, DIPEA (2.17 g, 16.876 mmol) was added and the mixture stirred for 10 min before dropwise addition of chloroacetyl chloride (1.24 mL, 12.65 mmol) at 0 °C. The reaction was stirred for 6 h at RT. The reaction was followed by thin-layer chromatography using 50% EtOAc in PE as eluent, until depletion of the starting material (Rf = 0.6). The reaction mixture was poured into ice cold water (100 mL) and extracted with DCM (2 × 50 mL). The organic layer was dried over anhydrous Na_2_SO_4_, filtered and concentrated under reduced pressure. The residue was triturated with PE (20 mL) and dried to afford **11** as a brown solid (2 g, crude).

To a stirred solution of **11** (2 g, 8.709 mmol) in ACN (40 mL) at RT, phosphoryl chloride (POCl_3_, 4 mL, 43.546 mmol) was added dropwise and the reaction mixture heated at 80 °C for 16 h. The reaction was followed by thin-layer chromatography using 20% EtOAc in PE as eluent, until depletion of the starting material (Rf = 0.5). The reaction mixture was poured into ice-cold water (100 mL) and extracted with EtOAc (2 × 100 mL). The organic layer was dried over anhydrous Na_2_SO_4_, filtered and concentrated under reduced pressure. The desired product was purified by column chromatography with silica gel (100–200 mesh) and eluted using 10% EtOAc in PE to afford **12** as a brown liquid (yield 61%).

To a stirred solution of **12** (350 mg, 1.653 mmol) and 4,5-dichloro-1H-pyridazin-6-one (DCP, 327 mg, 1.9846 mmol) in DMF (10 mL) was added K_2_CO_3_ (456 mg, 3.306 mmol) and NaI (3 mg, 0.0213 mmol). The reaction mixture was heated at 80 °C and stirred for 2 h. The reaction was followed by thin-layer chromatography using 20% EtOAc in PE as eluent, until depletion of the starting material (Rf = 0.2). The reaction mixture was poured into ice-cold water (100 mL) and extracted with EtOAc (2 × 50 mL). The organic layer was dried over anhydrous Na_2_SO_4_, filtered and concentrated under reduced pressure. The desired product was purified by column chromatography with silica gel (100–200 mesh) and eluted using 15% EtOAc in PE to afford **13** as a yellow solid (yield 36%).

#### Procedure for the preparation of DCP_7 (16)


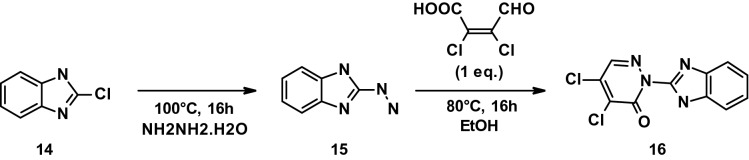


A solution of **14** (0.5 g, 3.27 mmol) in hydrazine hydrate (3.28 mL) was stirred at 100 °C for 16 h. The reaction was followed by thin-layer chromatography using EtOAc as eluent, until depletion of the starting material (Rf = 0.1). After completion, ice-cold water (25 mL) was added and the mixture stirred for 15 min. The precipitated solid was filtered off and dried under vacuum to afford **15** as a light brown solid (yield 61%).

To a stirred solution of **15** (0.3 g, 2.02 mmol) in EtOH (9.5 mL), (Z)-2,3-dichloro-4-oxobut-2-enoic acid (0.38 g, 2.02 mmol) was added and the reaction mixture stirred at 80 °C for 16 h. The reaction was followed by thin-layer chromatography using EtOAc as eluent, until depletion of the starting material (Rf = 0.5). After completion of the reaction, EtOH was distilled off and the mixture dried under vacuum. The desired product was purified by preparative HPLC using a gradient of ACN in water (containing 0.05% TFA) as eluent. Pure fractions were collected and the solvent evaporated under reduced pressure to afford **16** as a pale yellow solid (yield 14%).

## Supplementary Information

Supplementary Information 1.

Supplementary Information 2.

## Data Availability

The raw screening data, including structures and activity results, can be made available on reasonable request to the authors following signature of a collaboration agreement.
