# Supplementary material for: A combination screening to identify enhancers of para-aminosalicylic acid against Mycobacterium tuberculosis
Source: Sci Rep. 2022 Apr 4;12:5635. doi: 10.1038/s41598-022-08209-w (PMC8980009; doi:10.1038/s41598-022-08209-w)

A combination screening to identify enhancers of *para*-aminosalicylic acid against *Mycobacterium tuberculosis*

Appendix. NMR, HPLC and LC-MS data for the re-synthesized compounds

Jinyeong Heo[1], Dahae Koh[1], Minjeong Woo[2], Doyoon Kwon[2], Virginia Carla de Almeida Falcão[2], Connor Wood[2], Honggun Lee[1], Kideok Kim[1], Inhee Choi[3], Jichan Jang[4], Priscille Brodin[5], David Shum[1] and Vincent Delorme[2]#

1. Institut Pasteur Korea, Screening Discovery Platform, Seongnam, Gyeonggi, 13488 Republic of Korea

2. Institut Pasteur Korea, Tuberculosis Research Laboratory, Seongnam, Gyeonggi, 13488 Republic of Korea

3. Institut Pasteur Korea, Medicinal Chemistry, Seongnam, Gyeonggi, 13488 Republic of Korea

4. Molecular Mechanisms of Antibiotics, Division of Life Science, Research Institute of Life Science, Division of Applied Life Science (BK21plus Program), Gyeongsang National University, Jinju, 52828 Republic of Korea

5. University of Lille, CNRS, INSERM, CHU Lille, Institut Pasteur de Lille, U1019 - UMR 9017 - CIIL - Center for Infection and Immunity of Lille, Lille, France

# Address correspondence to: vincent.delorme@ip-korea.org.

Contents:

- DFA compounds **3a**-**3f** (DFA_1 to DFA_6)
- PL compounds **6a**-**6f** (PL_1 to PL_6)
- DCP compounds **13** and **16** (DCP_3 and DCP_7)

DFA_1 (3a) 3

DFA_2 (3b) 11

DFA_3 (3c) 20

DFA_5 (3e) 36

DFA_6 (3f) 45

PL_1 (6a) 54

PL_2 (6b) 64

PL_3 (6c) 74

PL_4 (6d) 84

PL_5 (6e) 94

PL_6 (6f) 104

DCP_3 (13) 114

DCP_7 (16) 121

DFA_1 (3a)

S.No	Test	Results
1	**Description**	White Solid
2	**Identification**<br><br>(a) **NMR**<br><br>(b) **Mass by LCMS**	 <br>Complies to structure<br><br>278.23[M+H]$^+$
3	**Chromatographic Purity by UPLC (Area %)**<br>Impurities>1.0%	99.86<br>Nil
4	**Chromatographic Purity by LCMS (Area %)**<br>Impurities>1.0%	99.46<br>Nil

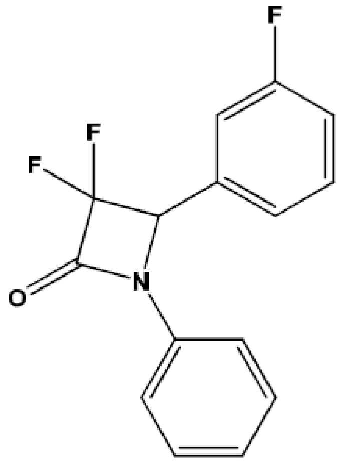

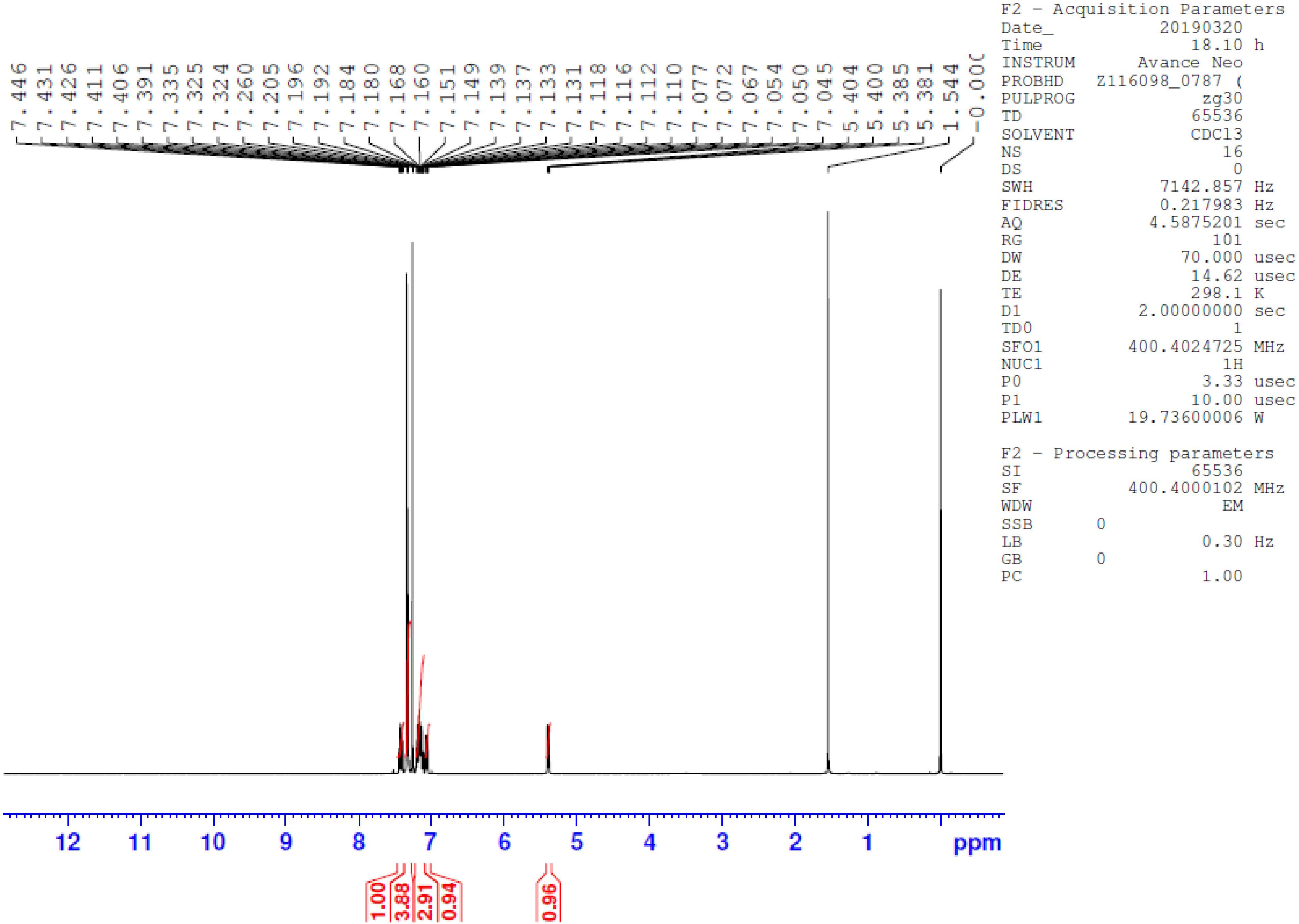
7.446
7.431
7.426
7.411
7.406
7.391
7.335
7.325
7.324
7.260
7.205
7.196
7.192
7.184
7.180
7.168
7.160
7.151
7.149
7.139
7.137
7.133
7.131
7.118
7.116
7.112
7.110
7.077
7.072
7.067
7.054
7.050
7.045
5.404
5.400
5.385
5.381
1.544
-0.00C
F2 - Acquisition Parameters
Date_ 20190320
Time 18.10 h
INSTRUM Avance Neo
PROBHD Z116098_0787 (
PULPROG zg30
TD 65536
SOLVENT CDCl3
NS 16
DS 0
SWH 7142.857 Hz
FIDRES 0.217983 Hz
AQ 4.5875201 sec
RG 101
DW 70.000 usec
DE 14.62 usec
TE 298.1 K
D1 2.00000000 sec
TD0 1
SFO1 400.4024725 MHz
NUC1 1H
P0 3.33 usec
P1 10.00 usec
PLW1 19.73600006 W
F2 - Processing parameters
SI 65536
SF 400.4000102 MHz
WDW EM
SSB 0
LB 0.30 Hz
GB 0
PC 1.00
12
11
10
9
8
7
6
5
4
3
2
1
ppm
1.00
3.88
2.91
0.94
0.96

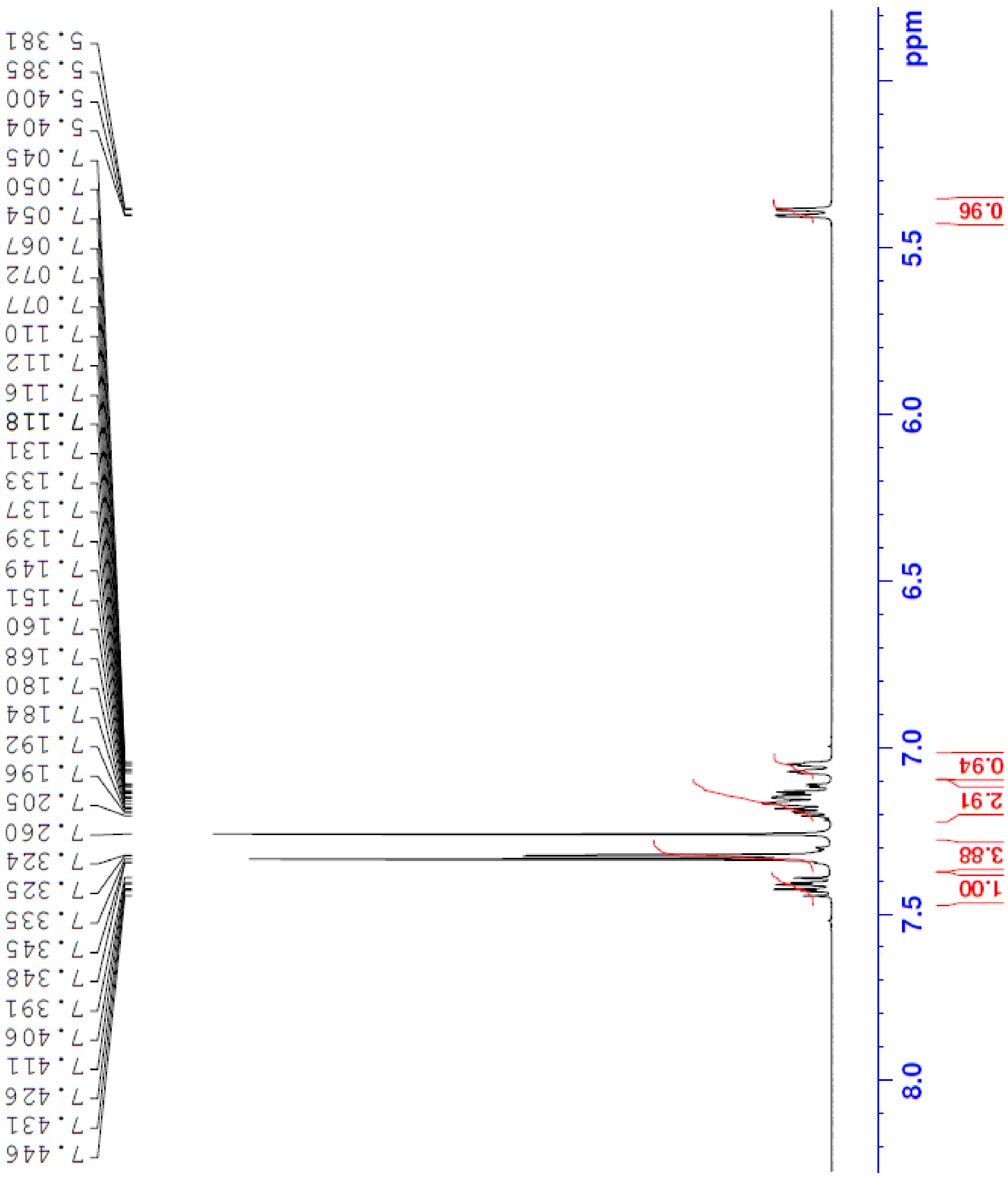
5.381
5.385
5.400
5.404
7.045
7.050
7.054
7.067
7.072
7.077
7.110
7.112
7.116
7.118
7.131
7.133
7.137
7.139
7.149
7.151
7.160
7.168
7.180
7.184
7.192
7.196
7.205
7.260
7.324
7.325
7.335
7.345
7.348
7.391
7.406
7.411
7.426
7.431
7.446
ppm
5.5
6.0
6.5
7.0
7.5
8.0
0.96
0.94
2.91
3.88
1.00

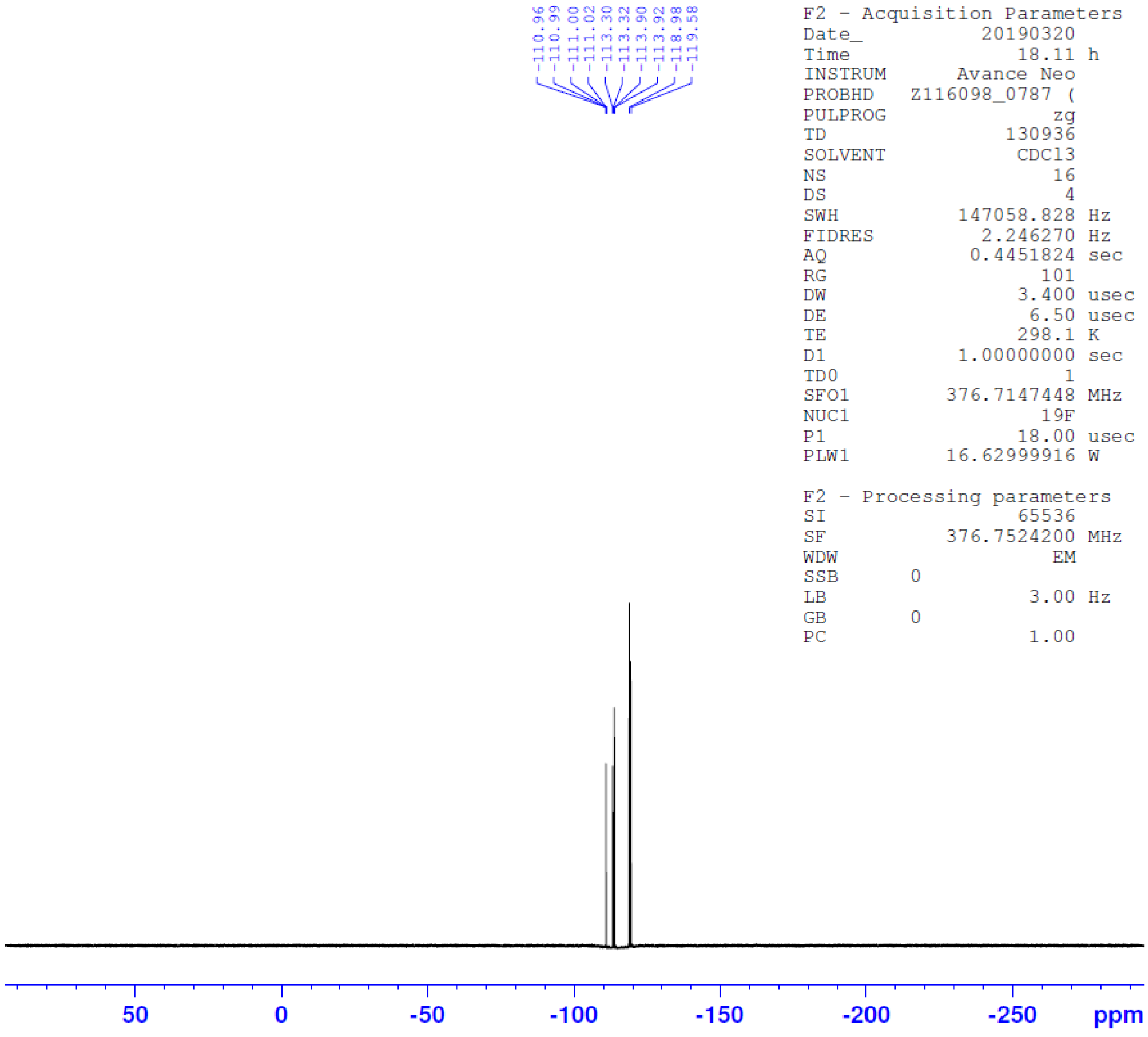

F2 - Acquisition Parameters

Date_	20190320	
Time	18.11	h
INSTRUM	Avance Neo	
PROBHD	Z116098_0787 (	
PULPROG	zg	
TD	130936	
SOLVENT	CDCl3	
NS	16	
DS	4	
SWH	147058.828	Hz
FIDRES	2.246270	Hz
AQ	0.4451824	sec
RG	101	
DW	3.400	usec
DE	6.50	usec
TE	298.1	K
D1	1.00000000	sec
TD0	1	
SFO1	376.7147448	MHz
NUC1	19F	
P1	18.00	usec
PLW1	16.62999916	W

F2 - Processing parameters

SI	65536	
SF	376.7524200	MHz
WDW	EM	
SSB	0	
LB	3.00	Hz
GB	0	
PC	1.00	

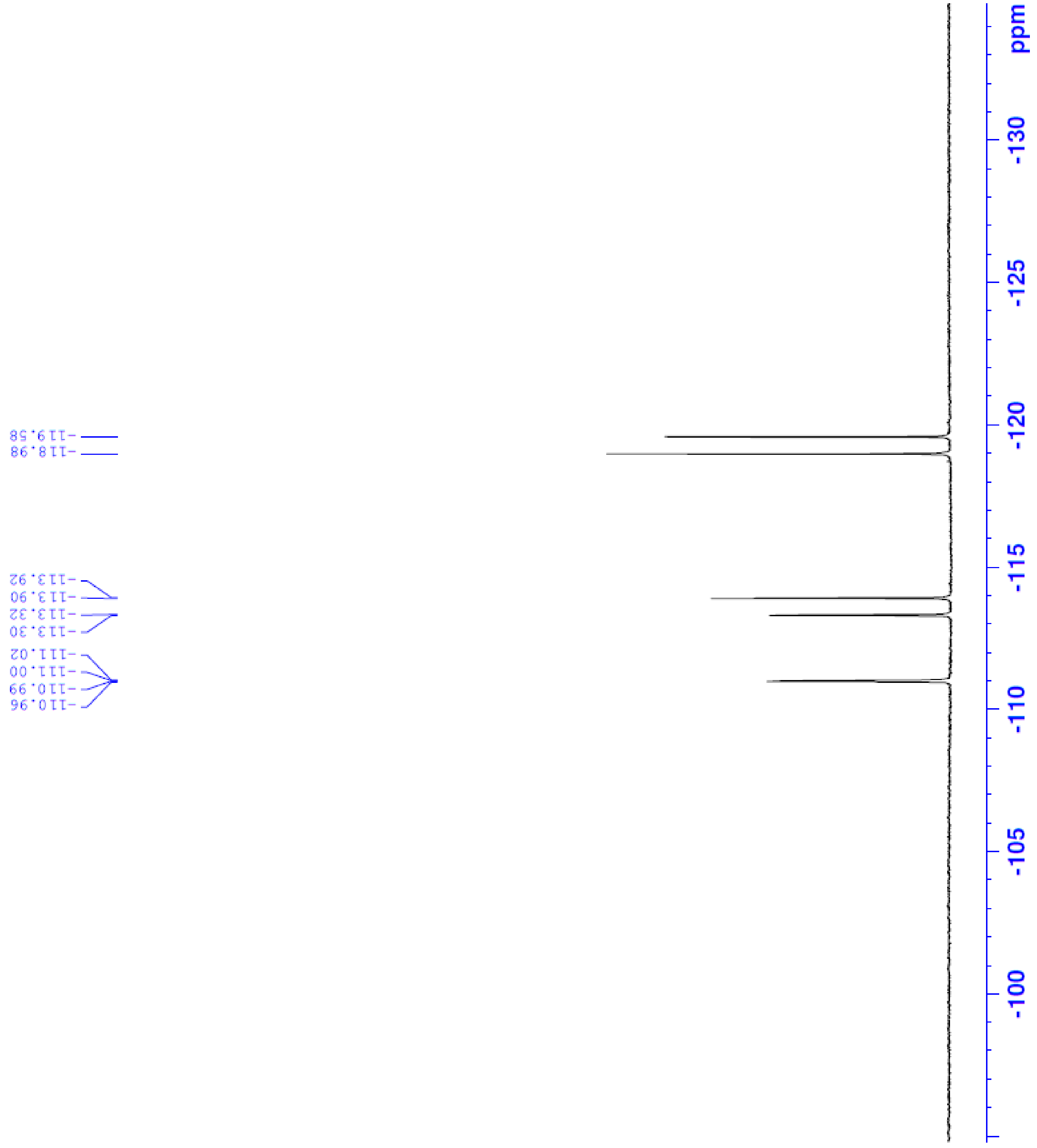
-119.58
-118.98
-113.92
-113.90
-113.32
-113.30
-111.02
-111.00
-110.99
-110.96
ppm
-130
-125
-120
-115
-110
-105
-100

UPLC Method Conditions :

Column	: Acquity UPLC BEH C18 (2.1x100) mm, 1.7µm
Mobile Phase-A	: 0.05% TFA in Water
Mobile Phase-B	: 0.05% TFA in Acetonitrile
Gradient (T/% B)	: 0/50,4/90,6/90,6.1/50
Flow Rate	: 0.3 mL/min
Temparature	: 40 °C
Diluent	: ACN+Water

Auto-Scaled Chromatogram

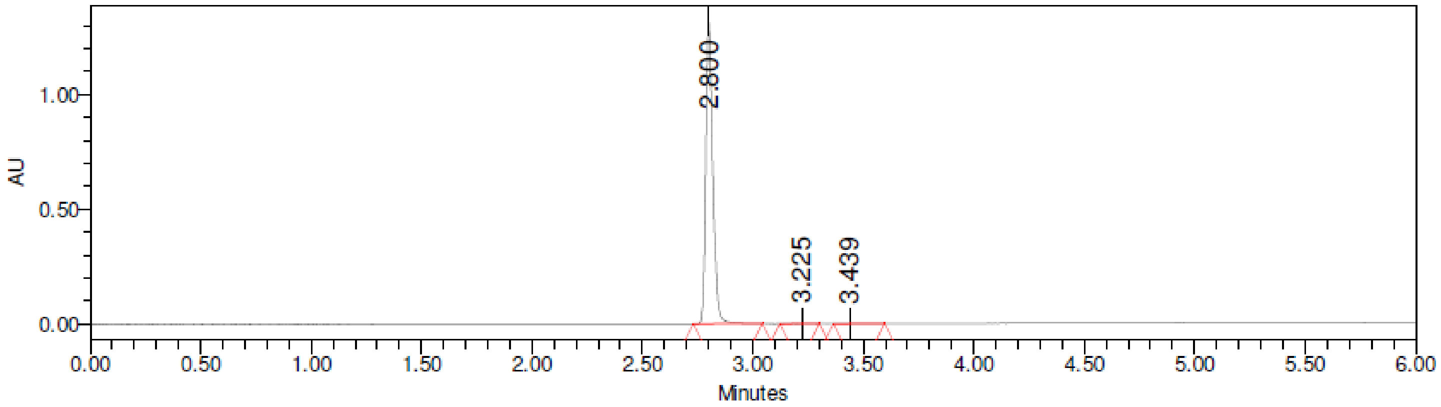

Peak Results

	RT	Area	Height	% Area
1	2.800	2913212	1320699	99.86
2	3.225	1588	448	0.05
3	3.439	2457	445	0.08

GVK Biosciences Private Limited
Discovery Chemistry-Analytical Services
================================

Sample ID:FAN Cluster-1 (C3804-129A2) **Date of analysis : 20-Mar-2019/19:00:41**
Acq Method :ATR-2 **Instrument ID: ANL-MCL2-LCMS-001**
1:B,2

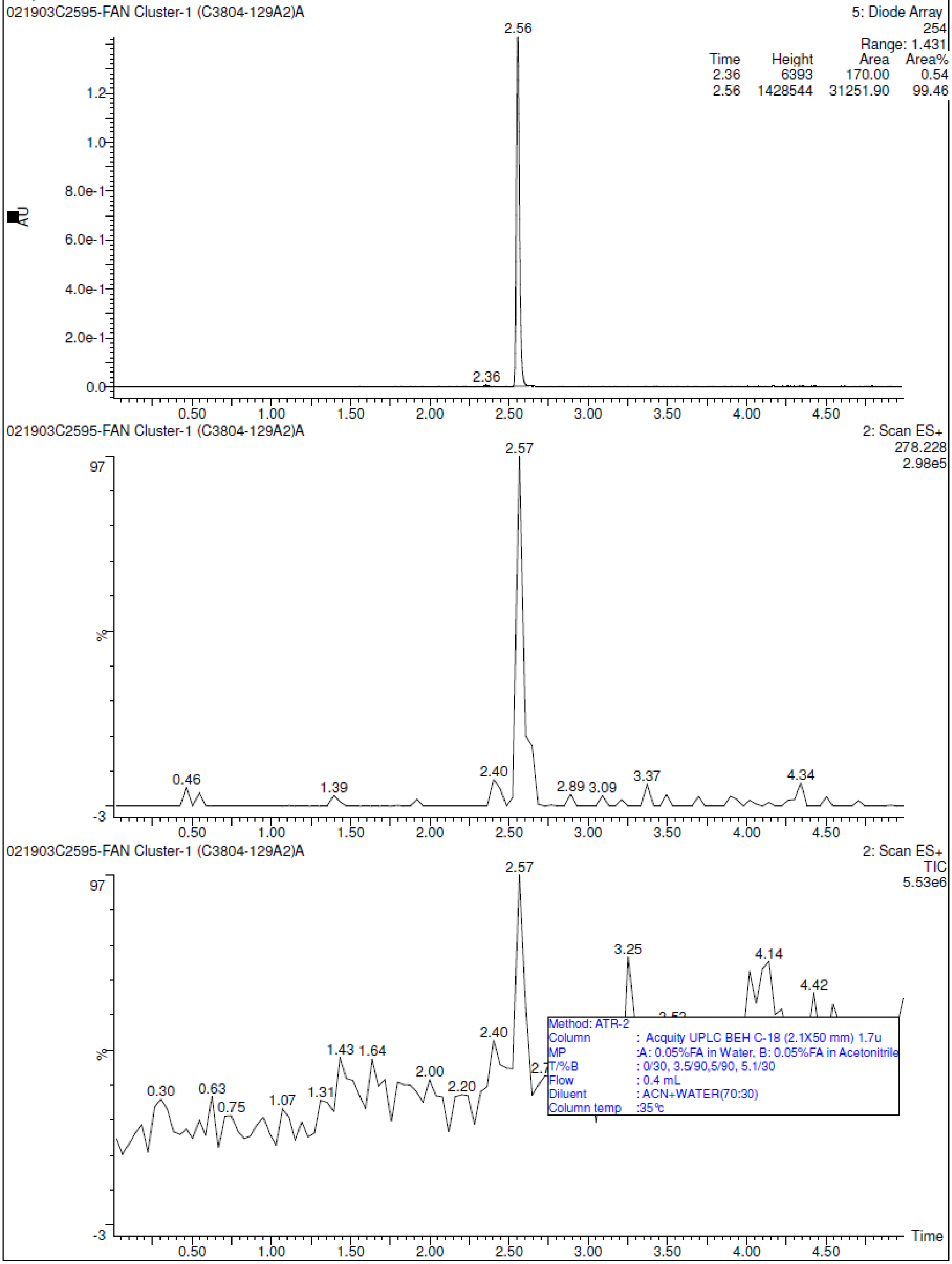

GVK Biosciences Private Limited
Discovery Chemistry-Analytical Services
================================

Sample ID:FAN Cluster-1 (C3804-129A2) **Date of analysis: 20-Mar-2019:19:00:41**
Acq Method :ATR-2 **Instrument ID:ANL-MCL2-LCMS-001**
1:B,2

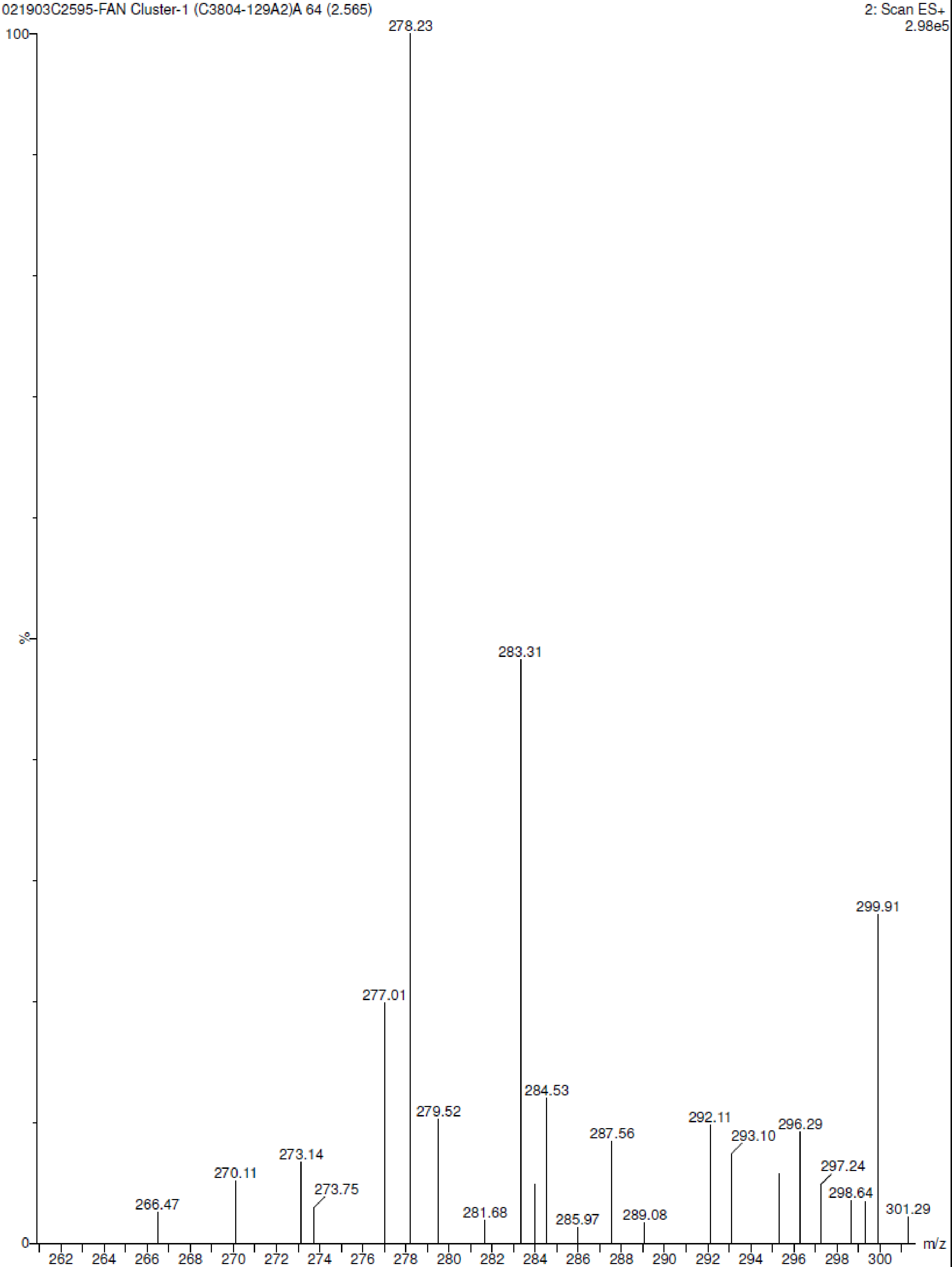

DFA_2 (3b)

S.No	Test	Results
1	**Description**	White Solid
2	**Identification**<br><br>(a) **NMR**<br><br>(b) **Mass by LCMS**	 <br>Complies to structure<br><br>308.20[M+H]$^+$
3	**Chromatographic Purity by UPLC (Area %)**<br>Impurities>1.0%	99.59<br>Nil
4	**Chromatographic Purity by LCMS (Area %)**<br>Impurities>1.0%	99.30<br>Nil

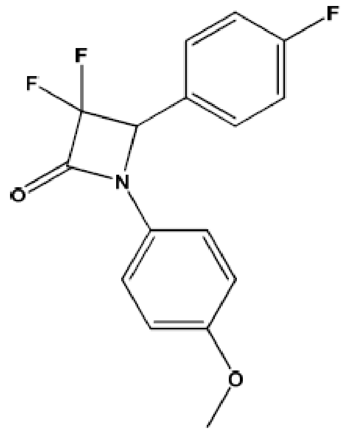

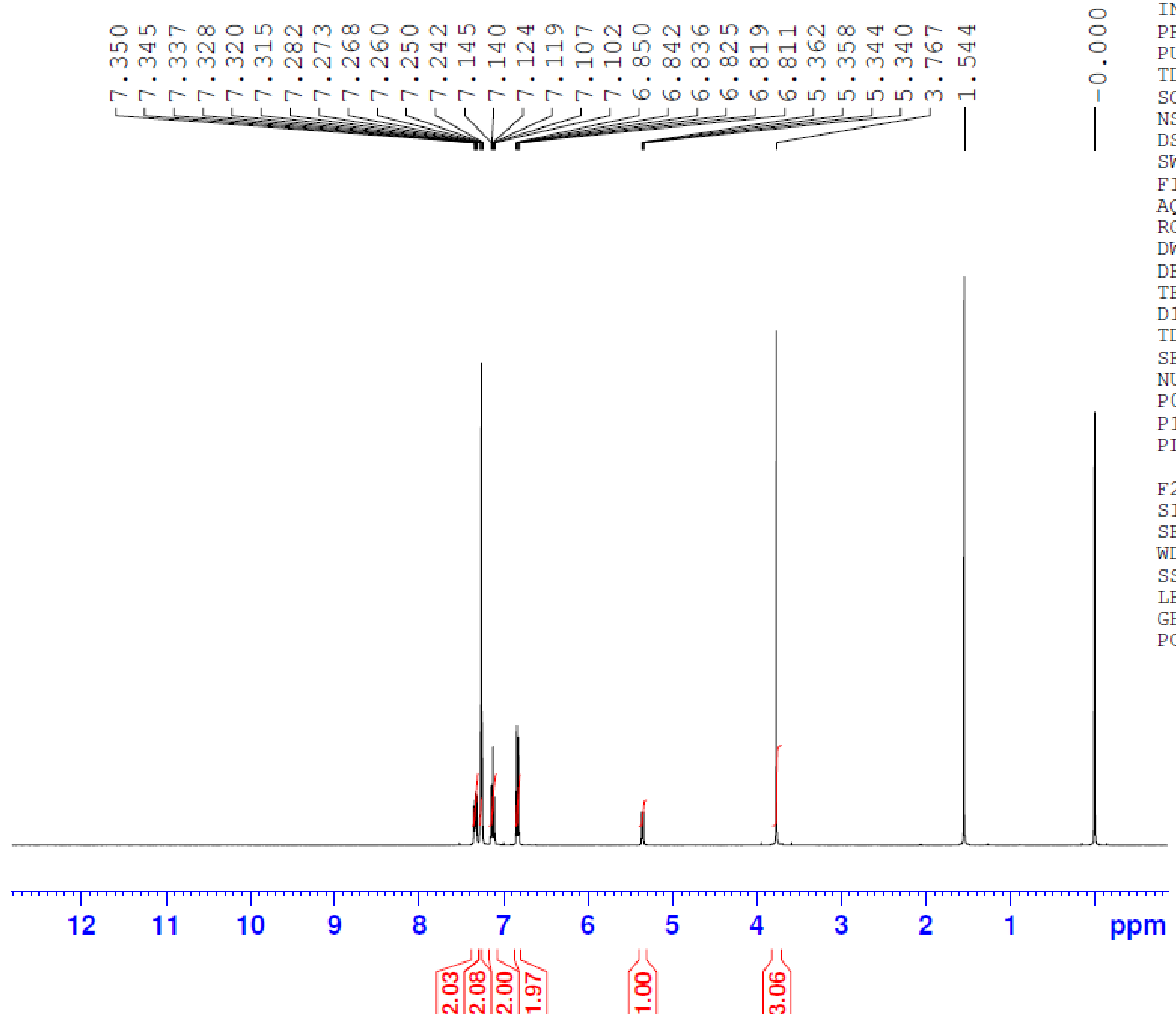

F2 - Acquisition Parameters

Date_	20190320
Time	18.28 h
INSTRUM	Avance Neo
PROBHD	Z116098_0787 (
PULPROG	zg30
TD	65536
SOLVENT	CDCl3
NS	16
DS	0
SWH	7142.857 Hz
FIDRES	0.217983 Hz
AQ	4.5875201 sec
RG	101
DW	70.000 usec
DE	14.62 usec
TE	298.1 K
D1	2.00000000 sec
TD0	1
SFO1	400.4024725 MHz
NUC1	1H
P0	3.33 usec
P1	10.00 usec
PLW1	19.73600006 W

F2 - Processing parameters

SI		65536
SF		400.4000101 MHz
WDW		EM
SSB	0	
LB		0.30 Hz
GB	0	
PC		1.00

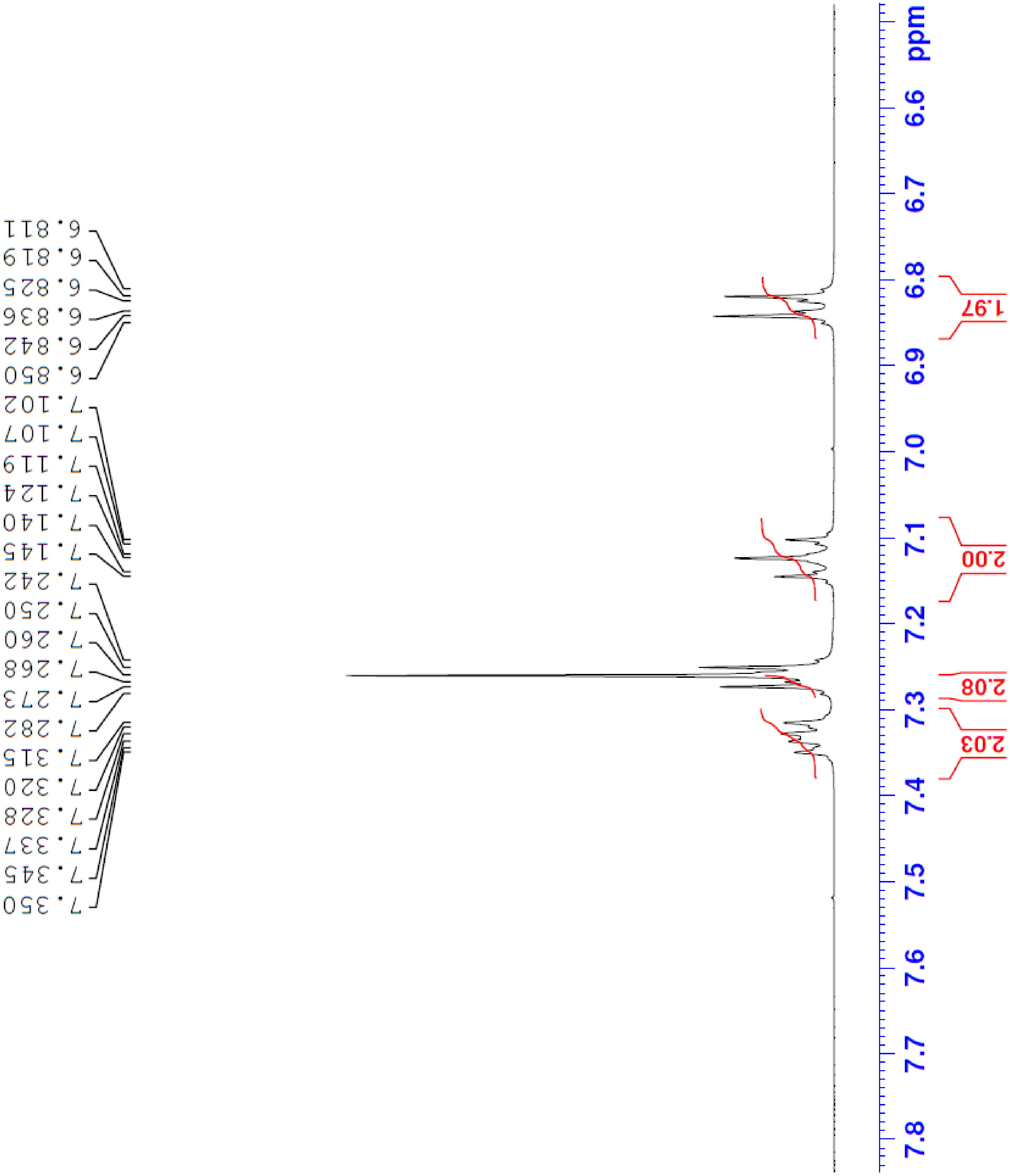
6.811
6.819
6.825
6.836
6.842
6.850
7.102
7.107
7.119
7.124
7.140
7.145
7.242
7.250
7.260
7.268
7.273
7.282
7.315
7.320
7.328
7.337
7.345
7.350
ppm
6.6
6.7
6.8
6.9
7.0
7.1
7.2
7.3
7.4
7.5
7.6
7.7
7.8
1.97
2.00
2.08
2.03

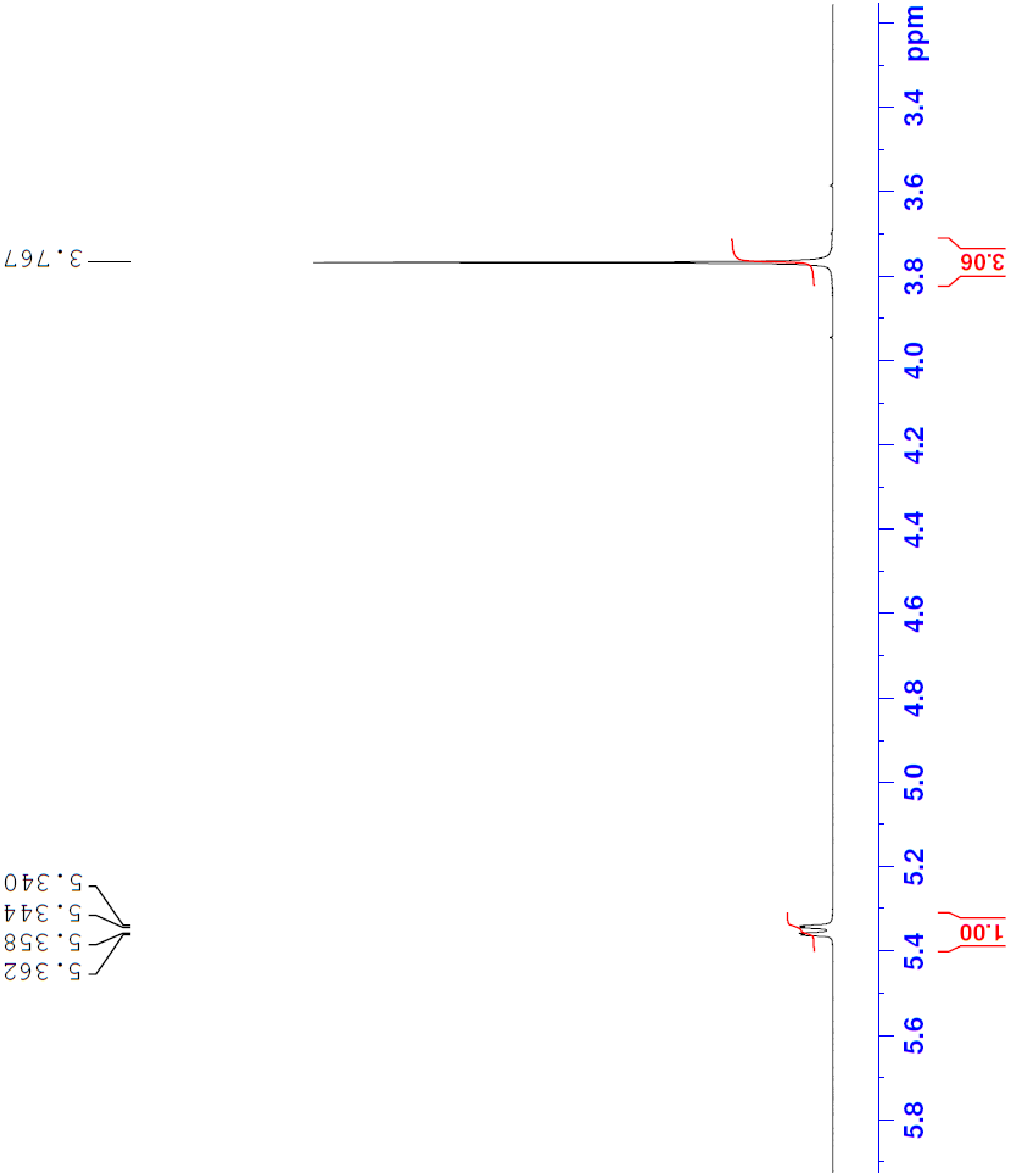
3.767
5.340
5.344
5.358
5.362
3.06
1.00
ppm
3.4
3.6
3.8
4.0
4.2
4.4
4.6
4.8
5.0
5.2
5.4
5.6
5.8

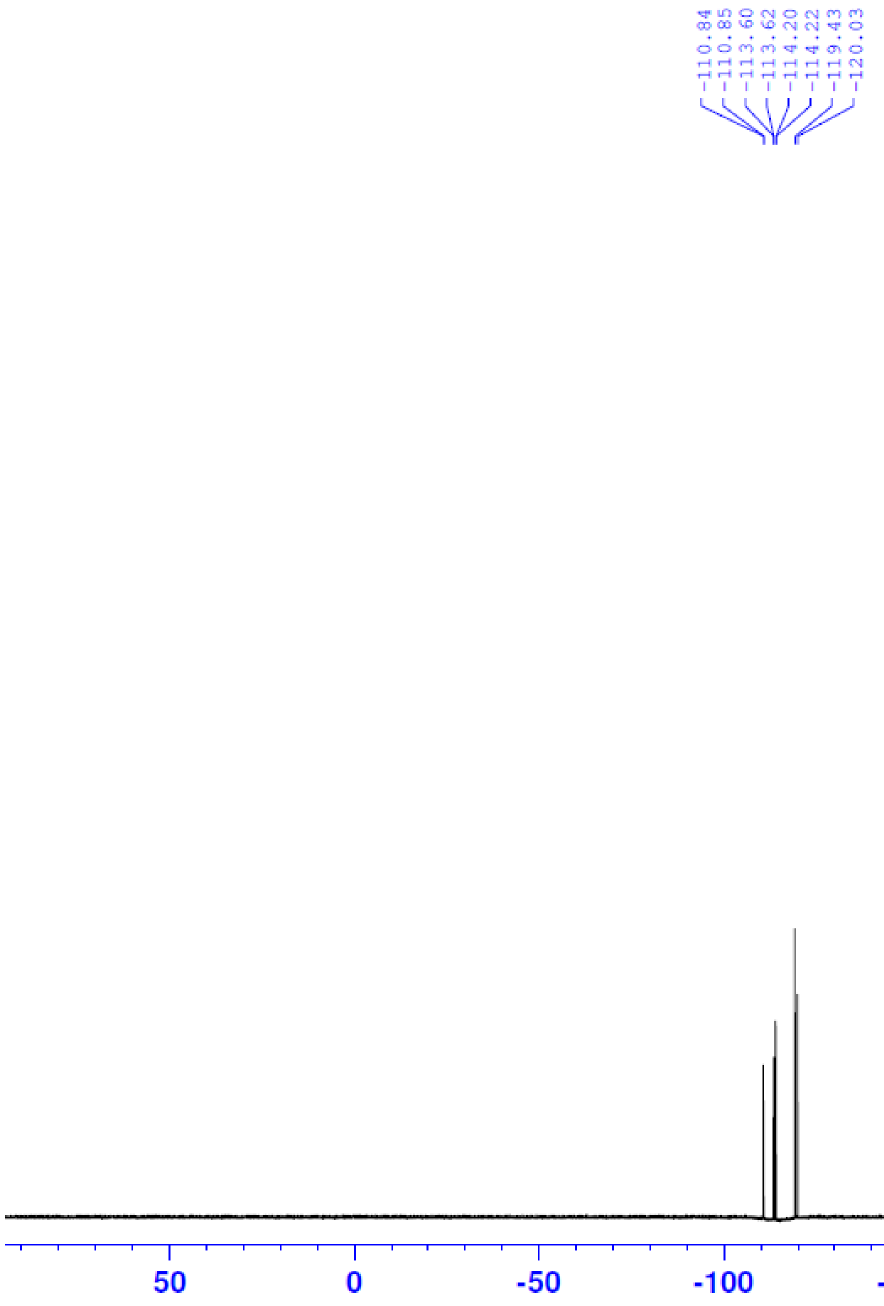

F2 - Acquisition Parameters
Date_ 20190320
Time 18.29 h
INSTRUM Avance Neo
PROBHD Z116098_0787 (
PULPROG zg
TD 130936
SOLVENT CDCl3
NS 16
DS 4
SWH 147058.828 Hz
FIDRES 2.246270 Hz
AQ 0.4451824 sec
RG 101
DW 3.400 usec
DE 6.50 usec
TE 298.1 K
D1 1.00000000 sec
TD0 1
SFO1 376.7147448 MHz
NUC1 19F
P1 18.00 usec
PLW1 16.62999916 W

F2 - Processing parameters
SI 65536
SF 376.7524200 MHz
WDW EM
SSB 0
LB 3.00 Hz
GB 0
PC 1.00

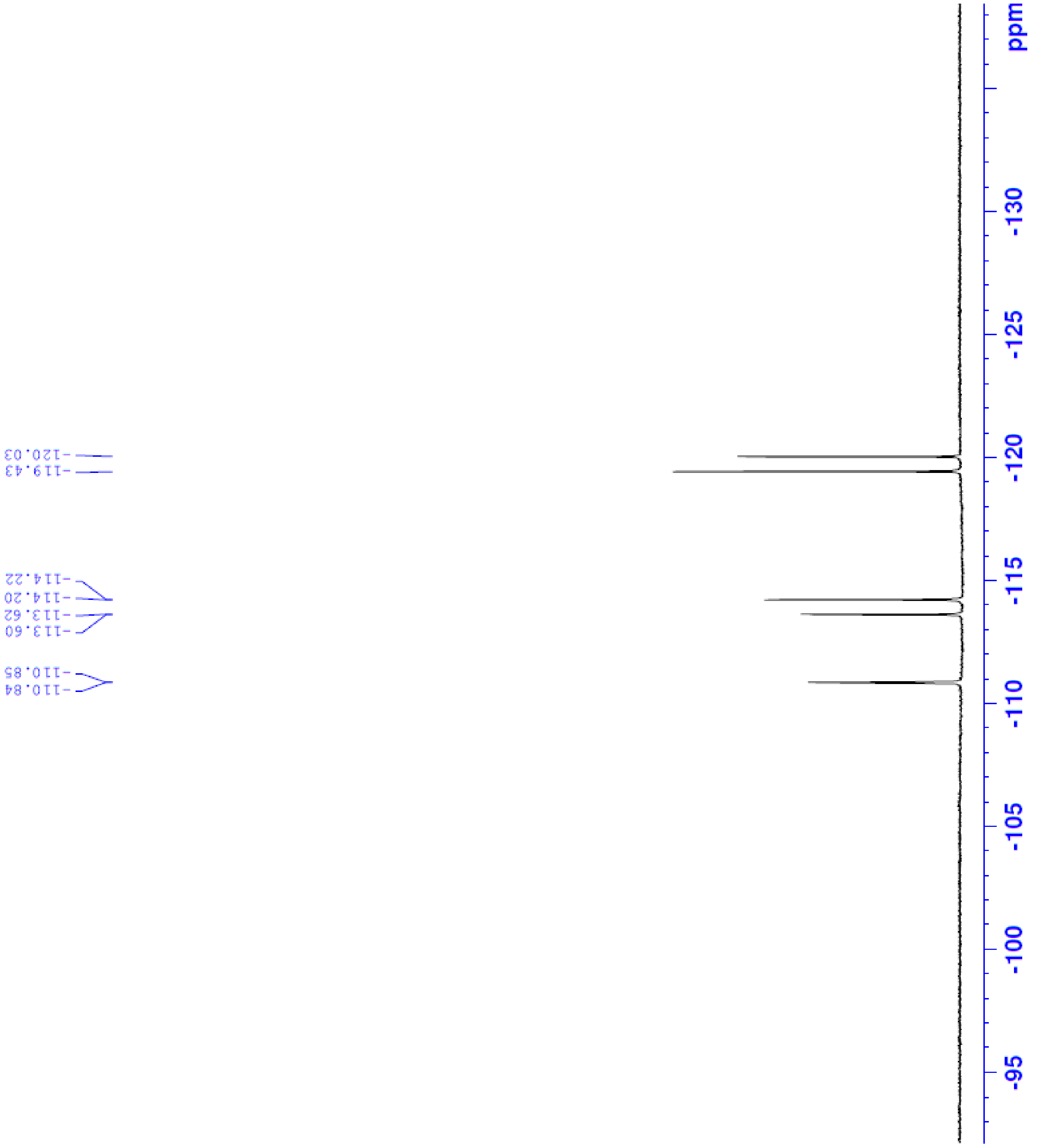

-120.03
-119.43
-114.22
-114.20
-113.62
-113.60
-110.85
-110.84
ppm
-130
-125
-120
-115
-110
-105
-100
-95

UPLC Method Conditions :

Column : Acquity UPLC BEH C18 (2.1x100) mm, 1.7µm
Mobile Phase-A : 0.05% TFA in Water
Mobile Phase-B : 0.05% TFA in Acetonitrile
Gradient (T/% B) : 0/50,4/90,6/90,6.1/50
Flow Rate : 0.3 mL/min
Temparature : 40 °C
Diluent : ACN+Water

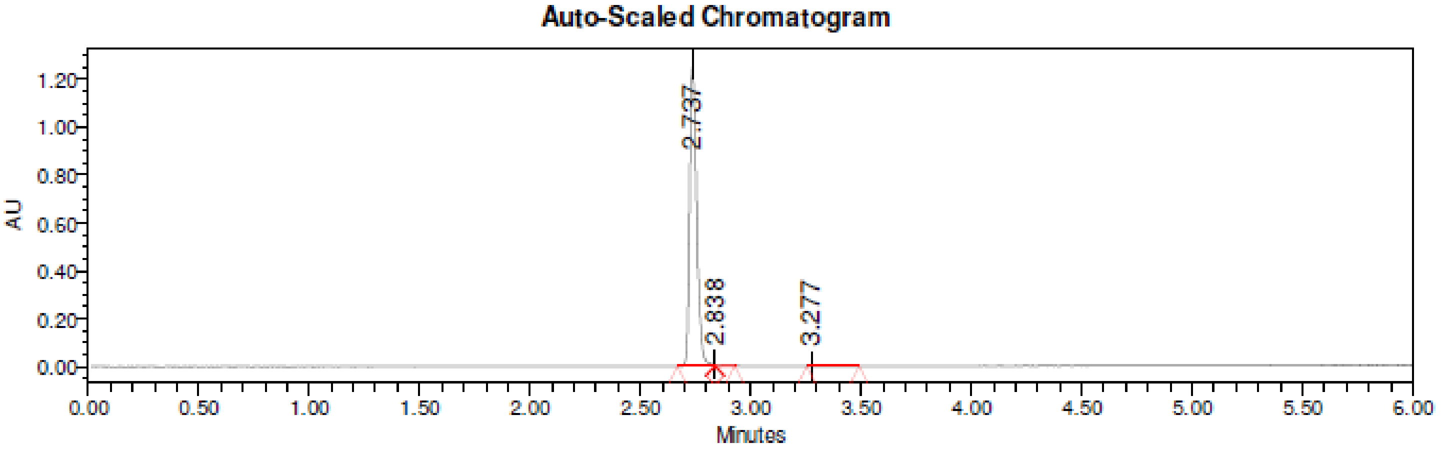

Peak Results

	RT	Area	Height	% Area
1	2.737	2765834	1264178	99.59
2	2.838	8998	5199	0.32
3	3.277	2423	539	0.09

GVK Biosciences Private Limited
Discovery Chemistry-Analytical Services
==================================

Sample ID:FAN Cluster-2 (C3804-127A2) **Date of analysis : 20-Mar-2019/19:19:18**
Acq Method :ATR-2 **Instrument ID: ANL-MCL2-LCMS-001**
1:B,4

021903C2596-FAN Clutser-2 (C3804-127A2)A — 5: Diode Array 270 Range: 1.617

Time	Height	Area	Area%
1.49	761	22.62	0.06
2.53	1616702	35791.50	99.30
2.57	13509	229.14	0.64

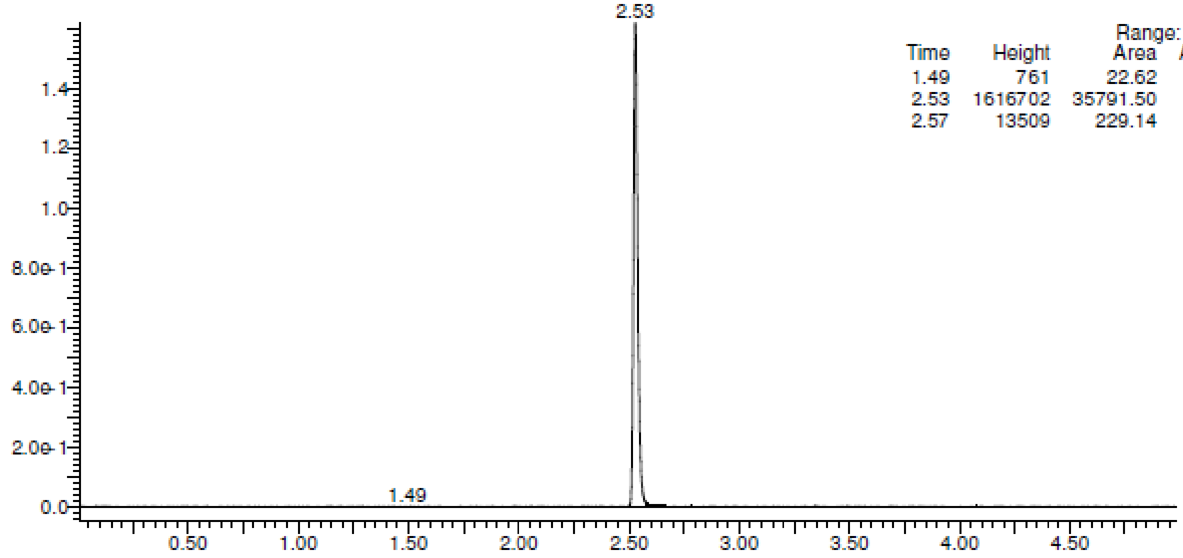

021903C2596-FAN Clutser-2 (C3804-127A2)A — 1: Scan ES+ 308.2 8.93e5

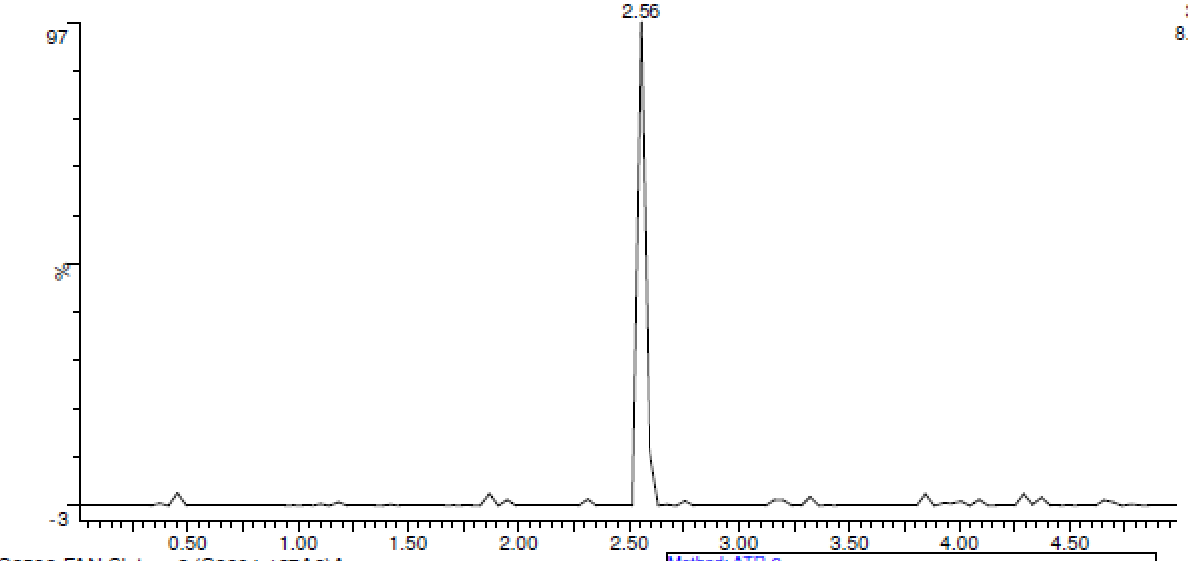

021903C2596-FAN Clutser-2 (C3804-127A2)A — Scan ES+ TIC 3.39e6

Method: ATR-2
Column : Acquity UPLC BEH C-18 (2.1X50 mm) 1.7u
MP :A: 0.05%FA in Water, B: 0.05%FA in Acetonitrile
T/%B : 0/30, 3.5/90,5/90, 5.1/30
Flow : 0.4 mL
Diluent : ACN+WATER(70:30)
Column temp :35°c

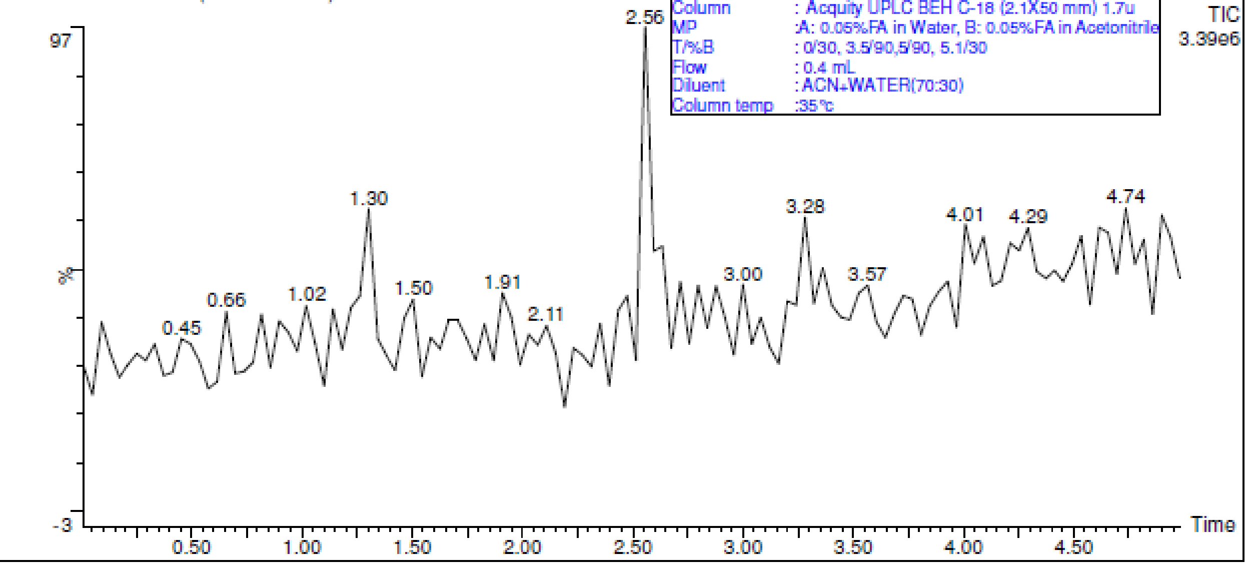

GVK Biosciences Private Limited
Discovery Chemistry-Analytical Services
=================================

Sample ID:FAN Cluster-2 (C3804-127A2) **Date of analysis: 20-Mar-2019:19:19:18**
Acq Method :ATR-2 **Instrument ID:ANL-MCL2-LCMS-001**
1:B,4

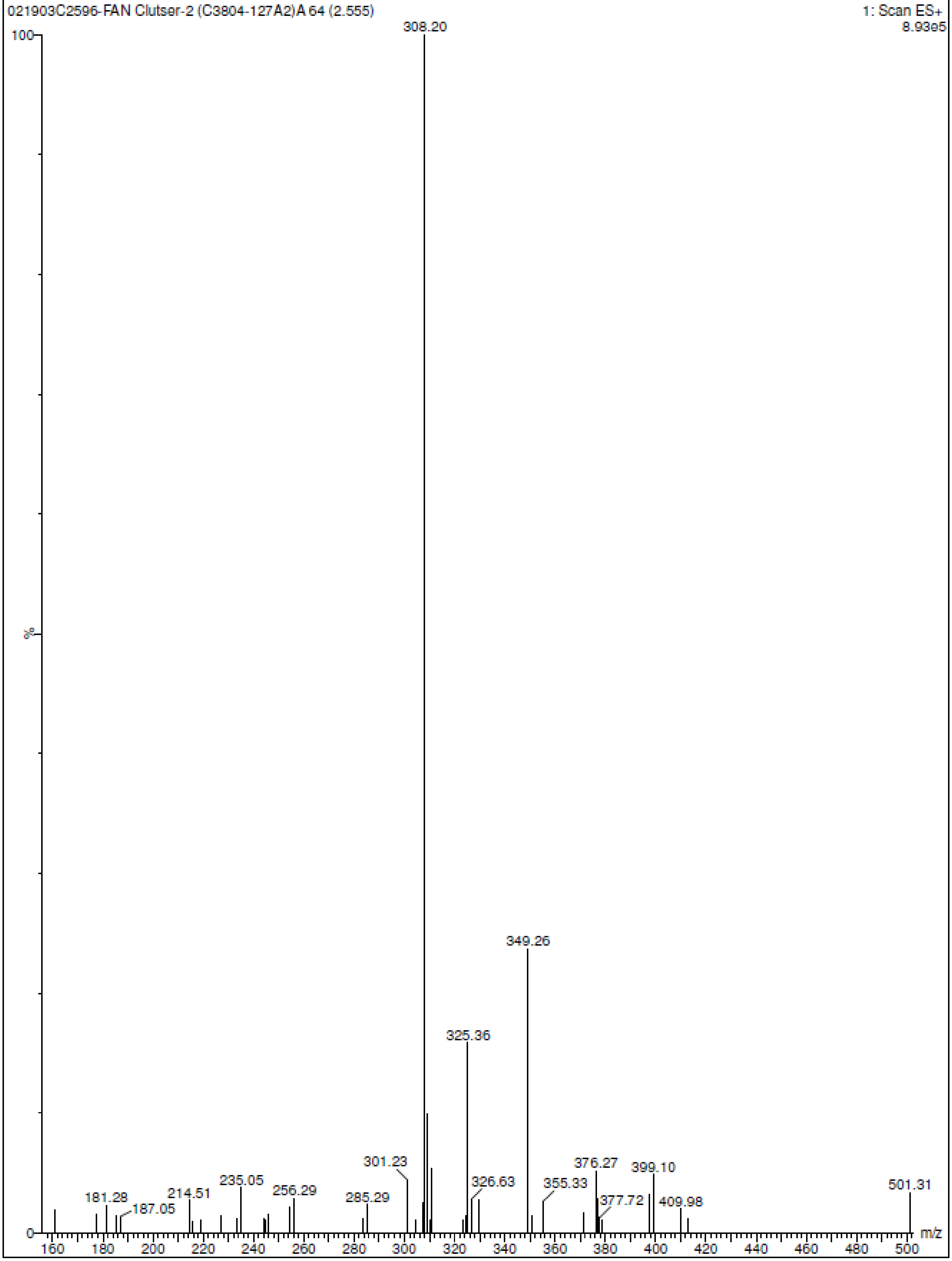

DFA_3 (3c)

S.No	Test	Results
1	**Description**	White Solid
2	**Identification**<br><br>(a) **NMR**<br><br>(b) **Mass by LCMS**	 <br>Complies to structure<br><br>308.20[M+H]+
3	**Chromatographic Purity by UPLC (Area %)**<br>Impurities>1.0%	99.86<br>Nil
4	**Chromatographic Purity by LCMS (Area %)**<br>Impurities>1.0%	99.53<br>Nil

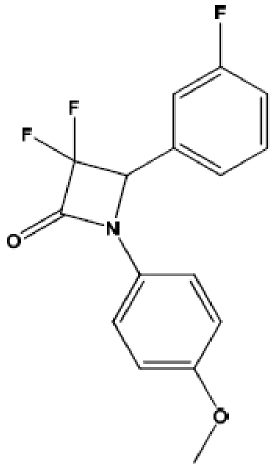

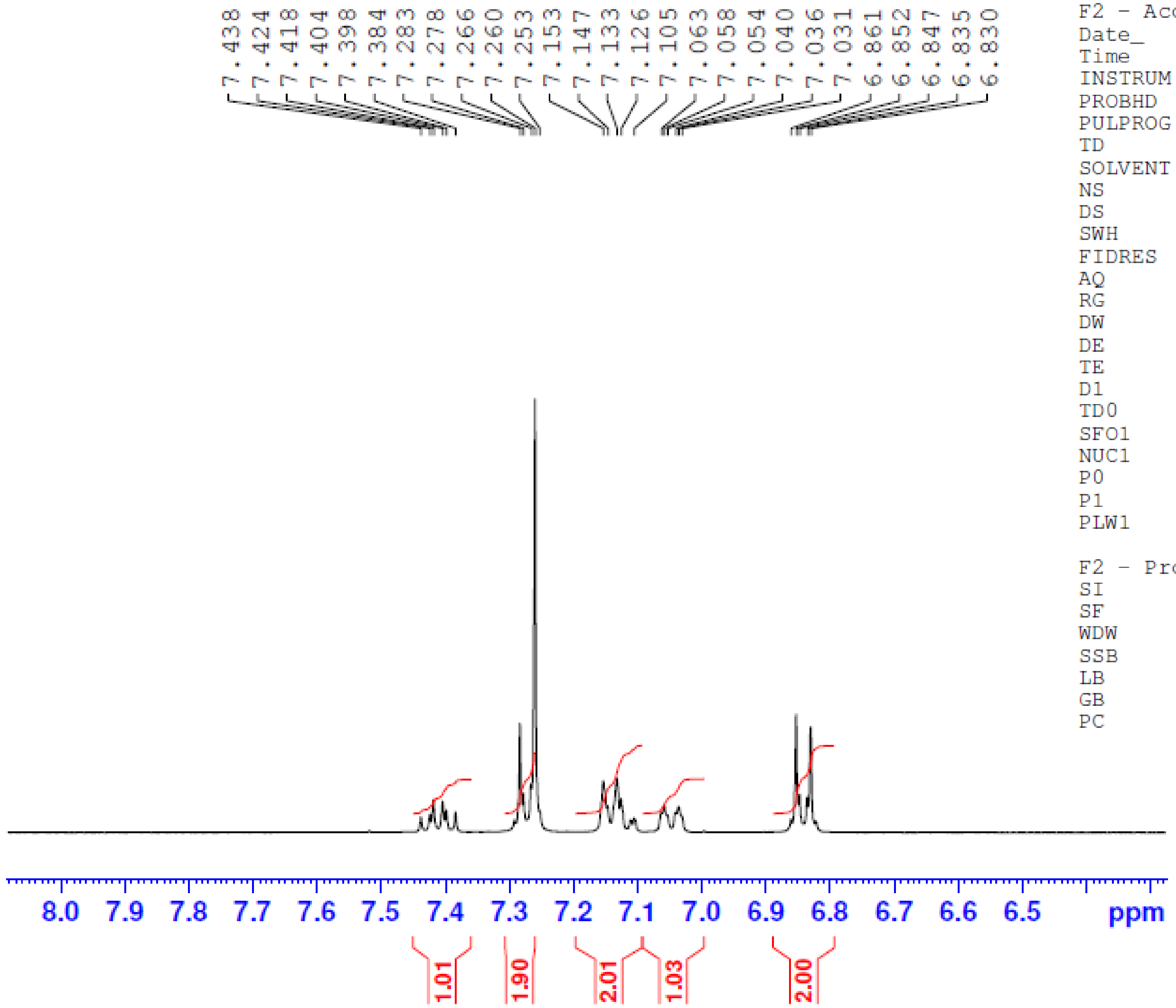

F2 - Acquisition Parameters

Date_	20190320
Time	16.23 h
INSTRUM	Avance Neo
PROBHD	Z116098_0787 (
PULPROG	zg30
TD	65536
SOLVENT	CDCl3
NS	16
DS	0
SWH	7142.857 Hz
FIDRES	0.217983 Hz
AQ	4.5875201 sec
RG	101
DW	70.000 usec
DE	14.62 usec
TE	298.1 K
D1	2.00000000 sec
TD0	1
SFO1	400.4024725 MHz
NUC1	1H
P0	3.33 usec
P1	10.00 usec
PLW1	19.73600006 W

F2 - Processing parameters

SI		65536
SF		400.4000101 MHz
WDW		EM
SSB	0	
LB		0.30 Hz
GB	0	
PC		1.00

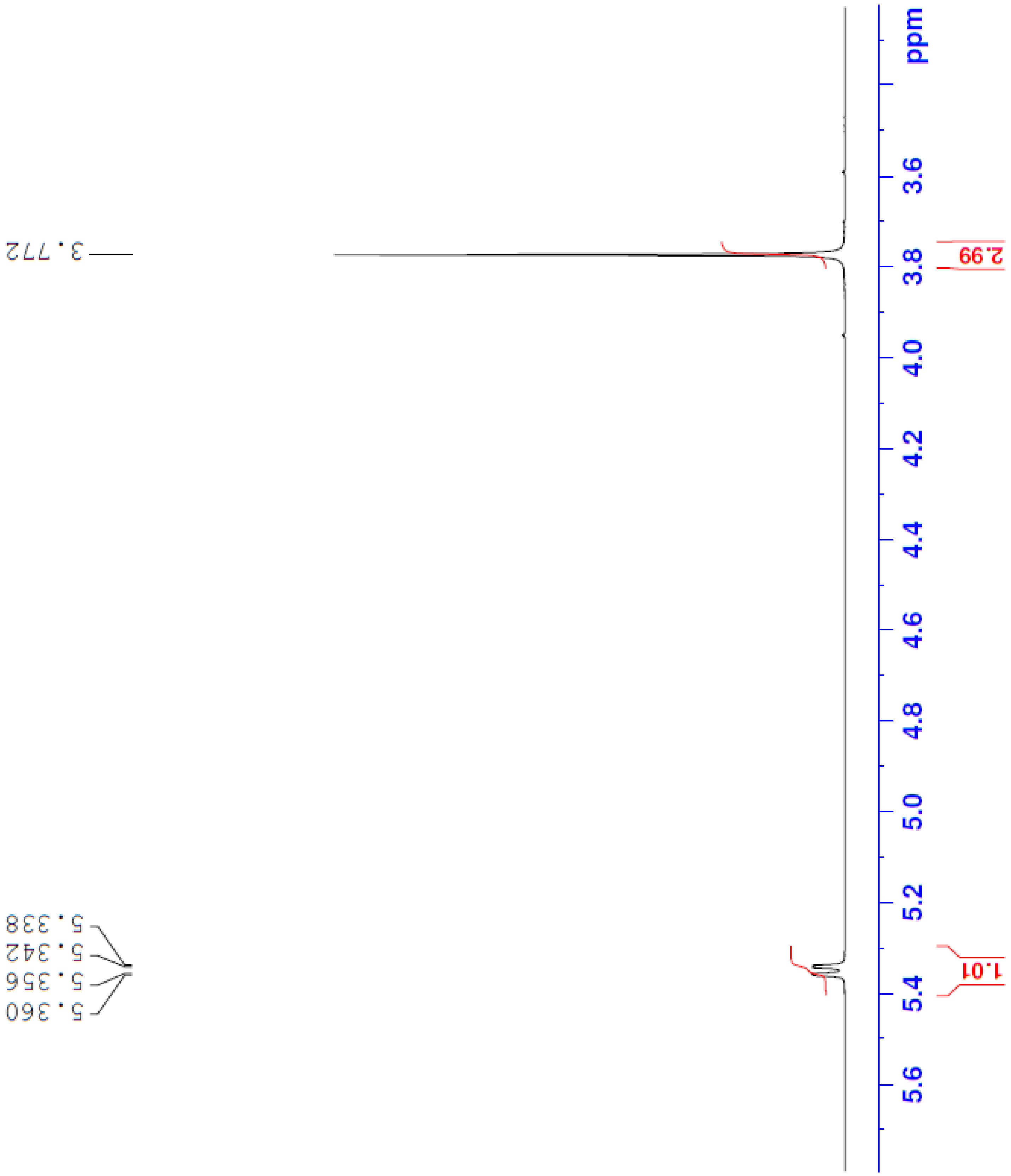
3.772
5.338
5.342
5.356
5.360
2.99
1.01
3.6
3.8
4.0
4.2
4.4
4.6
4.8
5.0
5.2
5.4
5.6
ppm

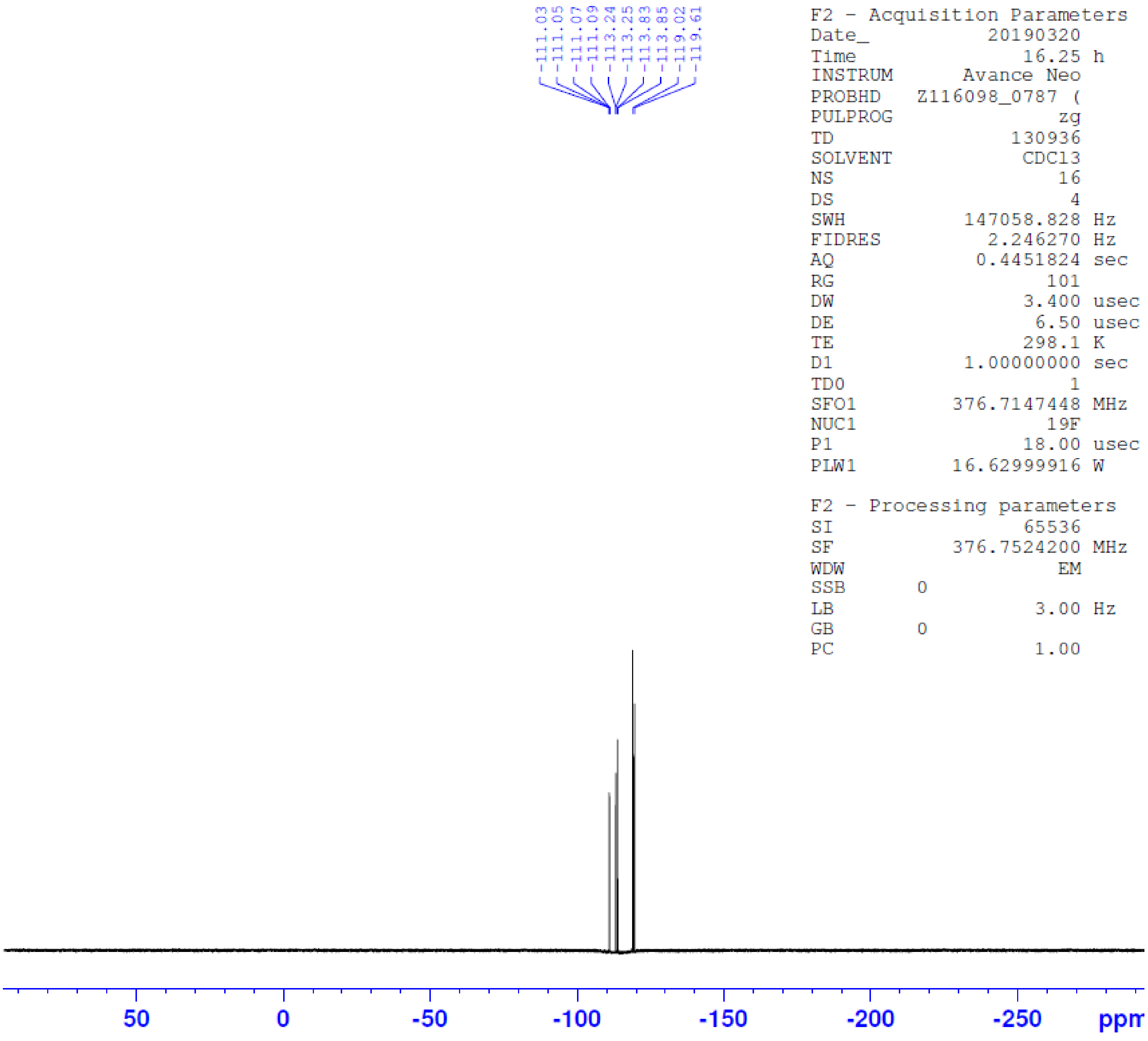

F2 - Acquisition Parameters
Date_ 20190320
Time 16.25 h
INSTRUM Avance Neo
PROBHD Z116098_0787 (
PULPROG zg
TD 130936
SOLVENT CDCl3
NS 16
DS 4
SWH 147058.828 Hz
FIDRES 2.246270 Hz
AQ 0.4451824 sec
RG 101
DW 3.400 usec
DE 6.50 usec
TE 298.1 K
D1 1.00000000 sec
TD0 1
SFO1 376.7147448 MHz
NUC1 19F
P1 18.00 usec
PLW1 16.62999916 W

F2 - Processing parameters
SI 65536
SF 376.7524200 MHz
WDW EM
SSB 0
LB 3.00 Hz
GB 0
PC 1.00

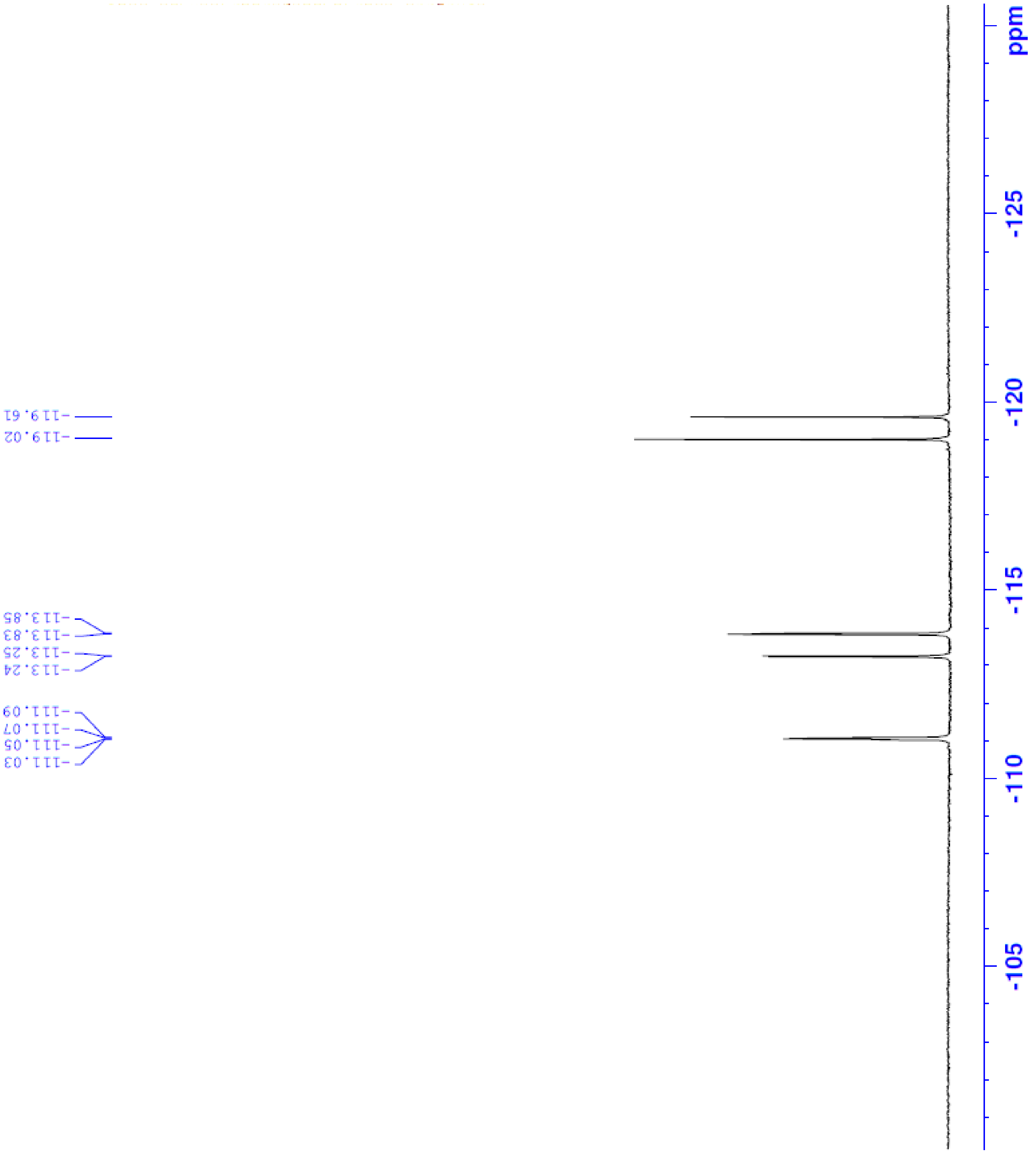
-119.61
-119.02
-113.85
-113.83
-113.25
-113.24
-111.09
-111.07
-111.05
-111.03
ppm
-125
-120
-115
-110
-105

UPLC Method Conditions :

Column	: Acquity UPLC BEH C18 (2.1x100) mm, 1.7µm
Mobile Phase-A	: 0.05% TFA in Water
Mobile Phase-B	: 0.05% TFA in Acetonitrile
Gradient (T/% B)	: 0/50,4/90,6/90,6.1/50
Flow Rate	: 0.3 mL/min
Temparature	: 40 °C
Diluent	: ACN+Water

Auto-Scaled Chromatogram

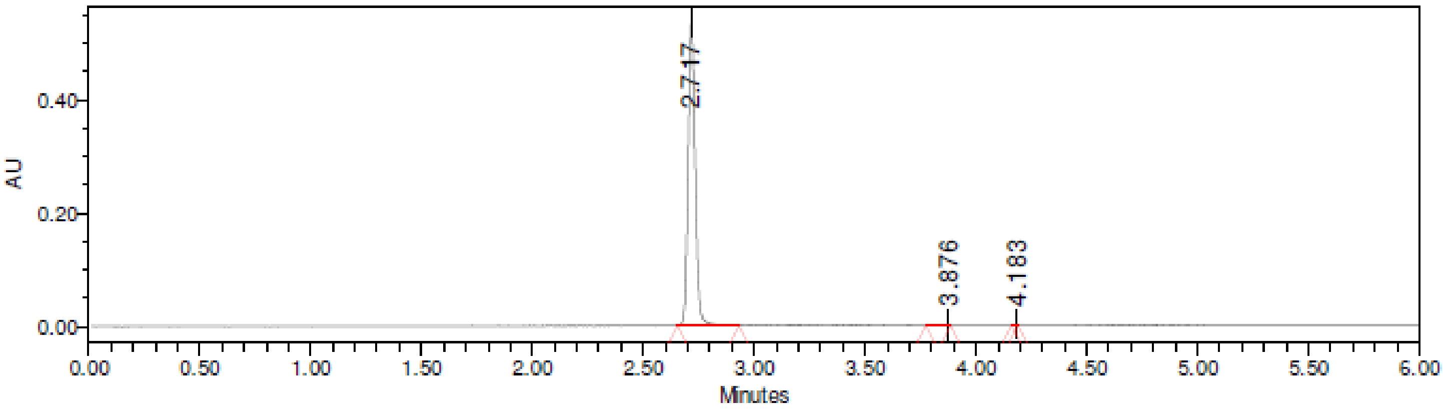

Peak Results

	RT	Area	Height	% Area
1	2.717	1198466	537256	99.86
2	3.876	1267	534	0.11
3	4.183	459	675	0.04

GVK Biosciences Private Limited
Discovery Chemistry-Analytical Services
================================

Sample ID:FAN cluster-3 (C4236-002-A3) **Date of analysis: 21-Mar-2019:00:01:17**
Acq Method :ATR-2 **Instrument ID:ANL-MCL2-LCMS-001**
2:F,5

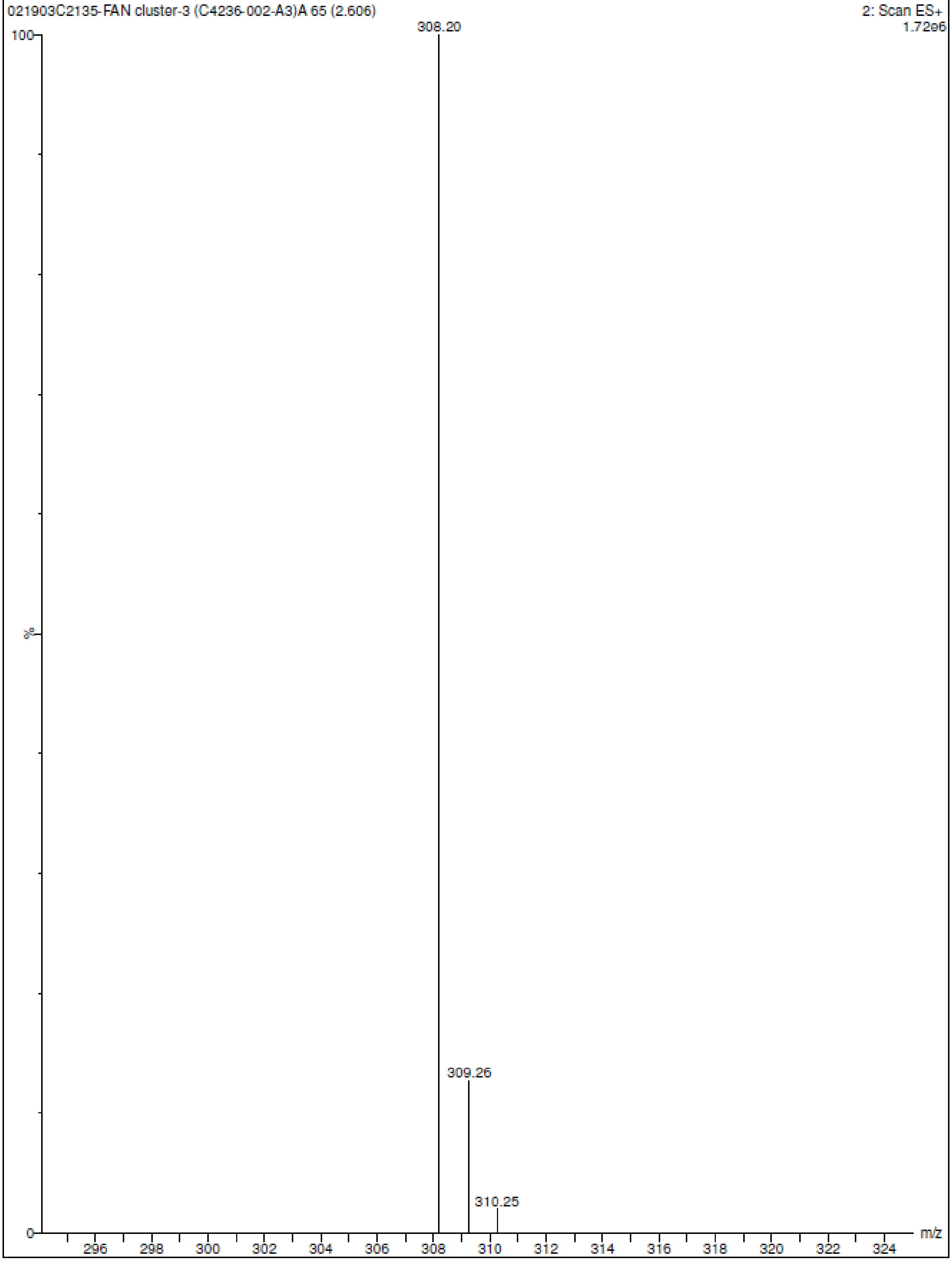

GVK Biosciences Private Limited
Discovery Chemistry-Analytical Services
==================================

Sample ID:FAN cluster-3 (C4236-002-A3) **Date of analysis : 21-Mar-2019/00:01:17**
Acq Method :ATR-2 **Instrument ID: ANL-MCL2-LCMS-001**
2:F,5

021903C2135-FAN cluster-3 (C4236-002-A3)A

5: Diode Array
270
Range: 1.035

Time	Height	Area	Area%
2.30	918	12.72	0.06
2.49	868	9.05	0.04
2.55	564	2.97	0.01
2.59	1034695	20935.41	99.53
2.63	3984	75.10	0.36

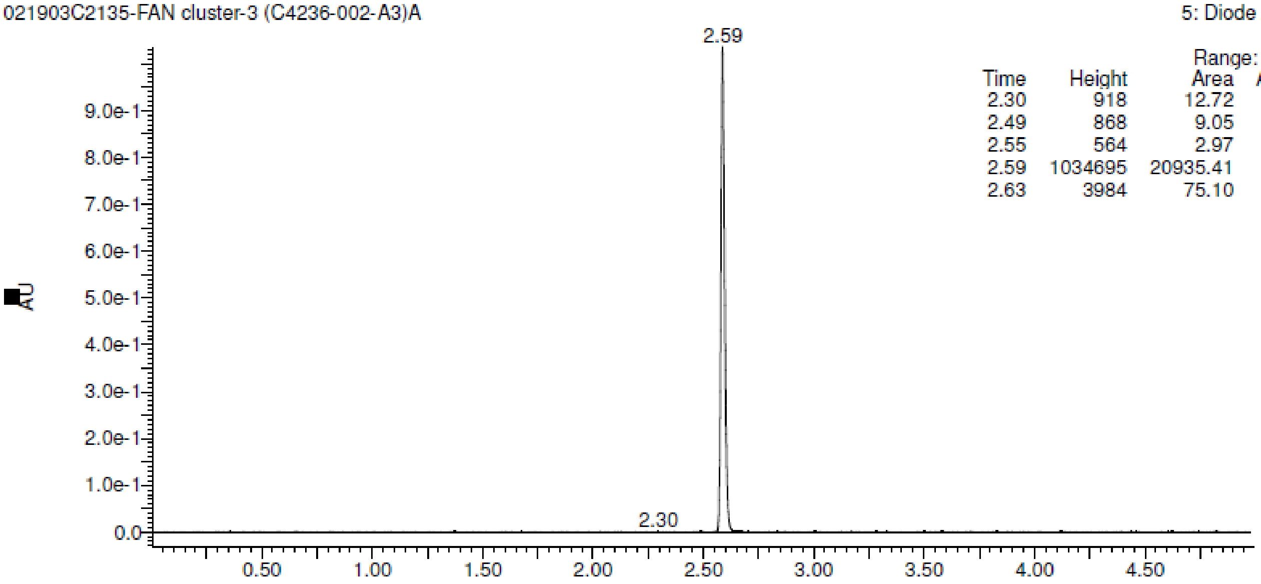

021903C2135-FAN cluster-3 (C4236-002-A3)A

2: Scan ES+
308.2
1.72e6

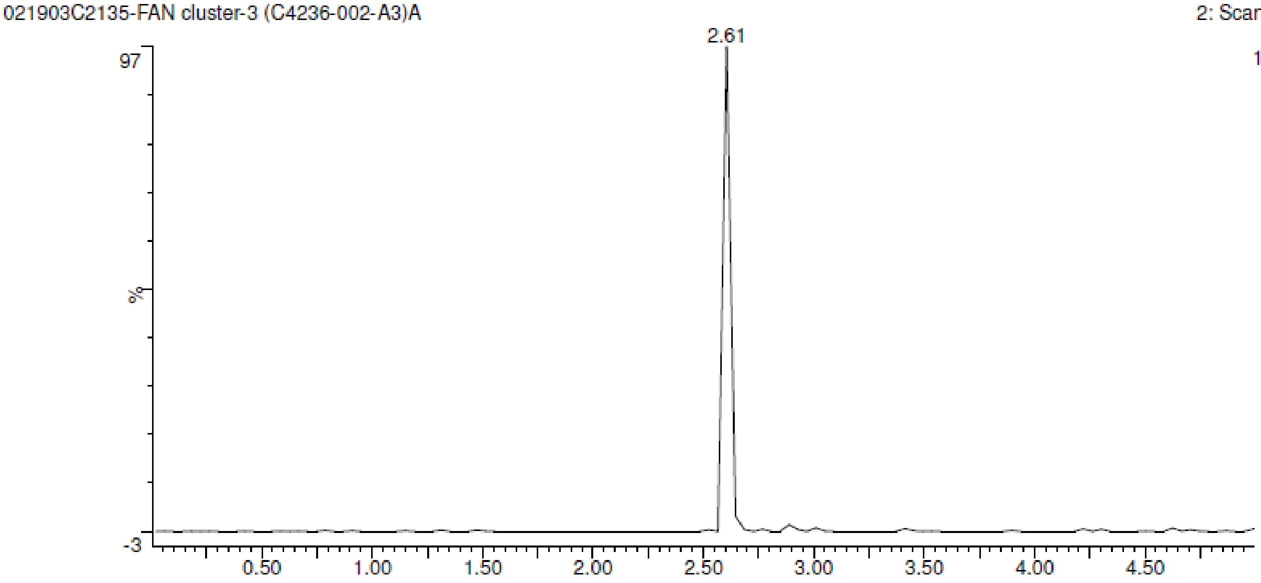

021903C2135-FAN cluster-3 (C4236-002-A3)A

2: Scan ES+
TIC
5.44e6

Method: ATR-2
Column : Acquity UPLC BEH C-18 (2.1X50 mm) 1.7u
MP :A: 0.05%FA in Water, B: 0.05%FA in Acetonitrile
T/%B : 0/30, 3.5/90,5/90, 5.1/30
Flow : 0.4 mL
Diluent : ACN+WATER(70:30)
Column temp :35°c

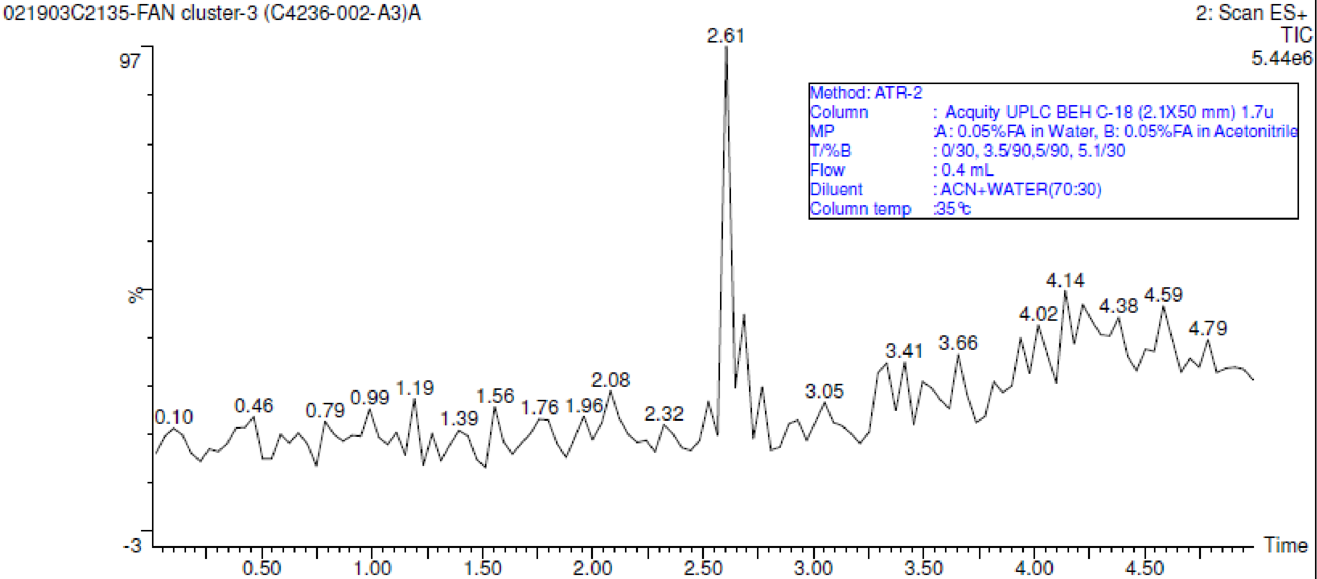

(3d)

S.No	Test	Results
1	**Description**	White Solid
2	**Identification**<br><br>(a) **NMR**<br><br>(b) **Mass by LCMS**	 <br>Complies to structure<br><br>260.24[M+H]$^+$
3	**Chromatographic Purity by UPLC (Area %)**<br>Impurities>1.0%	99.66<br>Nil
4	**Chromatographic Purity by LCMS (Area %)**<br>Impurities>1.0%	99.75<br>Nil

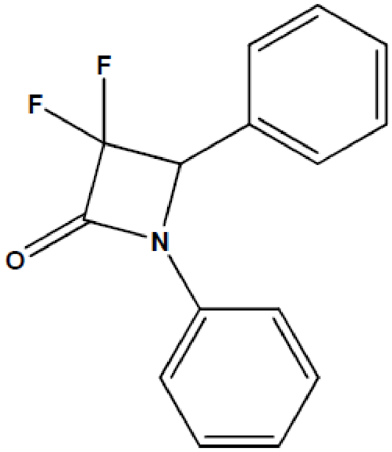

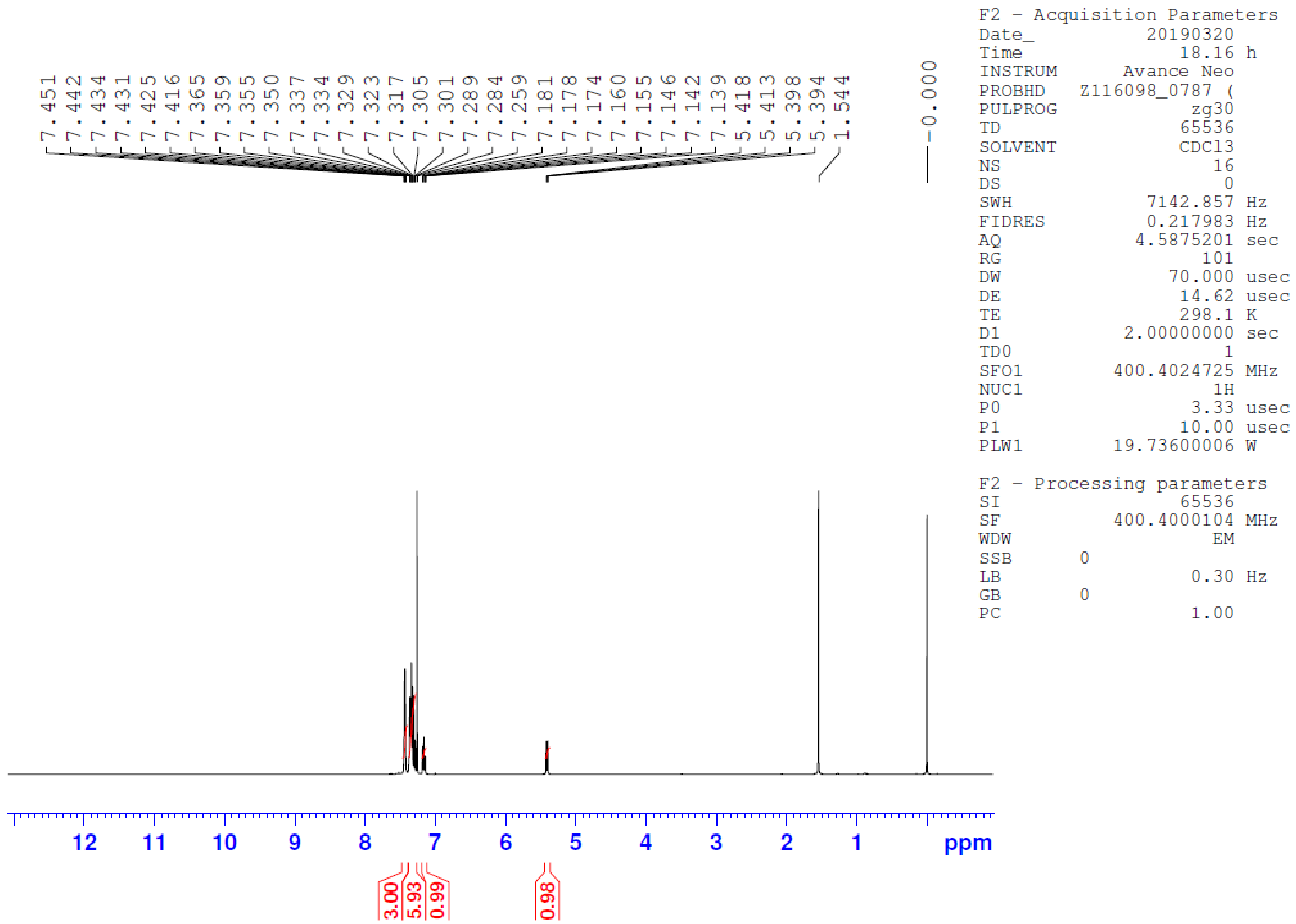
7.451
7.442
7.434
7.431
7.425
7.416
7.365
7.359
7.355
7.350
7.337
7.334
7.329
7.323
7.317
7.305
7.301
7.289
7.284
7.259
7.181
7.178
7.174
7.160
7.155
7.146
7.142
7.139
5.418
5.413
5.398
5.394
1.544
0.000
12
11
10
9
8
7
6
5
4
3
2
1
ppm
3.00
5.93
0.99
0.98
F2 - Acquisition Parameters
Date_ 20190320
Time 18.16 h
INSTRUM Avance Neo
PROBHD Z116098_0787 (
PULPROG zg30
TD 65536
SOLVENT CDCl3
NS 16
DS 0
SWH 7142.857 Hz
FIDRES 0.217983 Hz
AQ 4.5875201 sec
RG 101
DW 70.000 usec
DE 14.62 usec
TE 298.1 K
D1 2.00000000 sec
TD0 1
SFO1 400.4024725 MHz
NUC1 1H
P0 3.33 usec
P1 10.00 usec
PLW1 19.73600006 W
F2 - Processing parameters
SI 65536
SF 400.4000104 MHz
WDW EM
SSB 0
LB 0.30 Hz
GB 0
PC 1.00

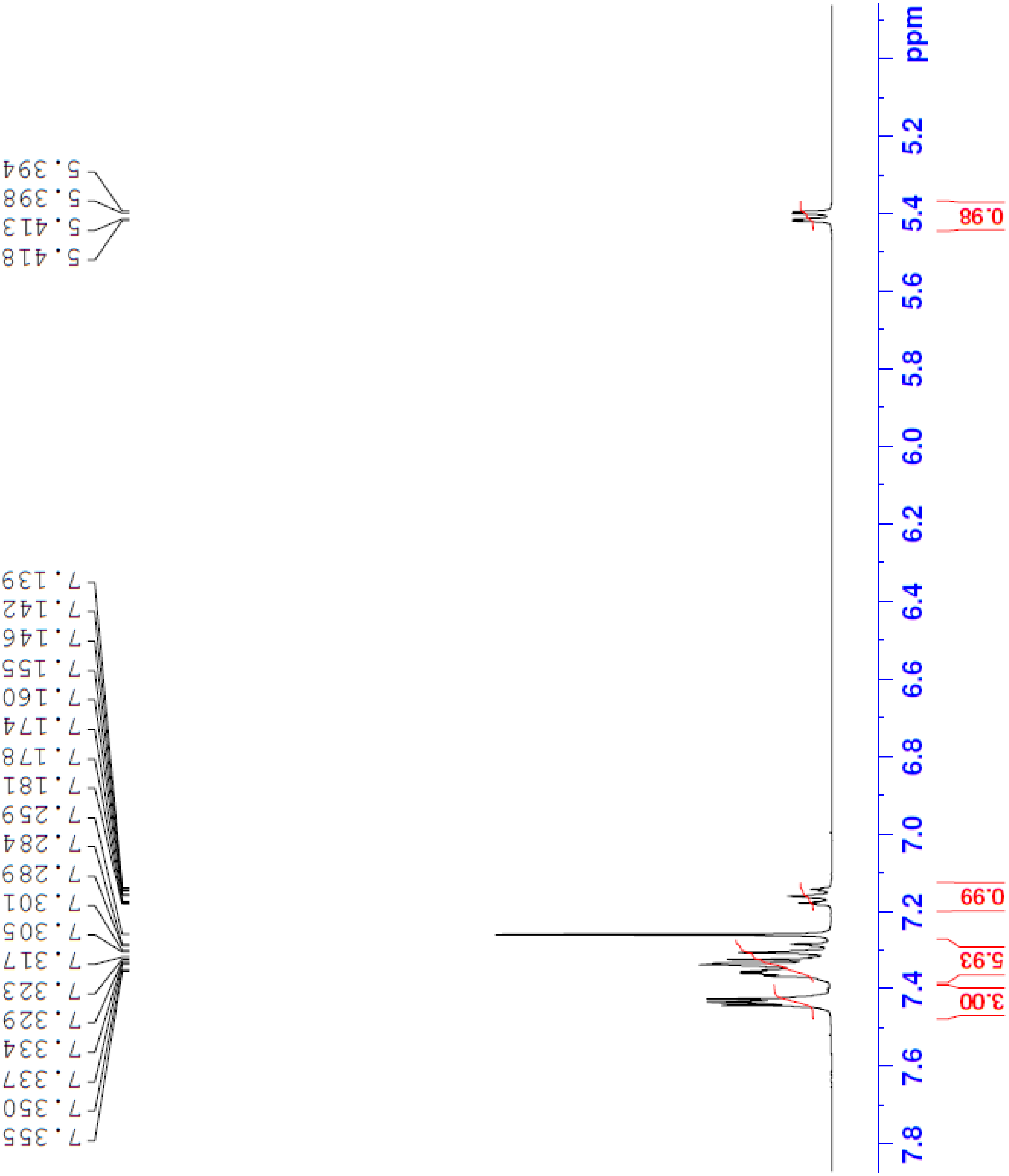
5.394
5.398
5.413
5.418
7.139
7.142
7.146
7.155
7.160
7.174
7.178
7.181
7.259
7.284
7.289
7.301
7.305
7.317
7.323
7.329
7.334
7.337
7.350
7.355
0.98
0.99
5.93
3.00
ppm
5.2
5.4
5.6
5.8
6.0
6.2
6.4
6.6
6.8
7.0
7.2
7.4
7.6
7.8

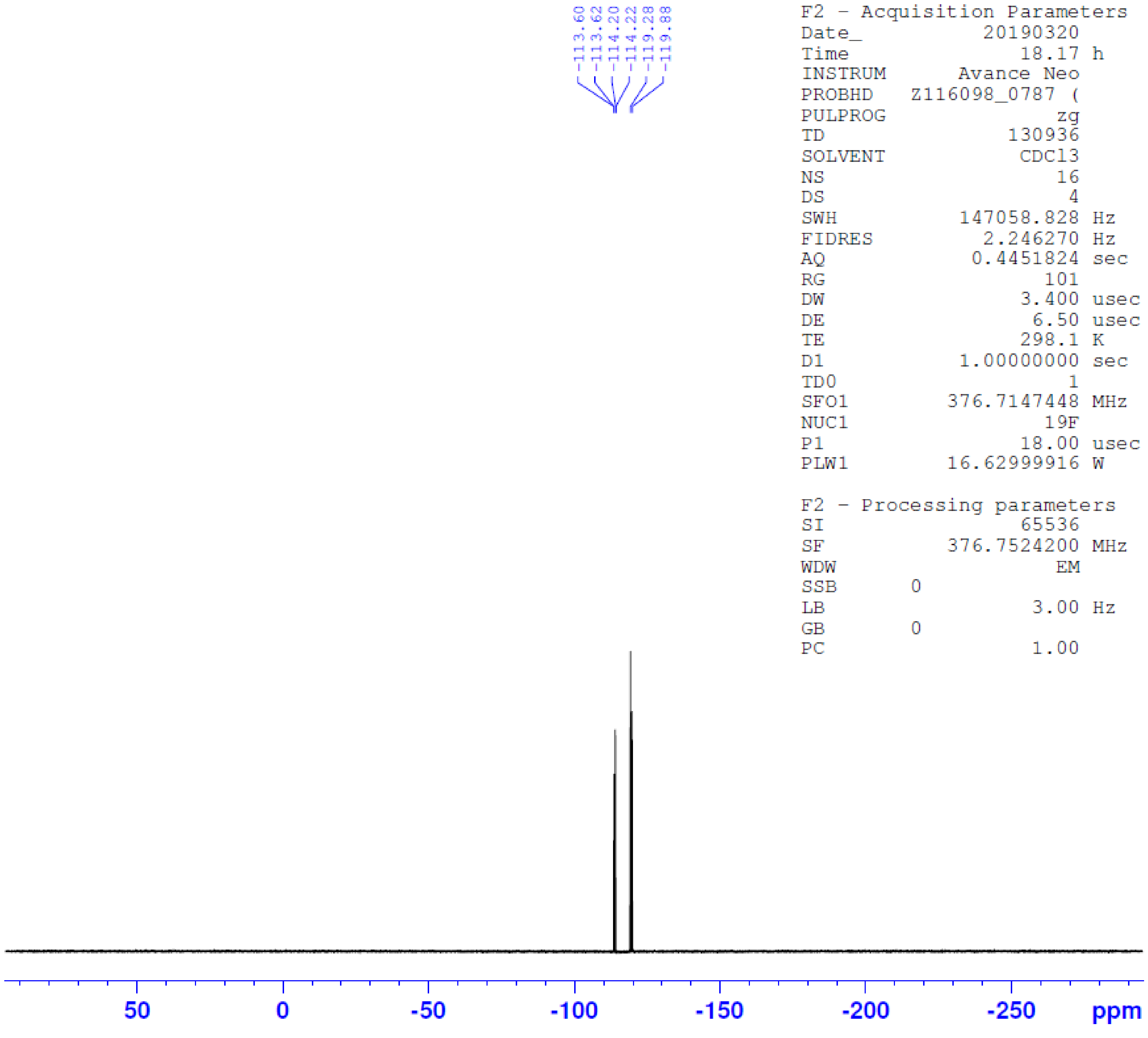
-113.60
-113.62
-114.20
-114.22
-119.28
-119.88
F2 - Acquisition Parameters
Date_ 20190320
Time 18.17 h
INSTRUM Avance Neo
PROBHD Z116098_0787 (
PULPROG zg
TD 130936
SOLVENT CDCl3
NS 16
DS 4
SWH 147058.828 Hz
FIDRES 2.246270 Hz
AQ 0.4451824 sec
RG 101
DW 3.400 usec
DE 6.50 usec
TE 298.1 K
D1 1.00000000 sec
TD0 1
SFO1 376.7147448 MHz
NUC1 19F
P1 18.00 usec
PLW1 16.62999916 W
F2 - Processing parameters
SI 65536
SF 376.7524200 MHz
WDW EM
SSB 0
LB 3.00 Hz
GB 0
PC 1.00
50
0
-50
-100
-150
-200
-250
ppm

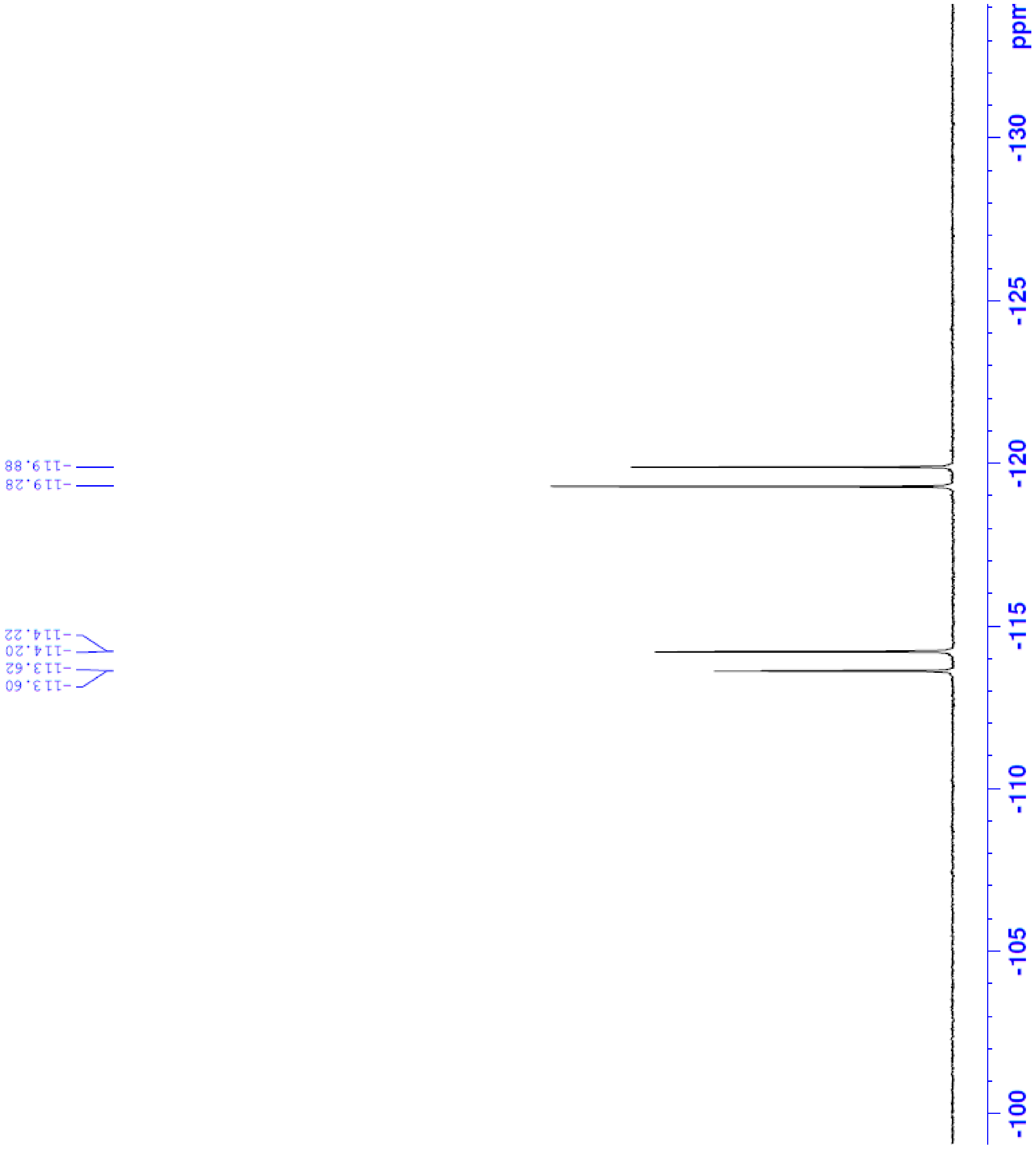

-119.88
-119.28
-114.22
-114.20
-113.62
-113.60
ppm
-130
-125
-120
-115
-110
-105
-100

UPLC Method Conditions :

Column	: Acquity UPLC BEH C18 (2.1x100) mm, 1.7µm
Mobile Phase-A	: 0.05% TFA in Water
Mobile Phase-B	: 0.05% TFA in Acetonitrile
Gradient (T/% B)	: 0/50,4/90,6/90,6.1/50
Flow Rate	: 0.3 mL/min
Temparature	: 40℃
Diluent	: ACN+Water

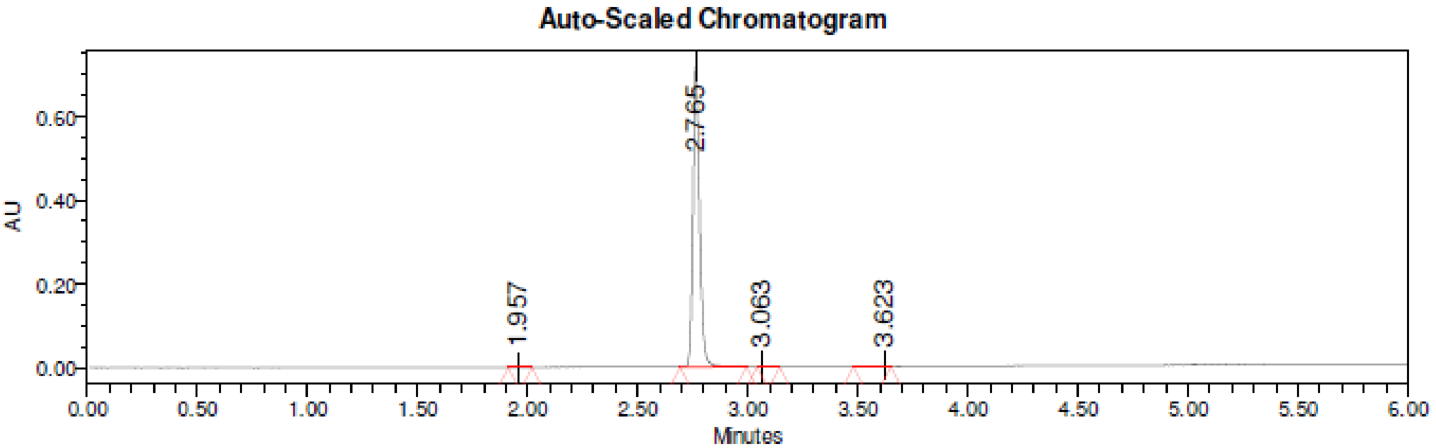

Peak Results

	RT	Area	Height	% Area
1	1.957	1367	478	0.09
2	2.765	1501509	720389	99.66
3	3.063	1320	525	0.09
4	3.623	2499	450	0.17

Sample ID:FAN Cluster-6 (C3804-124A2)
Acq.Method:
1:B,6

GVK Biosciences Private Limited
Discovery Chemistry-Analytical Services
=================================

19-Mar-2019/10:51:44/
Instrument ID: ANL-MCL1-LCMS-002

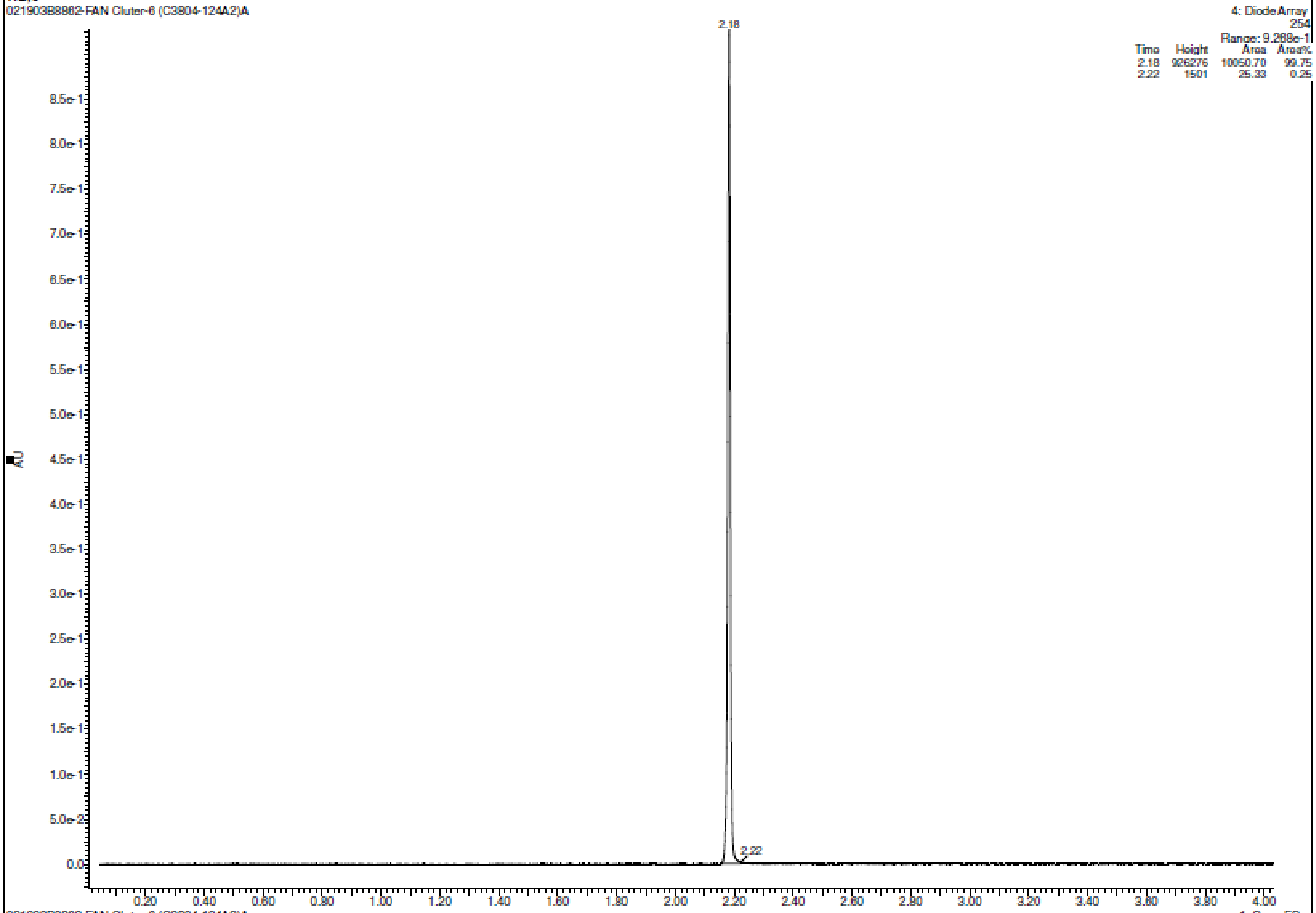

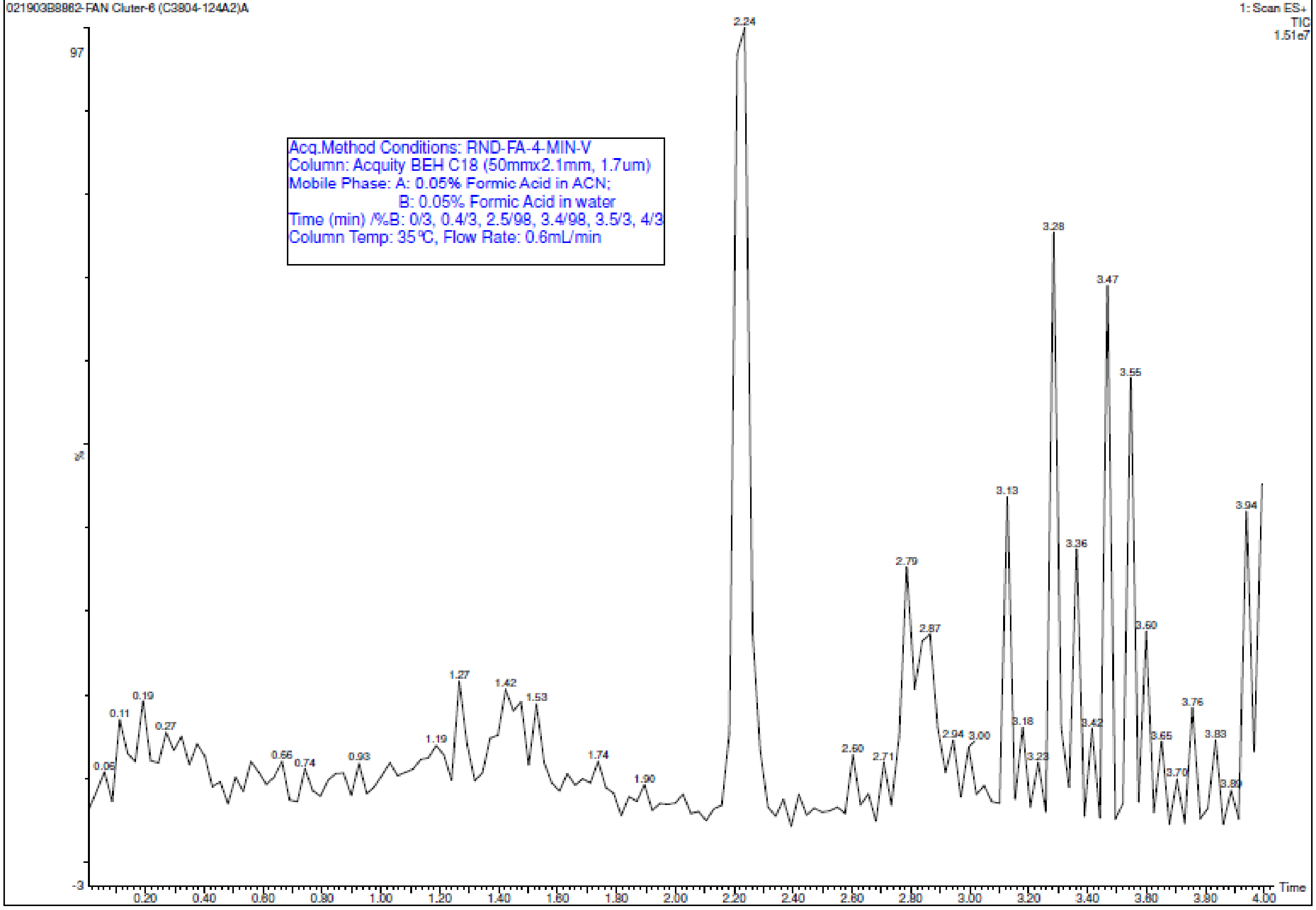

GVK Biosciences Private Limited
Discovery Chemistry-Analytical Services
================================

Sample ID:FAN Cluster-6 (C3804-124A2)
Acq.Method:
1:B,6

Date Of Analysis:19-Mar-2019;10:51:44
Instrument ID:ANL-MCL1-LCMS-002

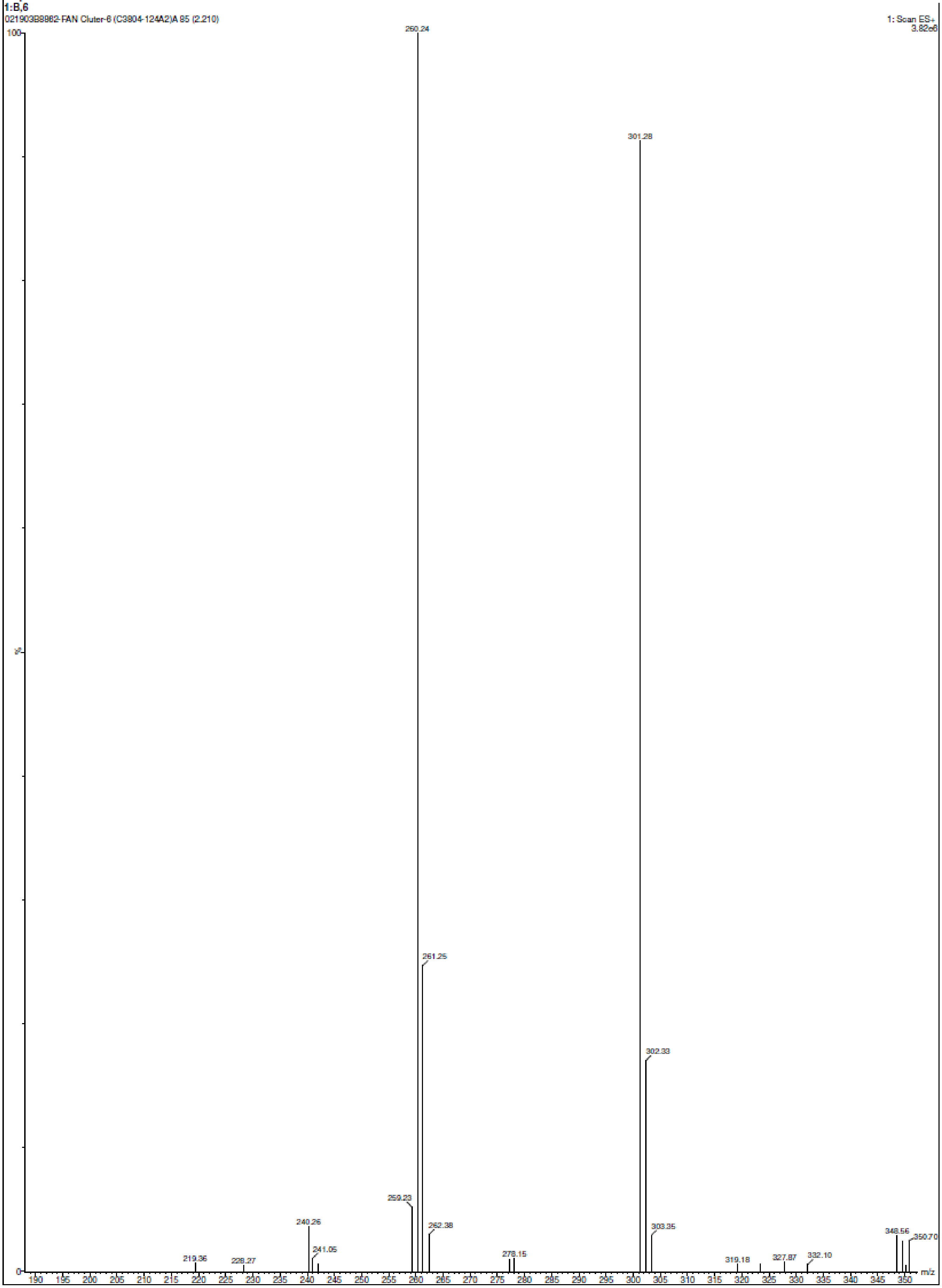

DFA_5 (3e)

S.No	Test	Results
1	**Description**	White Solid
2	**Identification**<br><br>(a) **NMR**<br><br>(b) **Mass by LCMS**	 <br>Complies to structure<br><br>308.20[M+H]$^+$
3	**Chromatographic Purity by UPLC (Area %)**<br>Impurities>1.0%	99.26<br>Nil
4	**Chromatographic Purity by LCMS (Area %)**<br>Impurities>1.0%	99.17<br>Nil
**Remarks :** Traces of Aliphatic impurities observed in NMR spectrum .		

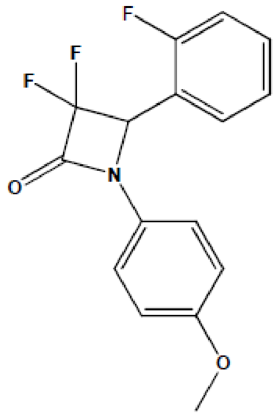

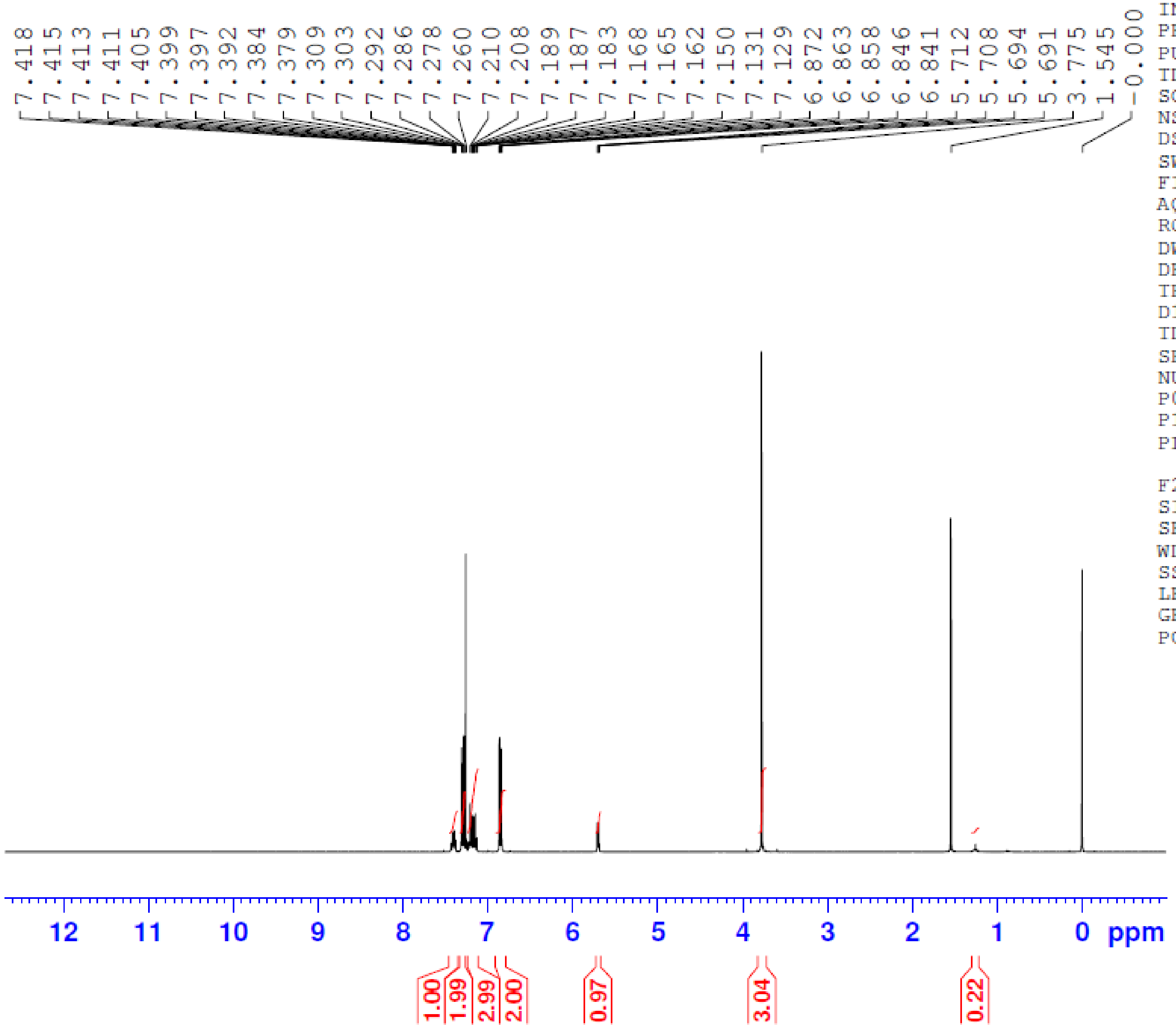

F2 - Acquisition Parameters		
Date_	20190320	
Time	16.29	h
INSTRUM	Avance Neo	
PROBHD	Z116098_0787 (	
PULPROG	zg30	
TD	65536	
SOLVENT	CDCl3	
NS	16	
DS	0	
SWH	7142.857	Hz
FIDRES	0.217983	Hz
AQ	4.5875201	sec
RG	101	
DW	70.000	usec
DE	14.62	usec
TE	298.1	K
D1	2.00000000	sec
TD0	1	
SFO1	400.4024725	MHz
NUC1	1H	
P0	3.33	usec
P1	10.00	usec
PLW1	19.73600006	W

F2 - Processing parameters		
SI	65536	
SF	400.4000101	MHz
WDW	EM	
SSB	0	
LB	0.30	Hz
GB	0	
PC	1.00	

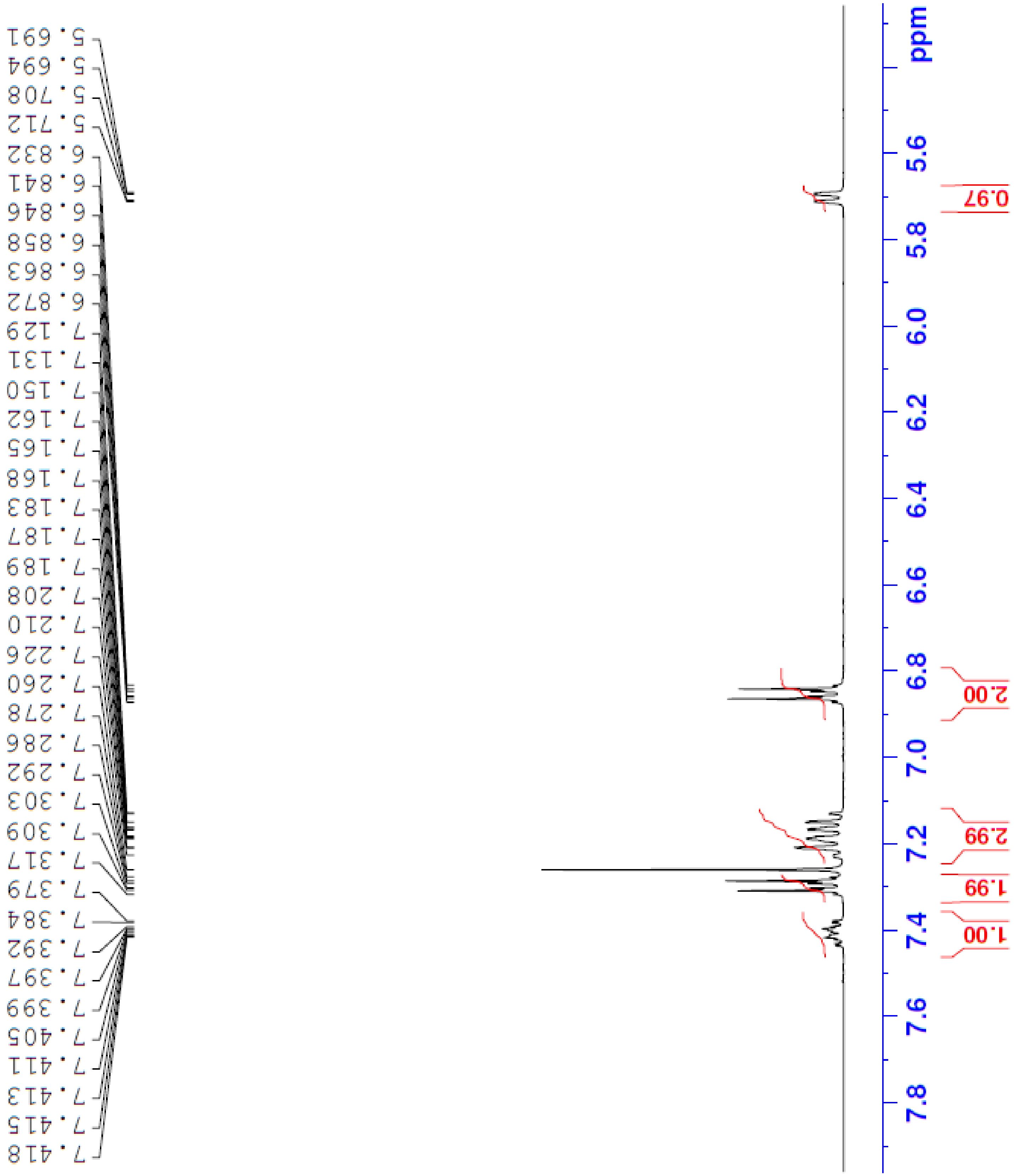

5.691
5.694
5.708
5.712
6.832
6.841
6.846
6.858
6.863
6.872
7.129
7.131
7.150
7.162
7.165
7.168
7.183
7.187
7.189
7.208
7.210
7.226
7.260
7.278
7.286
7.292
7.303
7.309
7.317
7.379
7.384
7.392
7.397
7.399
7.405
7.411
7.413
7.415
7.418
0.97
2.00
2.99
1.99
1.00
ppm
5.6
5.8
6.0
6.2
6.4
6.6
6.8
7.0
7.2
7.4
7.6
7.8

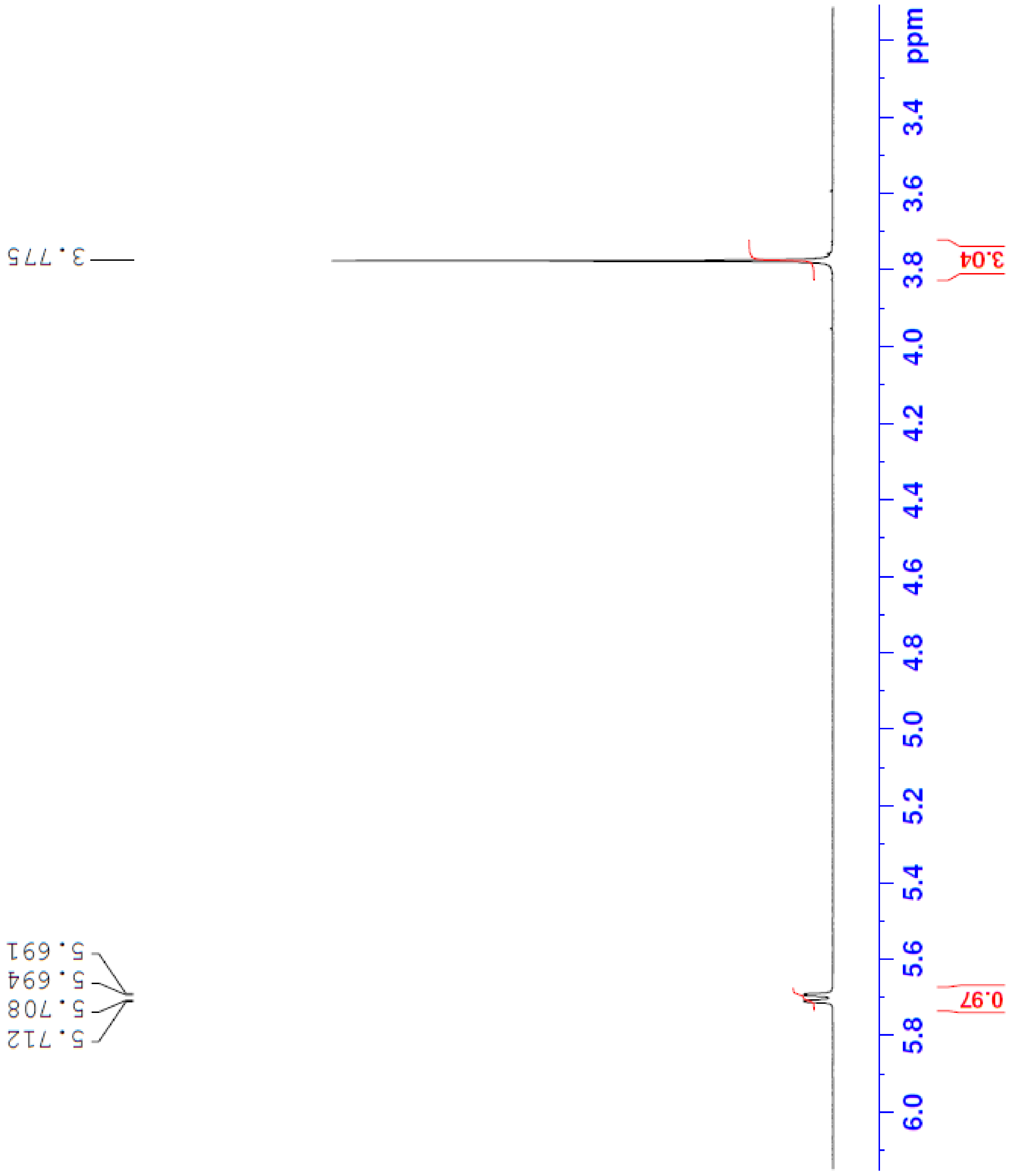
3.775
5.691
5.694
5.708
5.712
ppm
3.4
3.6
3.8
4.0
4.2
4.4
4.6
4.8
5.0
5.2
5.4
5.6
5.8
6.0
3.04
0.97

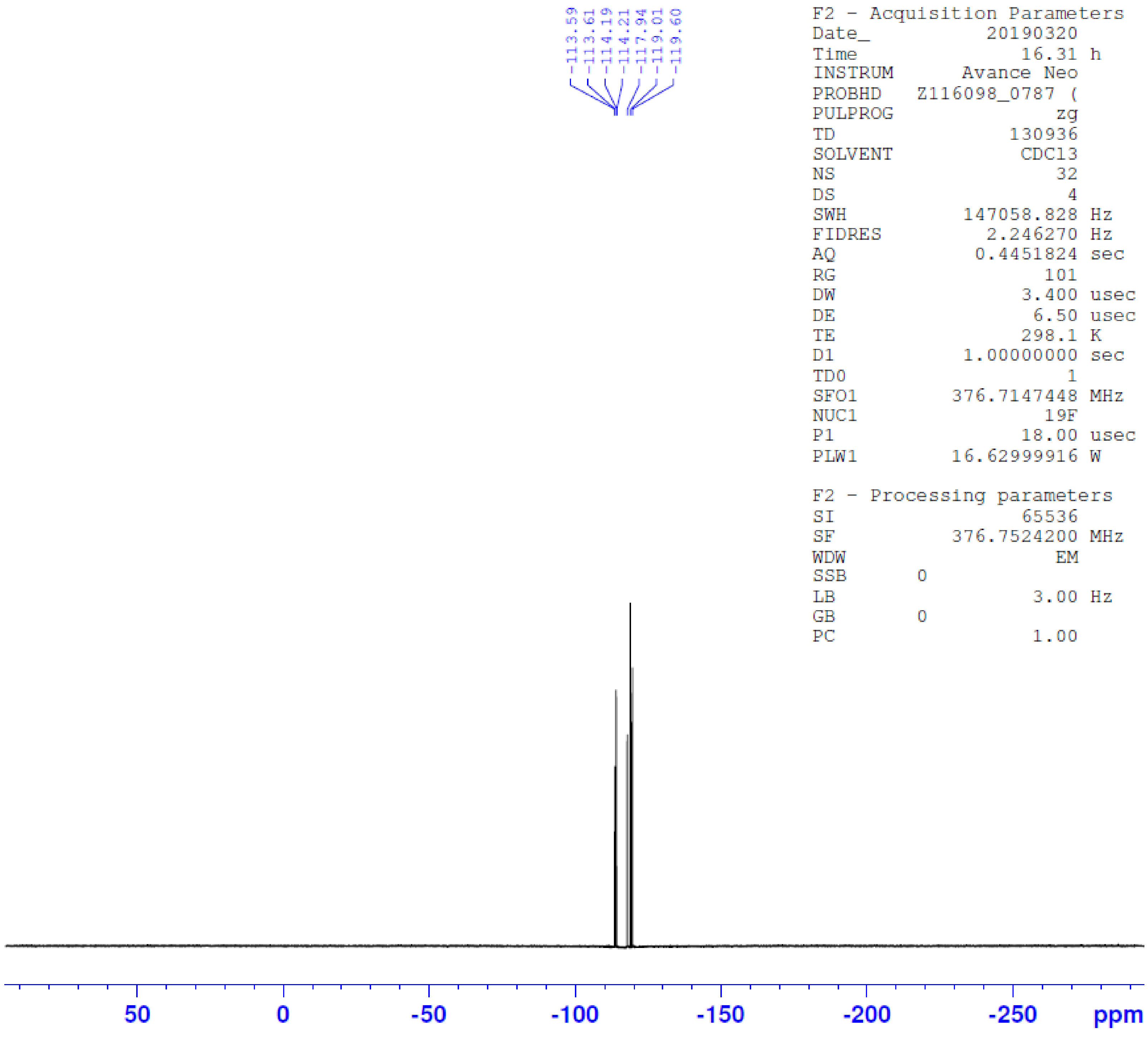


```
F2 - Acquisition Parameters
Date_              20190320
Time                  16.31 h
INSTRUM          Avance Neo
PROBHD    Z116098_0787 (
PULPROG                  zg
TD                   130936
SOLVENT               CDCl3
NS                       32
DS                        4
SWH              147058.828 Hz
FIDRES             2.246270 Hz
AQ                0.4451824 sec
RG                      101
DW                    3.400 usec
DE                     6.50 usec
TE                    298.1 K
D1               1.00000000 sec
TD0                       1
SFO1            376.7147448 MHz
NUC1                    19F
P1                    18.00 usec
PLW1            16.62999916 W

F2 - Processing parameters
SI                    65536
SF              376.7524200 MHz
WDW                      EM
SSB      0
LB                     3.00 Hz
GB       0
PC                     1.00
```

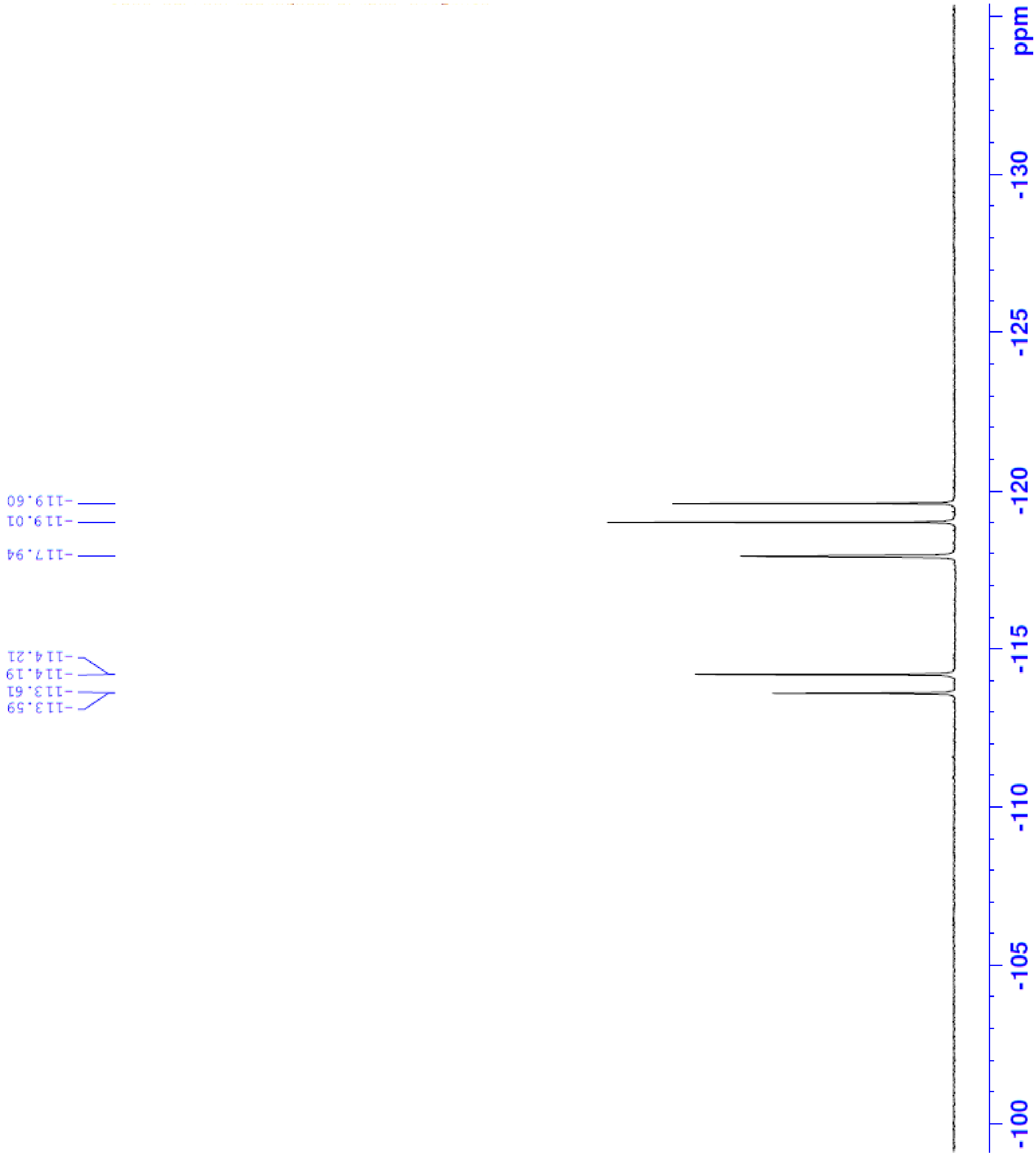

-119.60
-119.01
-117.94
-114.21
-114.19
-113.61
-113.59
ppm
-130
-125
-120
-115
-110
-105
-100

UPLC Method Conditions :

Column : Acquity UPLC BEH C18 (2.1x100) mm, 1.7µm
Mobile Phase-A : 0.05% TFA in Water
Mobile Phase-B : 0.05% TFA in Acetonitrile
Gradient (T/% B) : 0/50,4/90,6/90,6.1/50
Flow Rate : 0.3 mL/min
Temparature : 40 ℃
Diluent : ACN+Water

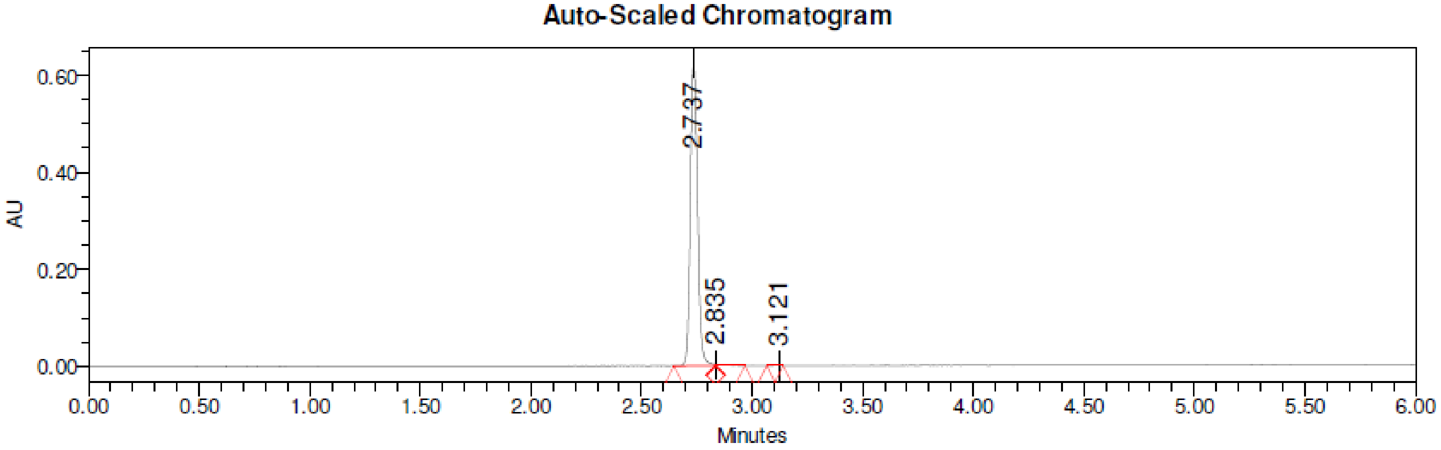

Peak Results

	RT	Area	Height	% Area
1	2.737	1393286	625365	99.26
2	2.835	8740	2445	0.62
3	3.121	1701	814	0.12

GVK Biosciences Private Limited
Discovery Chemistry-Analytical Services
==================================

Sample ID:FAN Cluster-5 (C4236-004-A1) | **Date of analysis : 21-Mar-2019/00:15:08**
Acq Method :ATR-2 | **Instrument ID: ANL-MCL2-LCMS-001**
2:F,7

021903C2169-FAN Cluster-5 (C4236-004-A1)A | 5: Diode Array 270 Range: 9.555e-1

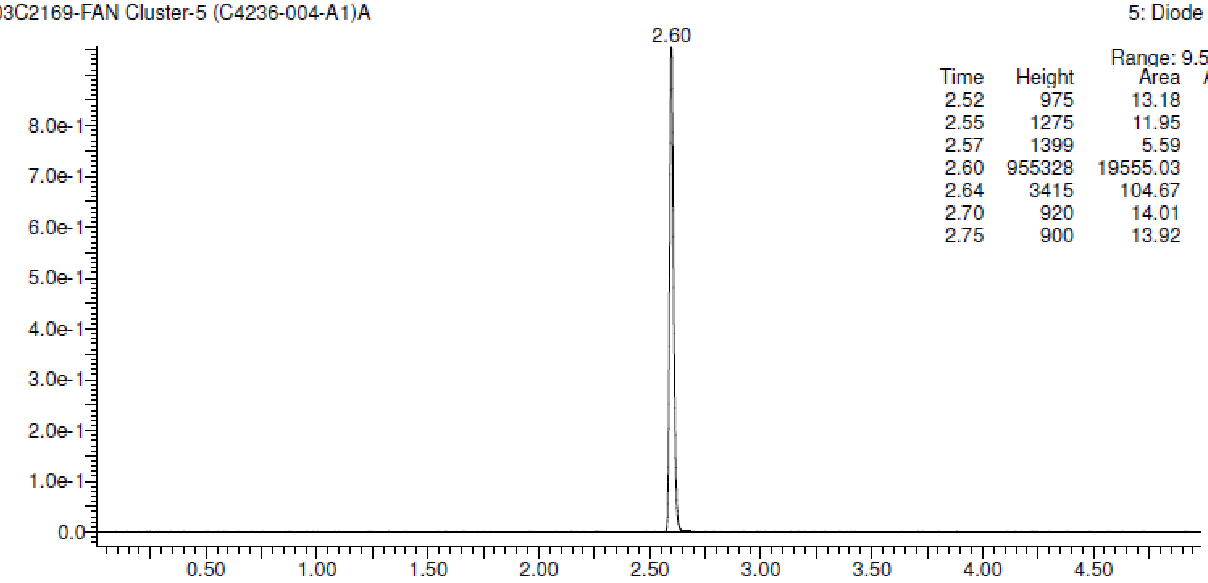

Time	Height	Area	Area%
2.52	975	13.18	0.07
2.55	1275	11.95	0.06
2.57	1399	5.59	0.03
2.60	955328	19555.03	99.17
2.64	3415	104.67	0.53
2.70	920	14.01	0.07
2.75	900	13.92	0.07

021903C2169-FAN Cluster-5 (C4236-004-A1)A | 1: Scan ES+ 308.2 8.66e5

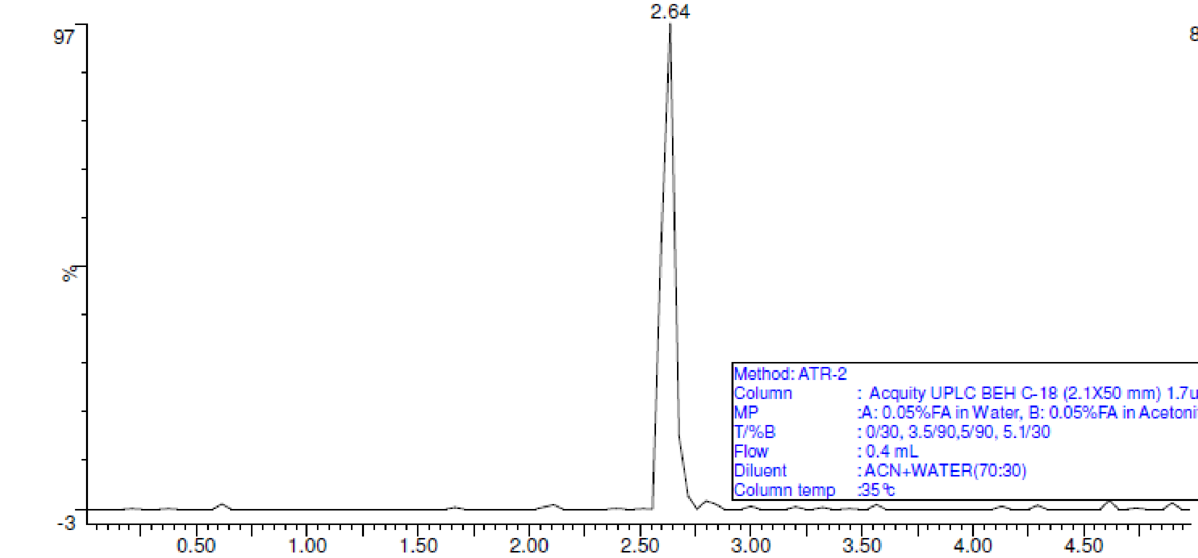

Method: ATR-2
Column : Acquity UPLC BEH C-18 (2.1X50 mm) 1.7u
MP :A: 0.05%FA in Water, B: 0.05%FA in Acetonitrile
T/%B : 0/30, 3.5/90,5/90, 5.1/30
Flow : 0.4 mL
Diluent : ACN+WATER(70:30)
Column temp :35℃

021903C2169-FAN Cluster-5 (C4236-004-A1)A | 1: Scan ES+ TIC 3.01e6

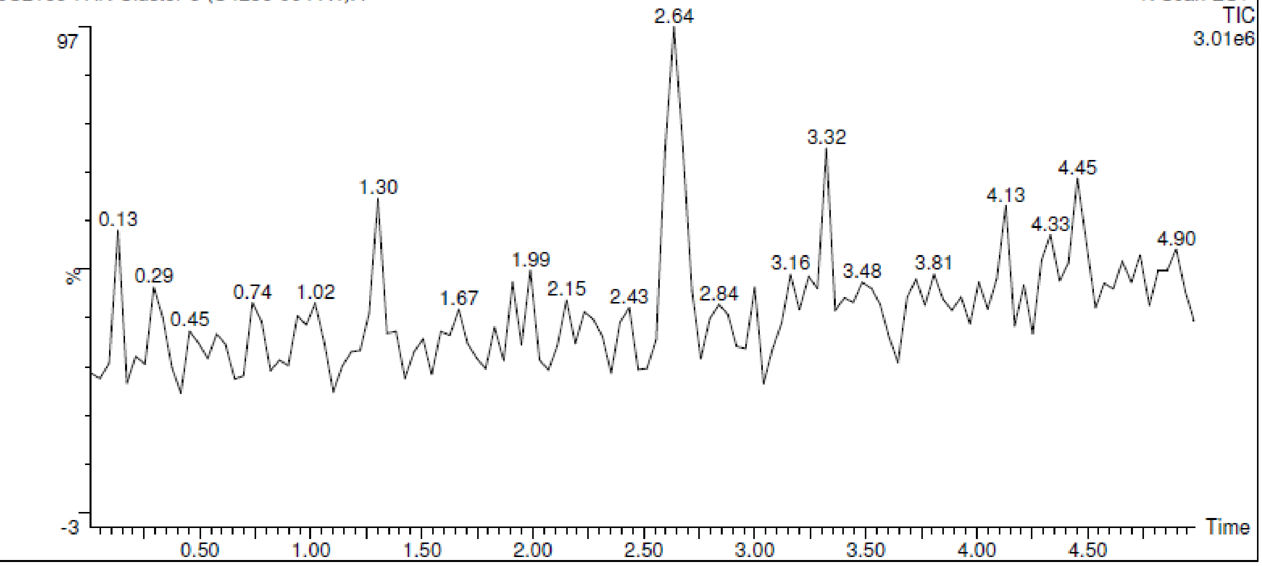

GVK Biosciences Private Limited
Discovery Chemistry-Analytical Services

================================

Sample ID:FAN Cluster-5 (C4236-004-A1) **Date of analysis: 21-Mar-2019:00:15:08**
Acq Method :ATR-2 **Instrument ID:ANL-MCL2-LCMS-001**
2:F,7

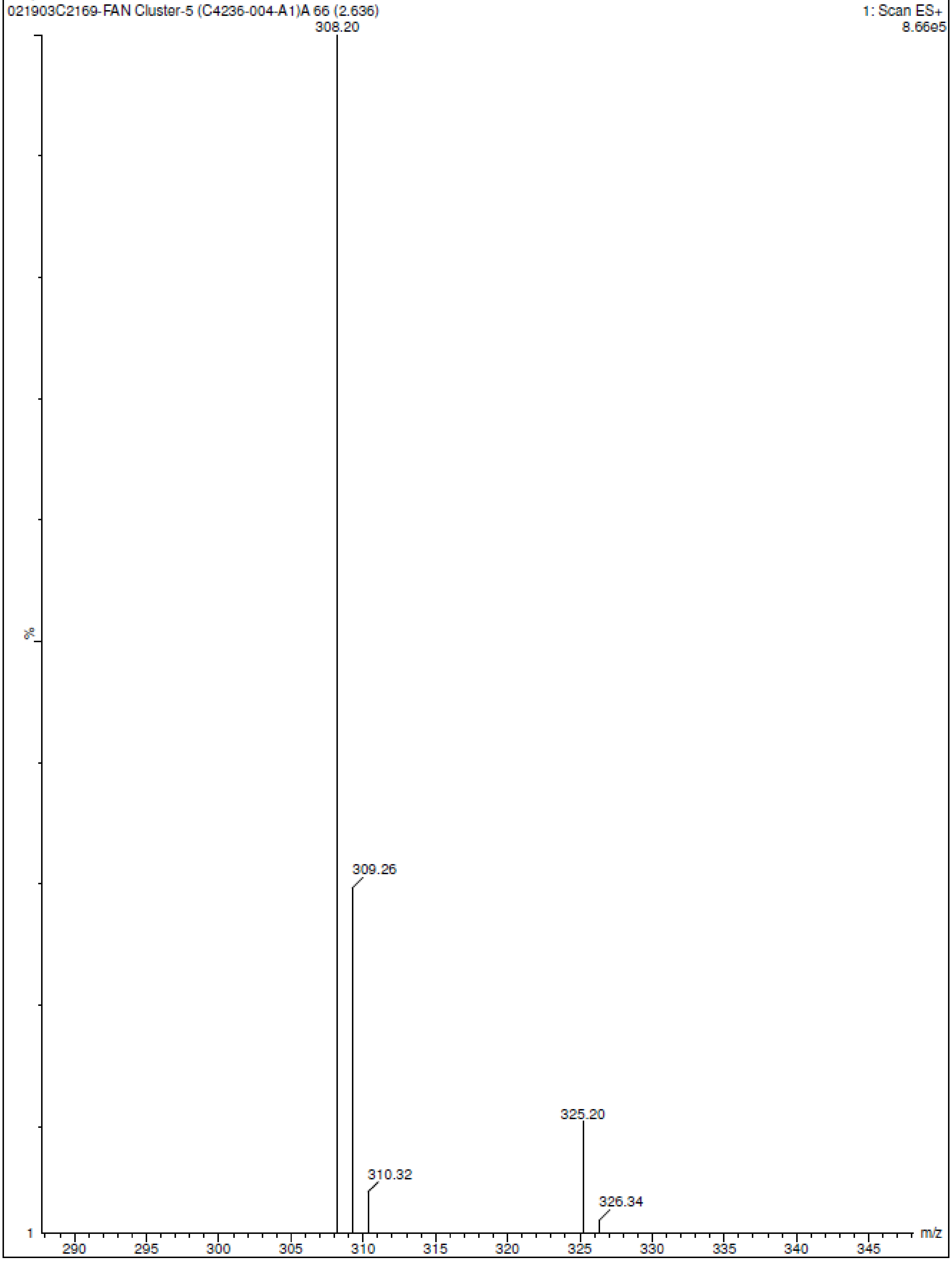

DFA_6 (3f)

S.No	Test	Results
1	**Description**	White Solid
2	**Identification**<br><br>(a) **NMR**<br><br>(b) **Mass by LCMS**	 <br>Complies to structure<br><br>320.29 $[M+H]^+$
3	**Chromatographic Purity by UPLC (Area %)**<br>Impurities>1.0%<br>Rt- 5.68 minutes	97.17<br><br>2.73
4	**Chromatographic Purity by LCMS (Area %)**<br>Impurities>1.0%<br>Rt- 2.17 minutes	96.22<br><br>3.62

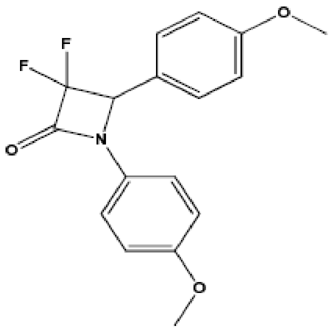

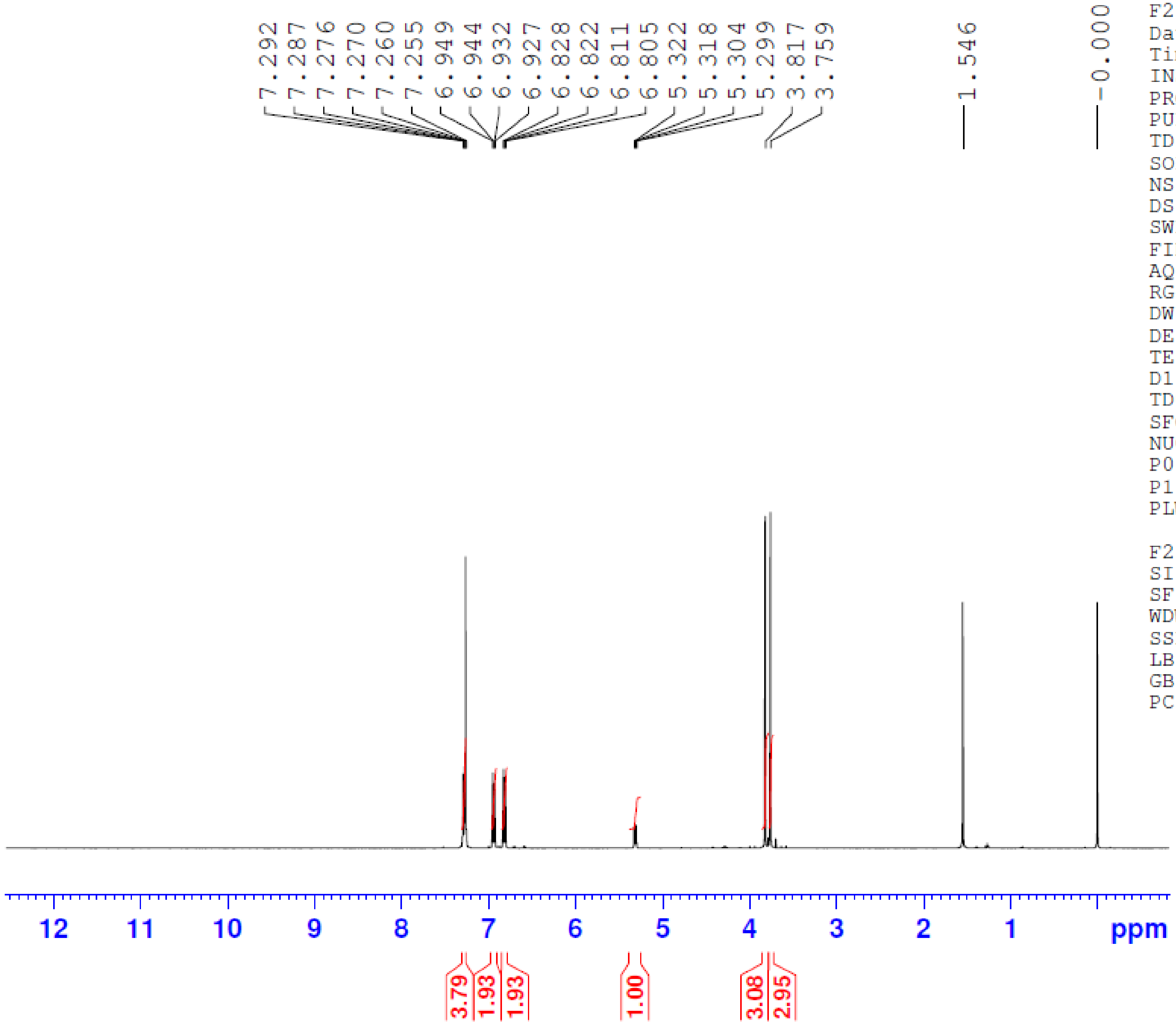

F2 - Acquisition Parameters
Date_ 20190320
Time 18.22 h
INSTRUM Avance Neo
PROBHD Z116098_0787 (
PULPROG zg30
TD 65536
SOLVENT CDCl3
NS 16
DS 0
SWH 7142.857 Hz
FIDRES 0.217983 Hz
AQ 4.5875201 sec
RG 101
DW 70.000 usec
DE 14.62 usec
TE 298.1 K
D1 2.00000000 sec
TD0 1
SFO1 400.4024725 MHz
NUC1 1H
P0 3.33 usec
P1 10.00 usec
PLW1 19.73600006 W

F2 - Processing parameters
SI 65536
SF 400.4000102 MHz
WDW EM
SSB 0
LB 0.30 Hz
GB 0
PC 1.00

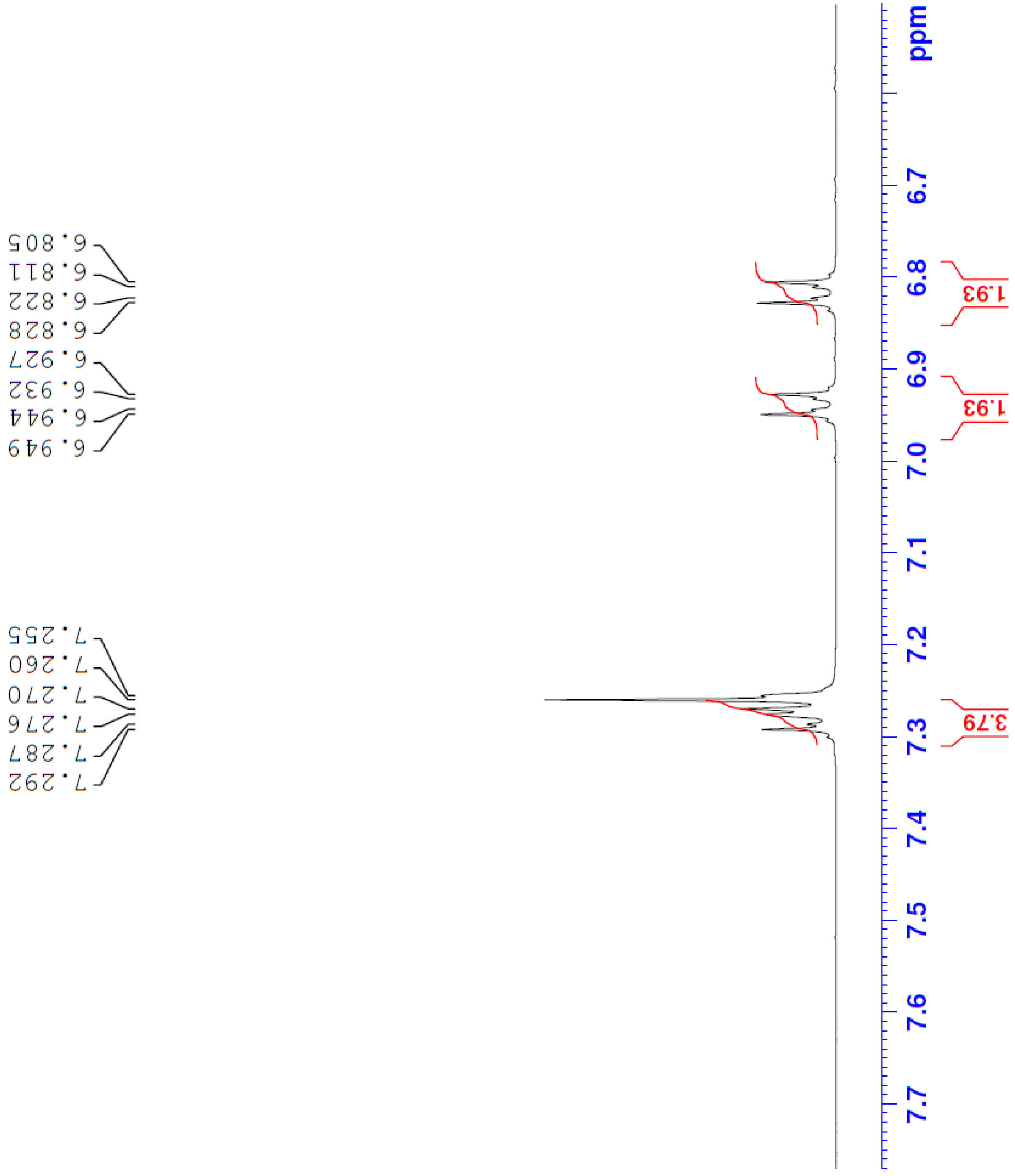

6.805
6.811
6.822
6.828
6.927
6.932
6.944
6.949
7.255
7.260
7.270
7.276
7.287
7.292
1.93
1.93
3.79
ppm
6.7
6.8
6.9
7.0
7.1
7.2
7.3
7.4
7.5
7.6
7.7

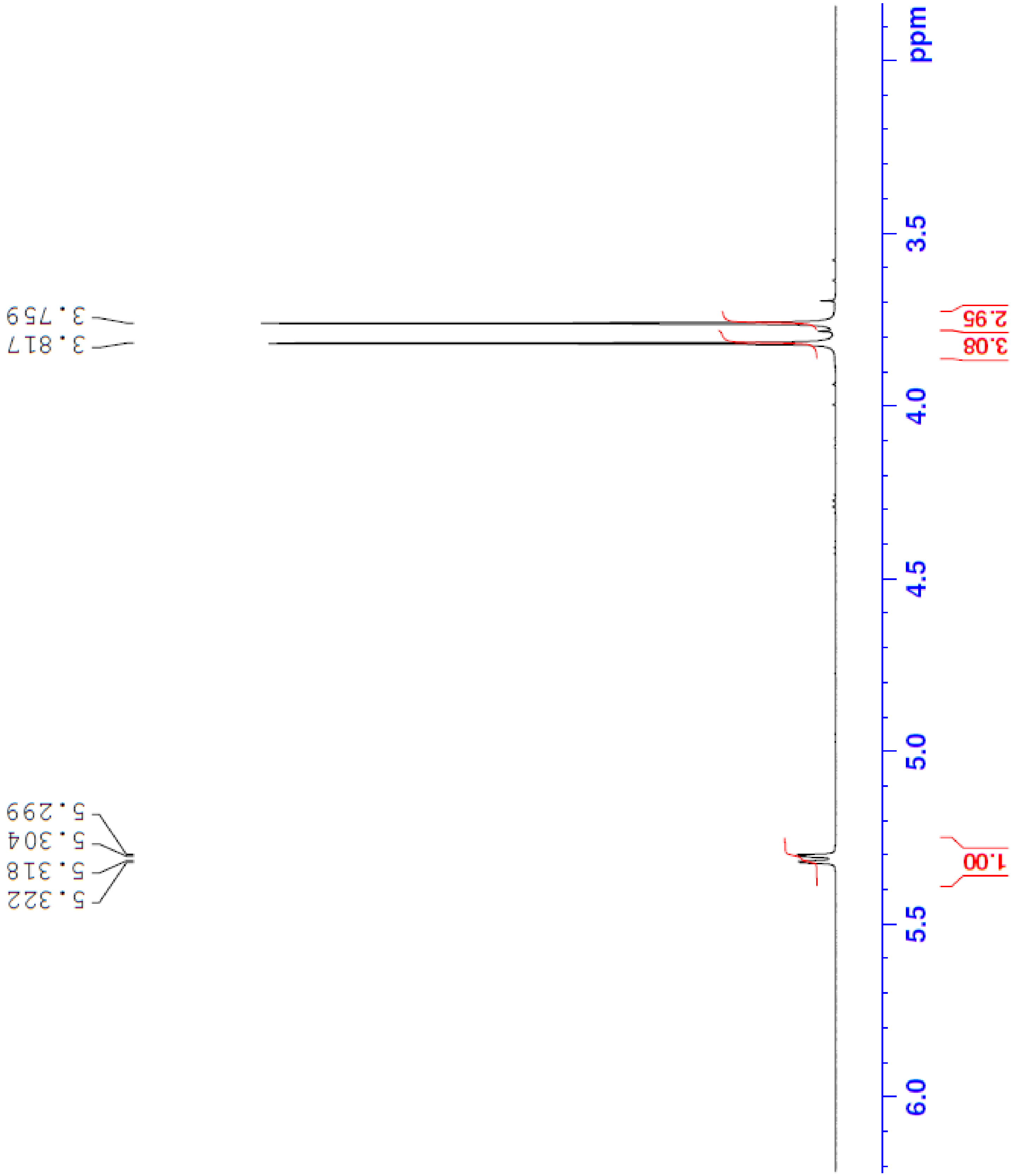
3.759
3.817
5.299
5.304
5.318
5.322
2.95
3.08
1.00
ppm
3.5
4.0
4.5
5.0
5.5
6.0

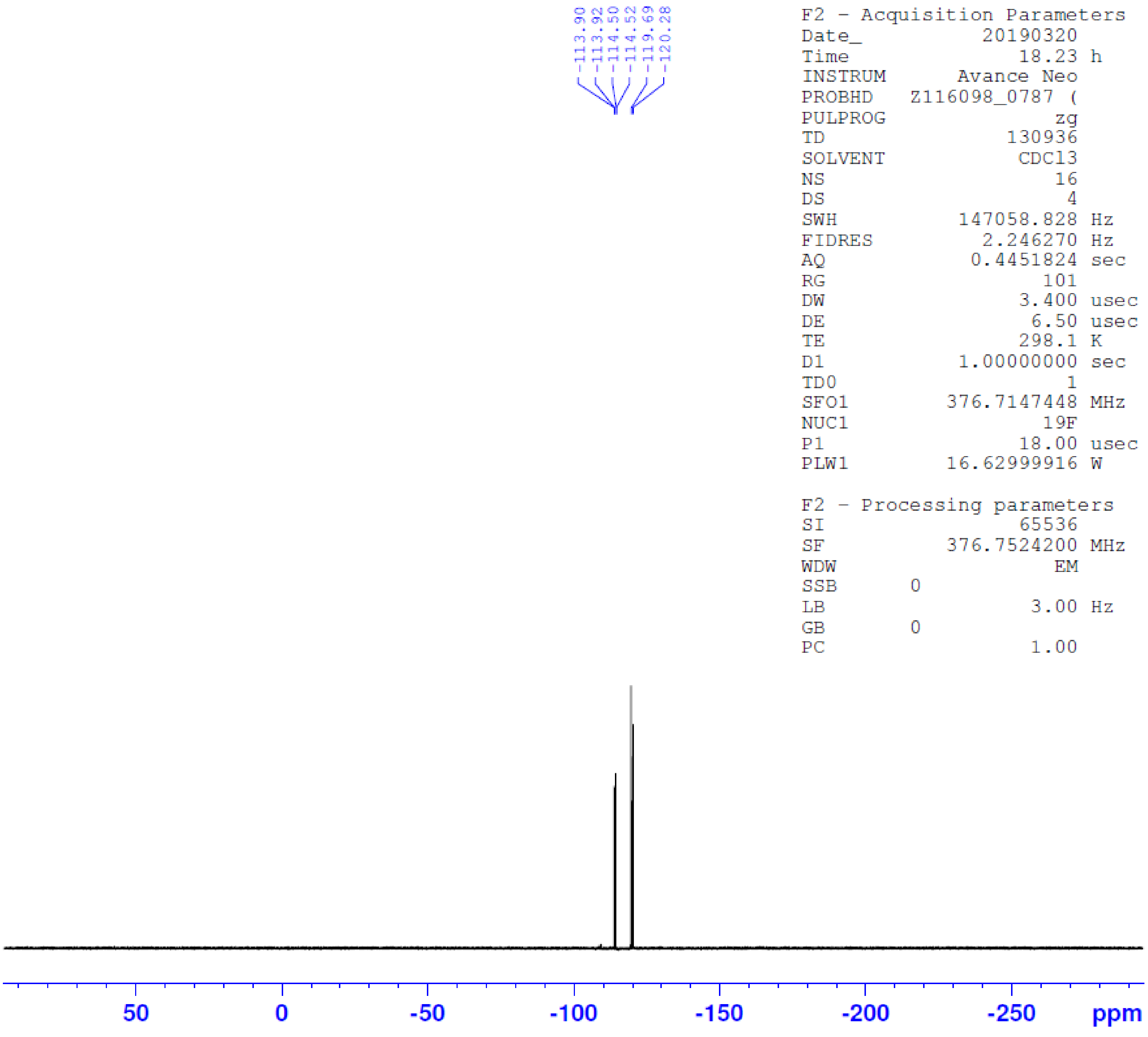
-113.90
-113.92
-114.50
-114.52
-119.69
-120.28
F2 - Acquisition Parameters
Date_ 20190320
Time 18.23 h
INSTRUM Avance Neo
PROBHD Z116098_0787 (
PULPROG zg
TD 130936
SOLVENT CDCl3
NS 16
DS 4
SWH 147058.828 Hz
FIDRES 2.246270 Hz
AQ 0.4451824 sec
RG 101
DW 3.400 usec
DE 6.50 usec
TE 298.1 K
D1 1.00000000 sec
TD0 1
SFO1 376.7147448 MHz
NUC1 19F
P1 18.00 usec
PLW1 16.62999916 W
F2 - Processing parameters
SI 65536
SF 376.7524200 MHz
WDW EM
SSB 0
LB 3.00 Hz
GB 0
PC 1.00
50
0
-50
-100
-150
-200
-250
ppm

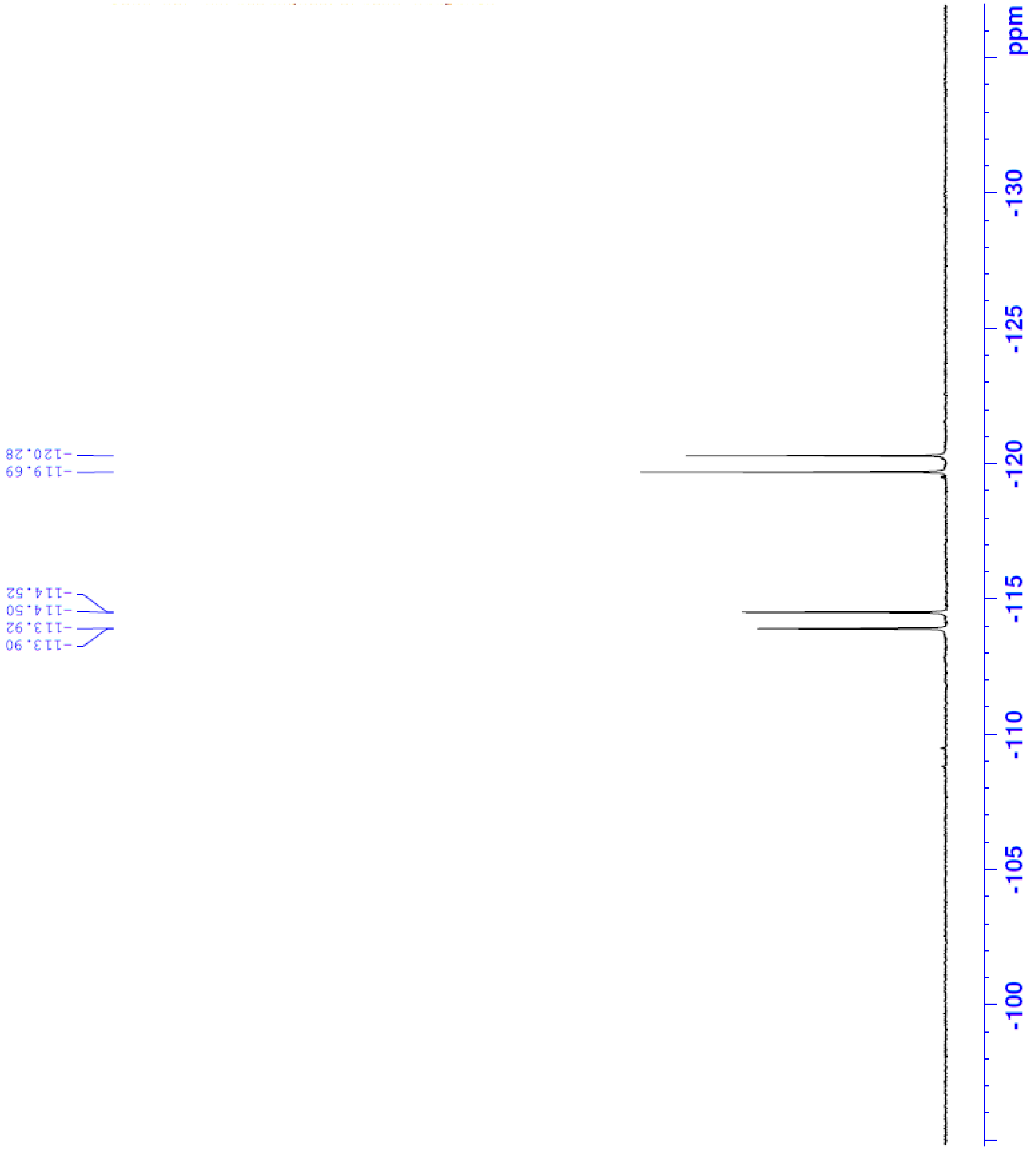
-120.28
-119.69
-114.52
-114.50
-113.92
-113.90
ppm
-130
-125
-120
-115
-110
-105
-100

UPLC Method Conditions :

Column	: Acquity UPLC BEH C18 (2.1x100) mm, 1.7µm
Mobile Phase-A	: 0.05% TFA in Water
Mobile Phase-B	: 0.05% TFA in Acetonitrile
Isocritic	: 50:50(A:B)
Flow Rate	: 0.3 mL/min
Temparature	: 40 °C
Diluent	: ACN+Water

Auto-Scaled Chromatogram

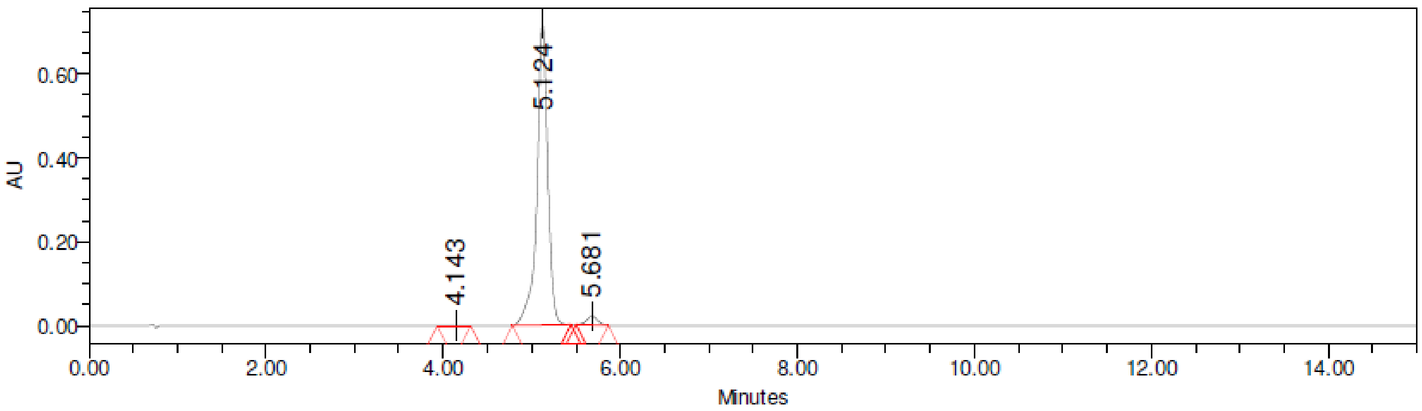

Peak Results

	RT	Area	Height	% Area
1	4.143	6325	711	0.10
2	5.124	6112806	719631	97.17
3	5.681	171821	21060	2.73

Sample ID:FAN Cluster-4 (C3804-125A1)
Acq.Method:
1:B,4

GVK Biosciences Private Limited
Discovery Chemistry-Analytical Services

19-Mar-2019/10:35:39/
Instrument ID: ANL-MCL1-LCMS-002

021903B8867-FAN Cluter-4 (C3804-125A1)A

4: Diode Array
230
Range: 7.757e-1

Time	Height	Area	Area%
2.15	774947	8170.44	96.22
2.17	32316	307.38	3.62
2.52	1367	13.94	0.16

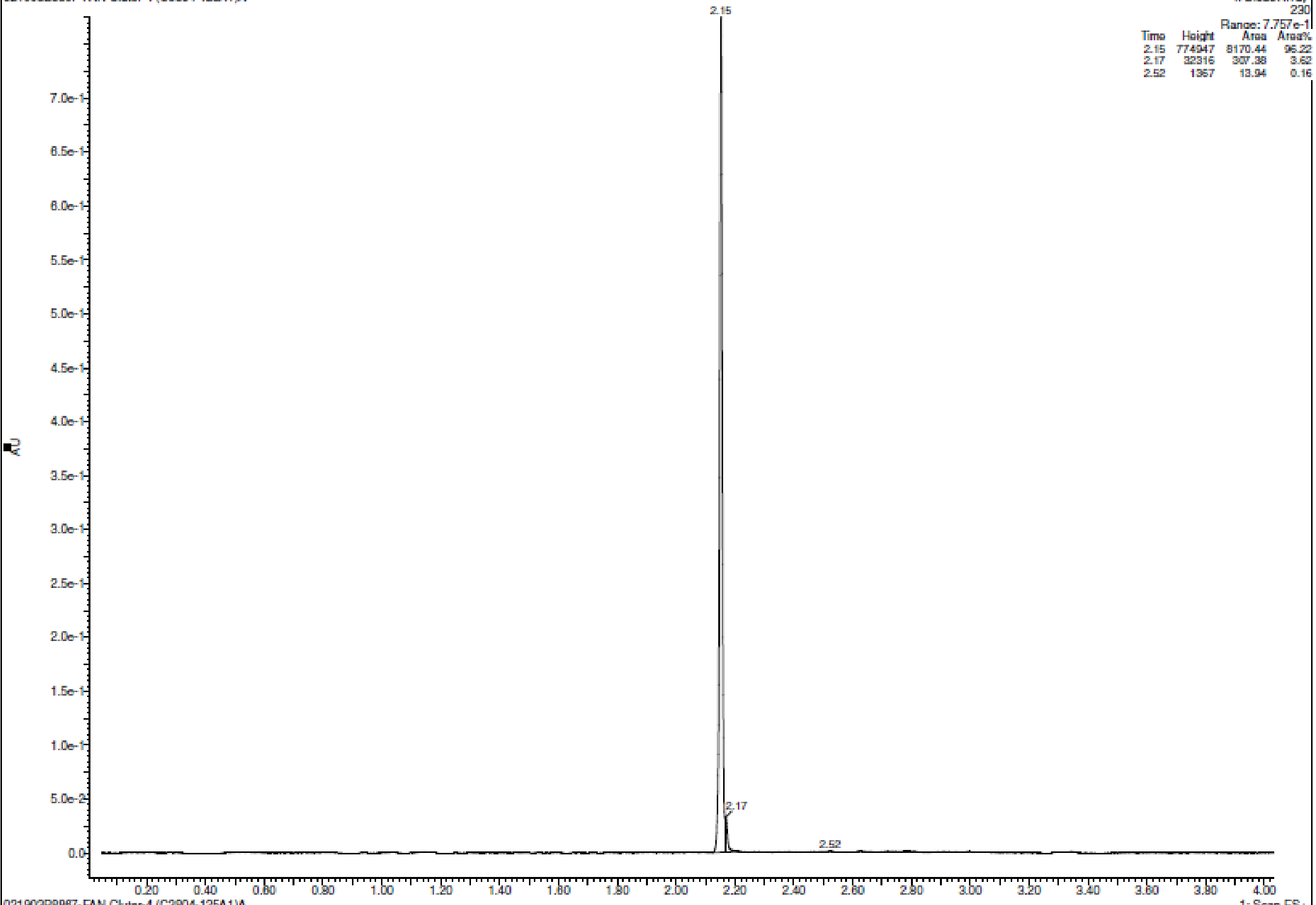

021903B8867-FAN Cluter-4 (C3804-125A1)A

1: Scan ES+
TIC
3.39e7

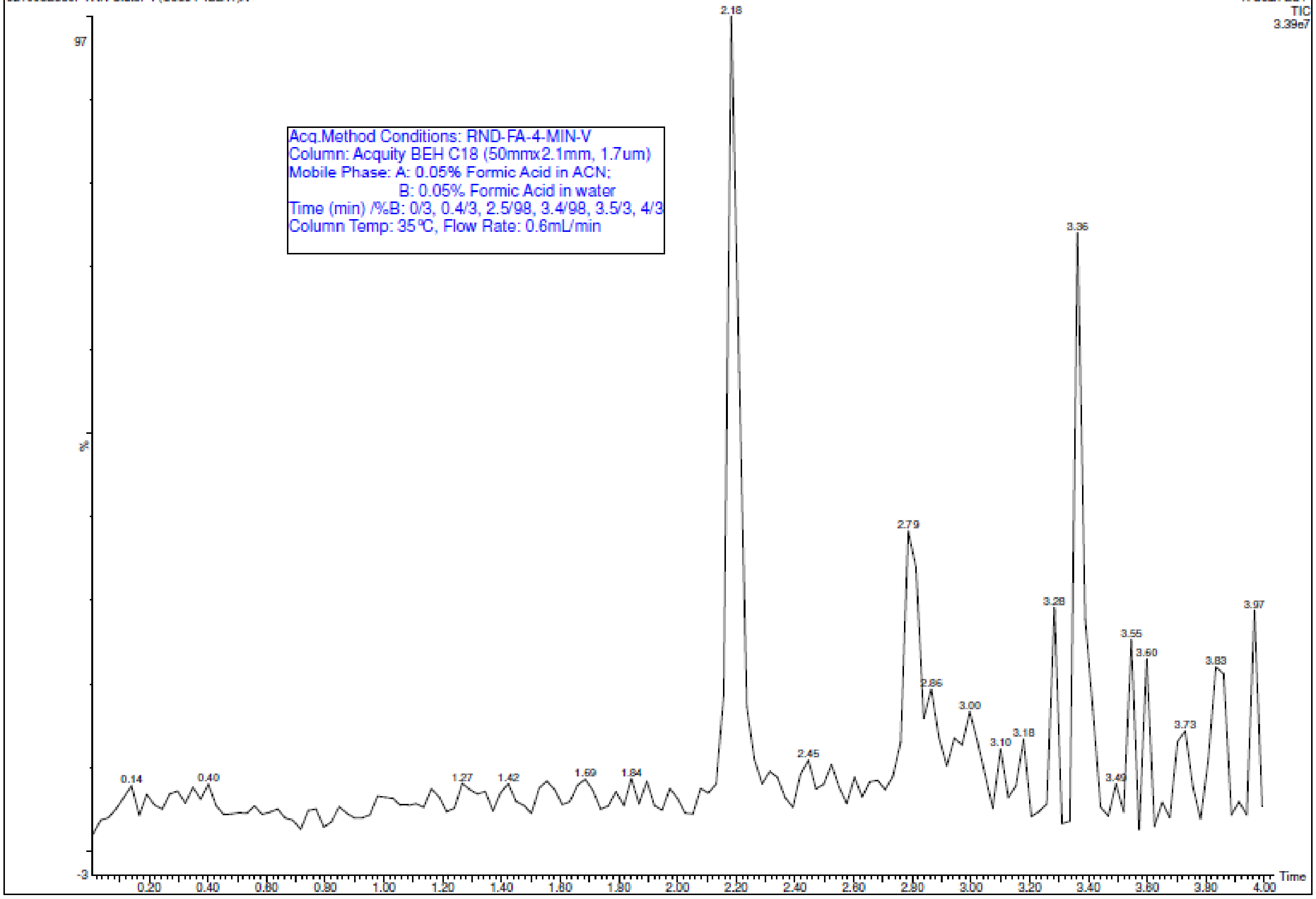

GVK Biosciences Private Limited
Discovery Chemistry-Analytical Services

Sample ID:FAN Cluster-4 (C3804-125A1)
Acq.Method:
1:B,4

Date Of Analysis:19-Mar-2019;10:35:39
Instrument ID:ANL-MCL1-LCMS-002

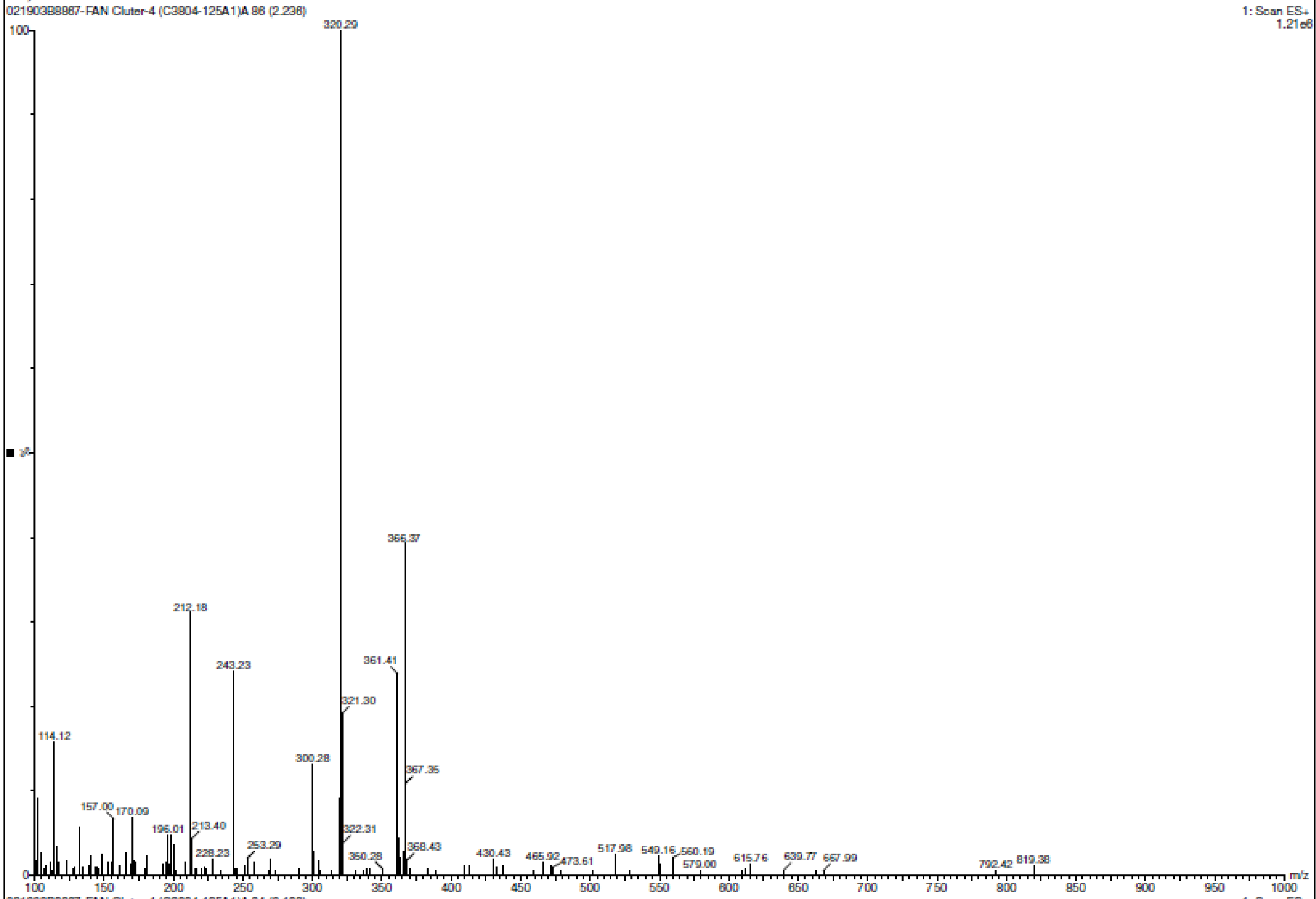

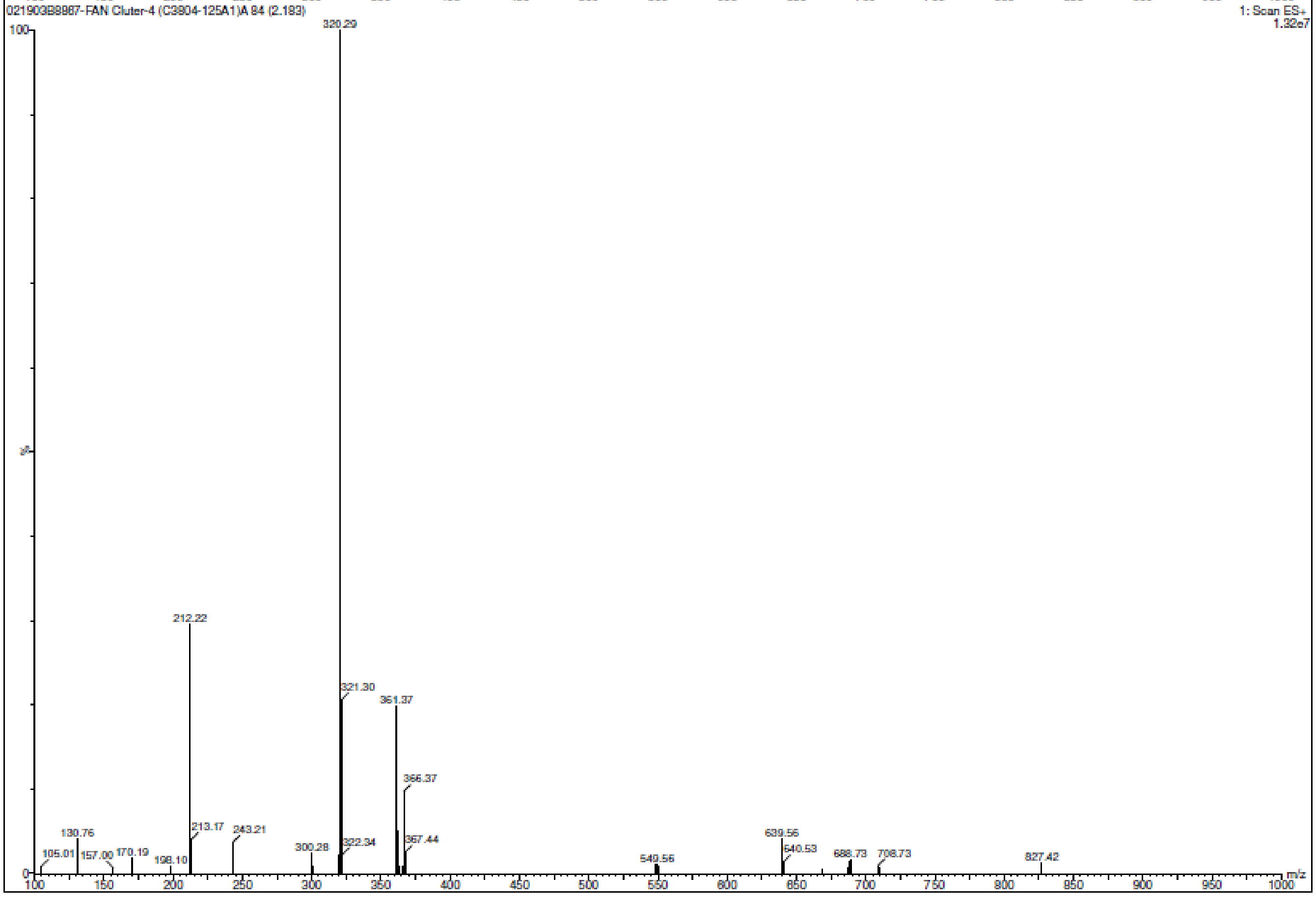

PL_1 (6a)

S.No	Test	Results
1	**Description**	Off-white solid
2	**Identification**<br>(a) **NMR**<br><br>(b) **Mass by LCMS**	 Complies to structure<br><br>445.43 $[M+H]^+$
3	**Chromatographic Purity by UPLC (Area %)**<br>Impurities>1.0%	99.86<br>Nil
4	**Chromatographic Purity by LCMS (Area %)**<br>Impurities>1.0%	99.59<br>Nil

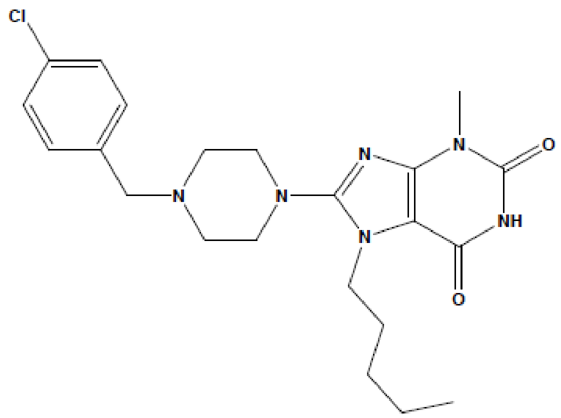

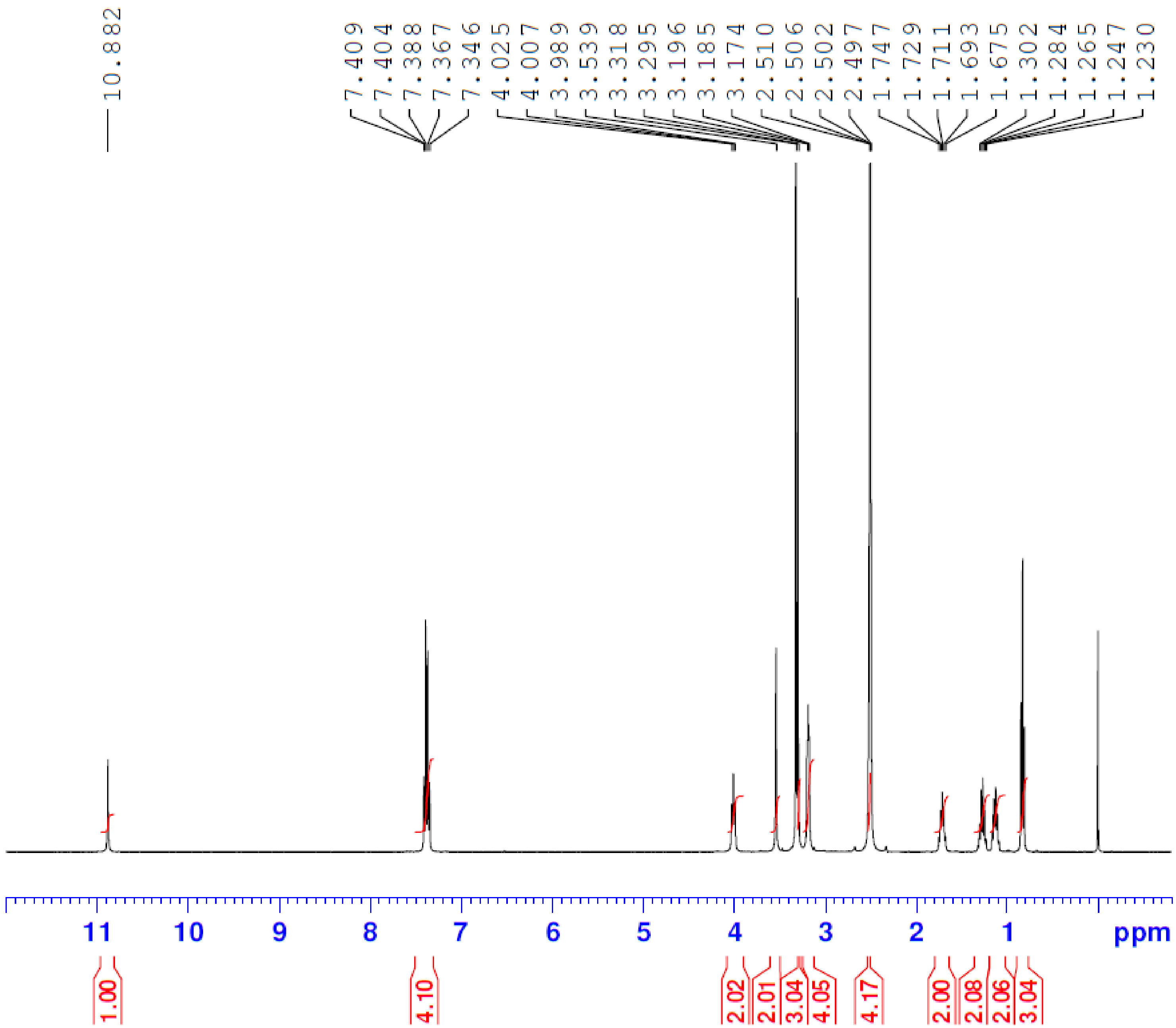

F2 - Acquisition Parameters

Date_	20190705	
Time	18.37	h
INSTRUM	Avance Neo	
PROBHD	Z116098_0787 (	
PULPROG	zg30	
TD	65536	
SOLVENT	DMSO	
NS	16	
DS	0	
SWH	7142.857	Hz
FIDRES	0.217983	Hz
AQ	4.5875201	sec
RG	101	
DW	70.000	usec
DE	14.62	usec
TE	298.1	K
D1	2.00000000	sec
TD0	1	
SFO1	400.4024725	MHz
NUC1	1H	
P0	3.33	usec
P1	10.00	usec
PLW1	19.73600006	W

F2 - Processing parameters

SI	65536	
SF	400.4000017	MHz
WDW	EM	
SSB	0	
LB	0.30	Hz
GB	0	
PC	1.00	

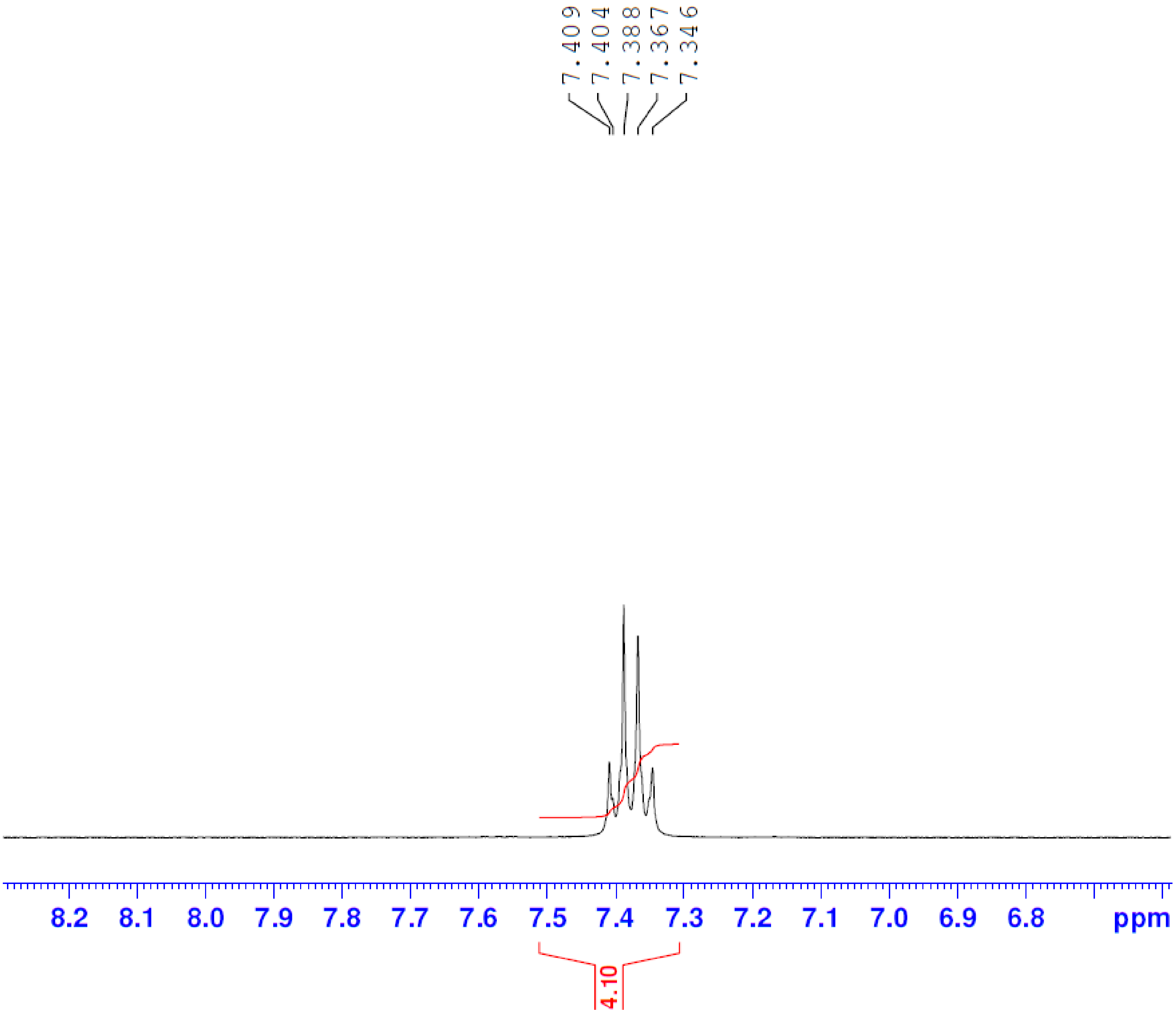

7.409
7.404
7.388
7.367
7.346
8.2
8.1
8.0
7.9
7.8
7.7
7.6
7.5
7.4
7.3
7.2
7.1
7.0
6.9
6.8
ppm
4.10

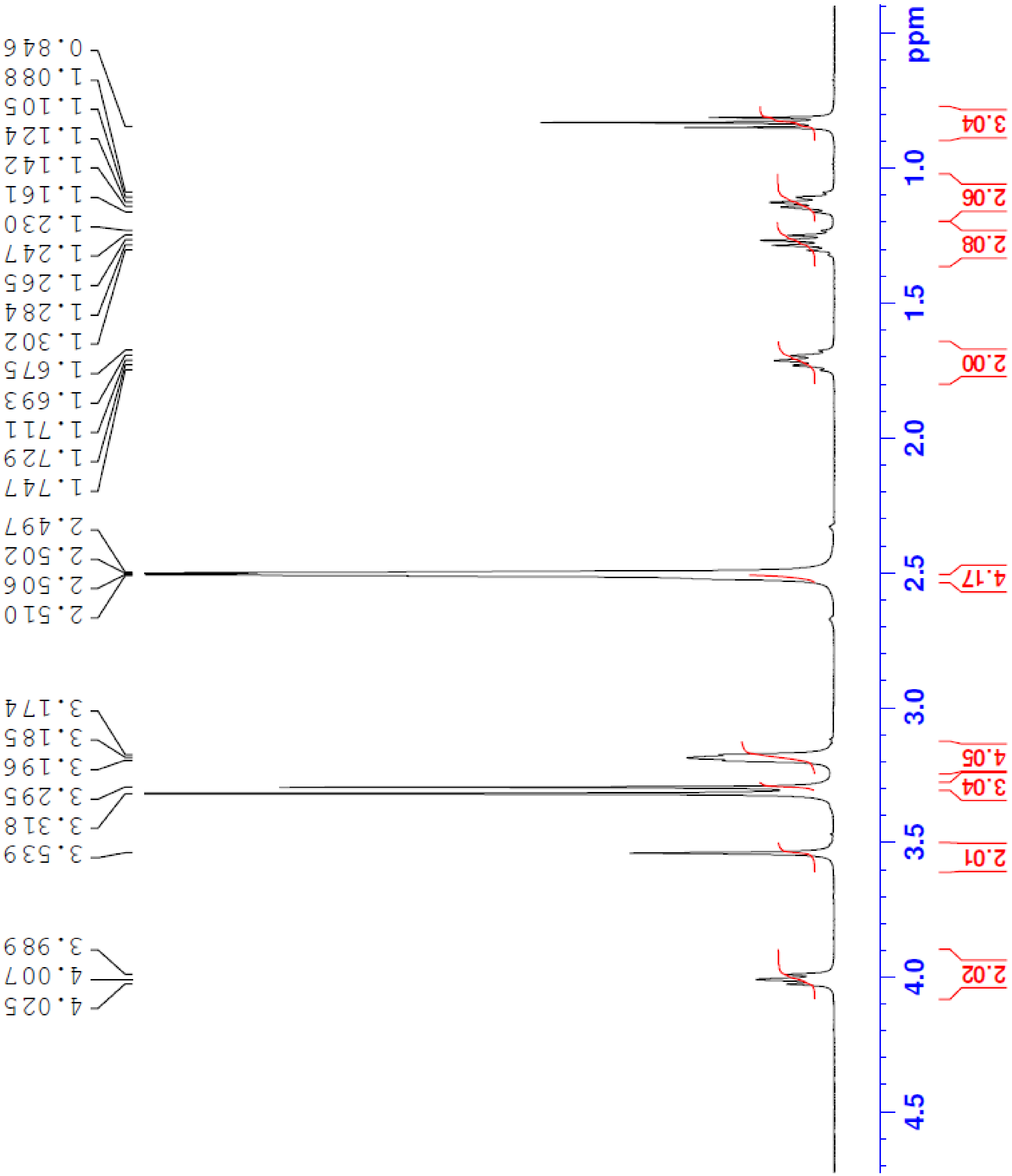
0.846
1.088
1.105
1.124
1.142
1.161
1.230
1.247
1.265
1.284
1.302
1.675
1.693
1.711
1.729
1.747
2.497
2.502
2.506
2.510
3.174
3.185
3.196
3.295
3.318
3.539
3.989
4.007
4.025
ppm
1.0
1.5
2.0
2.5
3.0
3.5
4.0
4.5
3.04
2.06
2.08
2.00
4.17
4.05
3.04
2.01
2.02

D_2O Exchange

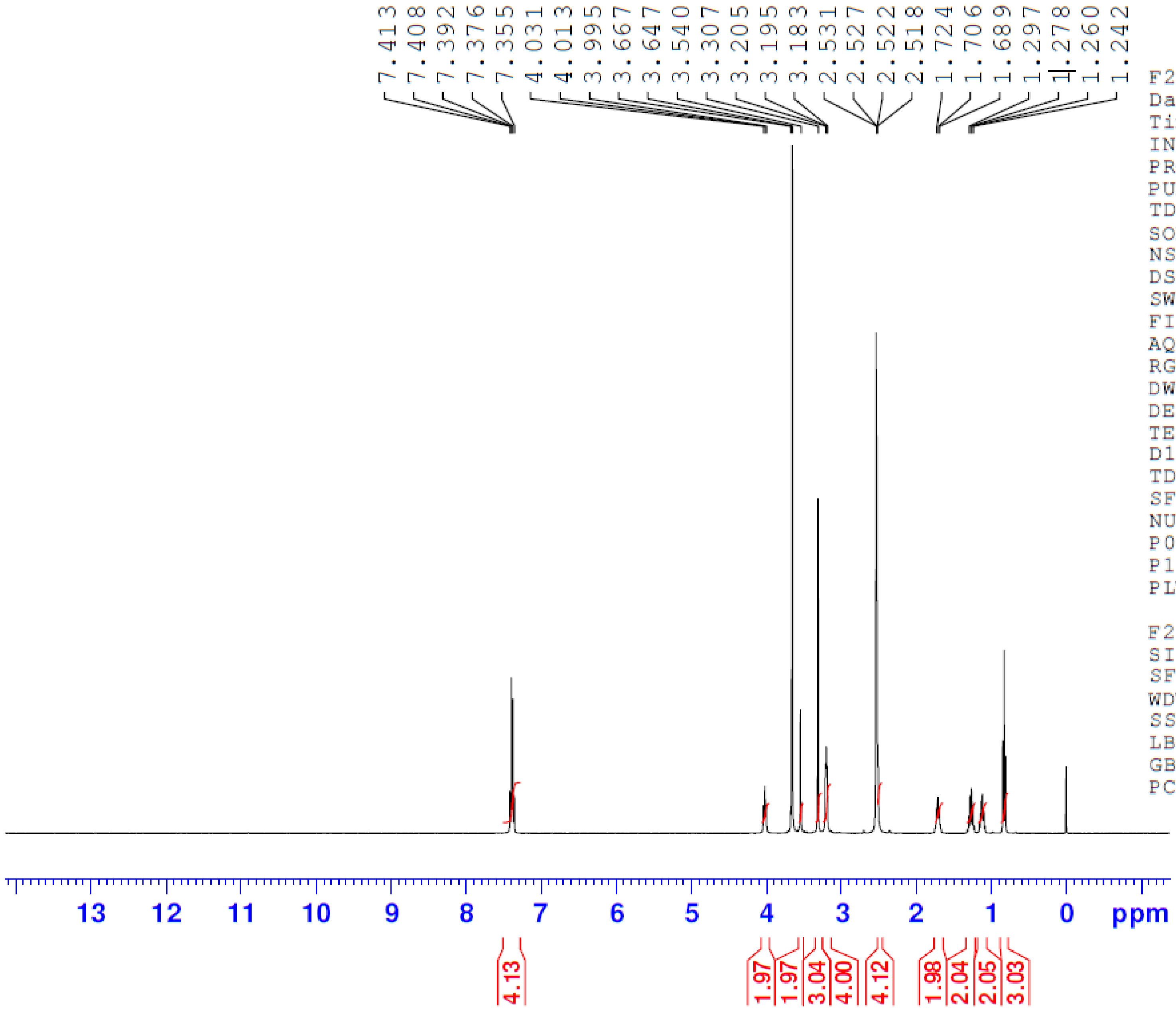

F2 - Acquisition Parameters

Date_	20190705	
Time	23.45	h
INSTRUM	Avance Neo	
PROBHD	Z116098_0787 (	
PULPROG	zg30	
TD	65536	
SOLVENT	DMSO	
NS	16	
DS	0	
SWH	7142.857	Hz
FIDRES	0.217983	Hz
AQ	4.5875201	sec
RG	101	
DW	70.000	usec
DE	14.62	usec
TE	298.1	K
D1	2.00000000	sec
TD0	1	
SFO1	400.4024725	MHz
NUC1	1H	
P0	3.33	usec
P1	10.00	usec
PLW1	19.73600006	W

F2 - Processing parameters

SI	65536	
SF	400.3999920	MHz
WDW	EM	
SSB	0	
LB	0.30	Hz
GB	0	
PC	1.00	

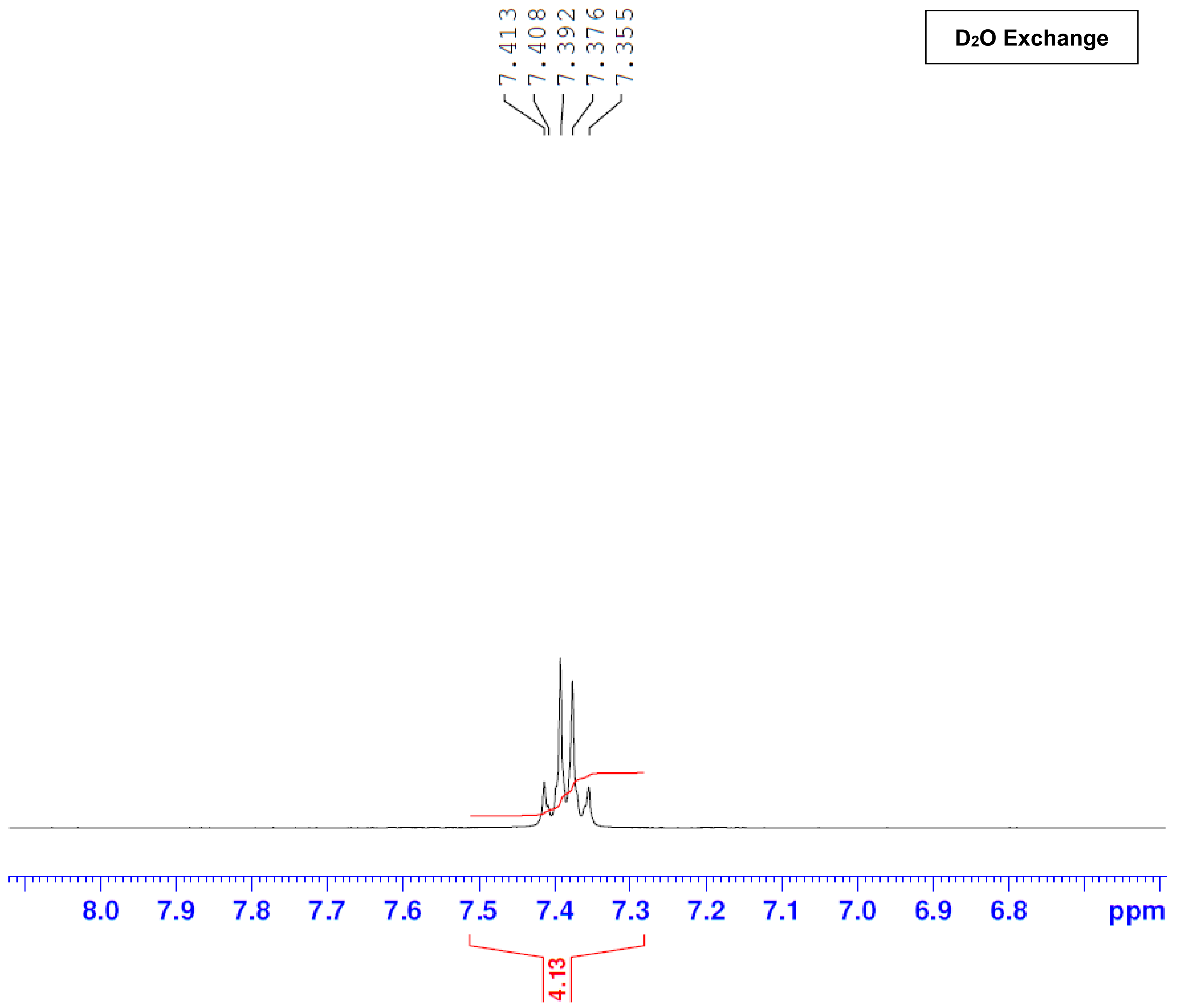
D2O Exchange
7.413
7.408
7.392
7.376
7.355
8.0
7.9
7.8
7.7
7.6
7.5
7.4
7.3
7.2
7.1
7.0
6.9
6.8
ppm
4.13

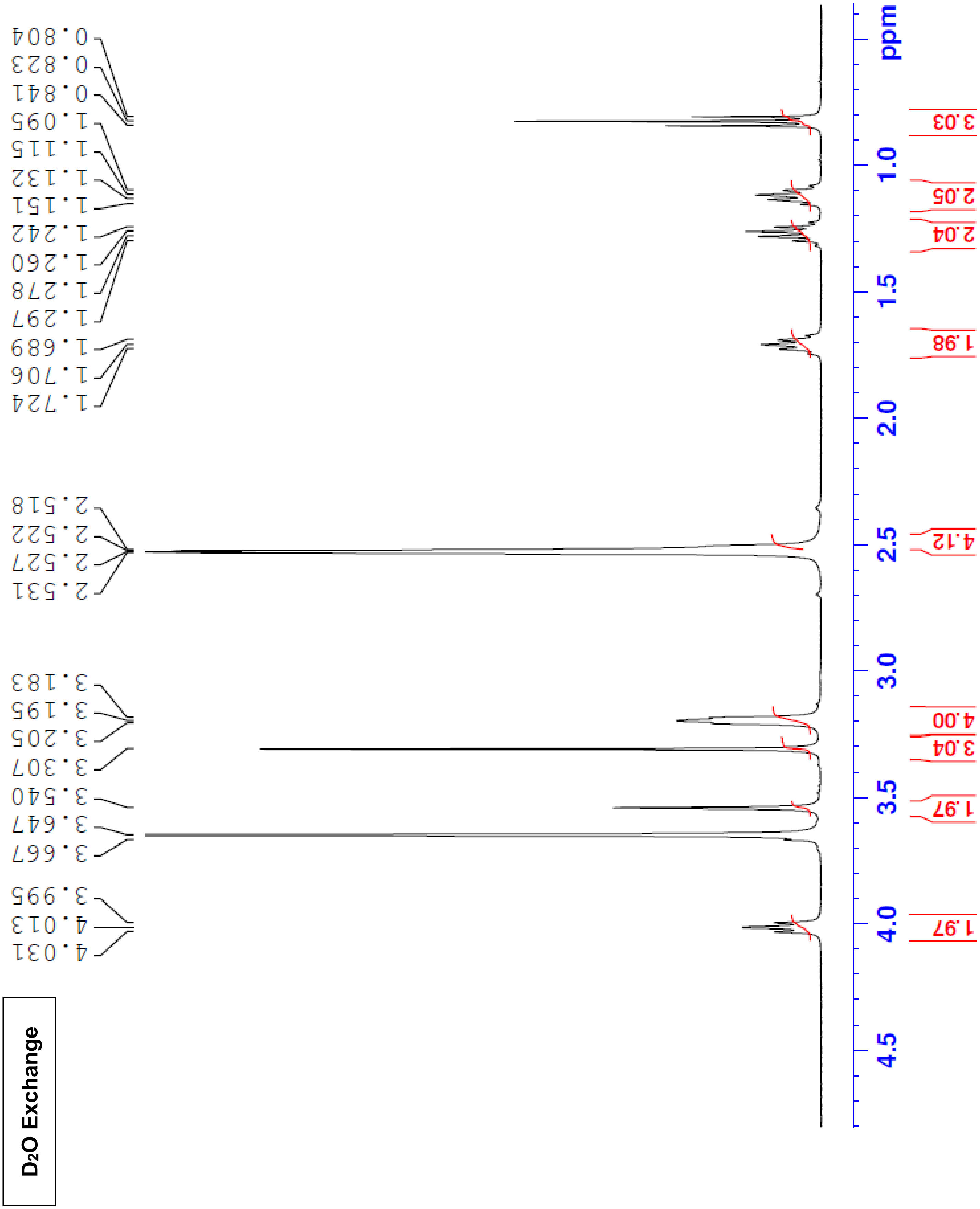
D2O Exchange
ppm
1.0
1.5
2.0
2.5
3.0
3.5
4.0
4.5
3.03
2.05
2.04
1.98
4.12
4.00
3.04
1.97
1.97
0.804
0.823
0.841
1.095
1.115
1.132
1.151
1.242
1.260
1.278
1.297
1.689
1.706
1.724
2.518
2.522
2.527
2.531
3.183
3.195
3.205
3.307
3.540
3.647
3.667
3.995
4.013
4.031

UPLC Method Conditions :

Column	: Acquity UPLC BEH C18 (2.1x100) mm, 1.7µm
Mobile Phase-A	: 0.05% TFA in Water
Mobile Phase-B	: 0.05% TFA in Acetonitrile
Gradient (T/% B)	: 0/10,4/90,6/90,6.1/10
Flow Rate	: 0.3 mL/min
Temparature	: 40℃
Diluent	: ACN+Water

Auto-Scaled Chromatogram

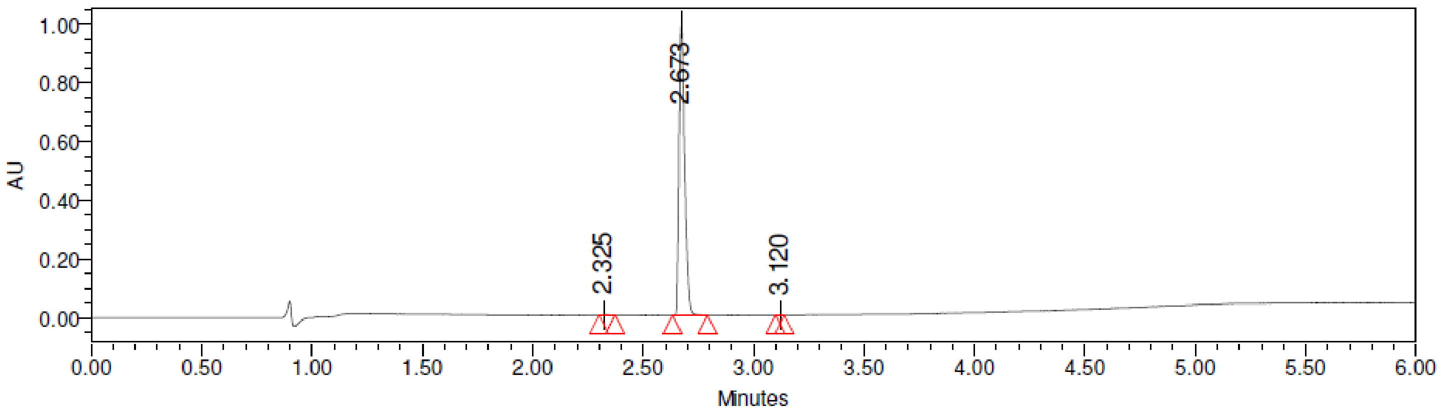

Peak Results

	RT	Area	Height	% Area
1	2.325	1456	1030	0.09
2	2.673	1706865	993842	99.86
3	3.120	938	737	0.05

GVK BIO Sciences Private Limited
Discovery Chemistry-Analytical Services
==

Sample ID :X Cluster Set-1-Target 1 (C3804-190A1) **Date of analysis : 06-Jul-2019/11:56:34**
Acq Method :ATR-1 **Instrument ID: ANL-MCL2-LCMS-001**
2:C,1

021907A7806_X Cluster Set-1-Target 1 (C3804-190A1)-AA

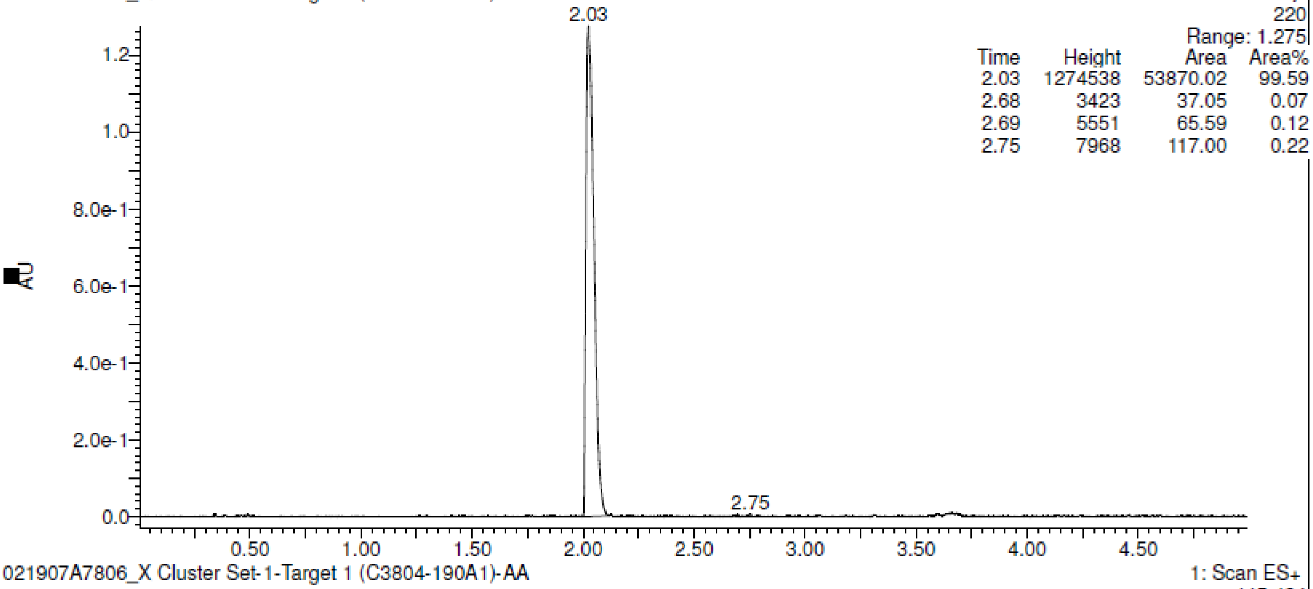

Time	Height	Area	Area%
2.03	1274538	53870.02	99.59
2.68	3423	37.05	0.07
2.69	5551	65.59	0.12
2.75	7968	117.00	0.22

021907A7806_X Cluster Set-1-Target 1 (C3804-190A1)-AA

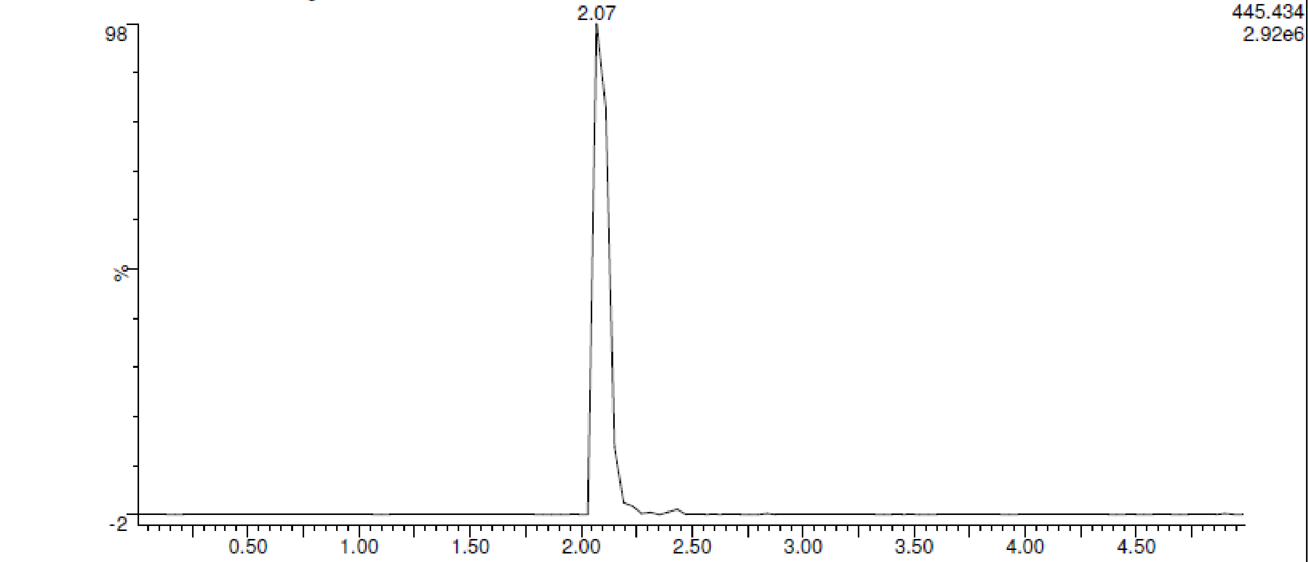

021907A7806_X Cluster Set-1-Target 1 (C3804-190A1)-AA

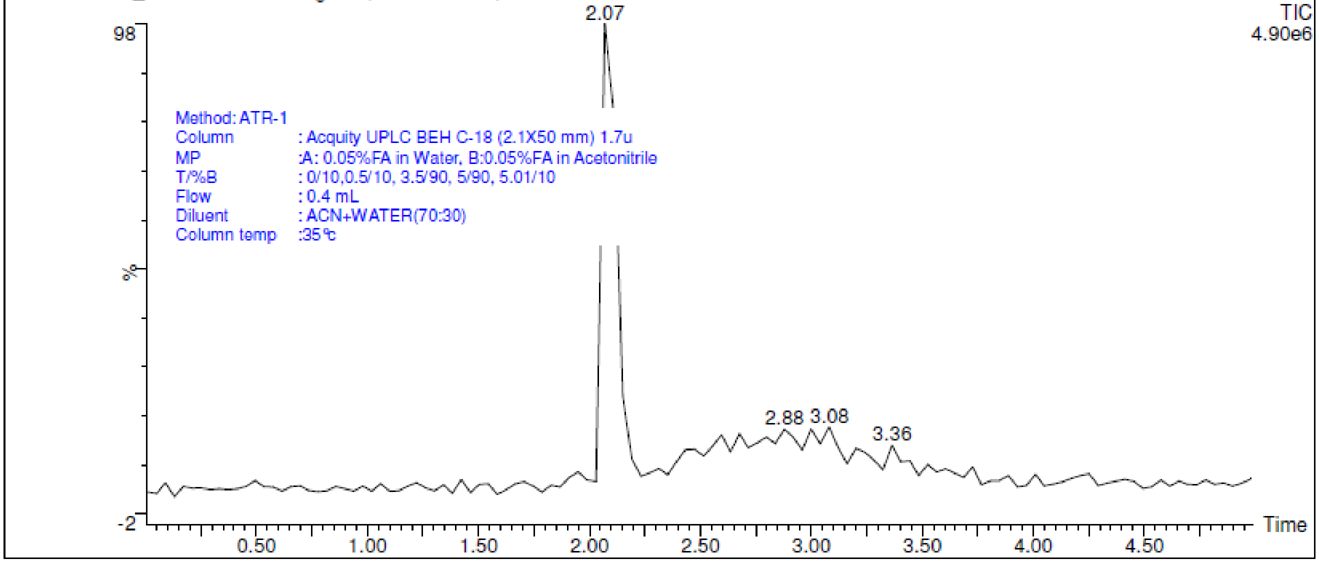

GVK Biosciences Private Limited
Discovery Chemistry-Analytical Services
================================

Sample ID: X Cluster Set-1-Target 1 (C3804-190A1) **Date of analysis: 06-Jul-2019:11:56:34**
Acq Method :ATR-1 **Instrument ID:ANL-MCL2-LCMS-001**
2:C,1

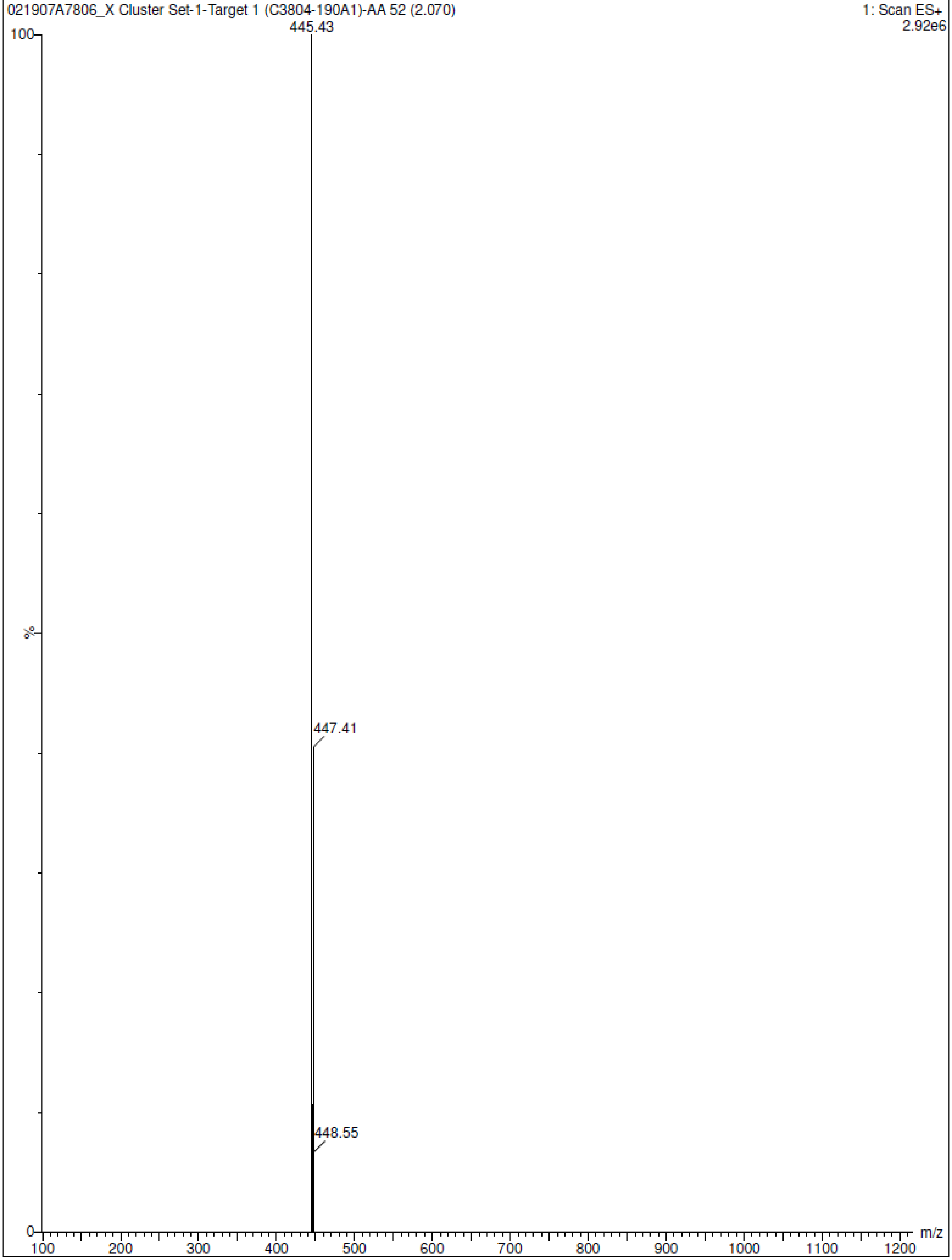

PL_2 (6b)

S.No	Test	Results
1	**Description**	Off white solid
2	**Identification**<br>(a) **NMR**<br>(b) **Mass by LCMS**	 Complies to structure<br>431.24 $[M+H]^+$
3	**Chromatographic Purity by UPLC (Area %)**<br>Impurities>1.0%<br>Rt- 2.73 minutes	98.65<br><br>1.16
4	**Chromatographic Purity by LCMS (Area %)**<br>Impurities>1.0%<br>Rt- 2.11 minutes	98.29<br><br>1.65

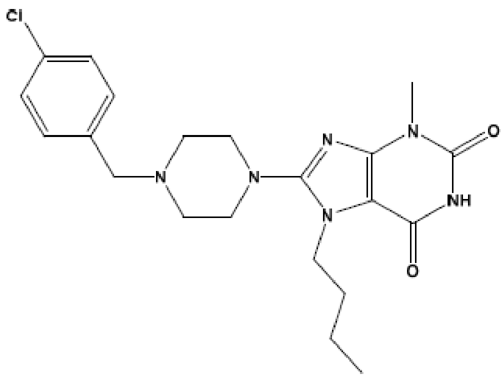

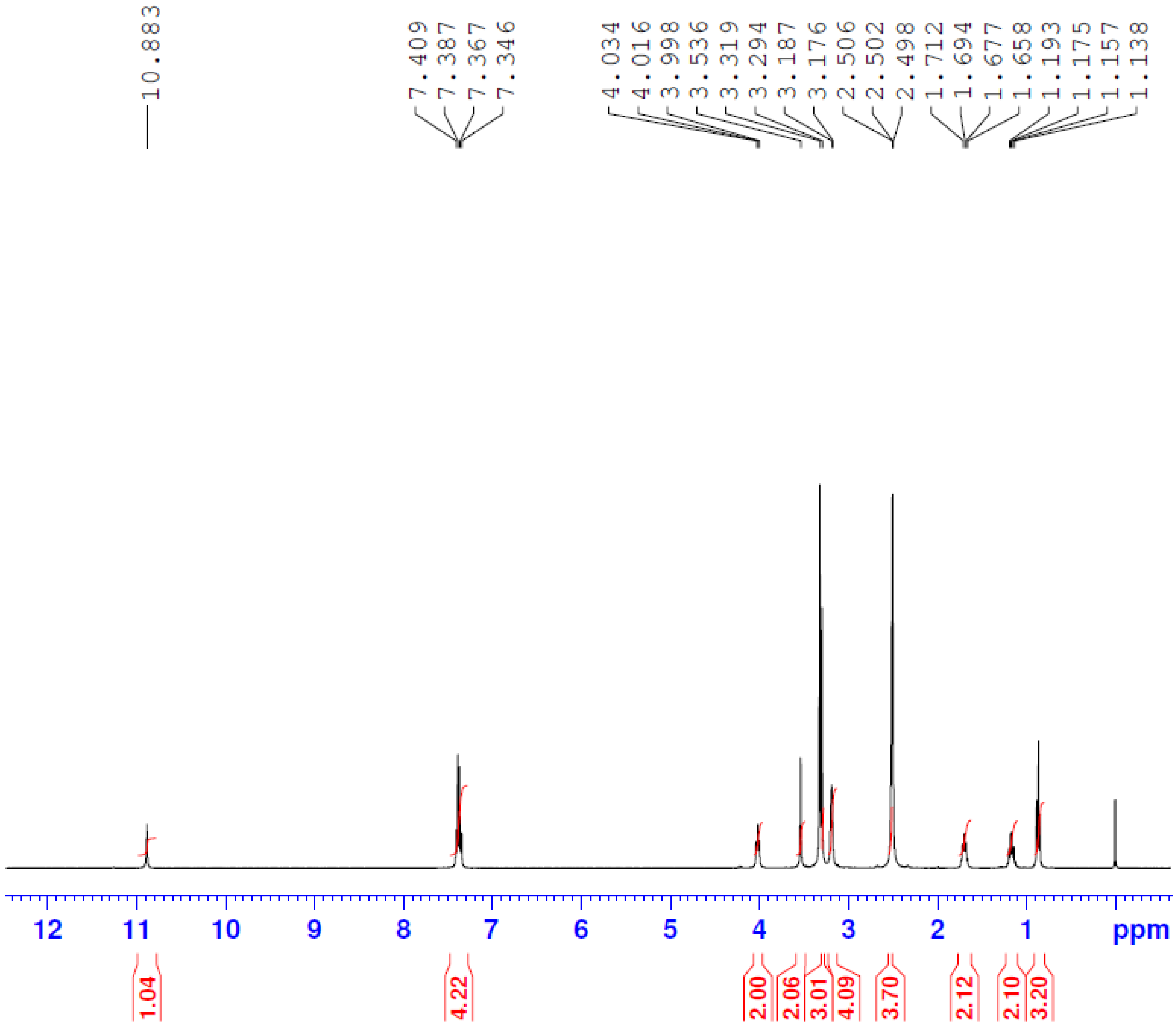

F2 - Acquisition Parameters
Date_ 20190710
Time 23.44 h
INSTRUM Avance Neo
PROBHD Z116098_0787 (
PULPROG zg30
TD 65536
SOLVENT DMSO
NS 8
DS 0
SWH 7142.857 Hz
FIDRES 0.217983 Hz
AQ 4.5875201 sec
RG 101
DW 70.000 usec
DE 14.62 usec
TE 298.1 K
D1 2.00000000 sec
TD0 1
SFO1 400.4024725 MHz
NUC1 1H
P0 3.33 usec
P1 10.00 usec
PLW1 19.73600006 W

F2 - Processing parameters
SI 65536
SF 400.4000016 MHz
WDW EM
SSB 0
LB 0.30 Hz
GB 0
PC 1.00

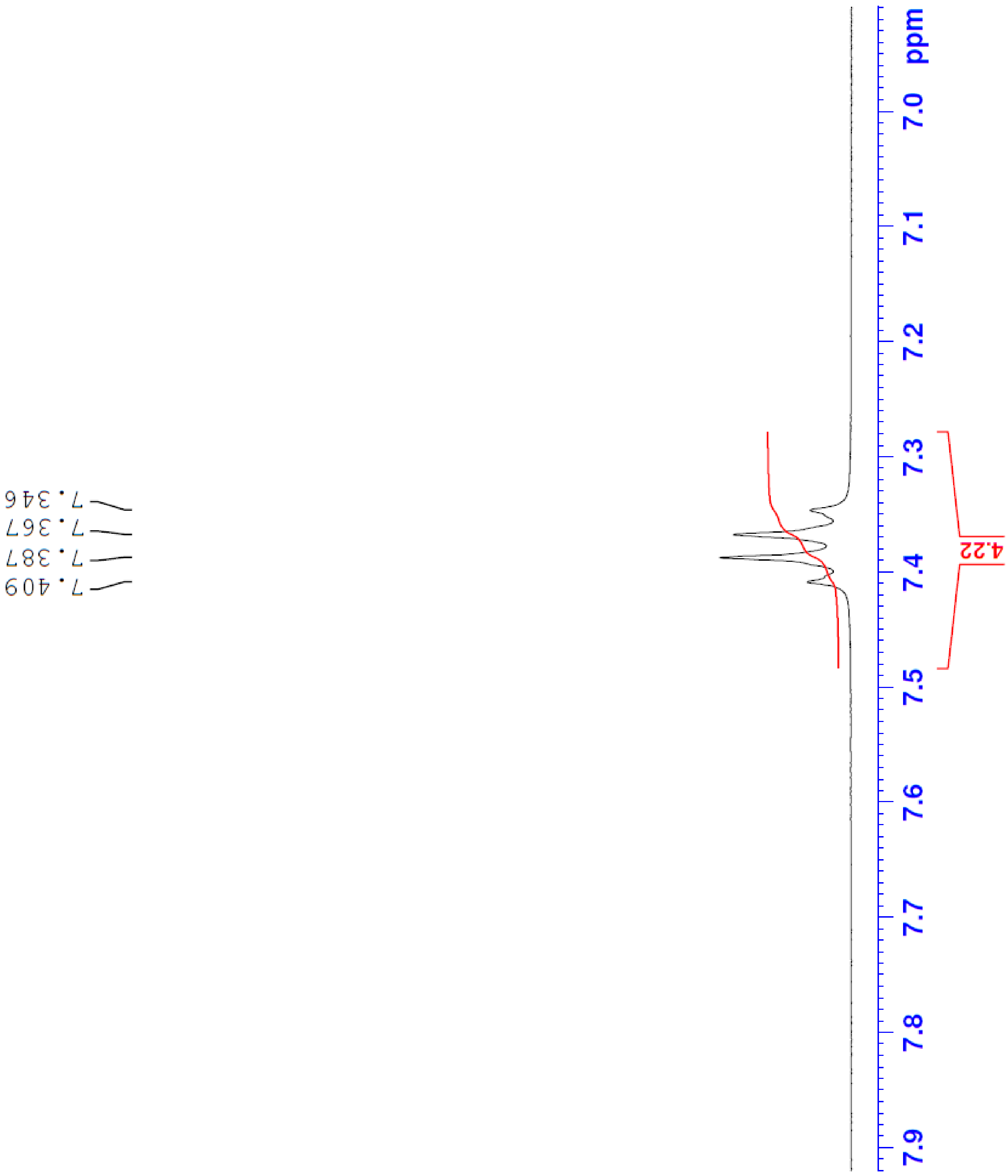
7.346
7.367
7.387
7.409
4.22
ppm
7.0
7.1
7.2
7.3
7.4
7.5
7.6
7.7
7.8
7.9

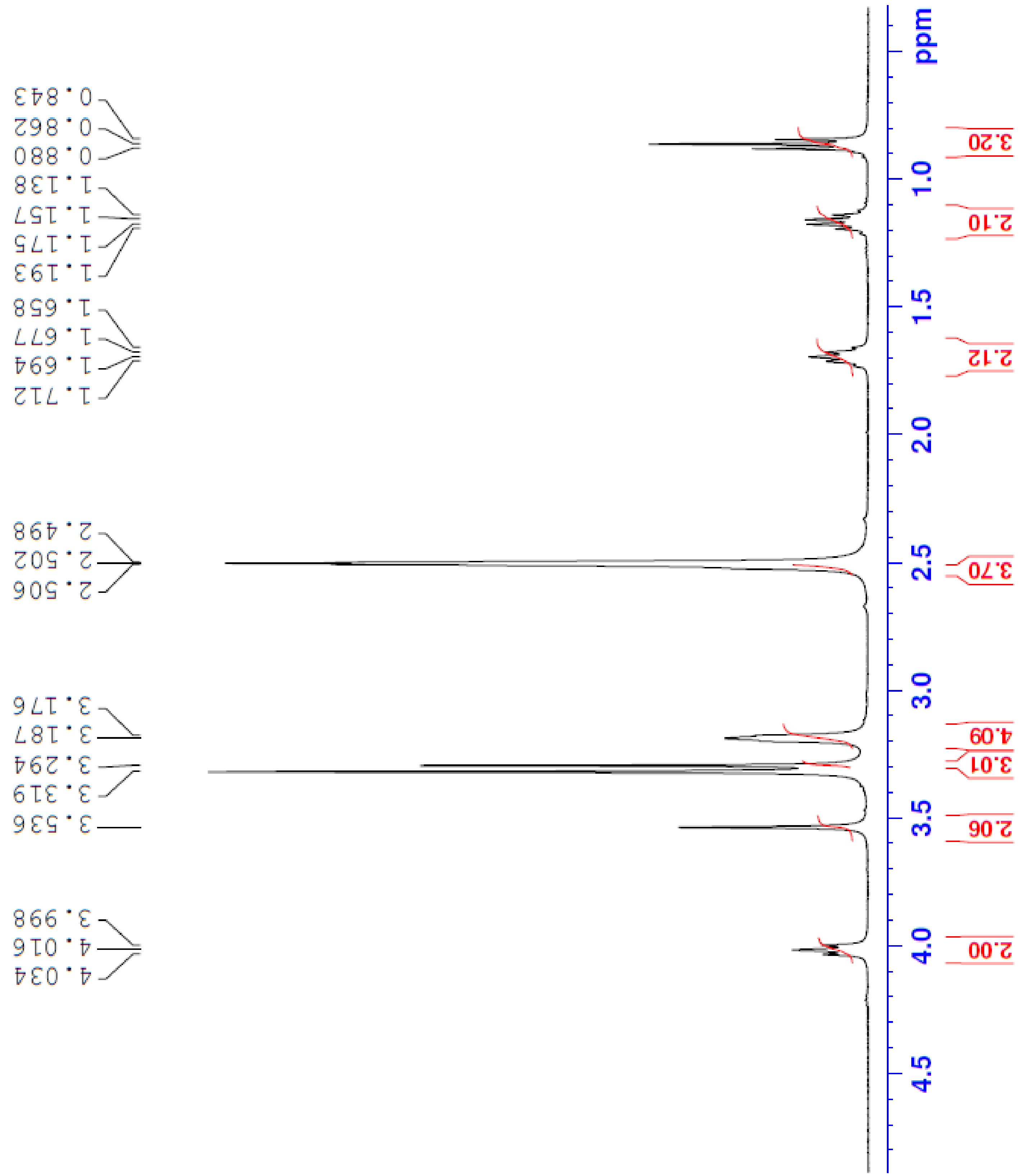
0.843
0.862
0.880
1.138
1.157
1.175
1.193
1.658
1.677
1.694
1.712
2.498
2.502
2.506
3.176
3.187
3.294
3.319
3.536
3.998
4.016
4.034
3.20
2.10
2.12
3.70
4.09
3.01
2.06
2.00
ppm
1.0
1.5
2.0
2.5
3.0
3.5
4.0
4.5

D_2O Exchange

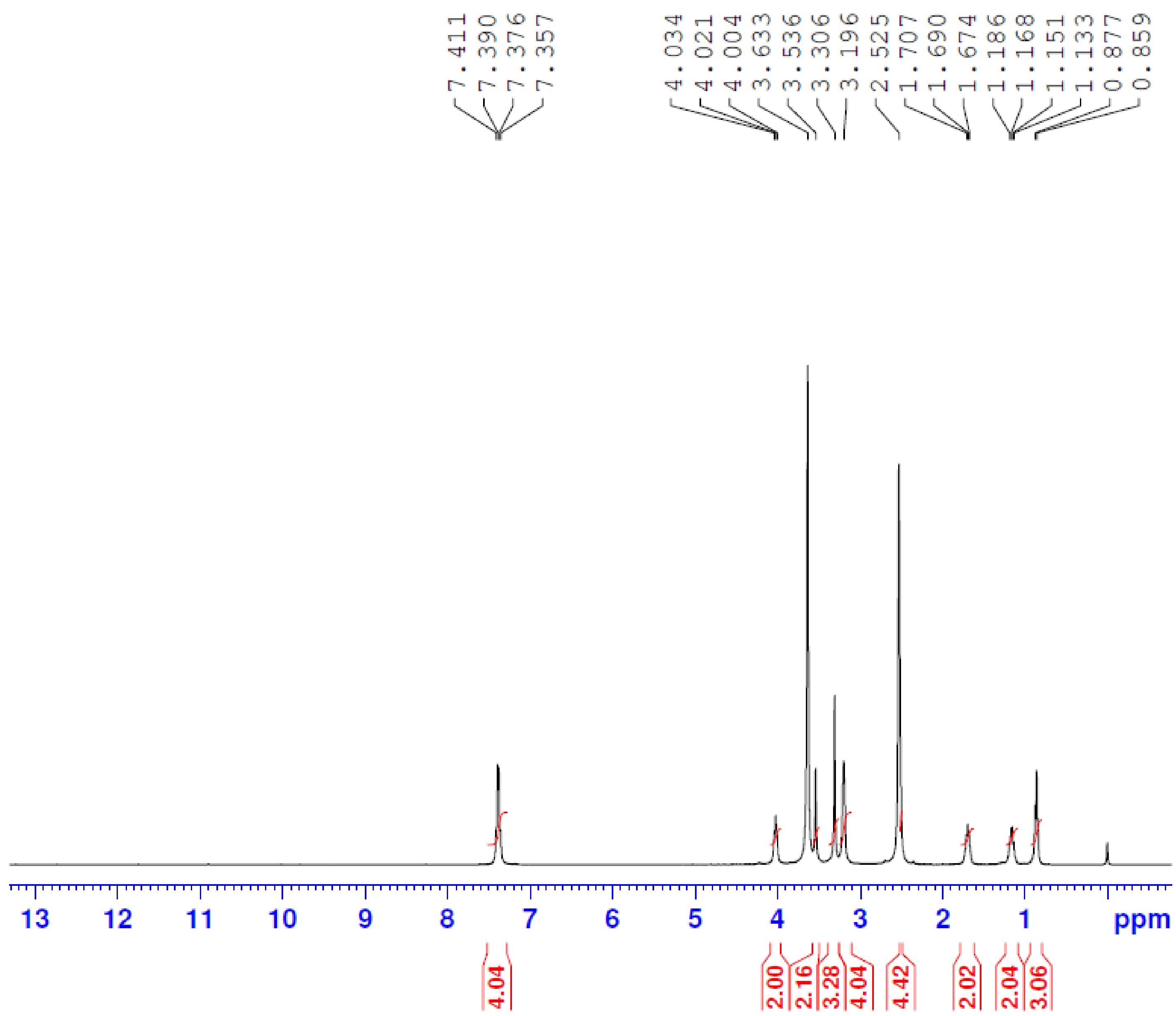

F2 - Acquisition Parameters

Date_	20190711	
Time	12.33	h
INSTRUM	Avance Neo	
PROBHD	Z116098_0787 (	
PULPROG	zg30	
TD	65536	
SOLVENT	DMSO	
NS	8	
DS	0	
SWH	7142.857	Hz
FIDRES	0.217983	Hz
AQ	4.5875201	sec
RG	101	
DW	70.000	usec
DE	14.62	usec
TE	298.1	K
D1	2.00000000	sec
TD0	1	
SFO1	400.4024725	MHz
NUC1	1H	
P0	3.33	usec
P1	10.00	usec
PLW1	19.73600006	W

F2 - Processing parameters

SI		65536	
SF		400.3999918	MHz
WDW		EM	
SSB	0		
LB		0.30	Hz
GB	0		
PC		1.00	

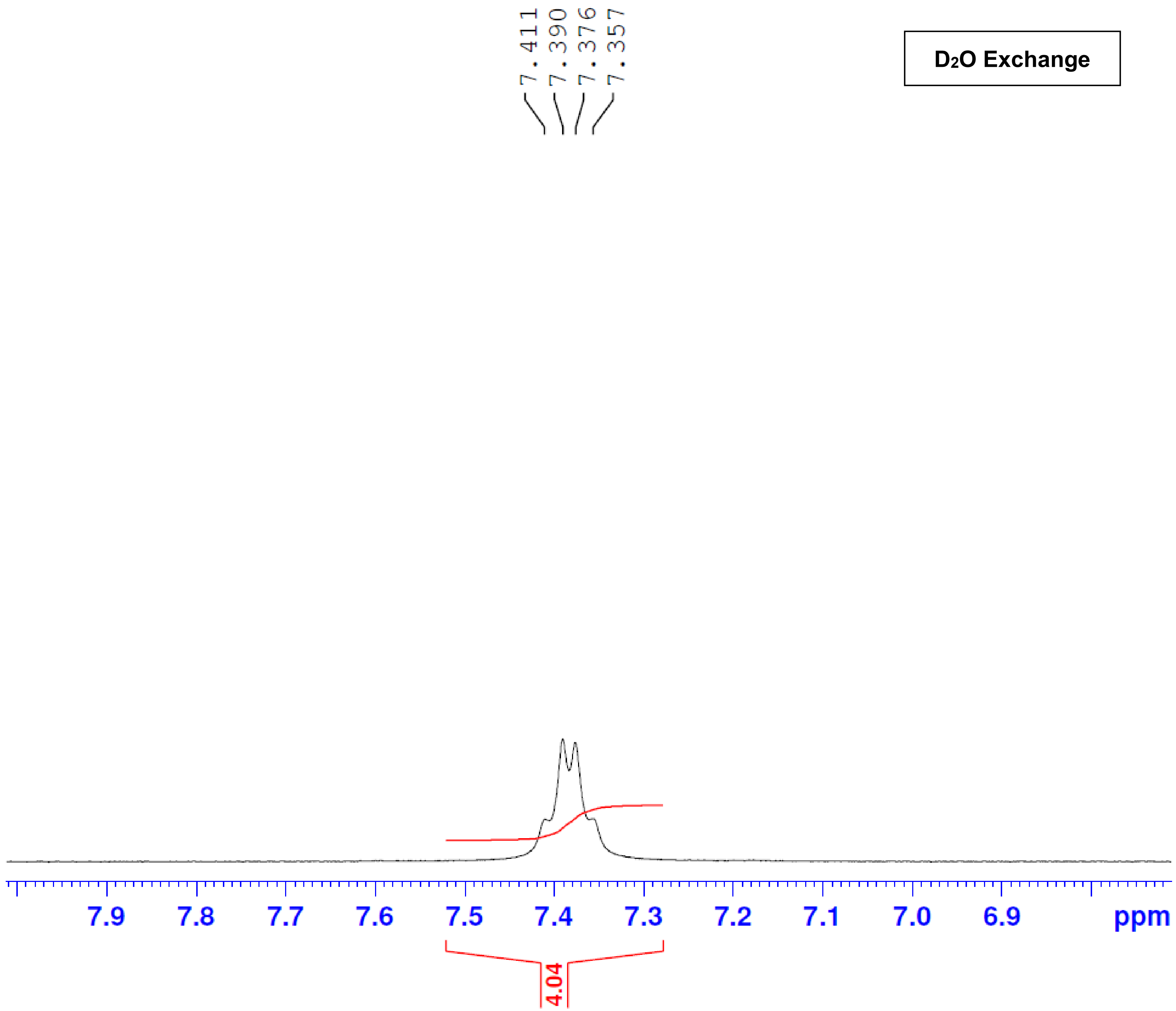

D2O Exchange
7.411
7.390
7.376
7.357
7.9
7.8
7.7
7.6
7.5
7.4
7.3
7.2
7.1
7.0
6.9
ppm
4.04

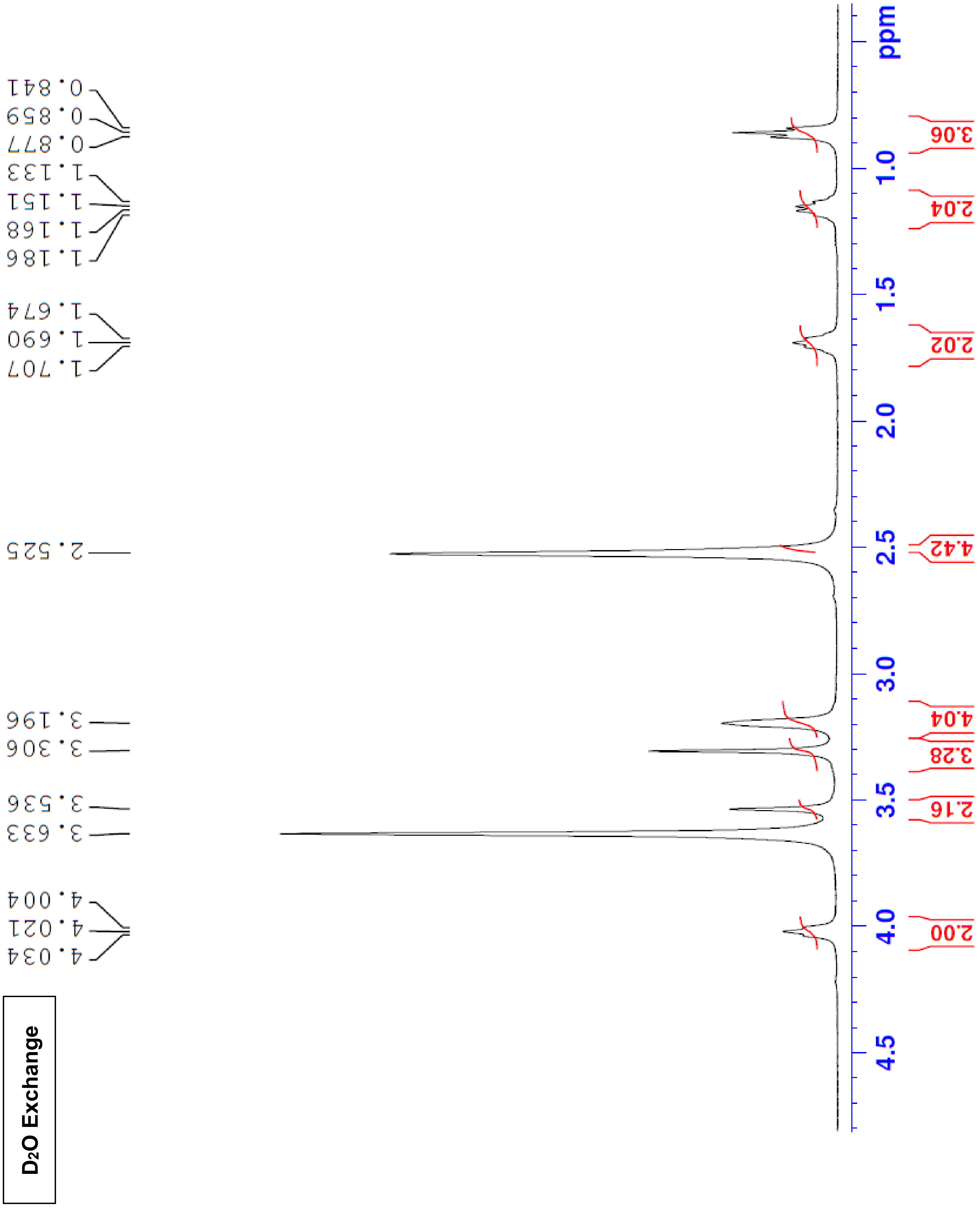
D$_2$O Exchange
0.841
0.859
0.877
1.133
1.151
1.168
1.186
1.674
1.690
1.707
2.525
3.196
3.306
3.536
3.633
4.004
4.021
4.034
ppm
1.0
1.5
2.0
2.5
3.0
3.5
4.0
4.5
3.06
2.04
2.02
4.42
4.04
3.28
2.16
2.00

UPLC Method Conditions :	
Column	: Acquity UPLC BEH C18 (2.1x100) mm, 1.7µm
Mobile Phase-A	: 0.05% TFA in Water
Mobile Phase-B	: 0.05% TFA in Acetonitrile
Gradient (T/% B)	: 0/10,4/90,6/90,6.1/10
Flow Rate	: 0.3 mL/min
Temparature	: 40℃
Diluent	: ACN+Water

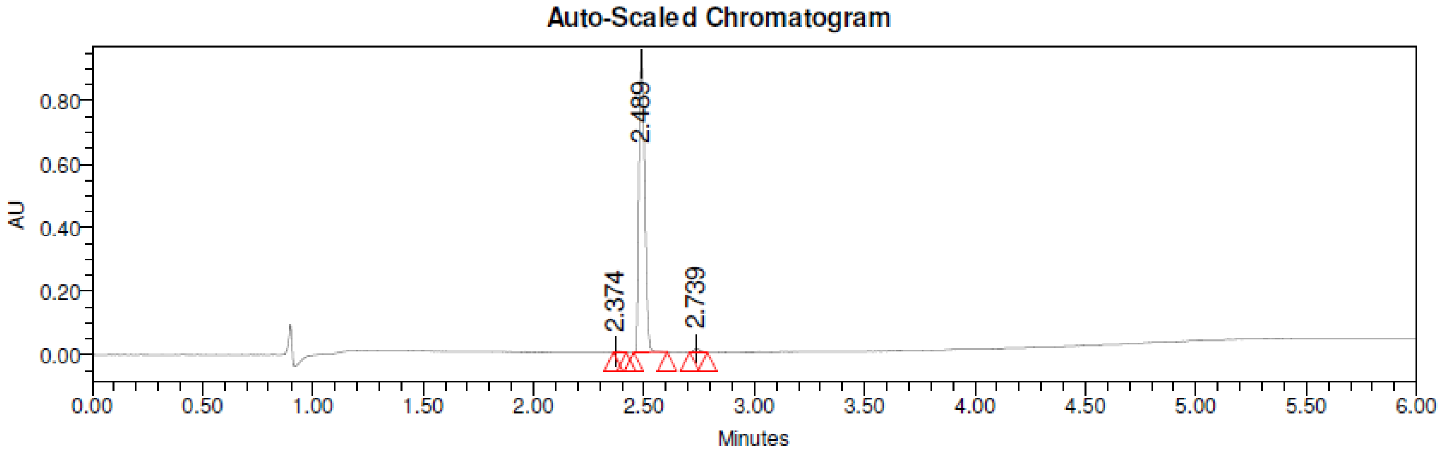

Peak Results

	RT	Area	Height	% Area
1	2.374	3179	1849	0.19
2	2.489	1682611	911715	98.65
3	2.739	19846	11240	1.16

GVK BIO Sciences Private Limited
Discovery Chemistry-Analytical Services

===

Sample ID :X CLUSTER SET-1-TARGET-2(C4536-002-A2) **Date of analysis : 11-Jul-2019/11:13:51**
Acq Method :ATR-1 **Instrument ID: ANL-MCL2-LCMS-001**
1:A,6

021907B3860-X CLUSTER SET-1-TARGET-2(C4536-002-A2)-A Sm (Mn, 7x7)

5: Diode Array
220
Range: 1.641

Time	Height	Area	Area%
1.88	1497412	90158.77	98.29
2.11	54172	1516.33	1.65
2.27	2079	53.24	0.06

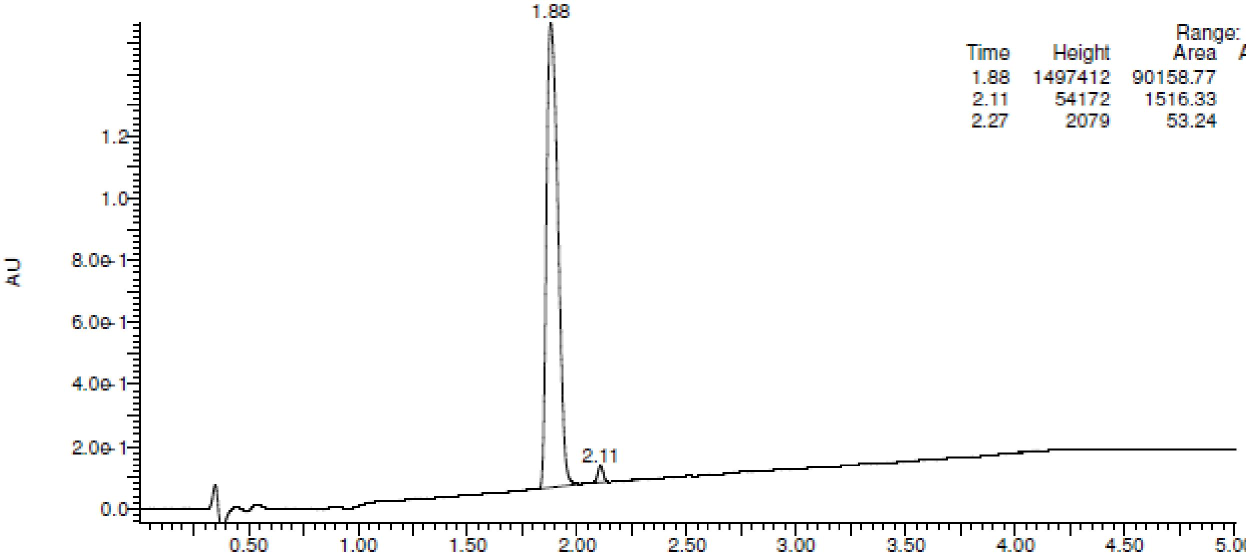

021907B3860-X CLUSTER SET-1-TARGET-2(C4536-002-A2)-A

2: Scan ES+
431.237
7.24e6

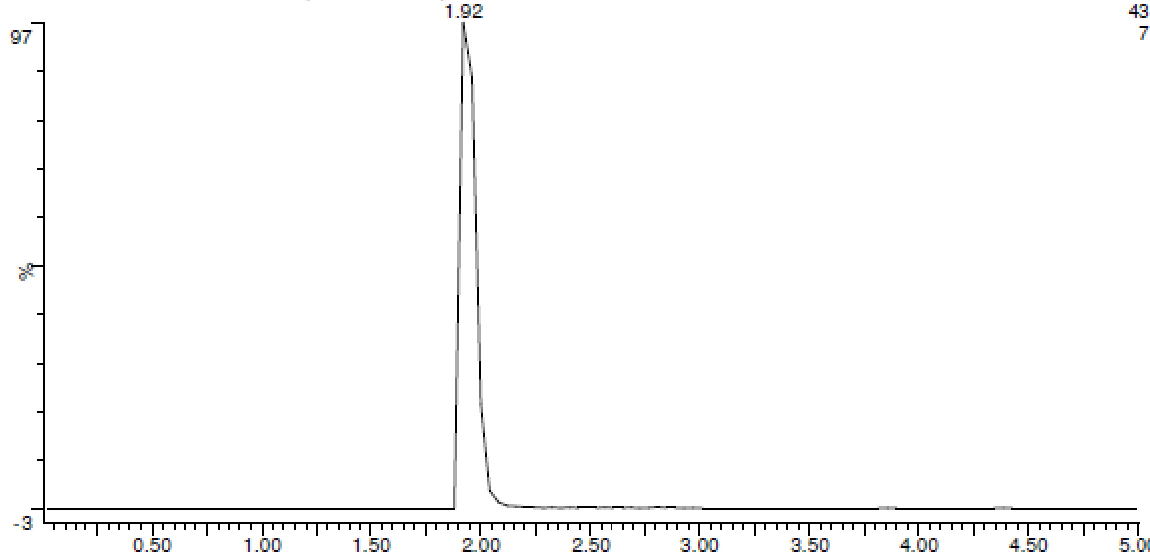

021907B3860-X CLUSTER SET-1-TARGET-2(C4536-002-A2)-A

2: Scan ES+
TIC
1.25e7

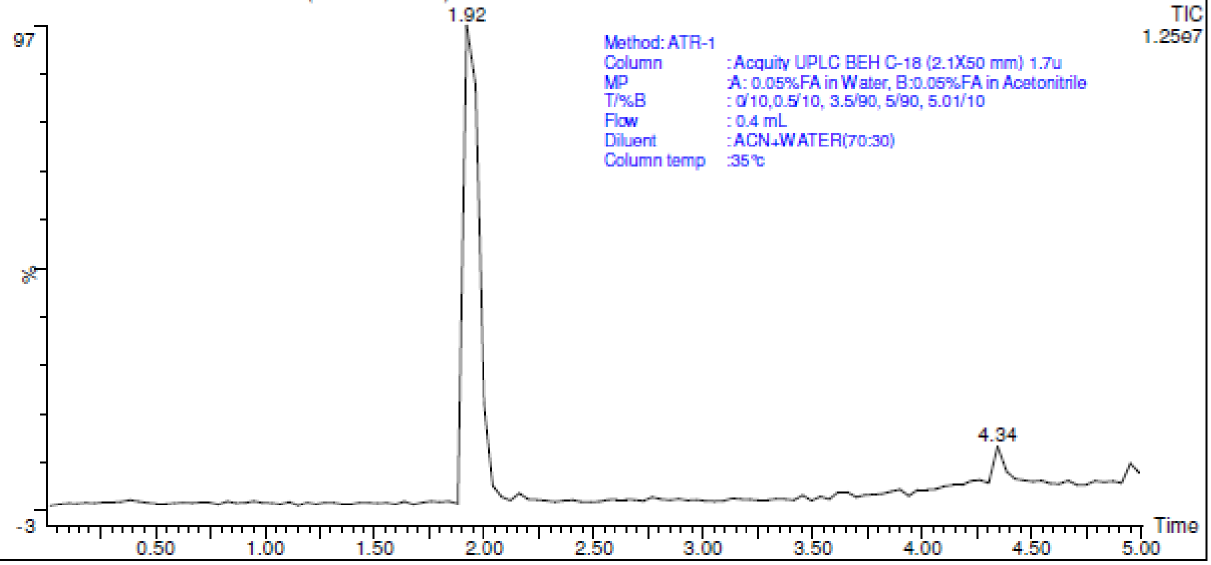

Method: ATR-1
Column : Acquity UPLC BEH C-18 (2.1X50 mm) 1.7u
MP :A: 0.05%FA in Water, B:0.05%FA in Acetonitrile
T/%B : 0/10,0.5/10, 3.5/90, 5/90, 5.01/10
Flow : 0.4 mL
Diluent : ACN+WATER(70:30)
Column temp :35℃

GVK Biosciences Private Limited
Discovery Chemistry-Analytical Services
================================

Sample ID: X CLUSTER SET-1-TARGET-2(C4536-002-A2) **Date of analysis: 11-Jul-2019:11:13:51**
Acq Method :ATR-1 **Instrument ID:ANL-MCL2-LCMS-001**
1:A,6

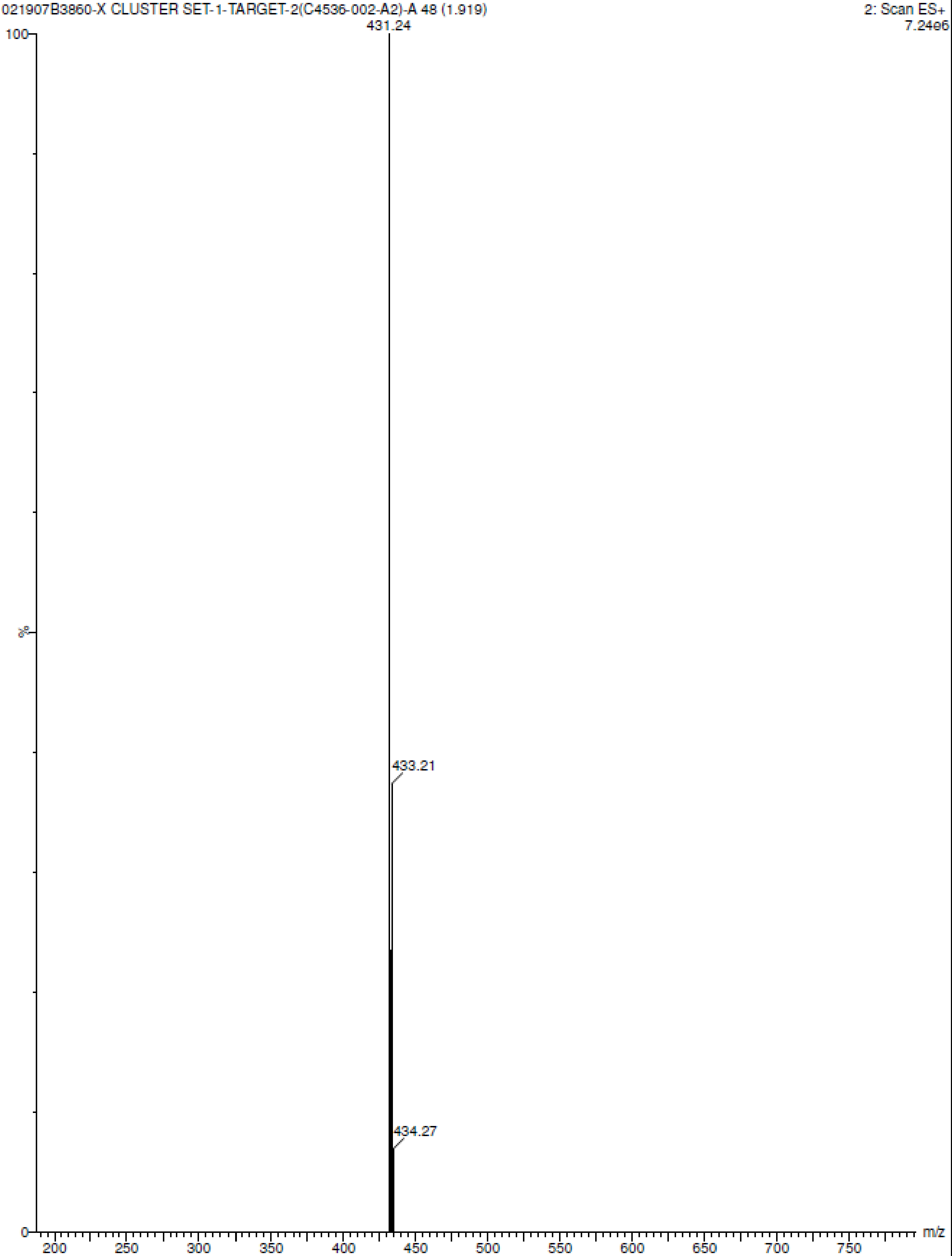

PL_3 (6c)

S.No	Test	Results
1	**Description**	Off white solid
2	**Identification**<br>(a) **NMR**<br>(b) **Mass by LCMS**	Complies to structure<br>411.27 [M+H]+
3	**Chromatographic Purity by UPLC (Area %)**<br>Impurities>1.0%<br>Rt- 2.73 minutes	97.62<br>1.77
4	**Chromatographic Purity by LCMS (Area %)**<br>Impurities>1.0%<br>Rt- 2.11 minutes	97.39<br>2.61

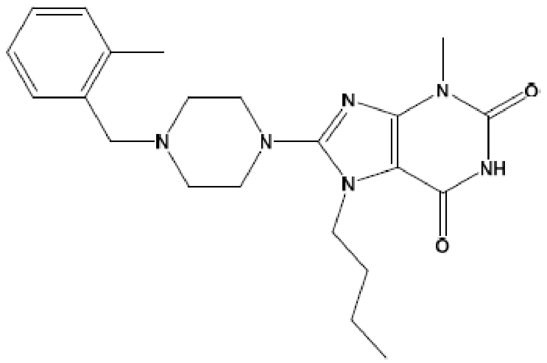

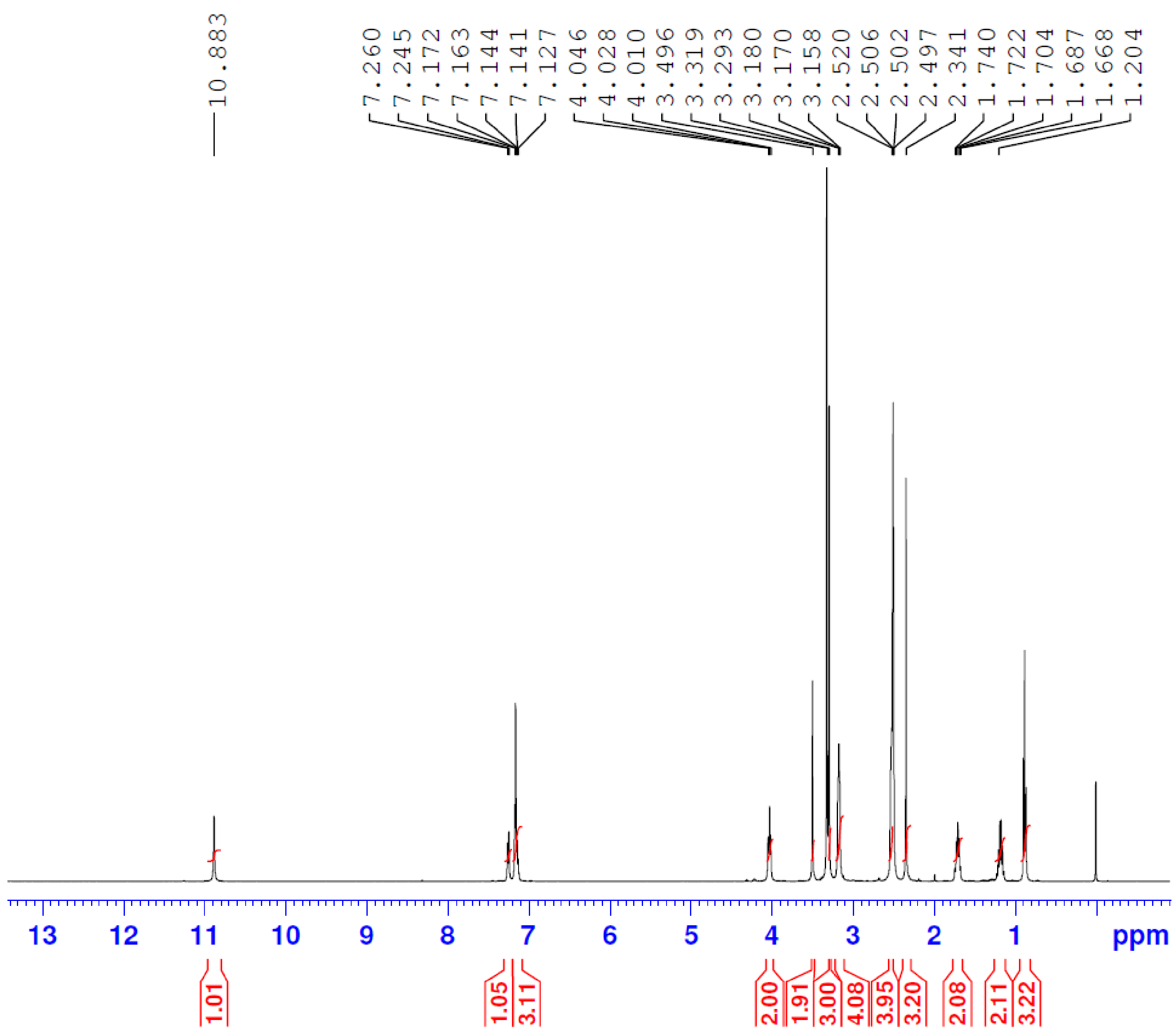

F2 - Acquisition Parameters	
Date_	20190710
Time	23.41 h
INSTRUM	Avance Neo
PROBHD	Z116098_0787 (
PULPROG	zg30
TD	65536
SOLVENT	DMSO
NS	8
DS	0
SWH	7142.857 Hz
FIDRES	0.217983 Hz
AQ	4.5875201 sec
RG	101
DW	70.000 usec
DE	14.62 usec
TE	298.1 K
D1	2.00000000 sec
TD0	1
SFO1	400.4024725 MHz
NUC1	1H
P0	3.33 usec
P1	10.00 usec
PLW1	19.73600006 W
F2 - Processing parameters	
SI	65536
SF	400.4000017 MHz
WDW	EM
SSB	0
LB	0.30 Hz
GB	0
PC	1.00

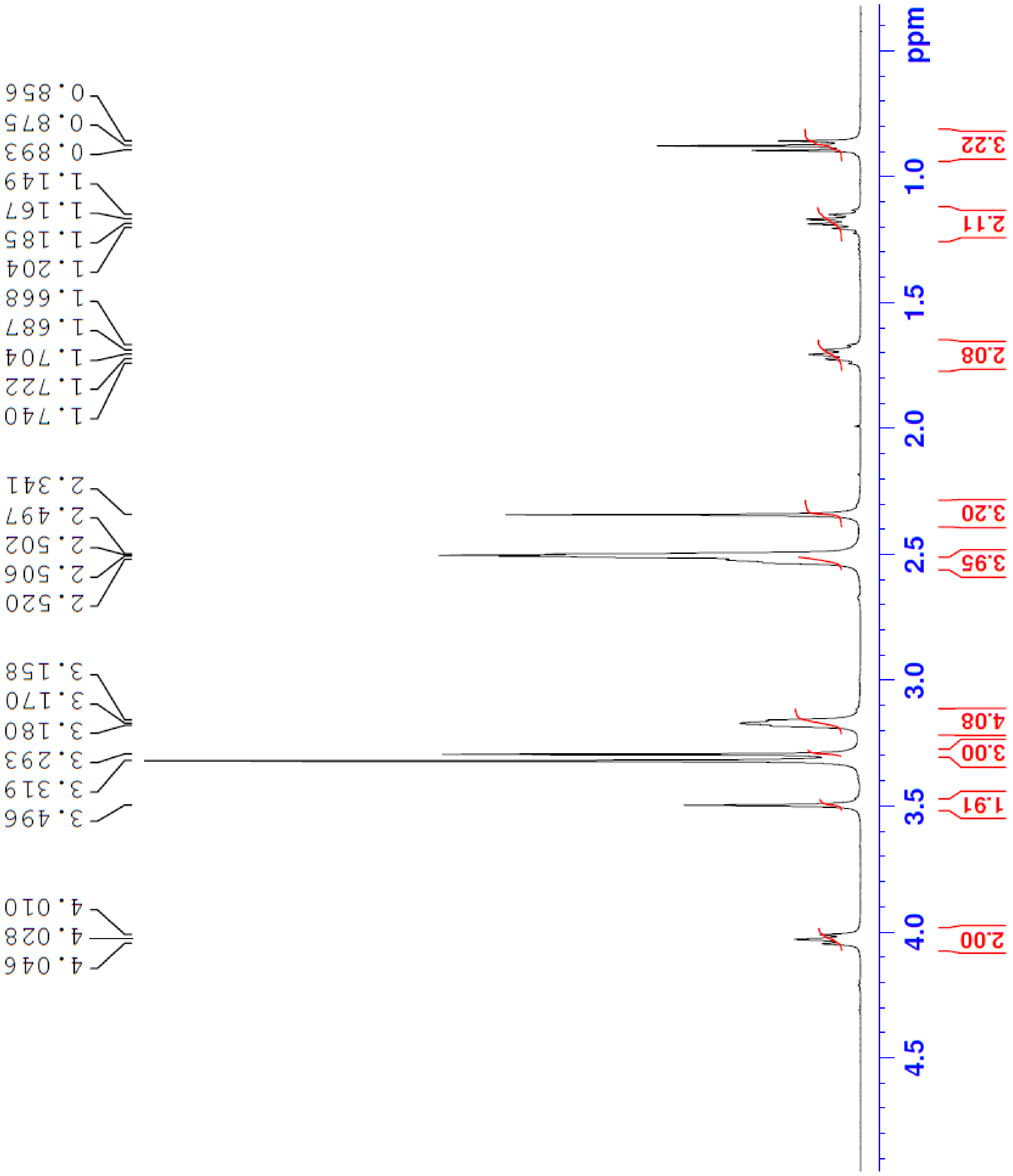
0.856
0.875
0.893
1.149
1.167
1.185
1.204
1.668
1.687
1.704
1.722
1.740
2.341
2.497
2.502
2.506
2.520
3.158
3.170
3.180
3.293
3.319
3.496
4.010
4.028
4.046
ppm
1.0
1.5
2.0
2.5
3.0
3.5
4.0
4.5
3.22
2.11
2.08
3.20
3.95
4.08
3.00
1.91
2.00

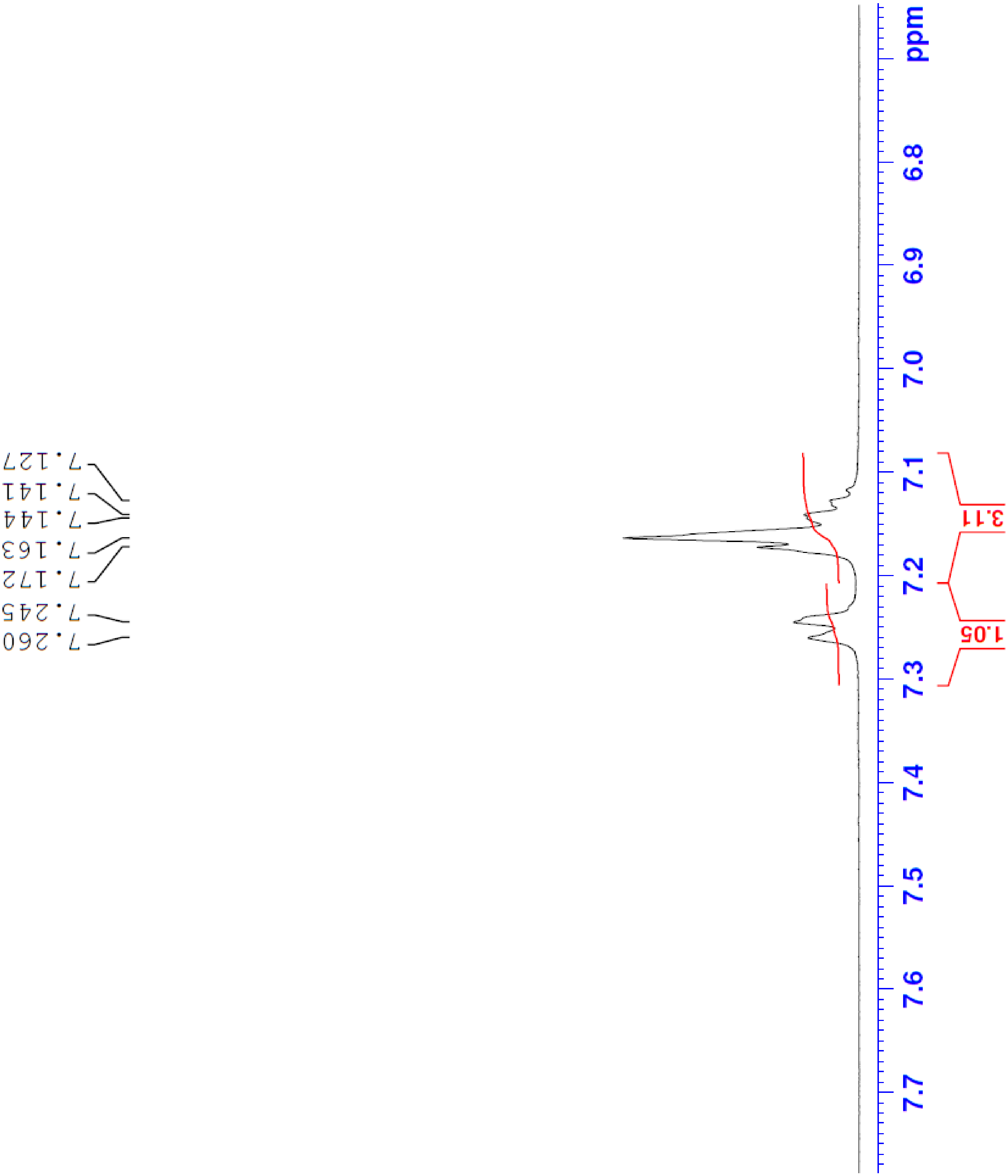

7.127
7.141
7.144
7.163
7.172
7.245
7.260
3.11
1.05
7.7
7.6
7.5
7.4
7.3
7.2
7.1
7.0
6.9
6.8
ppm

D_2O Exchange

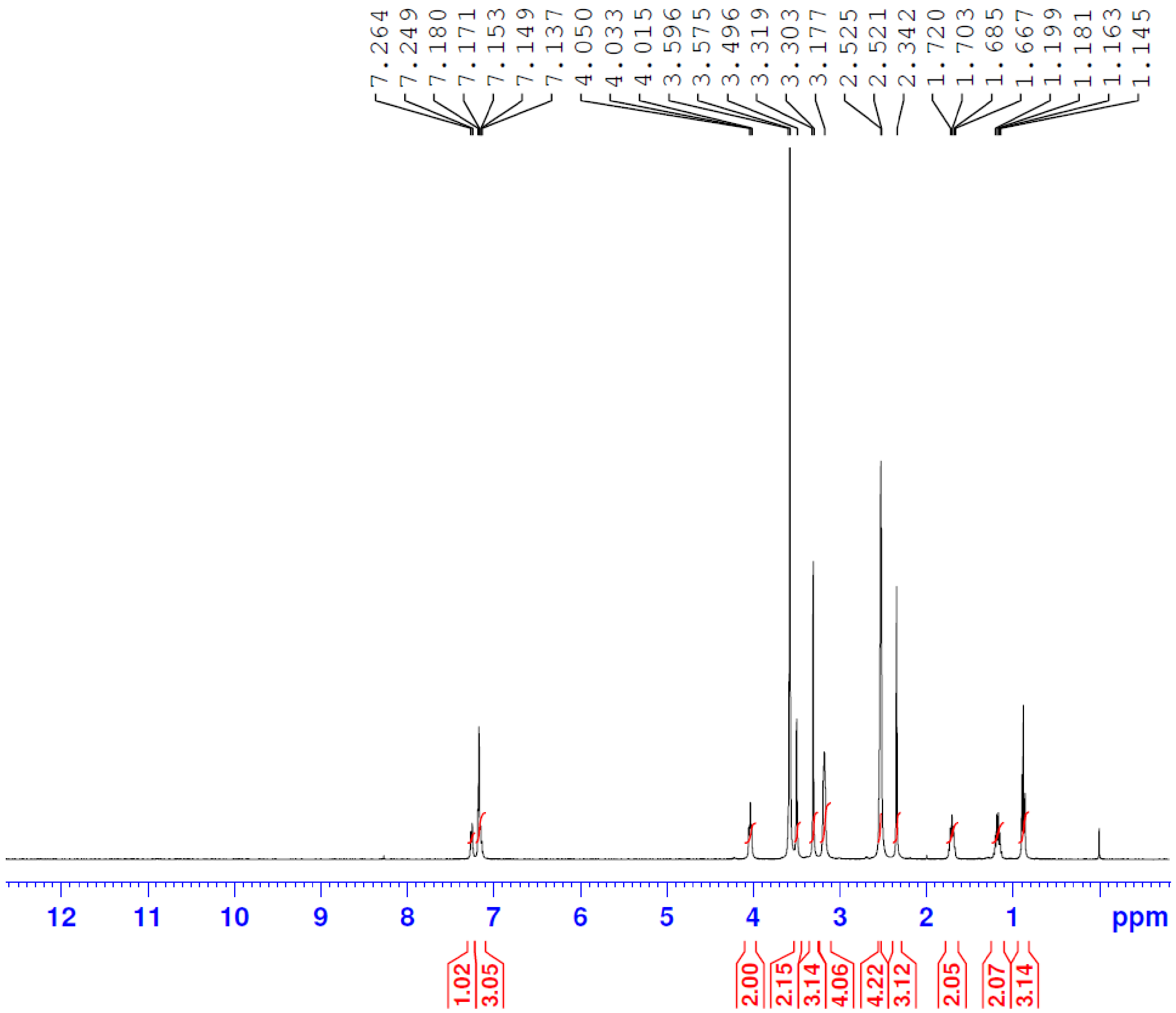


```
F2 - Acquisition Parameters
Date_            20190711
Time                12.30 h
INSTRUM        Avance Neo
PROBHD      Z116098_0787 (
PULPROG              zg30
TD                  65536
SOLVENT              DMSO
NS                      8
DS                      0
SWH              7142.857 Hz
FIDRES           0.217983 Hz
AQ              4.5875201 sec
RG                    101
DW                 70.000 usec
DE                  14.62 usec
TE                  298.1 K
D1             2.00000000 sec
TD0                     1
SFO1          400.4024725 MHz
NUC1                   1H
P0                   3.33 usec
P1                  10.00 usec
PLW1          19.73600006 W

F2 - Processing parameters
SI                  65536
SF            400.3999940 MHz
WDW                    EM
SSB      0
LB                   0.30 Hz
GB       0
PC                   1.00
```

D_2O Exchange

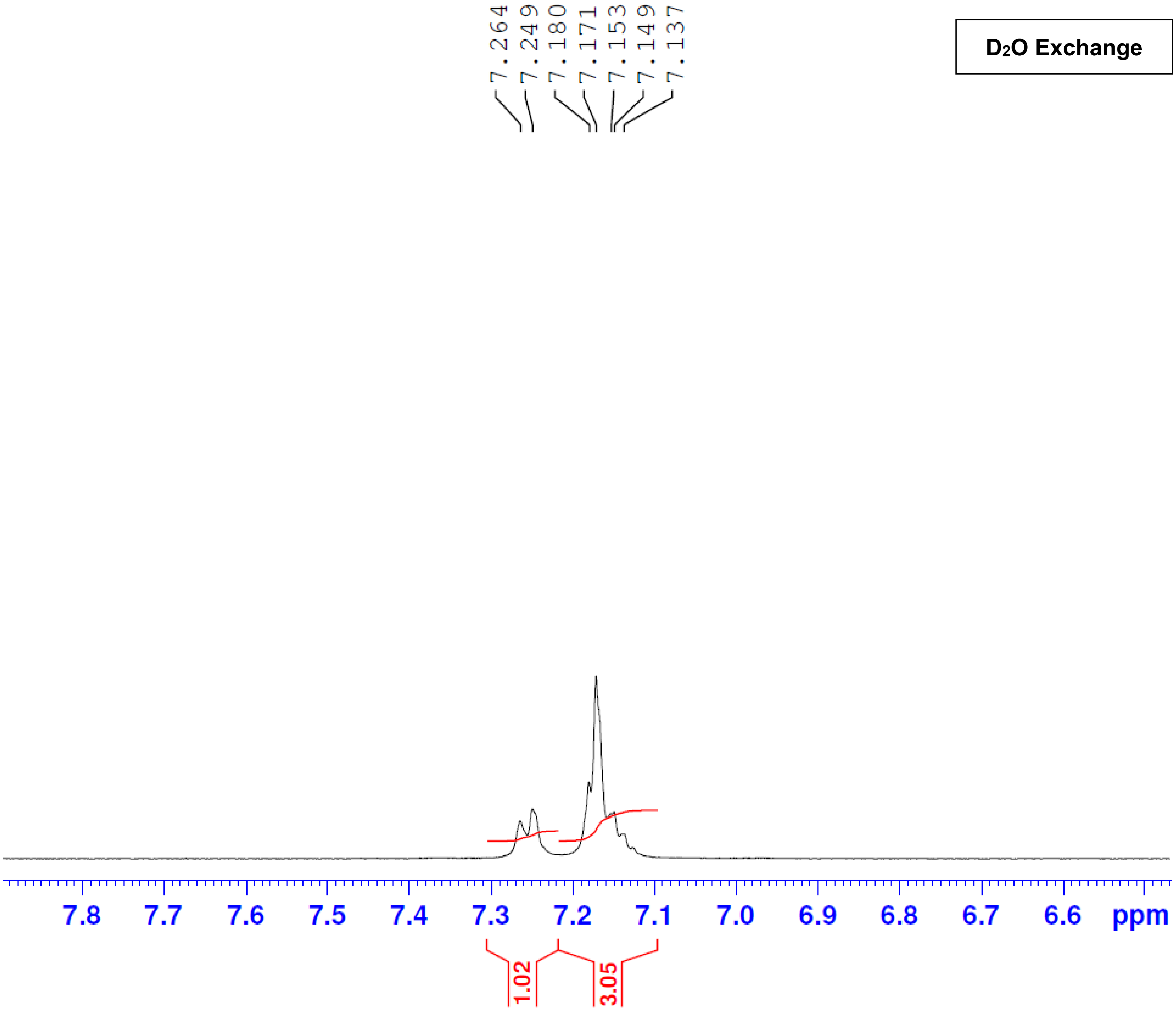

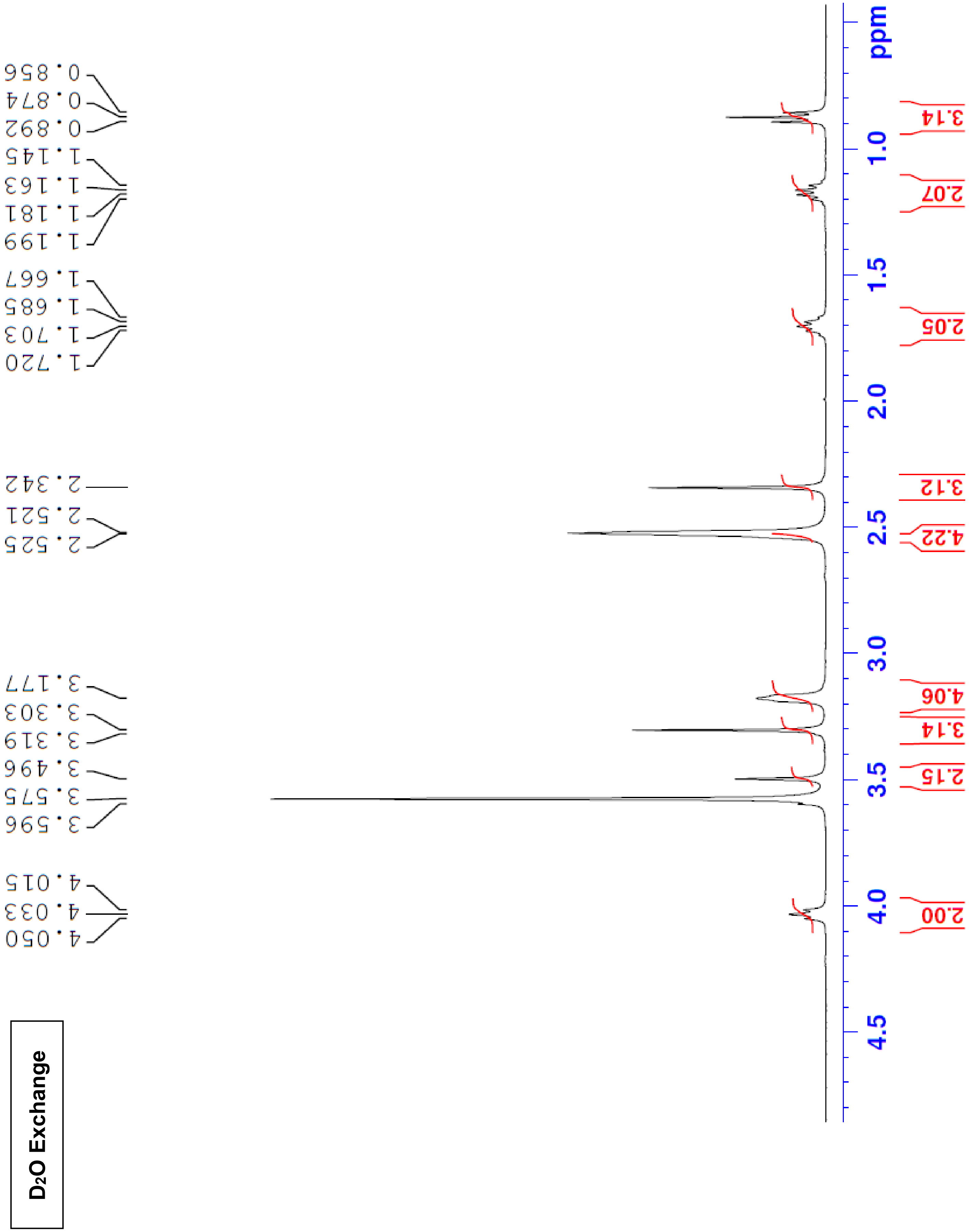
D$_2$O Exchange
0.856
0.874
0.892
1.145
1.163
1.181
1.199
1.667
1.685
1.703
1.720
2.342
2.521
2.525
3.177
3.303
3.319
3.496
3.575
3.596
4.015
4.033
4.050
ppm
1.0
1.5
2.0
2.5
3.0
3.5
4.0
4.5
3.14
2.07
2.05
3.12
4.22
4.06
3.14
2.15
2.00

UPLC Method Conditions :

Column : Acquity UPLC BEH C18 (2.1x100) mm, 1.7µm
Mobile Phase-A : 0.05% TFA in Water
Mobile Phase-B : 0.05% TFA in Acetonitrile
Gradient (T/% B) : 0/10,4/90,6/90,6.1/10
Flow Rate : 0.3 mL/min
Temparature : 40℃
Diluent : ACN+Water

Auto-Scaled Chromatogram

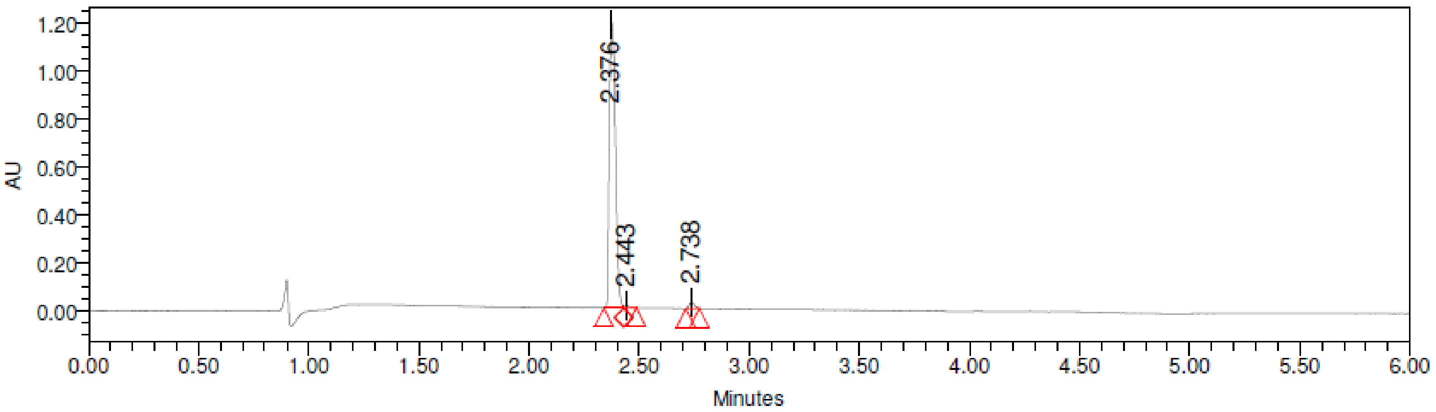

Peak Results

	RT	Area	Height	% Area
1	2.376	2325945	1188502	97.62
2	2.443	14664	8879	0.62
3	2.738	42055	24858	1.77

GVK BIO Sciences Private Limited
Discovery Chemistry-Analytical Services

Sample ID :X CLUSTER SET-1-TARGET-3(C4536-003-A2) **Date of analysis : 11-Jul-2019/11:20:48**
Acq Method :ATR-1 **Instrument ID: ANL-MCL2-LCMS-001**
1:A,7

021907B3730-X CLUSTER SET-1-TARGET-3(C4536-003-A2)-A Sm (Mn, 7x7) 5: Diode Array 214 Range: 1.322

Time	Height	Area	Area%
1.80	1161719	63457.09	97.39
2.11	59747	1697.76	2.61

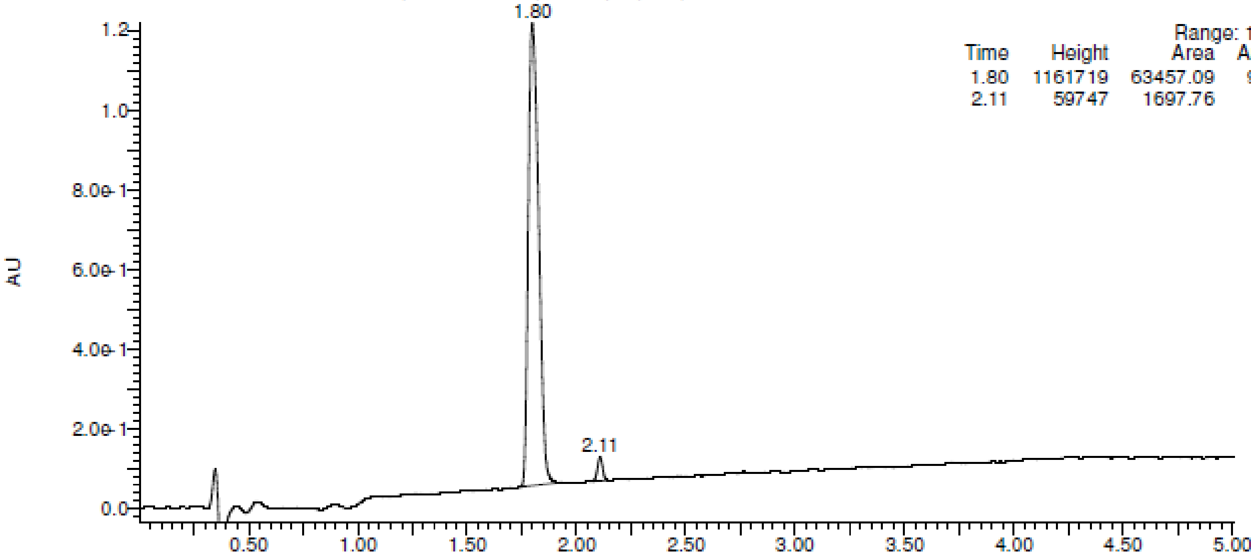

021907B3730-X CLUSTER SET-1-TARGET-3(C4536-003-A2)-A 2: Scan ES+ 411.271 9.44e6

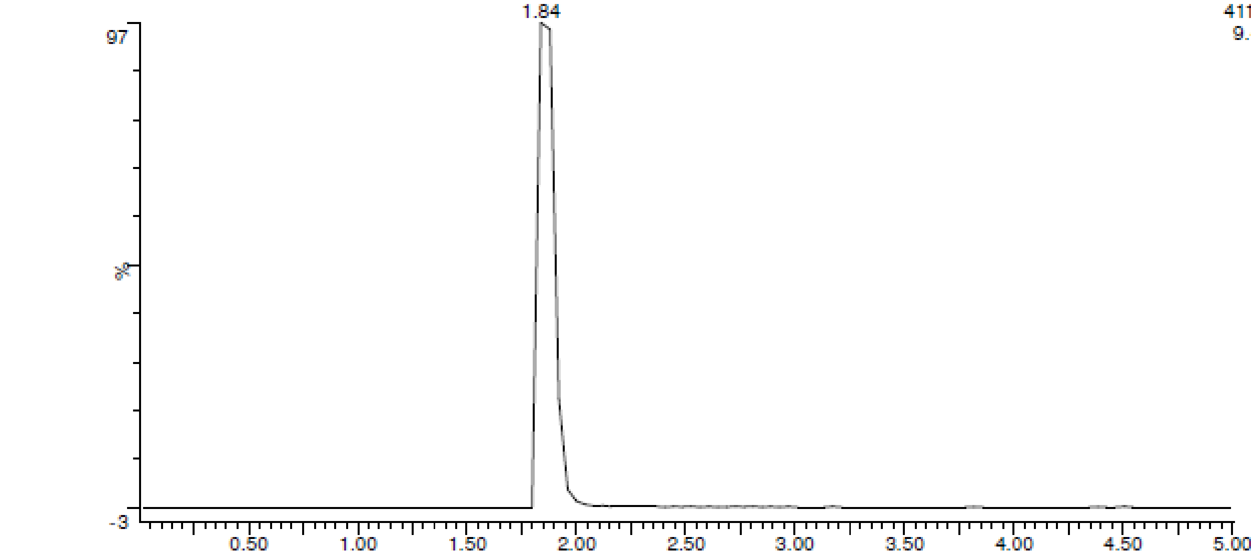

021907B3730-X CLUSTER SET-1-TARGET-3(C4536-003-A2)-A 2: Scan ES+ TIC 1.30e7

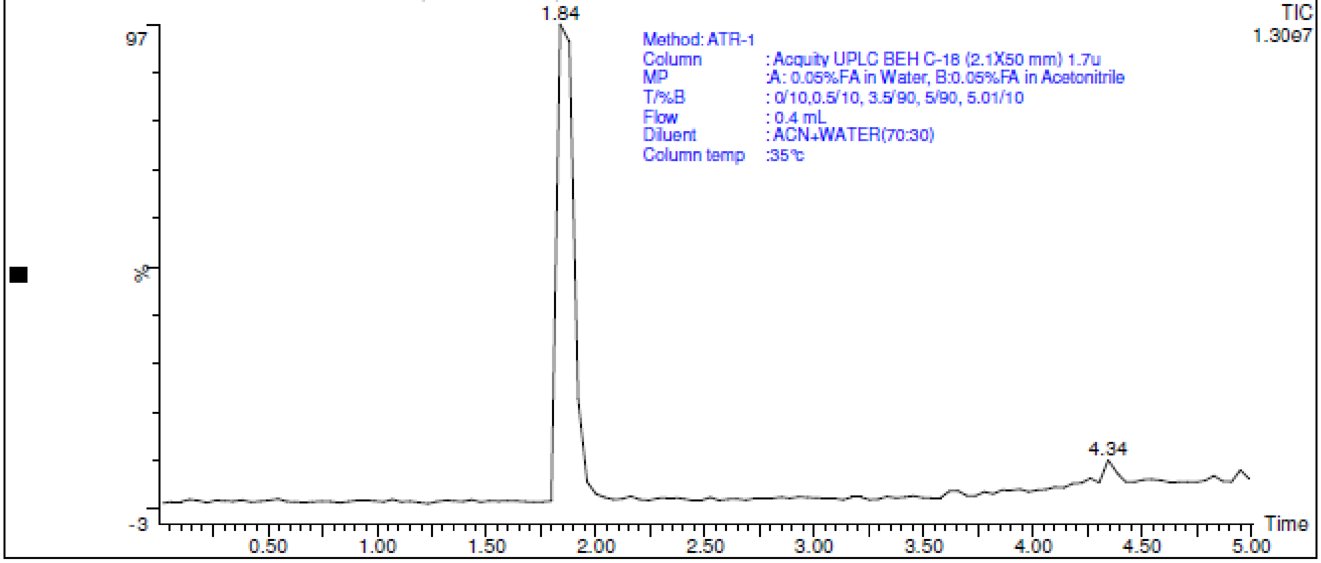

GVK Biosciences Private Limited
Discovery Chemistry-Analytical Services

Sample ID: X CLUSTER SET-1-TARGET-3(C4536-003-A2) **Date of analysis: 11-Jul-2019:11:20:48**
Acq Method :ATR-1 **Instrument ID:ANL-MCL2-LCMS-001**
1:A,7

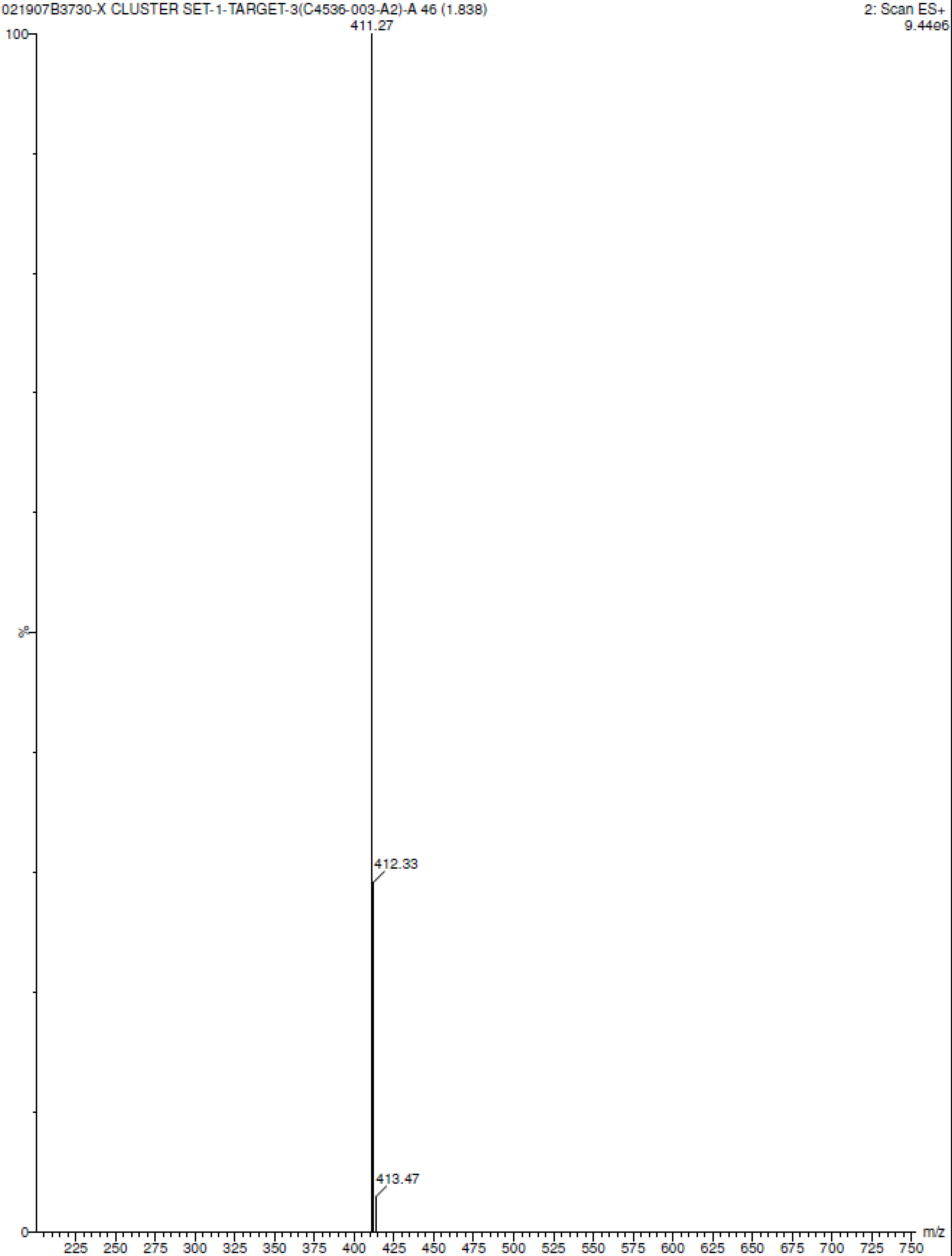

PL_4 (6d)

S.No	Test	Results
1	**Description**	Off-white solid
2	**Identification**<br>(a) **NMR**<br><br>(b) **Mass by LCMS**	 Complies to structure<br><br>456.34 $[M+H]^+$
3	**Chromatographic Purity by UPLC (Area %)**<br>Impurities>1.0%	98.83<br>Nil
4	**Chromatographic Purity by LCMS (Area %)**<br>Impurities>1.0%	99.52<br>Nil

Remarks: 1.Traces of Ethyl Acetate observed in NMR spectrum (Approximately 1.15%).
2. Traces of Aliphatic impurities observed in NMR spectrum.

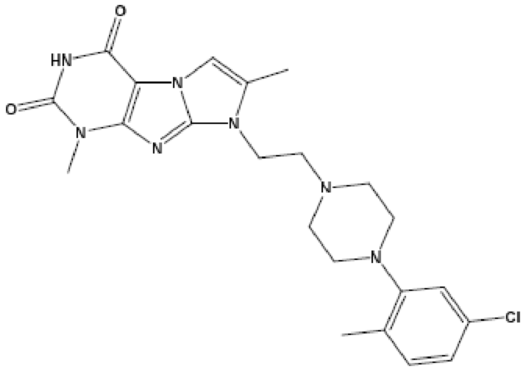

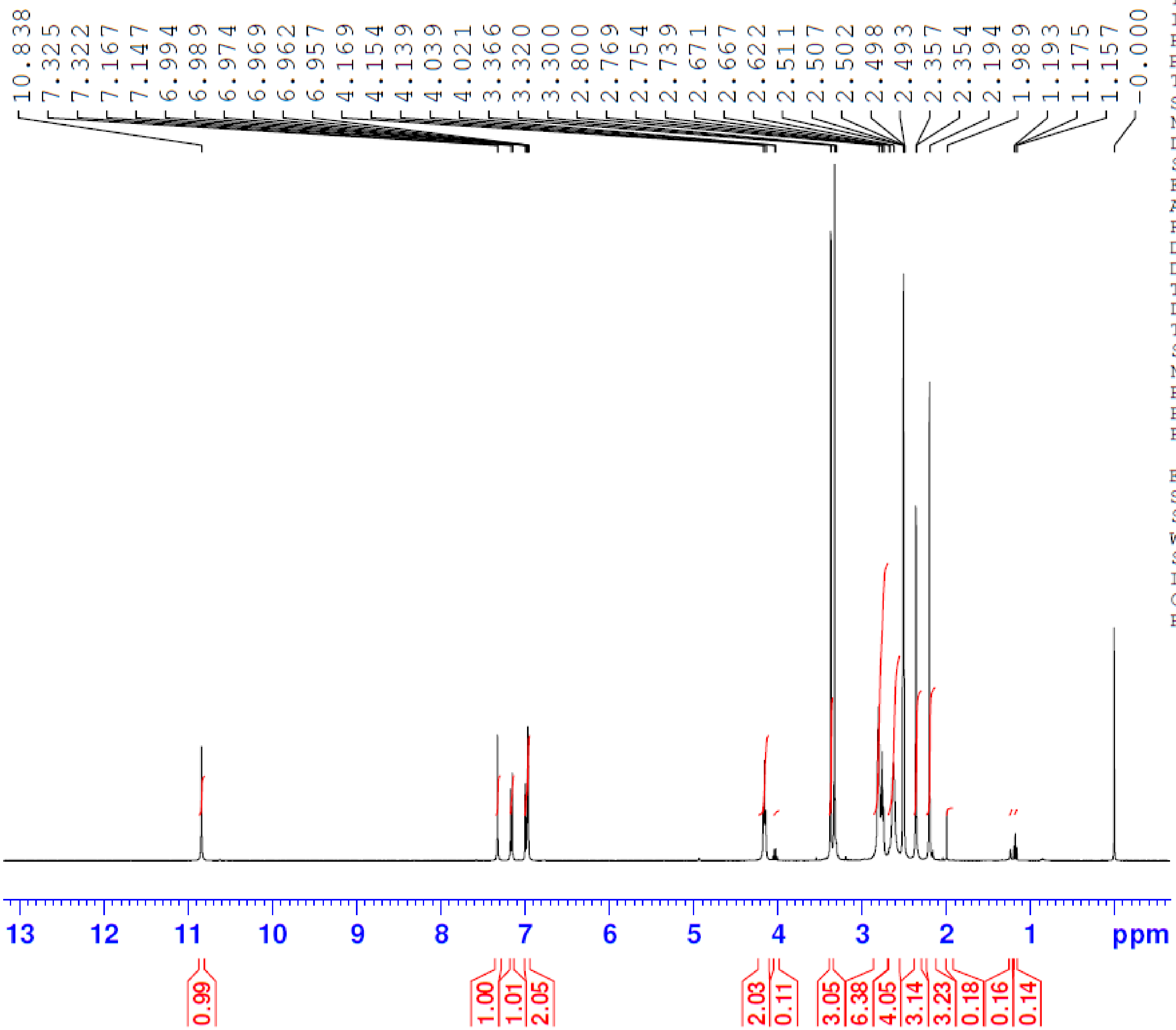


```
F2 - Acquisition Parameters
Date_          20190801
Time              16.01 h
INSTRUM       Avance Neo
PROBHD    Z116098_0787 (
PULPROG            zg30
TD                65536
SOLVENT            DMSO
NS                   32
DS                    0
SWH            7142.857 Hz
FIDRES         0.217983 Hz
AQ            4.5875201 sec
RG                  101
DW               70.000 usec
DE                14.62 usec
TE                298.1 K
D1           2.00000000 sec
TD0                   1
SFO1        400.4024725 MHz
NUC1                 1H
P0                 3.33 usec
P1                10.00 usec
PLW1        19.73600006 W

F2 - Processing parameters
SI                65536
SF          400.4000015 MHz
WDW                  EM
SSB      0
LB                 0.30 Hz
GB       0
PC                 1.00
```

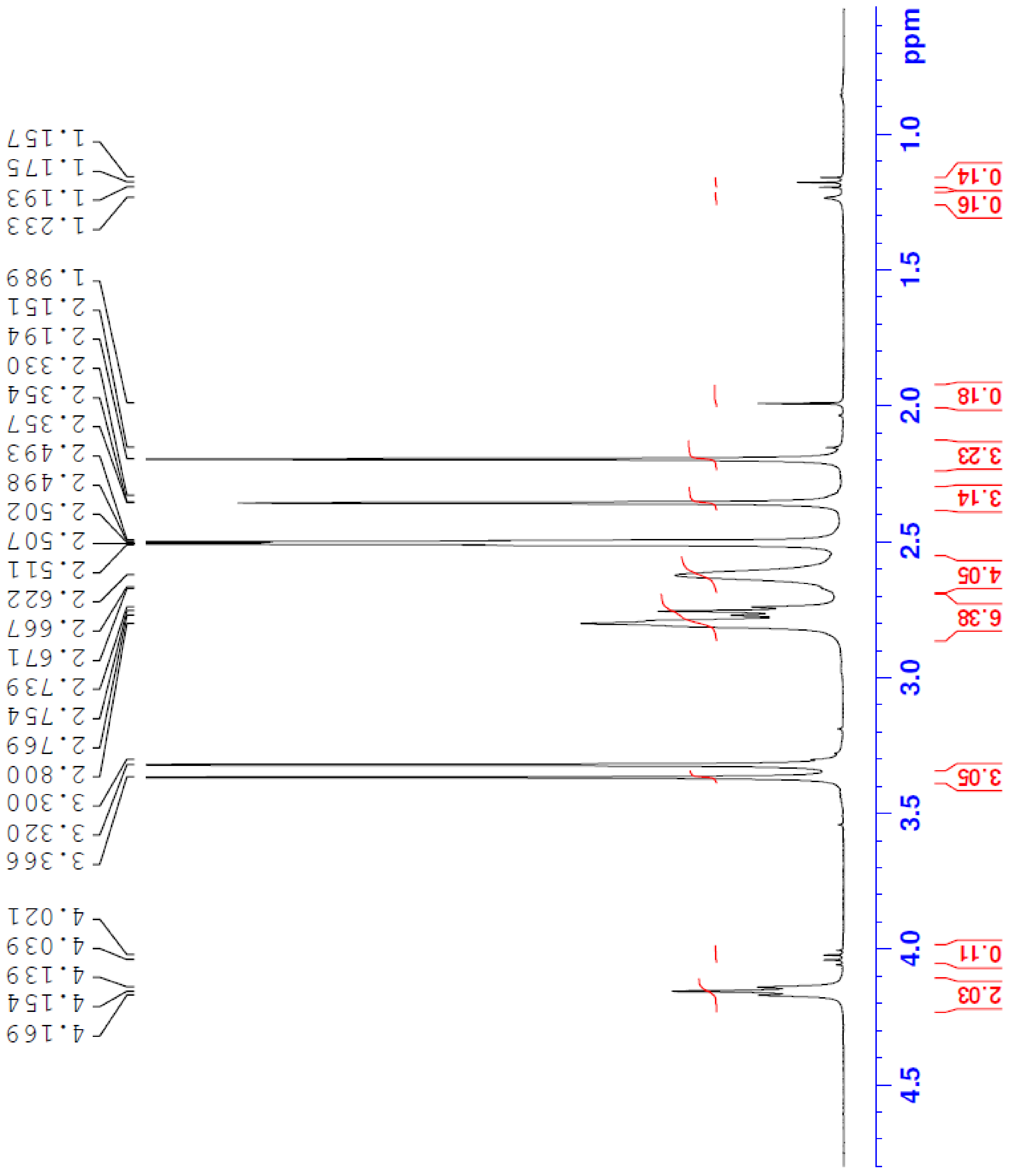
1.157
1.175
1.193
1.233
1.989
2.151
2.194
2.330
2.354
2.357
2.493
2.498
2.502
2.507
2.511
2.622
2.667
2.671
2.739
2.754
2.769
2.800
3.300
3.320
3.366
4.021
4.039
4.139
4.154
4.169
ppm
1.0
1.5
2.0
2.5
3.0
3.5
4.0
4.5
0.14
0.16
0.18
3.23
3.14
4.05
6.38
3.05
0.11
2.03

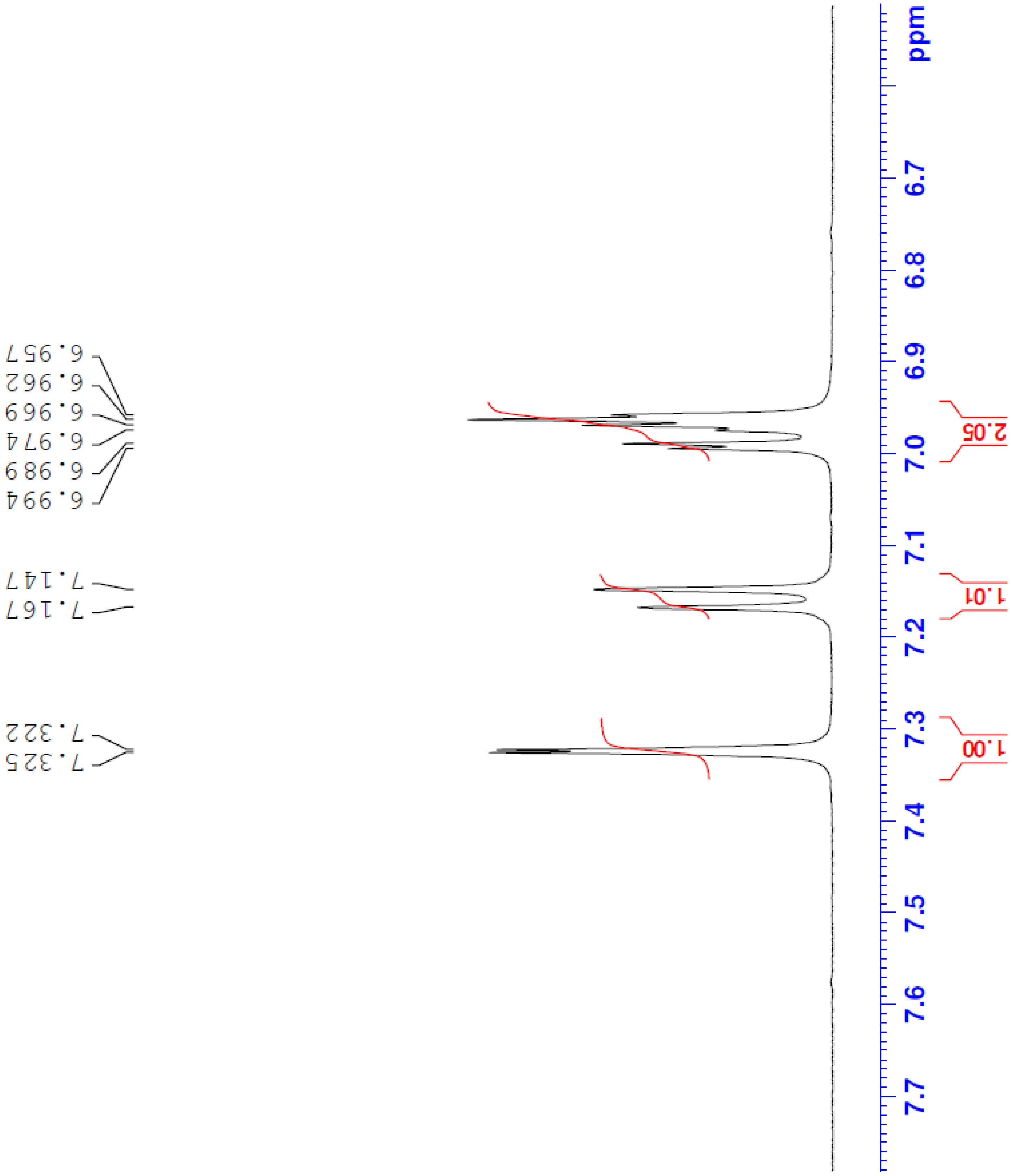
6.957
6.962
6.969
6.974
6.989
6.994
7.147
7.167
7.322
7.325
2.05
1.01
1.00
ppm
6.7
6.8
6.9
7.0
7.1
7.2
7.3
7.4
7.5
7.6
7.7

D_2O Exchange

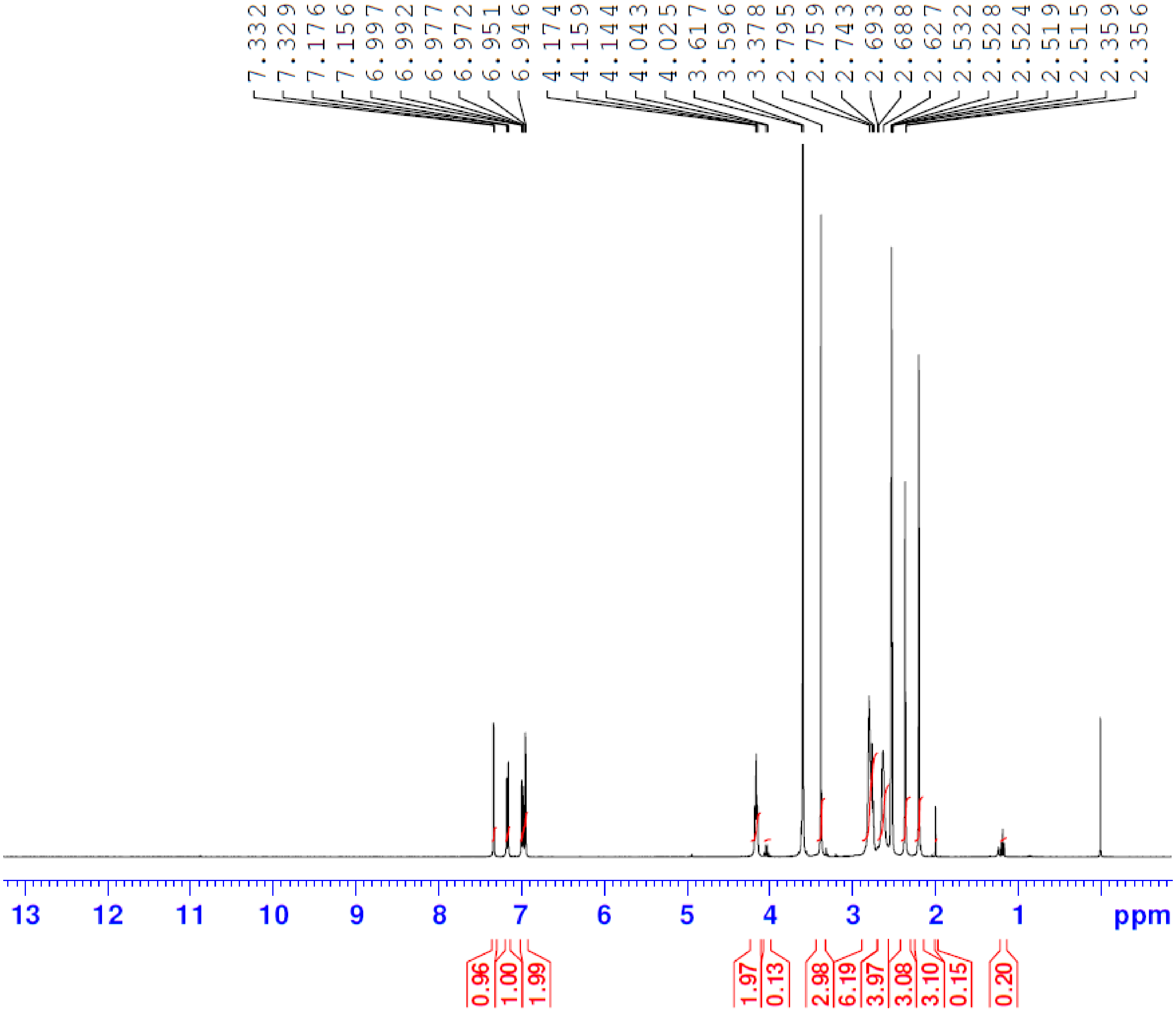


```
F2 - Acquisition Parameters
Date_          20190801
Time              23.30 h
INSTRUM      Avance Neo
PROBHD   Z116098_0787 (
PULPROG            zg30
TD                65536
SOLVENT            DMSO
NS                   32
DS                    0
SWH            7142.857 Hz
FIDRES         0.217983 Hz
AQ            4.5875201 sec
RG                  101
DW               70.000 usec
DE                14.62 usec
TE                298.1 K
D1           2.00000000 sec
TD0                   1
SFO1        400.4024725 MHz
NUC1                 1H
P0                 3.33 usec
P1                10.00 usec
PLW1        19.73600006 W

F2 - Processing parameters
SI                65536
SF          400.3999932 MHz
WDW                  EM
SSB      0
LB                 0.30 Hz
GB       0
PC                 1.00
```

D$_2$O Exchange

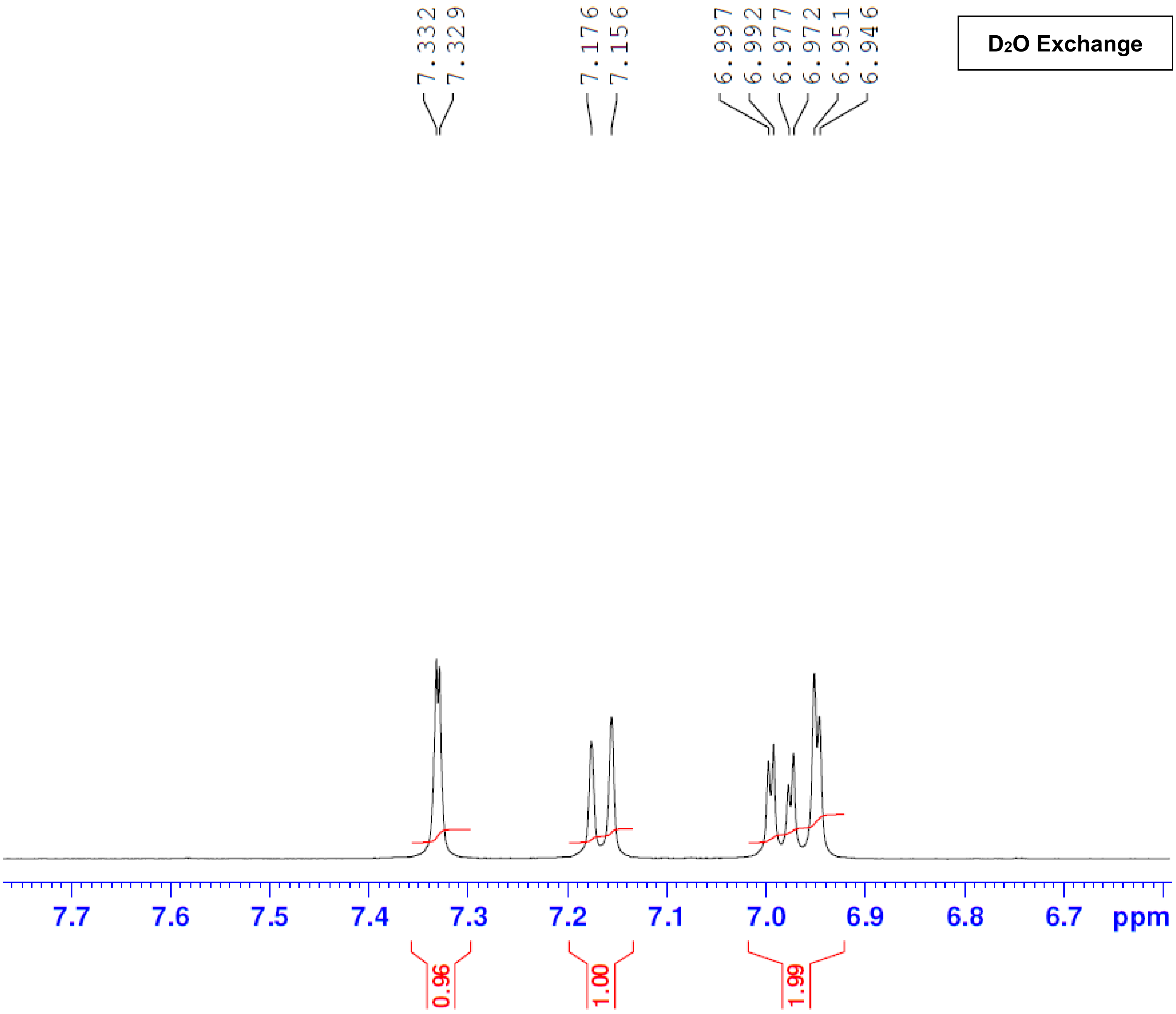

D2O Exchange

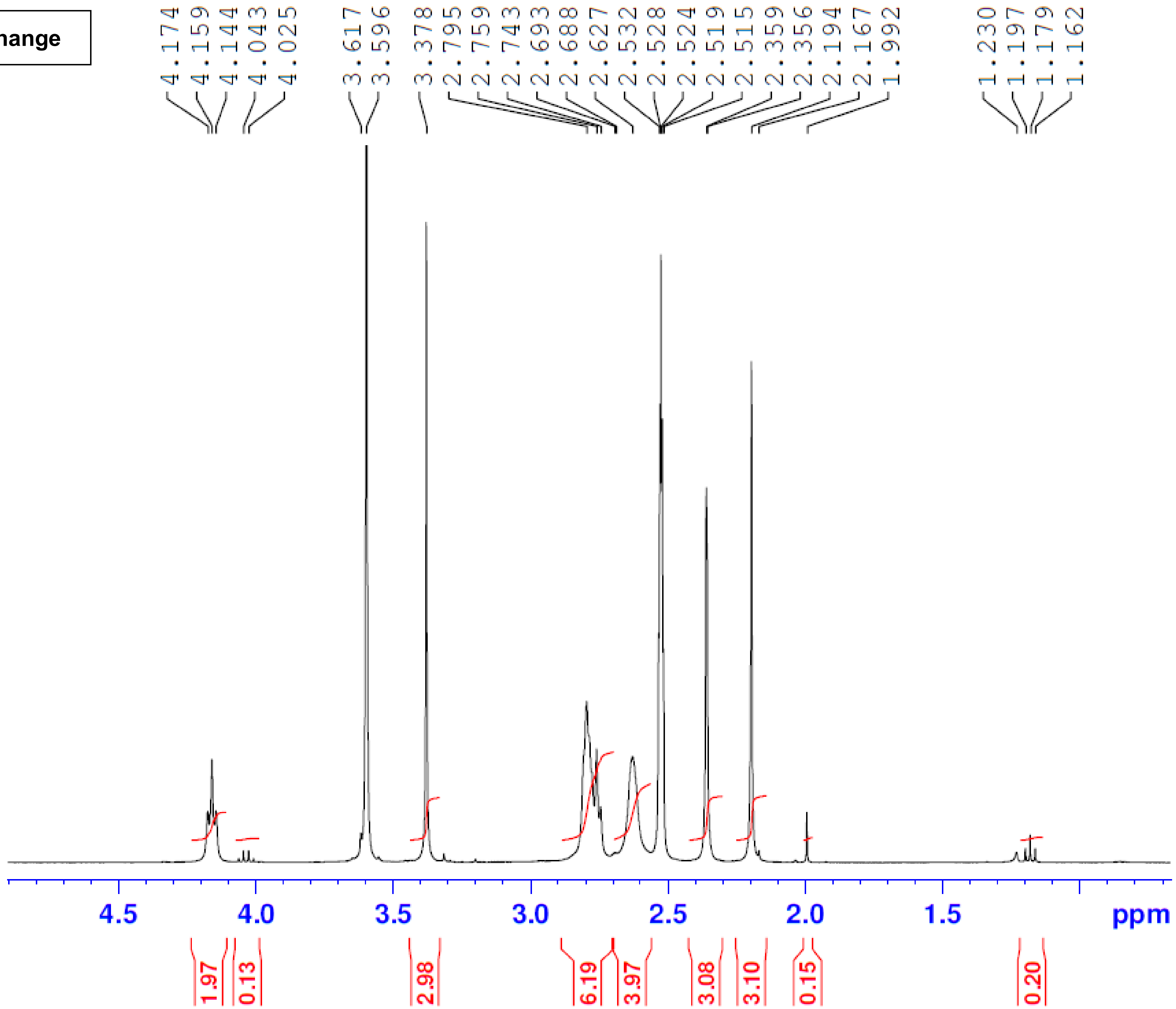

UPLC Method Conditions :

Column : Acquity UPLC BEH C18 (2.1x100) mm, 1.7µm
Mobile Phase-A : 0.05% TFA in Water
Mobile Phase-B : 0.05% TFA in Acetonitrile
Gradient (T/% B) : 0/30,4/90,6/90,6.1/30
Flow Rate : 0.3 mL/min
Temparature : 40℃
Diluent : ACN+Water

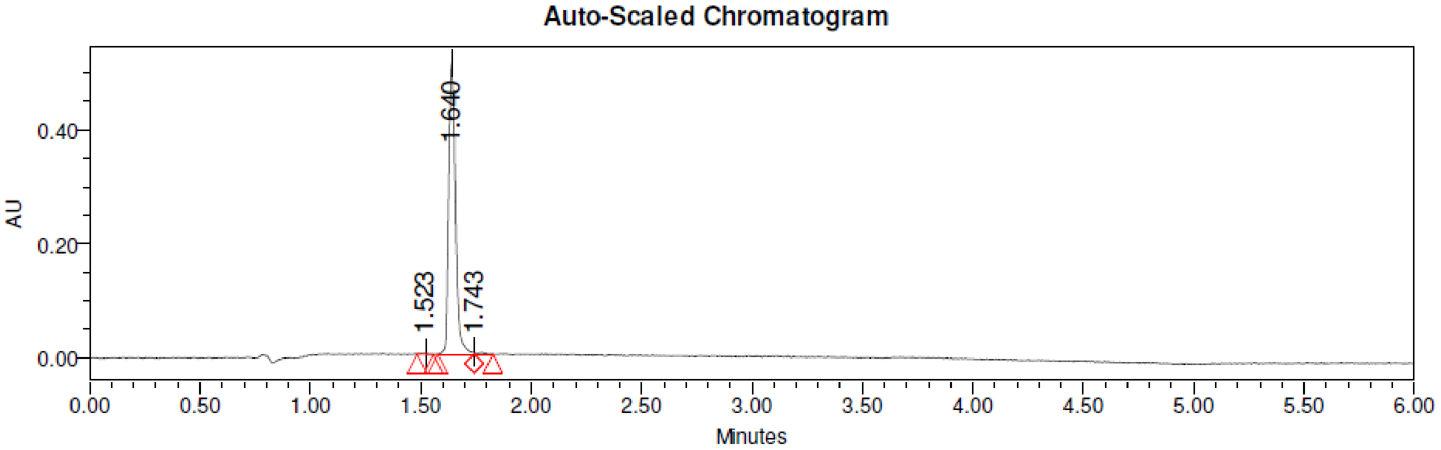

Peak Results

	RT	Area	Height	% Area
1	1.523	5085	2270	0.45
2	1.640	1104796	514156	98.83
3	1.743	7949	3140	0.71

GVK BIO Sciences Private Limited
Discovery Chemistry-Analytical Services
==

Sample ID :X cluster-set-1-Target 6(C4536-020-A1) **Date of analysis : 02-Aug-2019/08:59:35**
Acq Method :ATR-1 **Instrument ID: ANL-MCL2-LCMS-001**
2:B,2

021908A0101-X cluster-set-1-Target 6(C4536-020-A1)-A Sm (Mn, 5x5)

5: Diode Array
214
Range: 1.027

Time	Height	Area	Area%
1.86	2387	60.18	0.14
1.92	939457	42535.93	99.52
2.04	6138	146.51	0.34

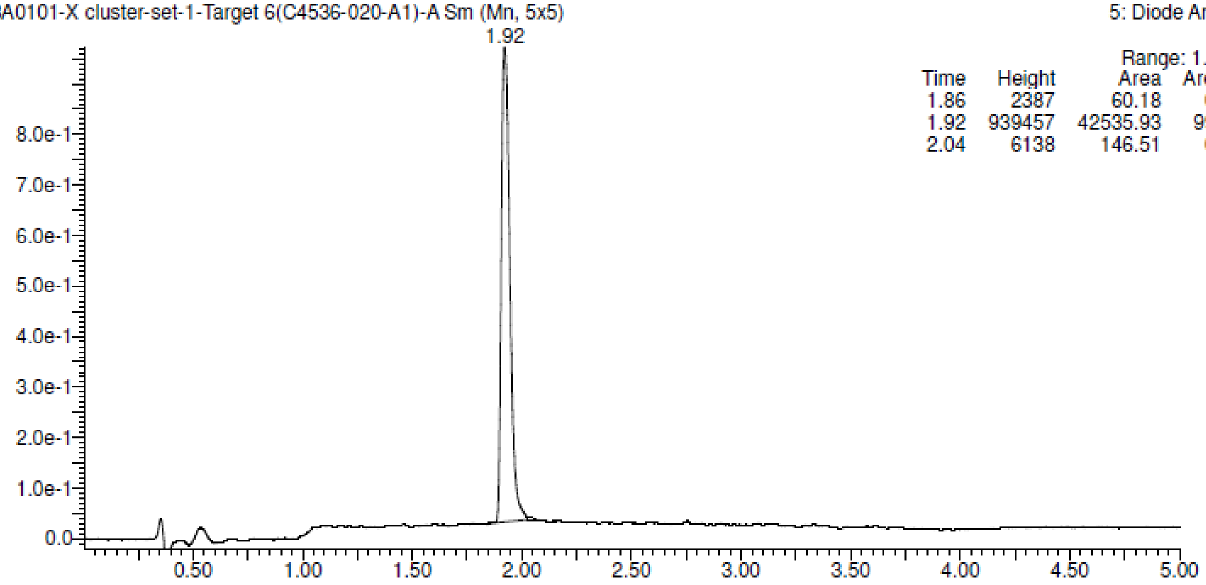

021908A0101-X cluster-set-1-Target 6(C4536-020-A1)-A

1: Scan ES+
456.339
2.81e6

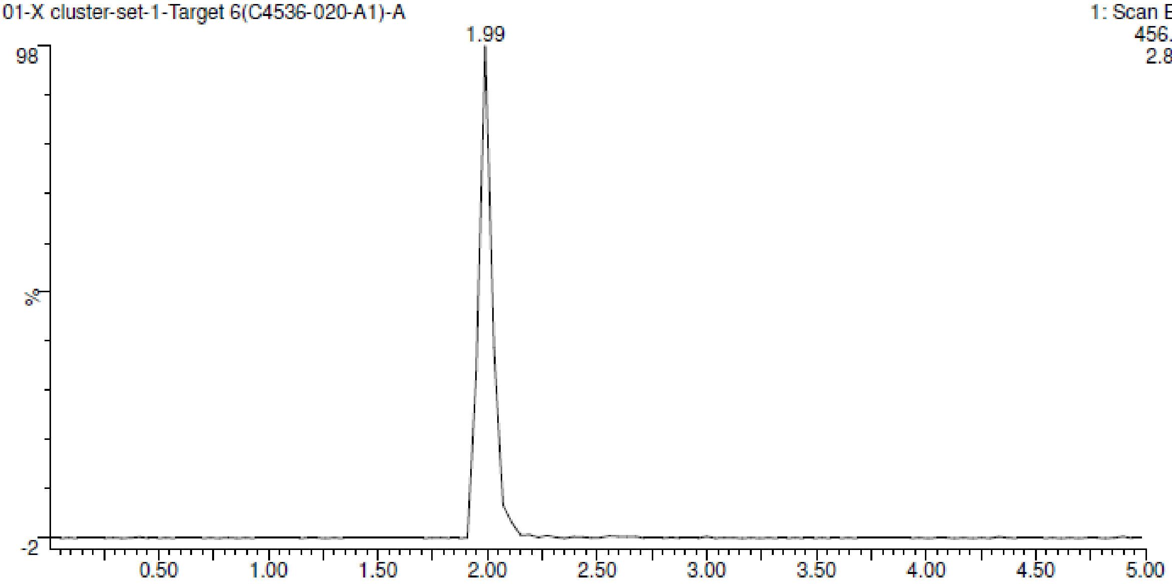

021908A0101-X cluster-set-1-Target 6(C4536-020-A1)-A

1: Scan ES+
TIC
9.11e6

Method: ATR-1
Column : Acquity UPLC BEH C-18 (2.1X50 mm) 1.7u
MP :A: 0.05%FA in Water, B:0.05%FA in Acetonitrile
T/%B : 0/10,0.5/10, 3.5/90, 5/90, 5.01/10
Flow : 0.4 mL
Diluent : ACN+WATER(70:30)
Column temp :35℃

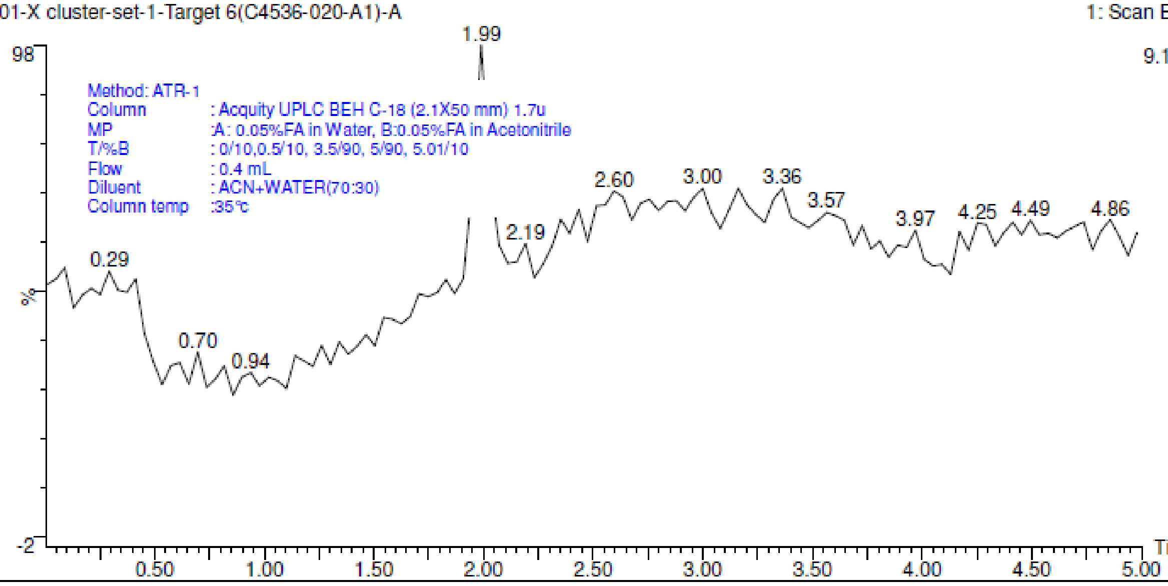

GVK Biosciences Private Limited
Discovery Chemistry-Analytical Services
================================

Sample ID: X cluster-set-1-Target 6(C4536-020-A1) Date of analysis: 02-Aug-2019:08:59:35
Acq Method :ATR-1 Instrument ID:ANL-MCL2-LCMS-001
2:B,2

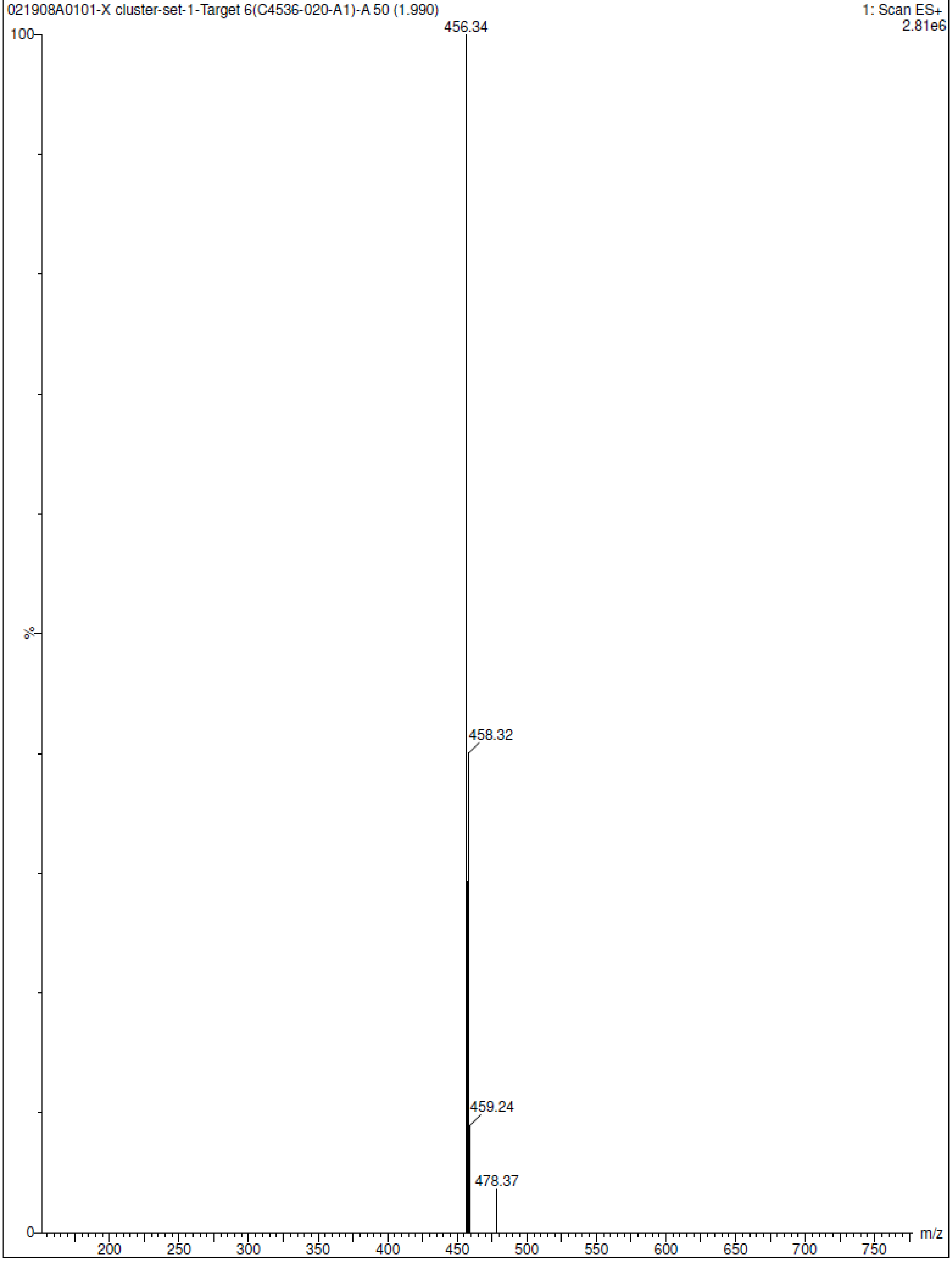

PL_5 (6e)

S.No	Test	Results
1	**Description**	Pale Brown solid
2	**Identification**<br>(a) **NMR**<br>(b) **Mass by LCMS**	 Complies to structure<br>417.50 [M+H]$^+$
3	**Chromatographic Purity by UPLC (Area %)**<br>Impurities>1.0%	99.58<br>Nil
4	**Chromatographic Purity by LCMS (Area %)**<br>Impurities>1.0%	99.29<br>Nil

Remarks:

1. Traces of Ethyl Acetate observed in NMR spectrum (Approximately 0.42%).

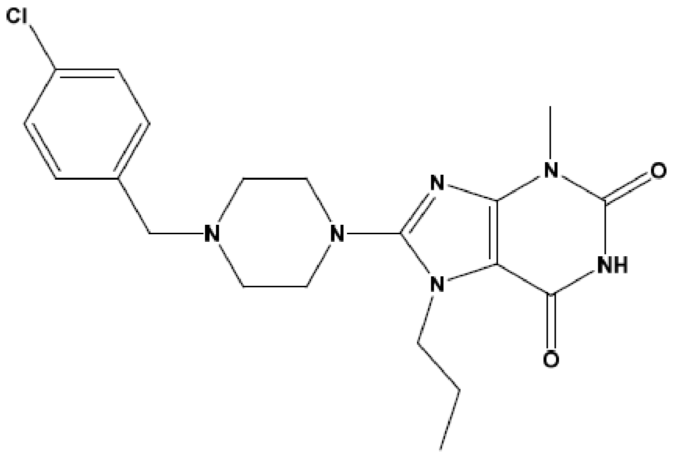

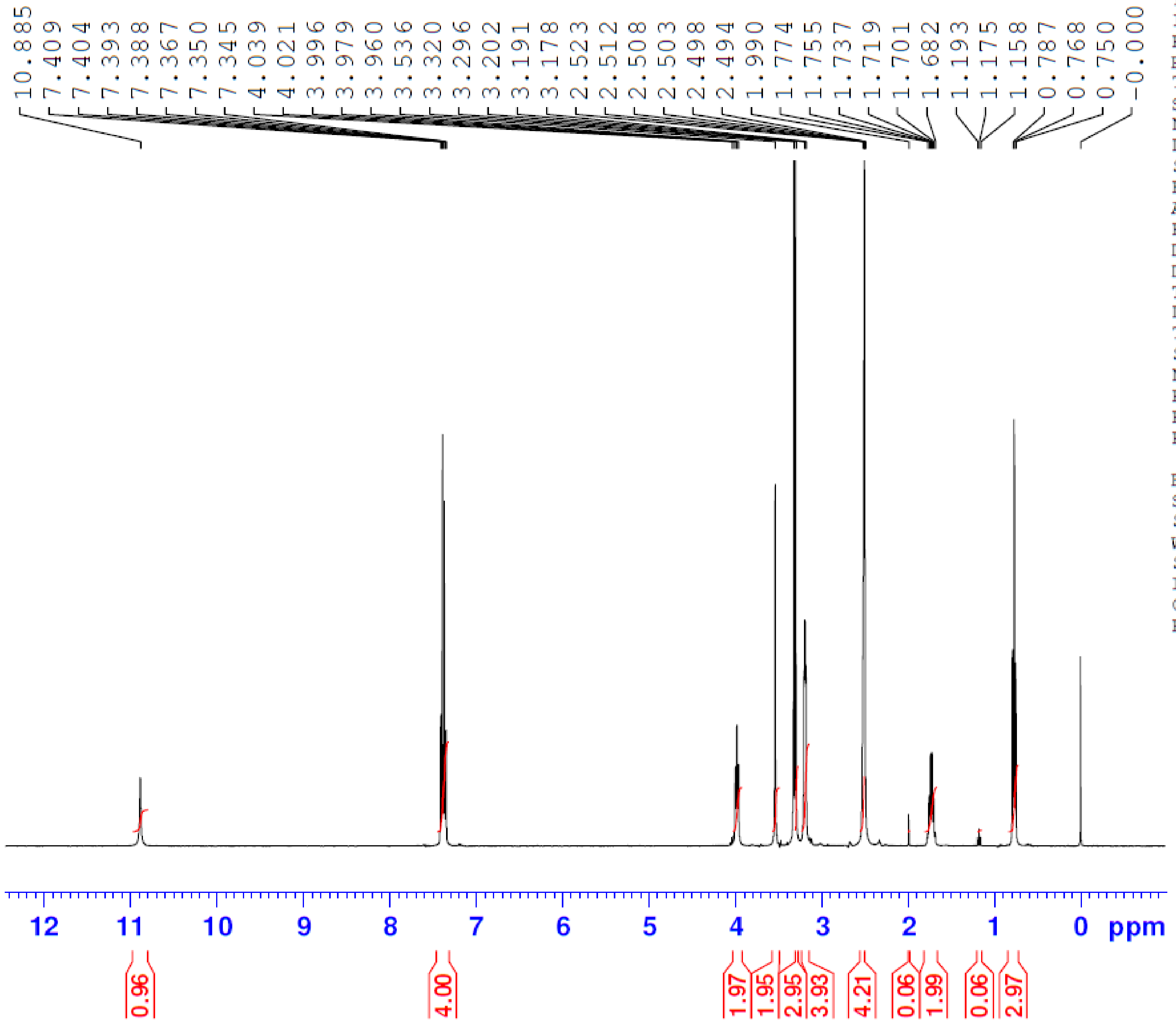

F2 - Acquisition Parameters
Date_ 20190705
Time 18.51 h
INSTRUM Avance Neo
PROBHD Z116098_0787 (
PULPROG zg30
TD 65536
SOLVENT DMSO
NS 16
DS 0
SWH 7142.857 Hz
FIDRES 0.217983 Hz
AQ 4.5875201 sec
RG 101
DW 70.000 usec
DE 14.62 usec
TE 298.1 K
D1 2.00000000 sec
TD0 1
SFO1 400.4024725 MHz
NUC1 1H
P0 3.33 usec
P1 10.00 usec
PLW1 19.73600006 W

F2 - Processing parameters
SI 65536
SF 400.4000013 MHz
WDW EM
SSB 0
LB 0.30 Hz
GB 0
PC 1.00

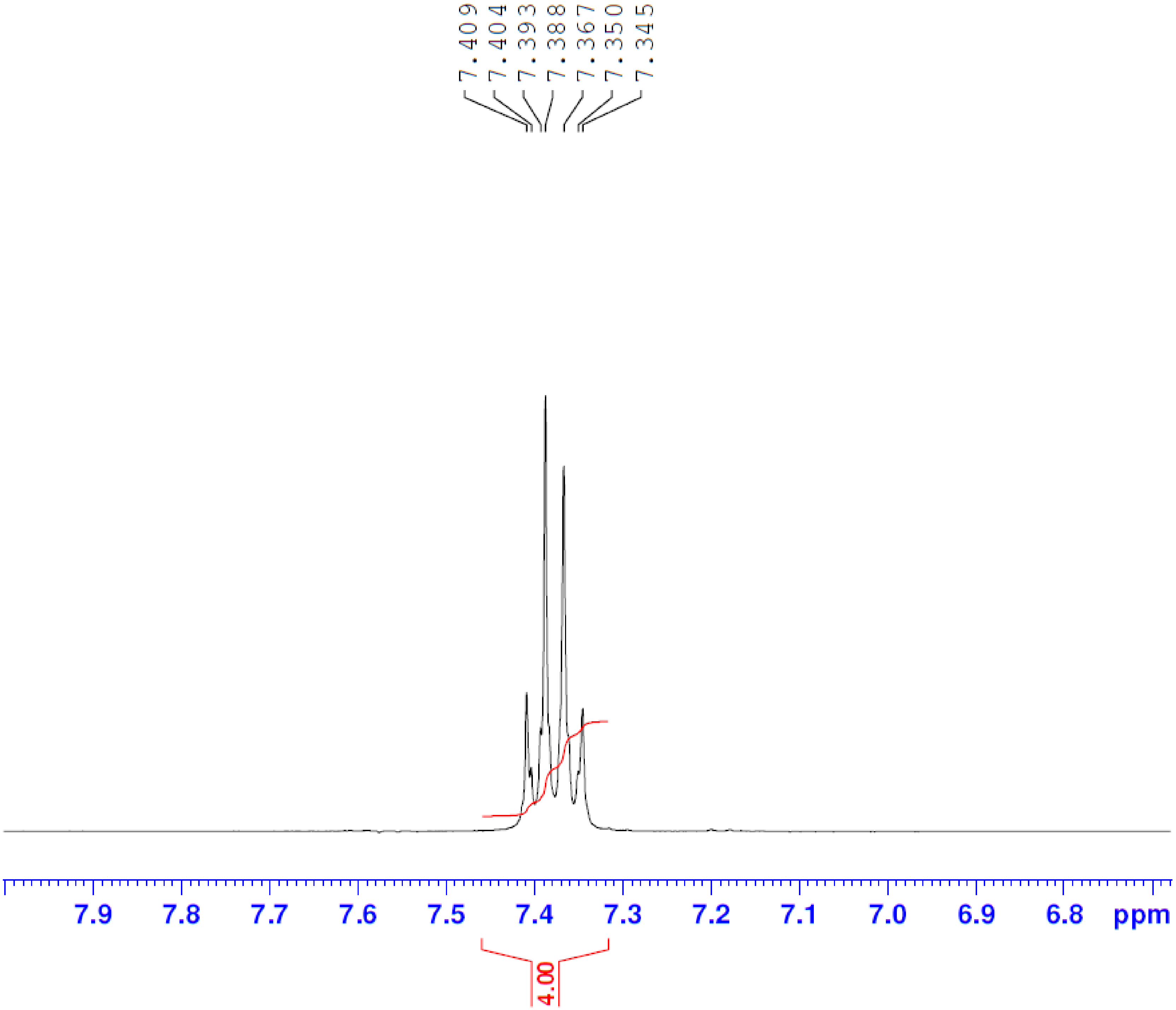
7.409
7.404
7.393
7.388
7.367
7.350
7.345
7.9
7.8
7.7
7.6
7.5
7.4
7.3
7.2
7.1
7.0
6.9
6.8
ppm
4.00

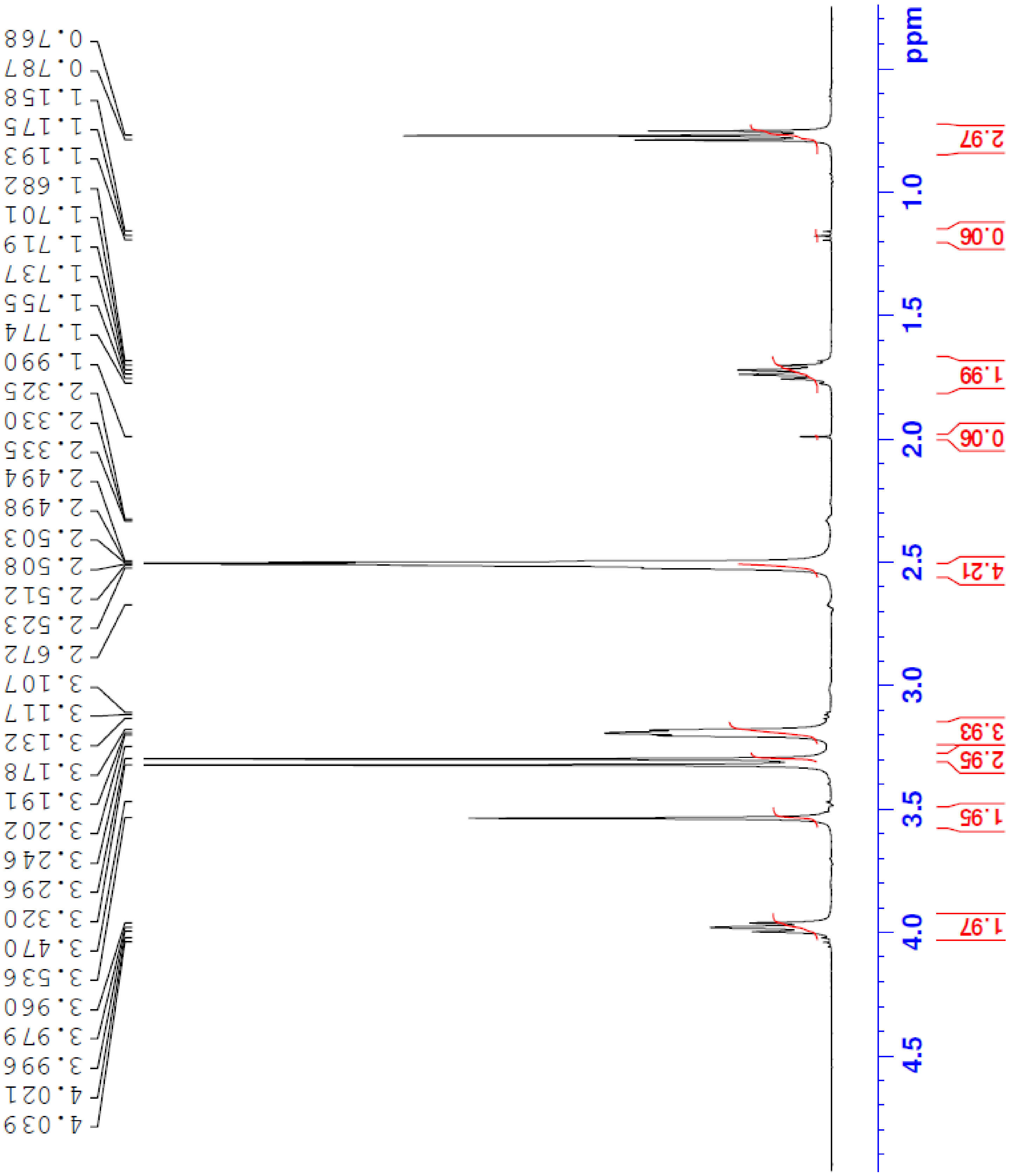
0.768
0.787
1.158
1.175
1.193
1.682
1.701
1.719
1.737
1.755
1.774
1.990
2.325
2.330
2.335
2.494
2.498
2.503
2.508
2.512
2.523
2.672
3.107
3.117
3.132
3.178
3.191
3.202
3.246
3.296
3.320
3.470
3.536
3.960
3.979
3.996
4.021
4.039
ppm
1.0
1.5
2.0
2.5
3.0
3.5
4.0
4.5
2.97
0.06
1.99
0.06
4.21
3.93
2.95
1.95
1.97

D_2O Exchange

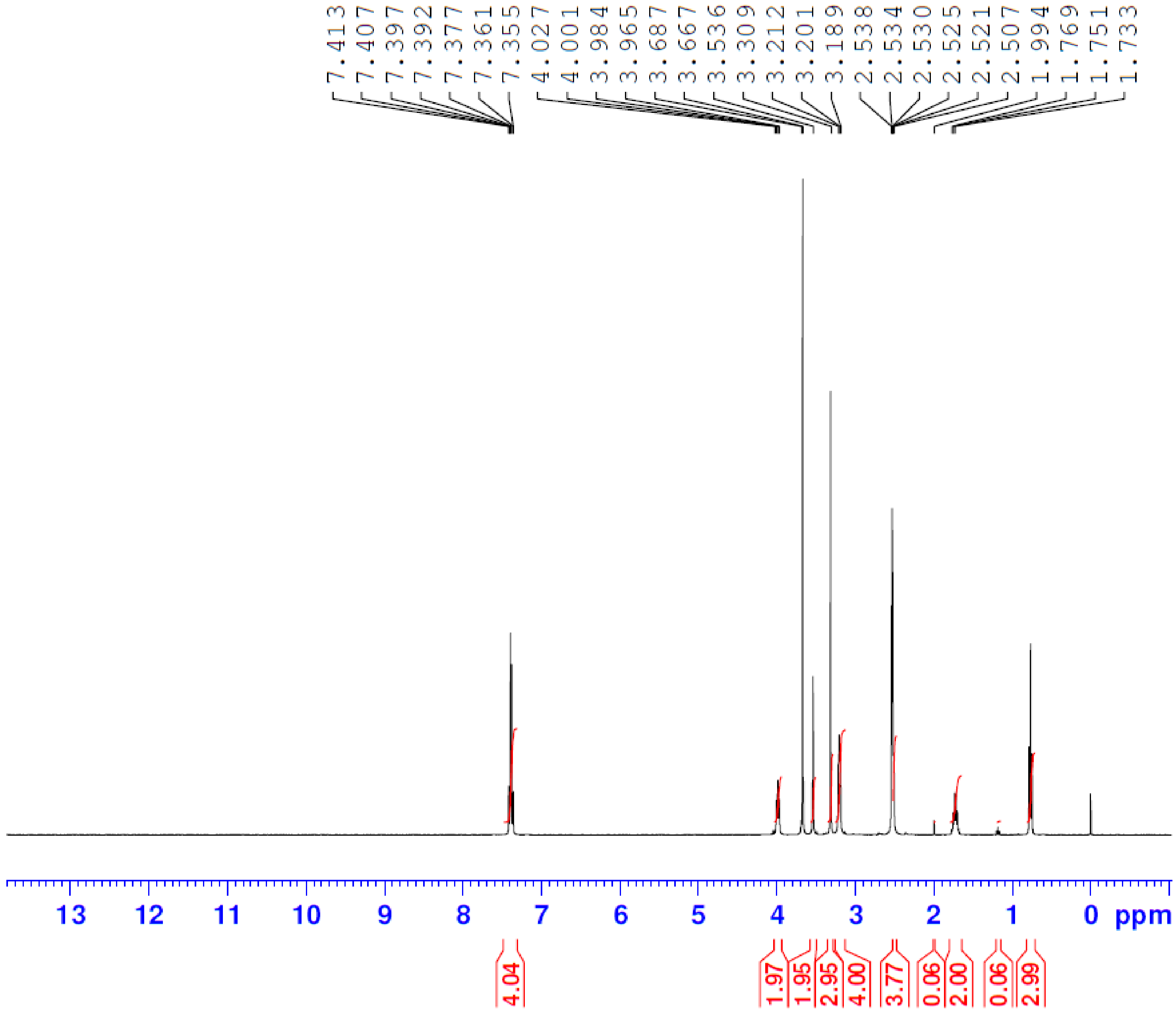

F2 - Acquisition Parameters

Date_	20190705
Time	23.57 h
INSTRUM	Avance Neo
PROBHD	Z116098_0787 (
PULPROG	zg30
TD	65536
SOLVENT	DMSO
NS	16
DS	0
SWH	7142.857 Hz
FIDRES	0.217983 Hz
AQ	4.5875201 sec
RG	101
DW	70.000 usec
DE	14.62 usec
TE	298.1 K
D1	2.00000000 sec
TD0	1
SFO1	400.4024725 MHz
NUC1	1H
P0	3.33 usec
P1	10.00 usec
PLW1	19.73600006 W

F2 - Processing parameters

SI		65536
SF		400.3999908 MHz
WDW		EM
SSB	0	
LB		0.30 Hz
GB	0	
PC		1.00

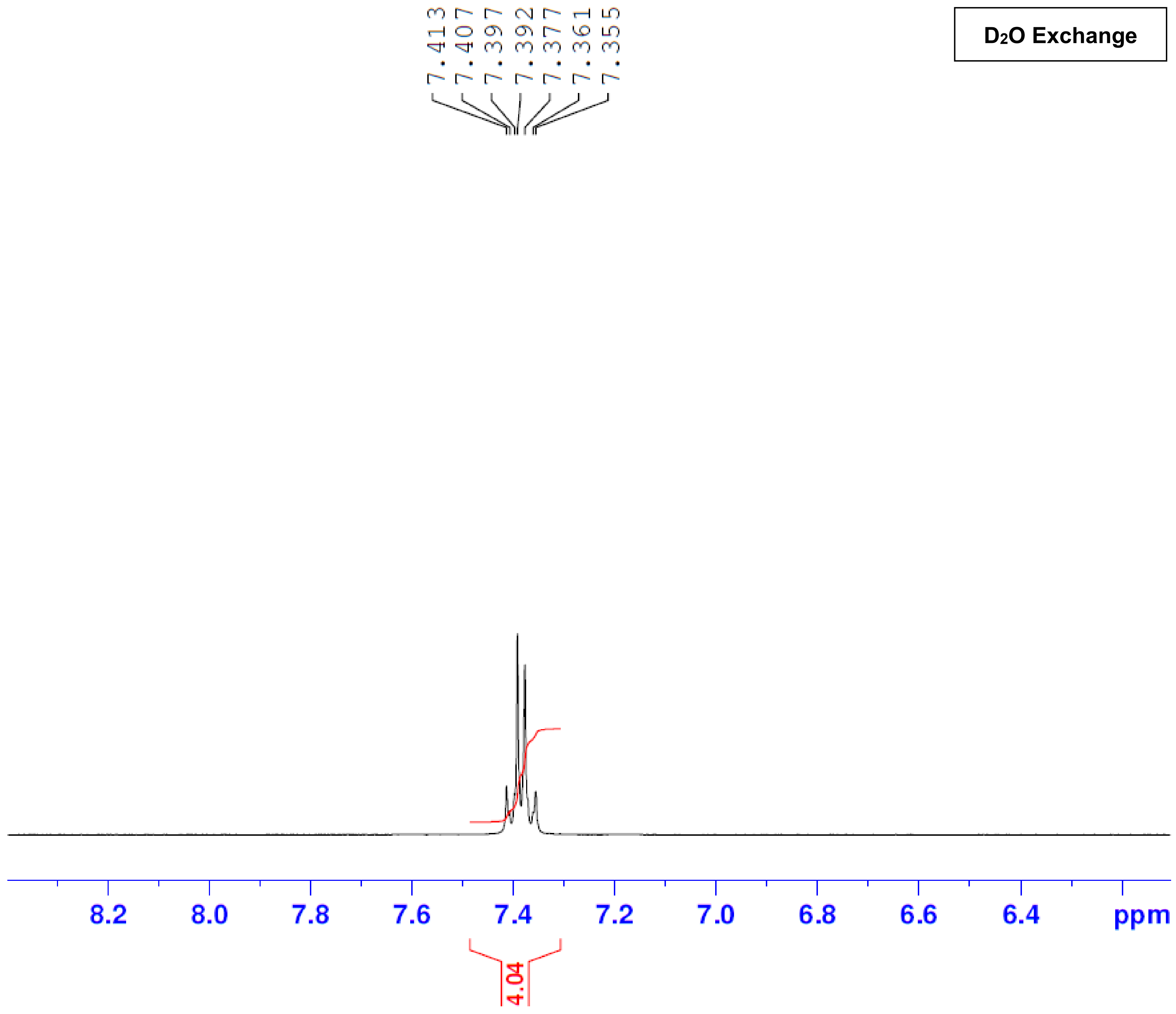

7.413
7.407
7.397
7.392
7.377
7.361
7.355
D2O Exchange
8.2
8.0
7.8
7.6
7.4
7.2
7.0
6.8
6.6
6.4
ppm
4.04

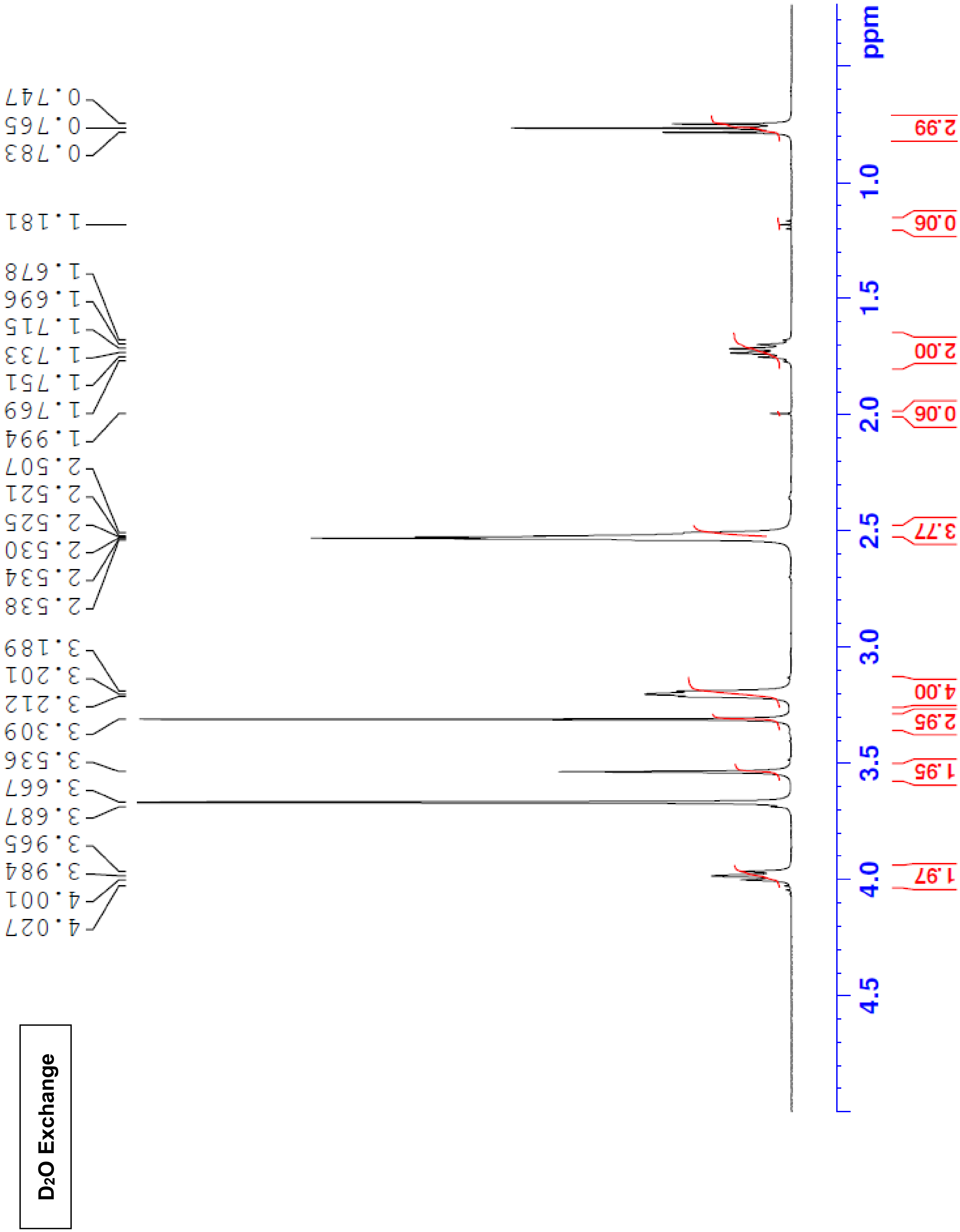

D2O Exchange
0.747
0.765
0.783
1.181
1.678
1.696
1.715
1.733
1.751
1.769
1.994
2.507
2.521
2.525
2.530
2.534
2.538
3.189
3.201
3.212
3.309
3.536
3.667
3.687
3.965
3.984
4.001
4.027
ppm
1.0
1.5
2.0
2.5
3.0
3.5
4.0
4.5
2.99
0.06
2.00
0.06
3.77
4.00
2.95
1.95
1.97

UPLC Method Conditions :

Column : Acquity UPLC BEH C18 (2.1x100) mm, 1.7µm
Mobile Phase-A : 0.05% TFA in Water
Mobile Phase-B : 0.05% TFA in Acetonitrile
Gradient (T/% B) : 0/10,4/90,6/90,6.1/10
Flow Rate : 0.3 mL/min
Temparature : 40℃
Diluent : ACN+Water

Auto-Scaled Chromatogram

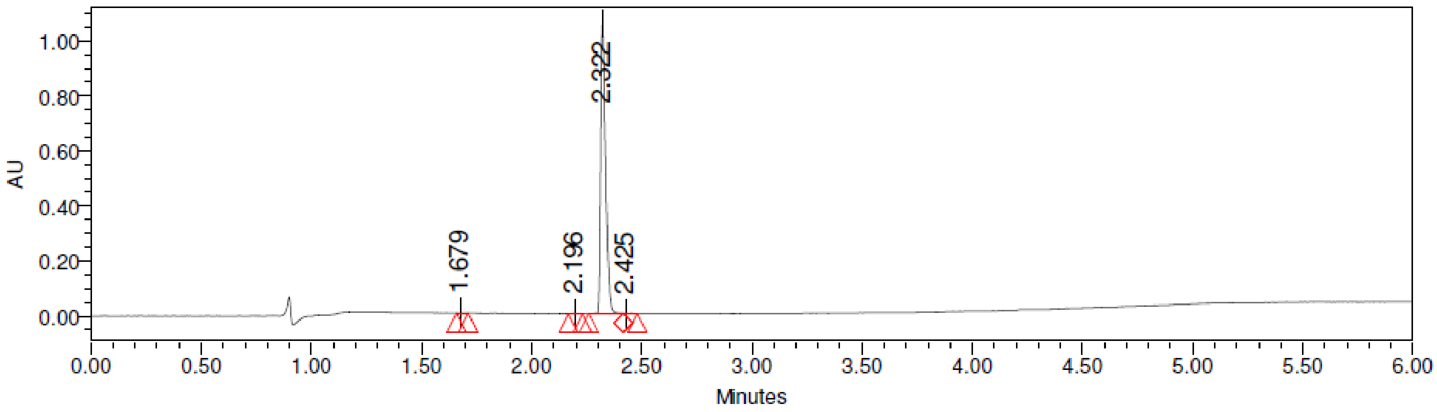

Peak Results

	RT	Area	Height	% Area
1	1.679	1005	916	0.06
2	2.196	2770	1677	0.17
3	2.322	1663791	1059196	99.58
4	2.425	3210	1457	0.19

GVK BIO Sciences Private Limited
Discovery Chemistry-Analytical Services
==

Sample ID :X Cluster Set-1-Target 5 (C3804-193A2) | **Date of analysis : 06-Jul-2019/12:03:29**
Acq Method :ATR-1 | **Instrument ID: ANL-MCL2-LCMS-001**
2:C,4

021907A7814_X Cluster Set-1-Target 5 (C3804-193A2)-AA

5: Diode Array
220
Range: 1.458

Time	Height	Area	Area%
1.75	1454711	58926.02	99.29
1.94	4439	120.34	0.20
2.76	8387	143.33	0.24
3.61	7385	158.39	0.27

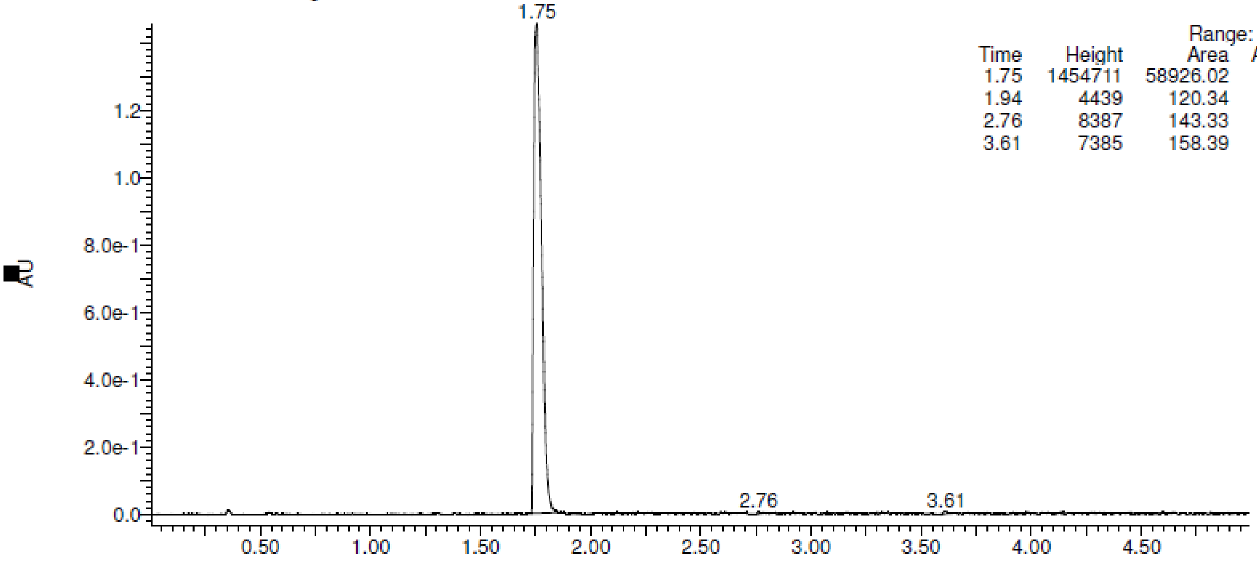

021907A7814_X Cluster Set-1-Target 5 (C3804-193A2)-AA

1: Scan ES+
417.496
2.72e6

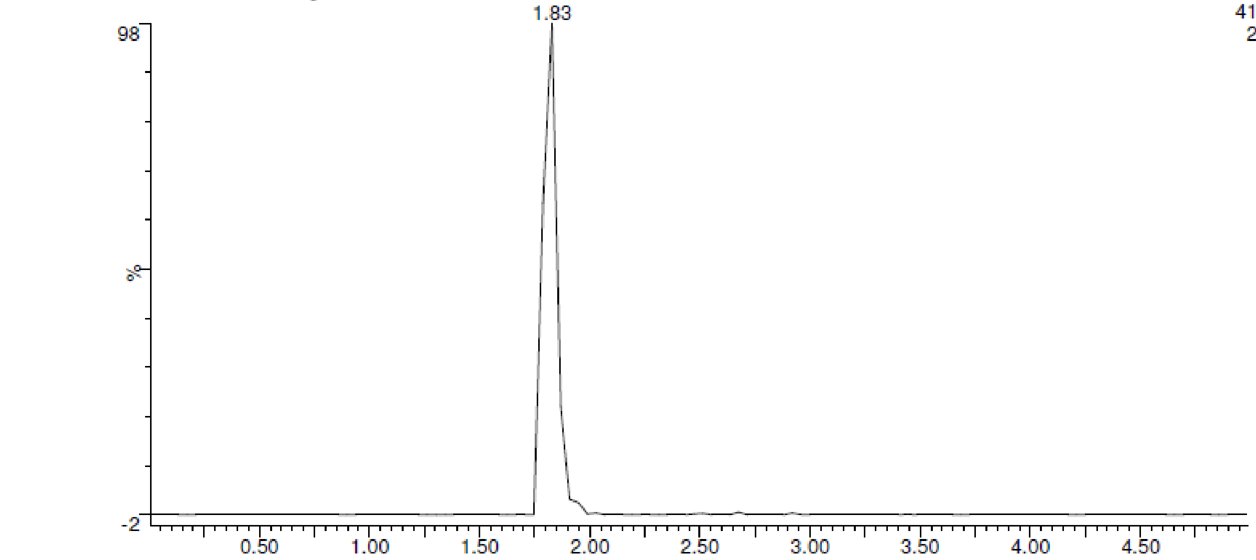

021907A7814_X Cluster Set-1-Target 5 (C3804-193A2)-AA

1: Scan ES+
TIC
4.58e6

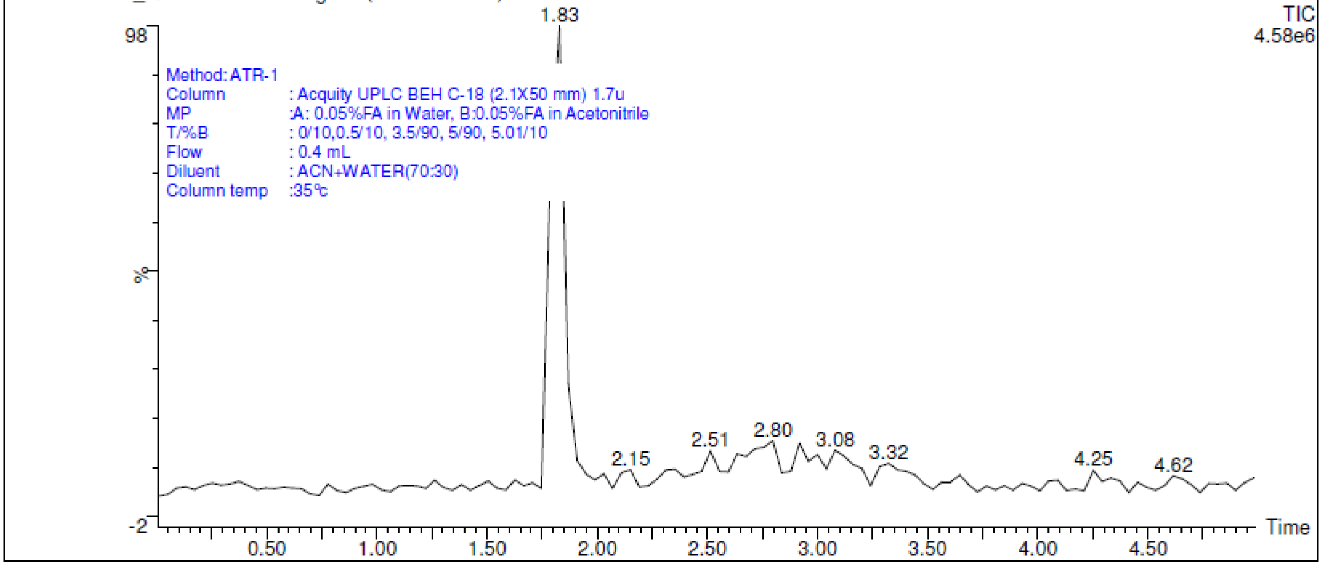

GVK Biosciences Private Limited
Discovery Chemistry-Analytical Services
================================

Sample ID: X Cluster Set-1-Target 5 (C3804-193A2) **Date of analysis: 06-Jul-2019:12:03:29**
Acq Method :ATR-1 **Instrument ID:ANL-MCL2-LCMS-001**
2:C,4

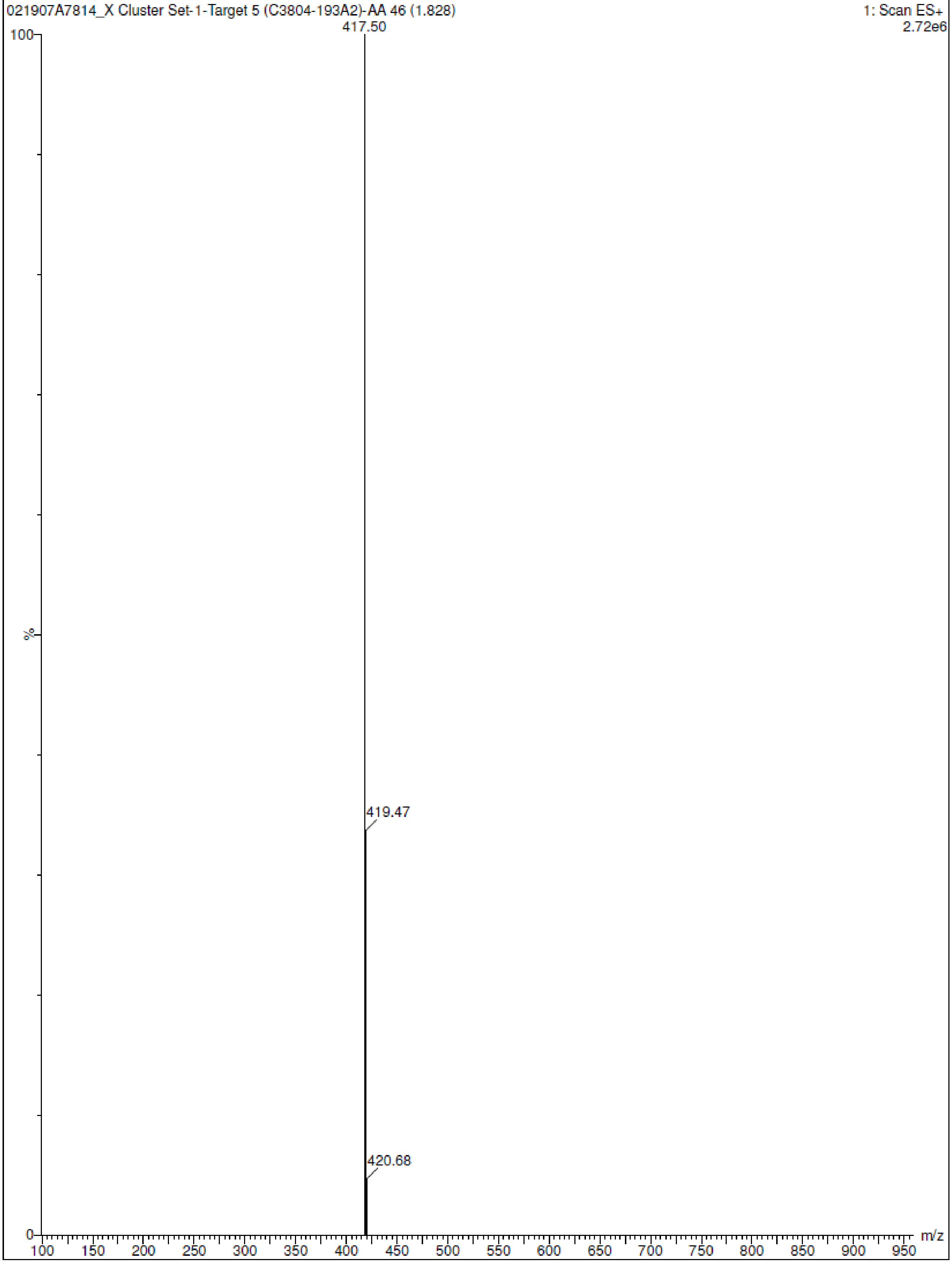

PL_6 (6f)

S.No	Test	Results
1	**Description**	Off-white solid
2	**Identification**<br>(a) **NMR**<br>(b) **Mass by LCMS**	Complies to structure<br>456.52 $[M+H]^+$
3	**Chromatographic Purity by UPLC (Area %)**<br>Impurities>1.0%<br>Rt-2.11 minutes	96.33<br><br>3.45
4	**Chromatographic Purity by LCMS (Area %)**<br>Impurities>1.0%<br>Rt-1.57 minutes	96.31<br><br>2.01

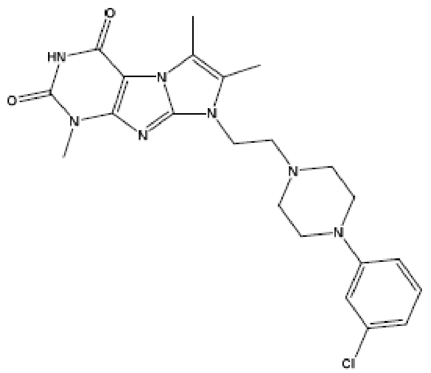

Solvent: dmso
Date: Jul 15 2019
Agilent 400-MR / NRM-3
Request No: 021907B8538-X_Cluster_Set-1-Target_7_C4537-001A1__PROTON

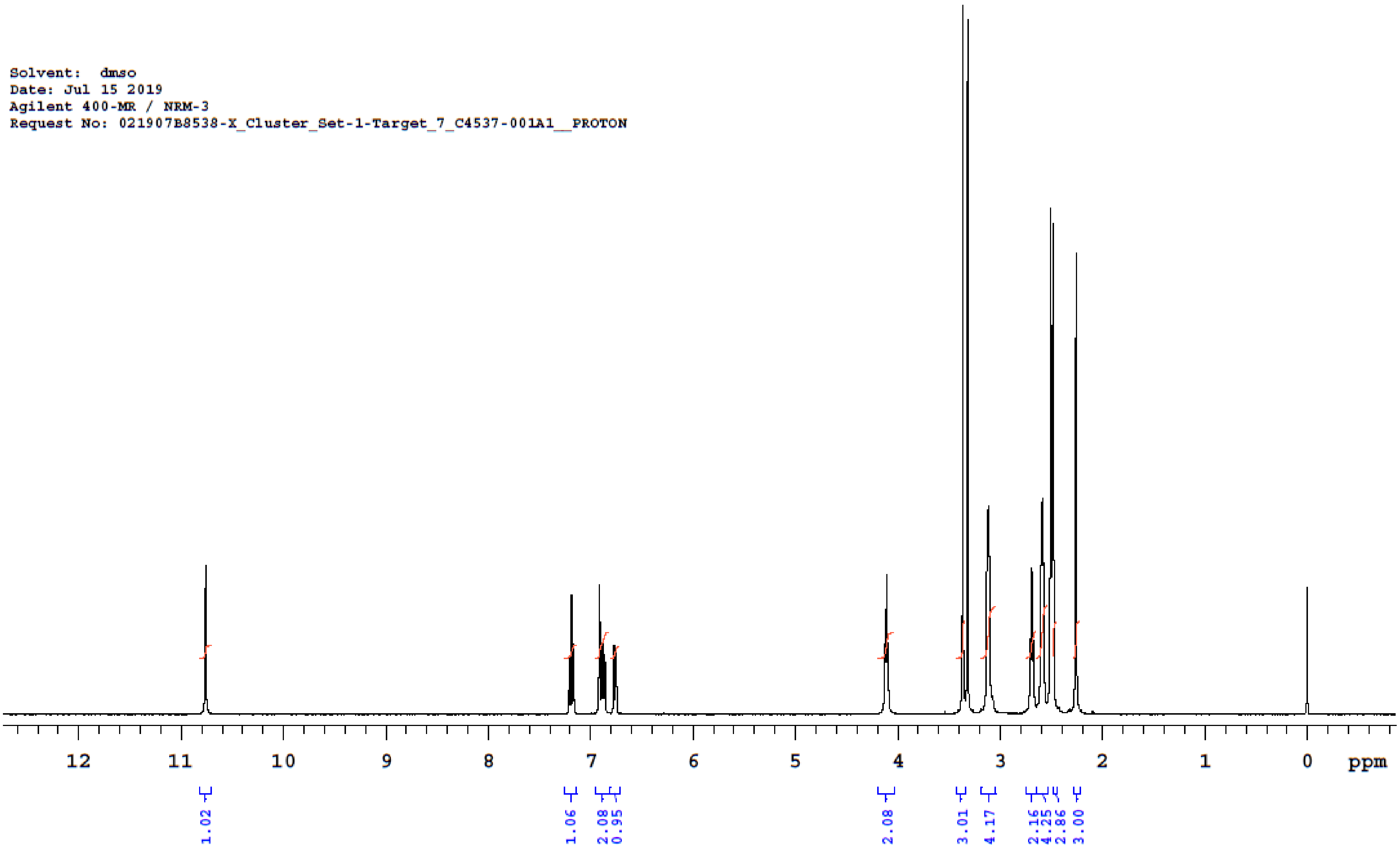

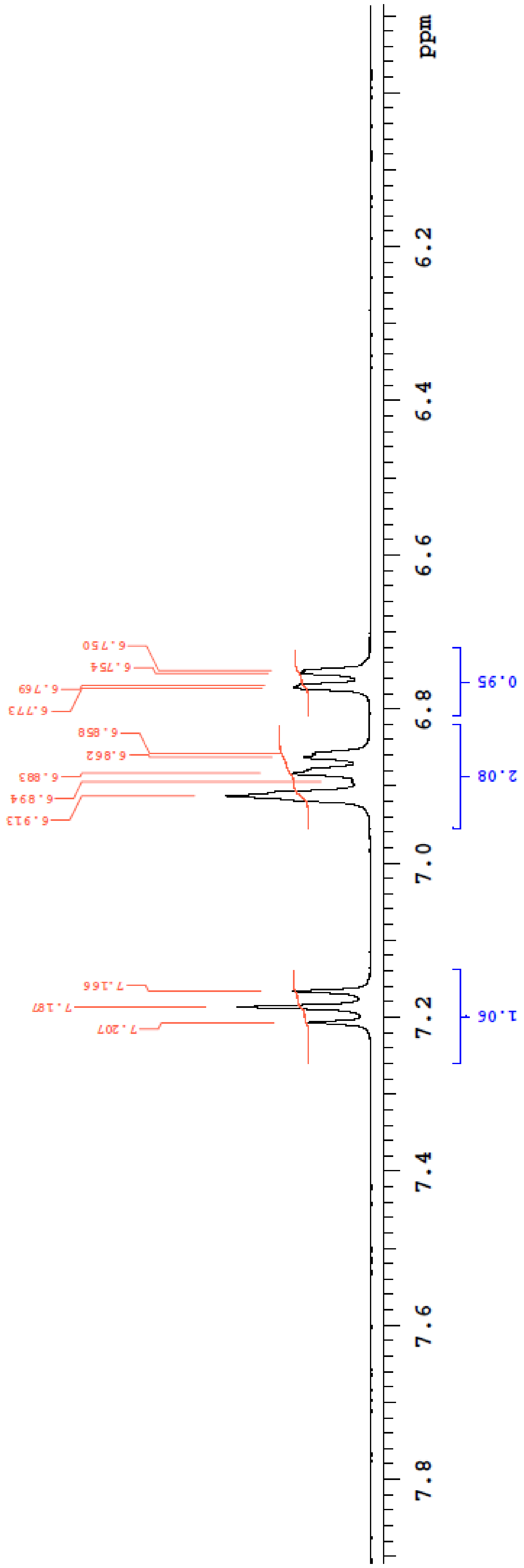
ppm
6.2
6.4
6.6
6.8
7.0
7.2
7.4
7.6
7.8
6.750
6.754
6.769
6.773
6.858
6.862
6.883
6.894
6.913
7.166
7.187
7.207
0.95
2.08
1.06

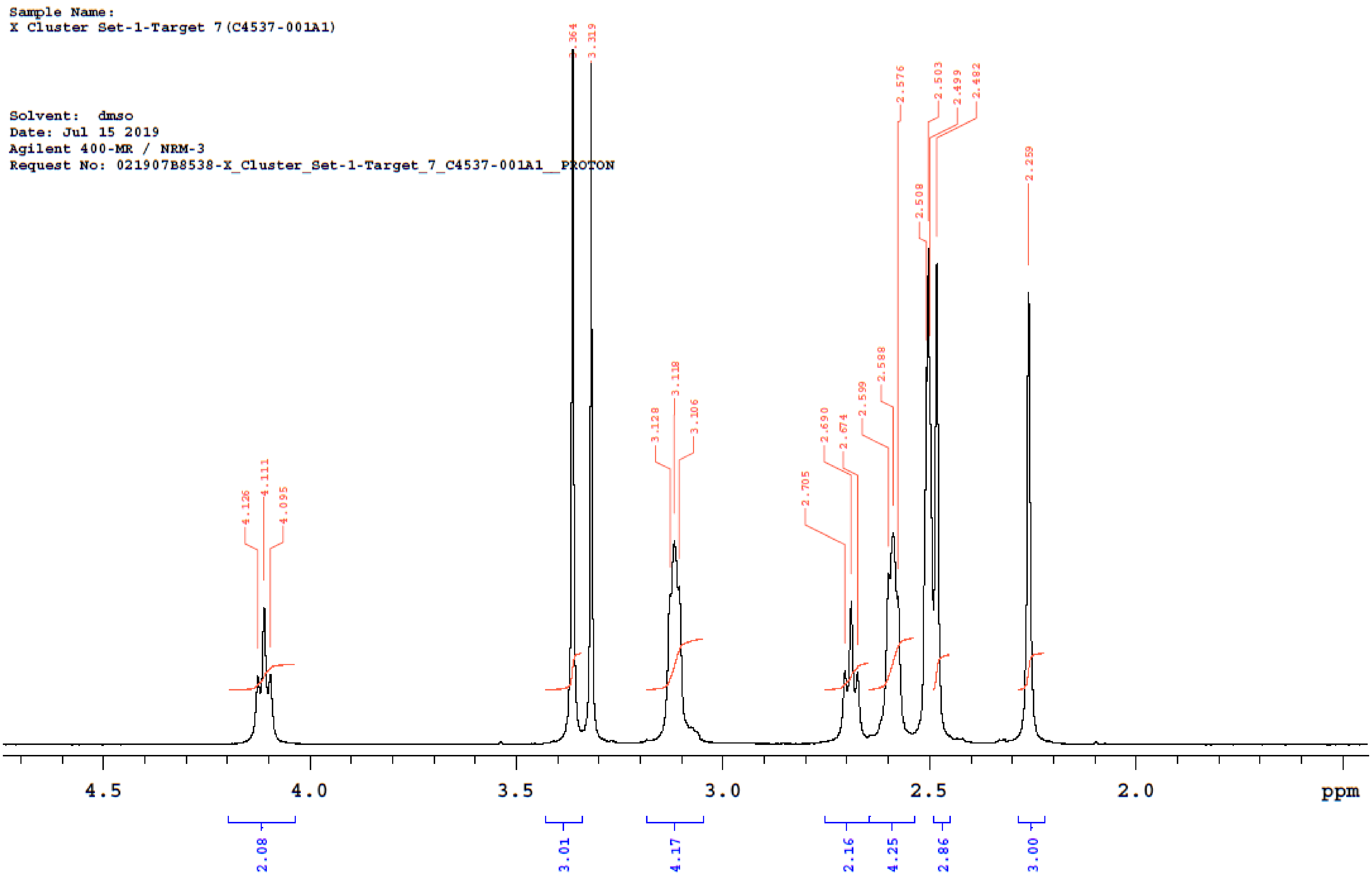
Sample Name:
X Cluster Set-1-Target 7(C4537-001A1)
Solvent: dmso
Date: Jul 15 2019
Agilent 400-MR / NRM-3
Request No: 021907B8538-X_Cluster_Set-1-Target_7_C4537-001A1__PROTON
4.126
4.111
4.095
3.364
3.319
3.128
3.118
3.106
2.705
2.690
2.674
2.599
2.588
2.576
2.508
2.503
2.499
2.482
2.259
4.5
4.0
3.5
3.0
2.5
2.0
ppm
2.08
3.01
4.17
2.16
4.25
2.86
3.00

D_2O Exchange

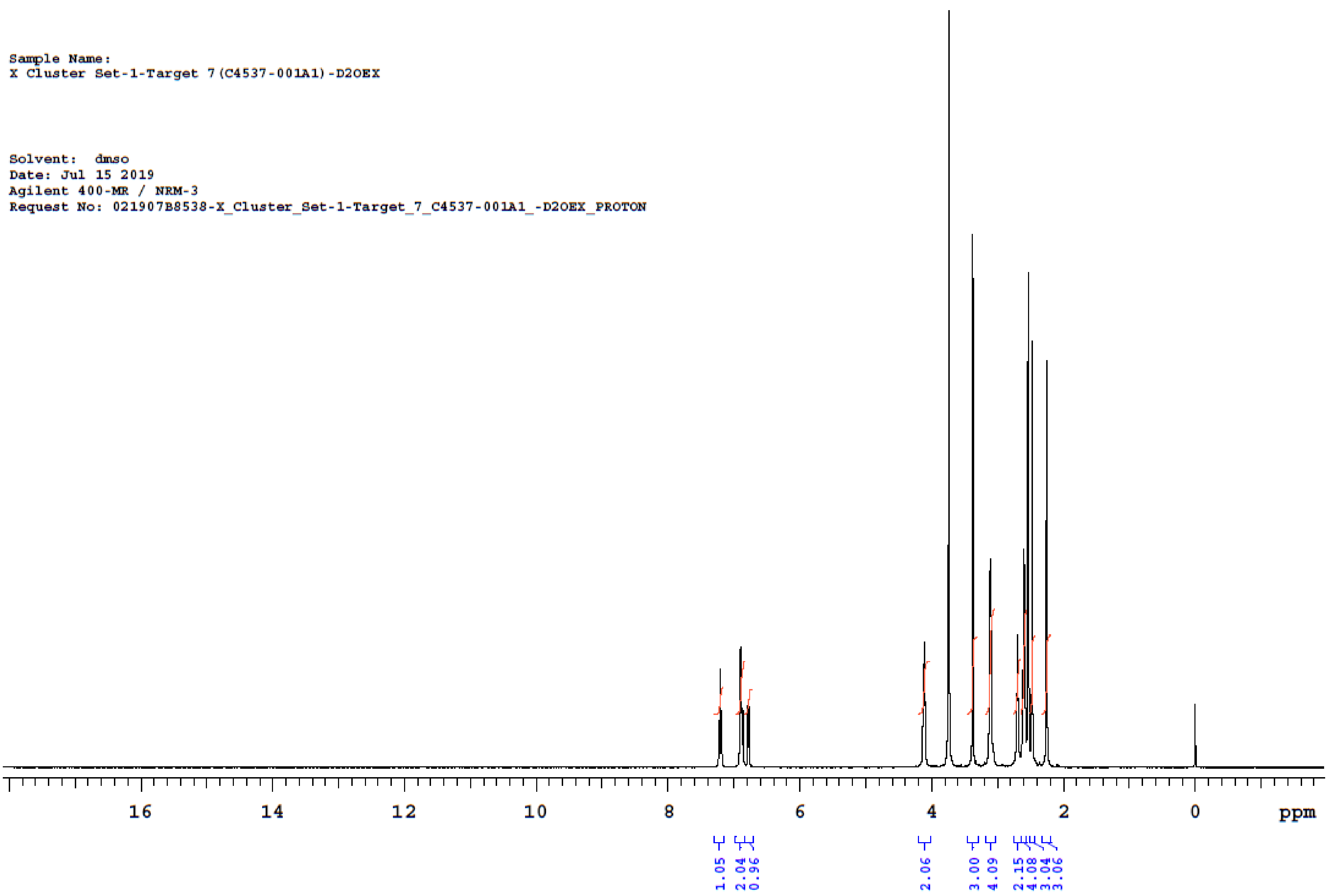

D_2O Exchange

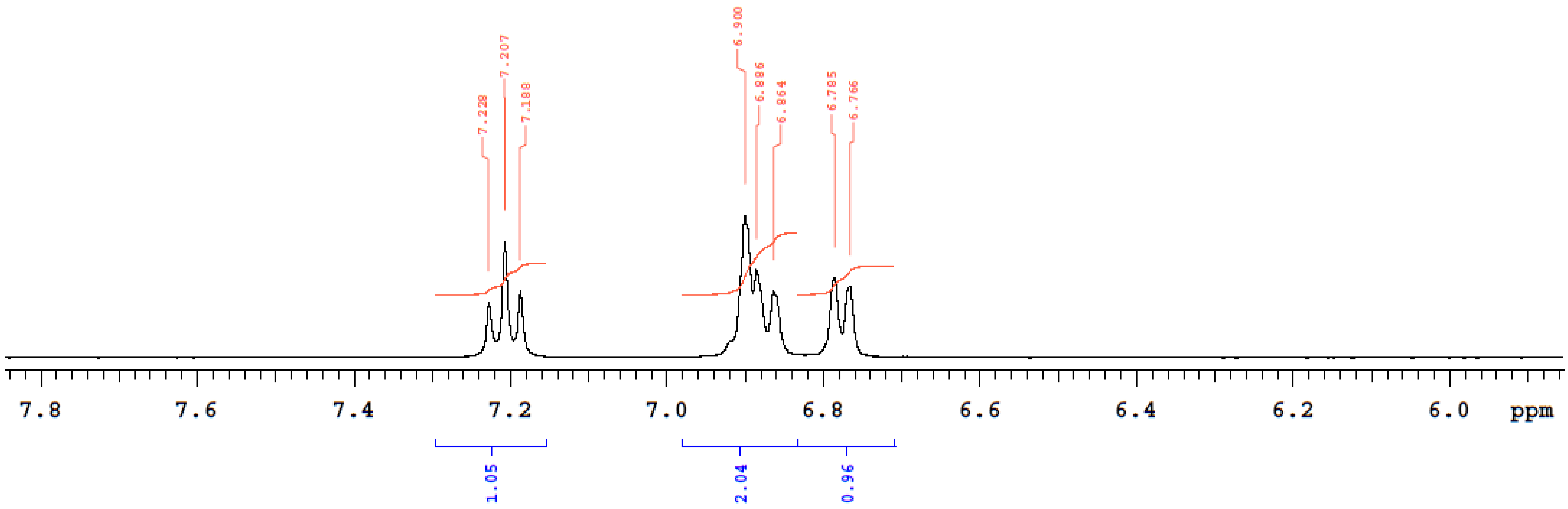

D$_2$O Exchange

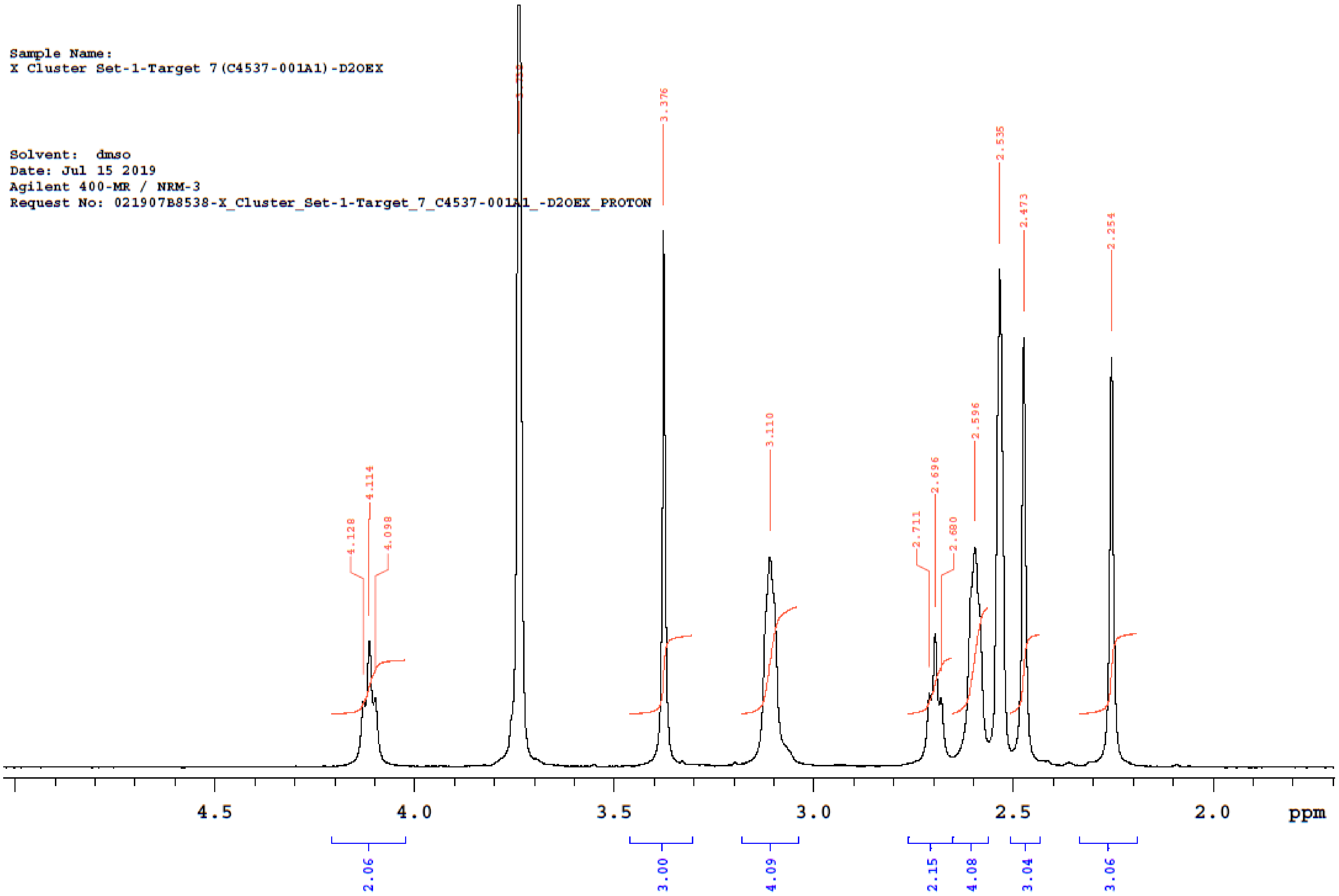

UPLC Method Conditions :

Column : Acquity UPLC BEH C18 (2.1x100) mm, 1.7µm
Mobile Phase-A : 0.05% TFA in Water
Mobile Phase-B : 0.05% TFA in Acetonitrile
Gradient (T/% B) : 0/10,4/90,6/90,6.1/10
Flow Rate : 0.3 mL/min
Temparature : 40 °C
Diluent : ACN+Water

Auto-Scaled Chromatogram

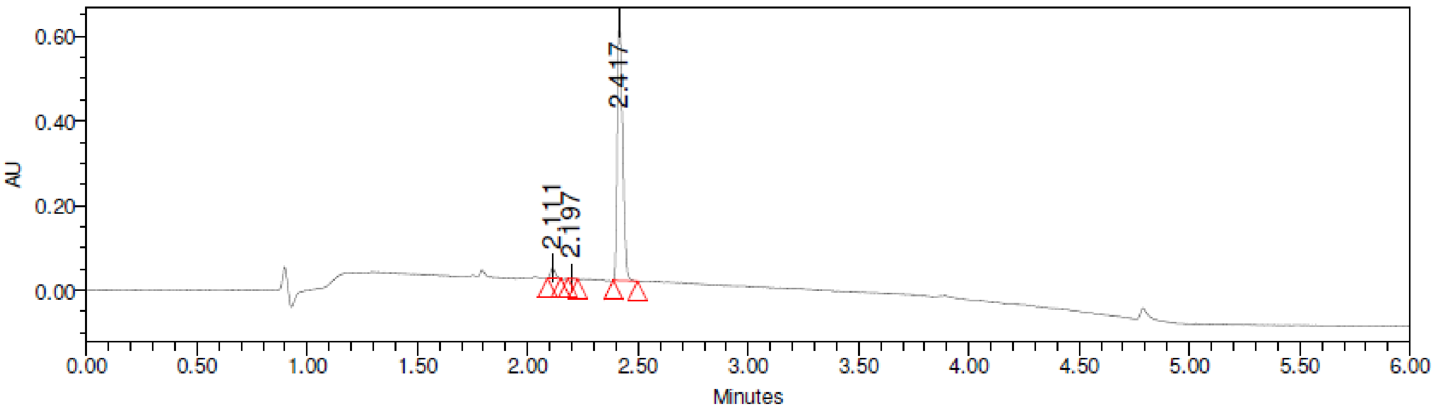

Peak Results

	RT	Area	Height	% Area
1	2.111	37009	23837	3.45
2	2.197	2429	1890	0.23
3	2.417	1033868	610123	96.33

GVK BIO Sciences Private Limited
Discovery Chemistry-Analytical Services
==

Sample ID :X Cluster Set-1-Target 7(C4537-001A1) **Date of analysis : 15-Jul-2019/13:44:51**
Acq Method :ATR-1 **Instrument ID: ANL-MCL2-LCMS-001**
1:D,6

021907B8538-X Cluster Set-1-Target 7(C4537-001A1)A Sm (Mn, 7x7) 5: Diode Array 210 Range: 3.698e-1

Time	Height	Area	Area%
1.39	1841	80.38	0.65
1.57	9922	248.12	2.01
1.83	368828	11863.56	96.31
2.21	1718	57.25	0.46
2.31	1141	36.78	0.30
2.57	1179	32.32	0.26

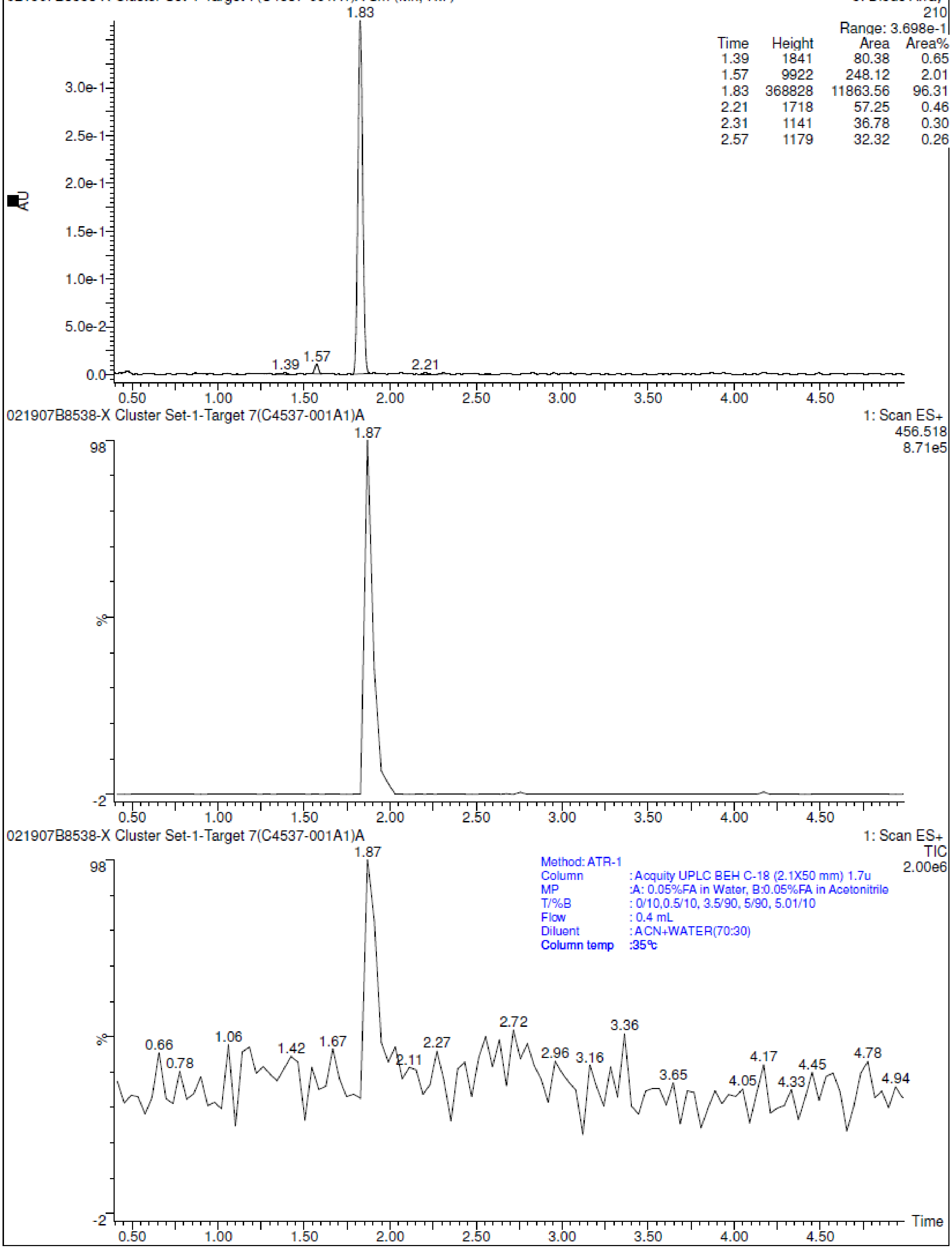

GVK Biosciences Private Limited
Discovery Chemistry-Analytical Services
================================

Sample ID: X Cluster Set-1-Target 7(C4537-001A1) **Date of analysis: 15-Jul-2019:13:44:51**
Acq Method :ATR-1 **Instrument ID:ANL-MCL2-LCMS-001**
1:D,6

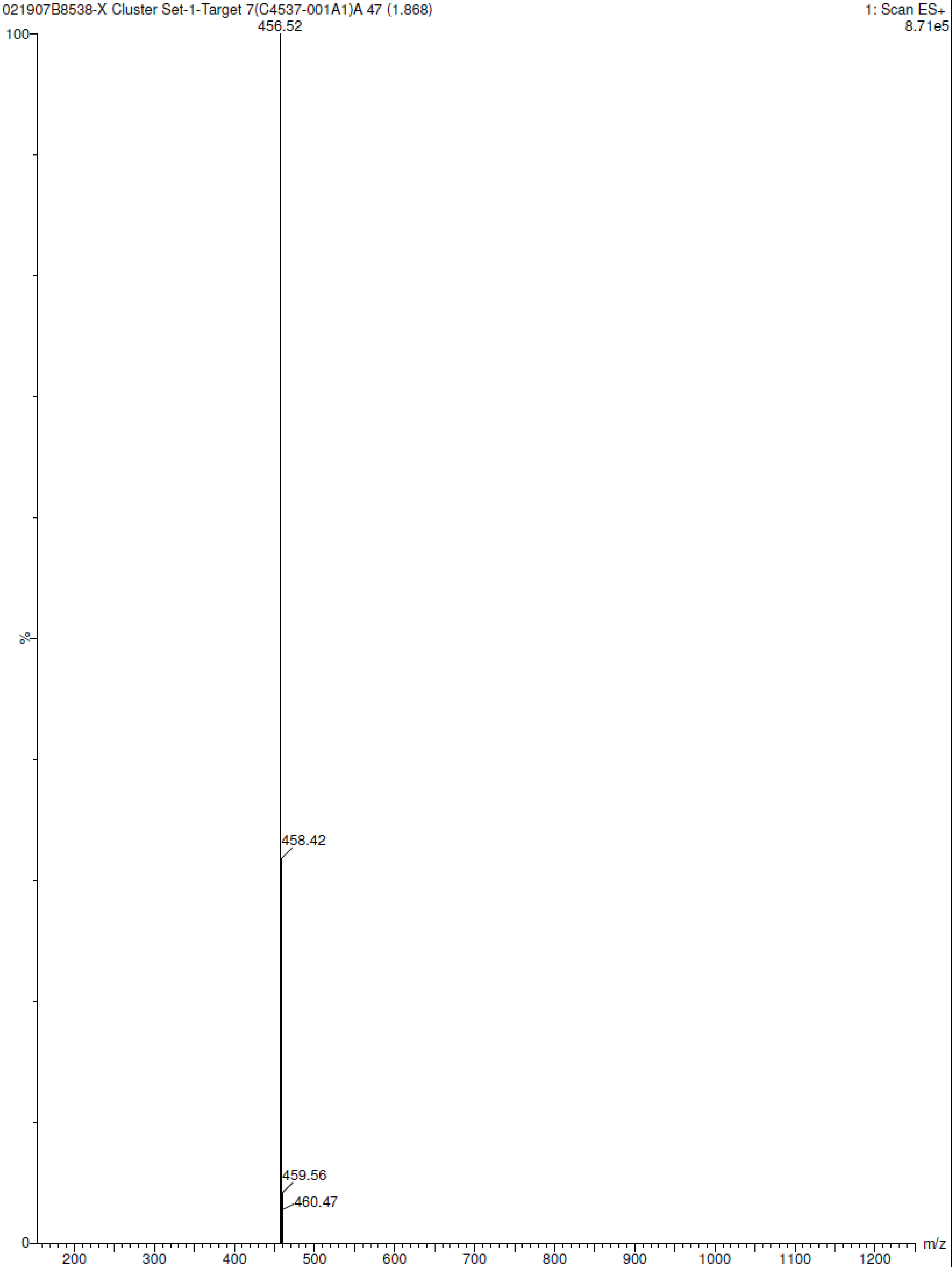

DCP_3 (13)

S.No	Test	Results
1	**Description**	Pale yellow solid
2	**Identification**<br>(a) **NMR**<br>(b) **Mass by LCMS**	 Complies to structure<br>340.13 [M+H]$^+$
3	**Chromatographic Purity by UPLC (Area %)**<br>Impurities>1.0%	96.48<br>Nil
4	**Chromatographic Purity by LCMS (Area %)**<br>Impurities>1.0%<br>Rt- 1.69 minutes<br>Rt- 2.11 minutes	96.43<br><br>2.48<br>1.09

Remarks:

1. Traces of Aliphatic impurities observed in NMR spectrum.

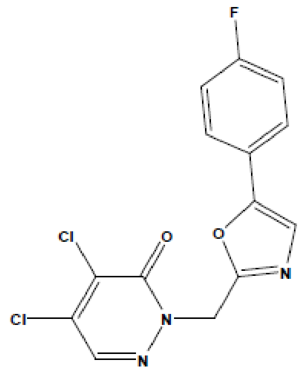

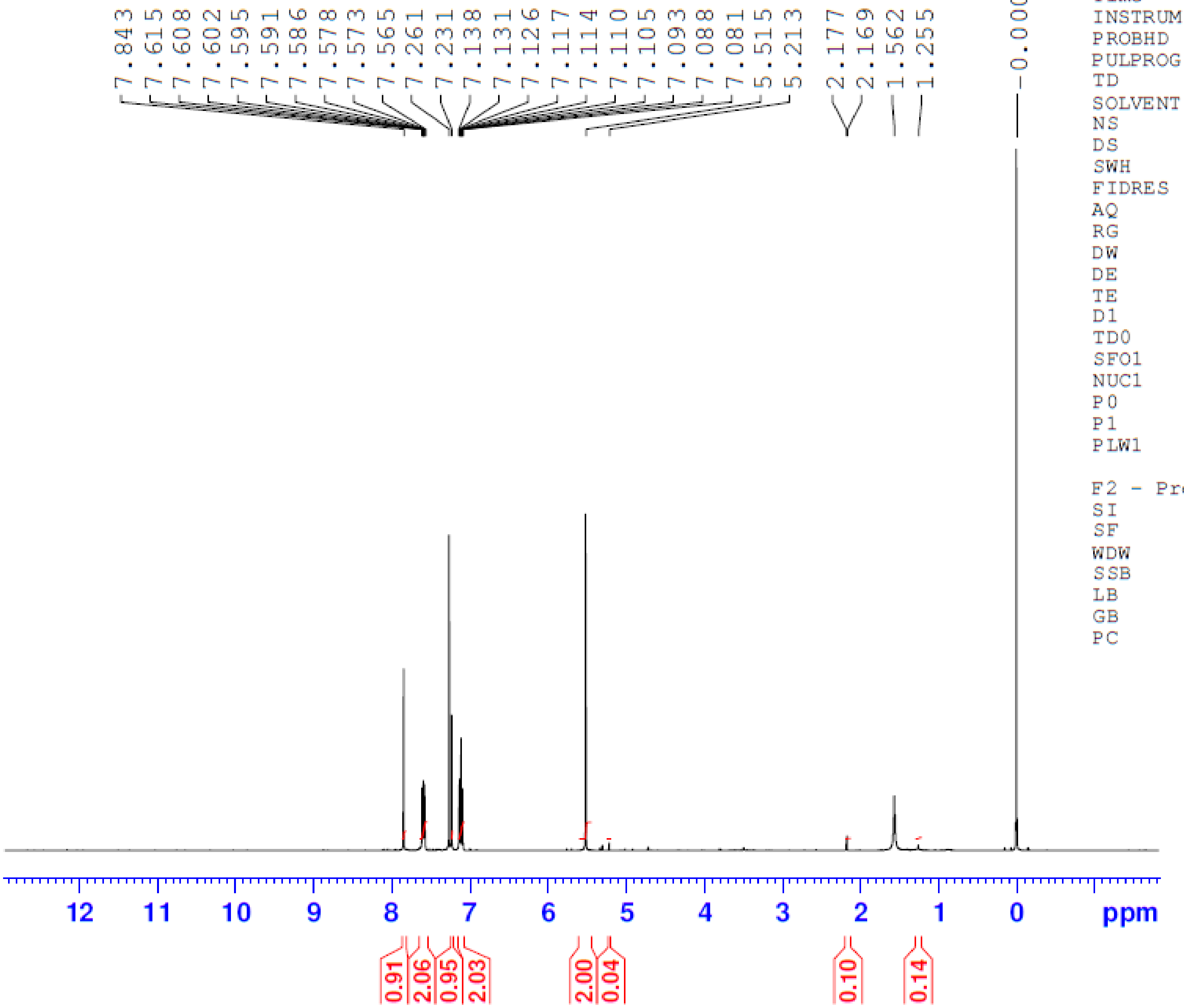

F2 - Acquisition Parameters		
Date_	20190930	
Time	18.36	h
INSTRUM	Avance Neo	
PROBHD	Z116098_0787 (	
PULPROG	zg30	
TD	65536	
SOLVENT	CDCl3	
NS	16	
DS	0	
SWH	7142.857	Hz
FIDRES	0.217983	Hz
AQ	4.5875201	sec
RG	101	
DW	70.000	usec
DE	14.62	usec
TE	298.1	K
D1	2.00000000	sec
TD0	1	
SFO1	400.4024725	MHz
NUC1	1H	
P0	3.33	usec
P1	10.00	usec
PLW1	19.73600006	W
F2 - Processing parameters		
SI	65536	
SF	400.4000097	MHz
WDW	EM	
SSB	0	
LB	0.30	Hz
GB	0	
PC	1.00	

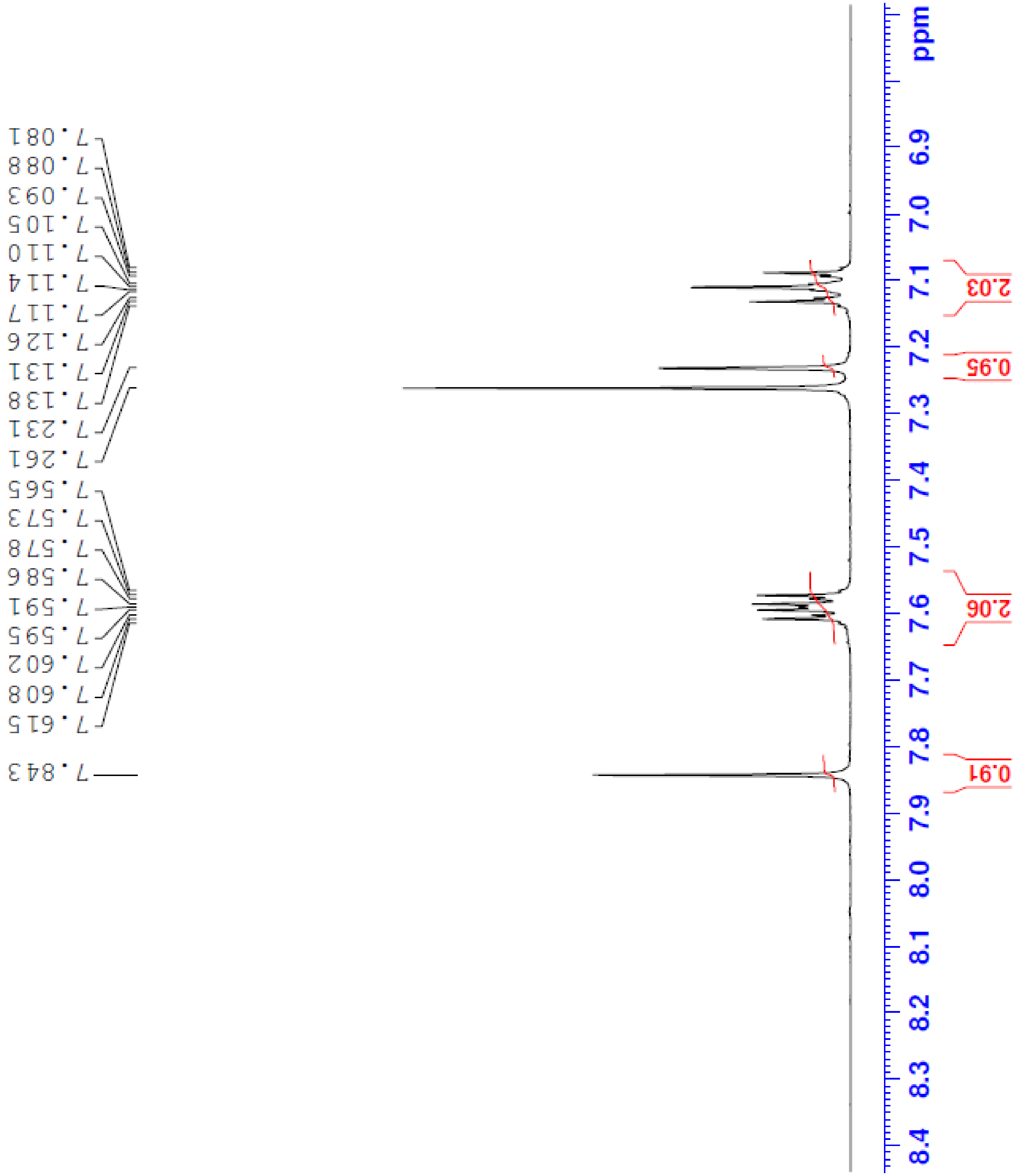
7.081
7.088
7.093
7.105
7.110
7.114
7.117
7.126
7.131
7.138
7.231
7.261
7.565
7.573
7.578
7.586
7.591
7.595
7.602
7.608
7.615
7.843
2.03
0.95
2.06
0.91
ppm
6.9
7.0
7.1
7.2
7.3
7.4
7.5
7.6
7.7
7.8
7.9
8.0
8.1
8.2
8.3
8.4

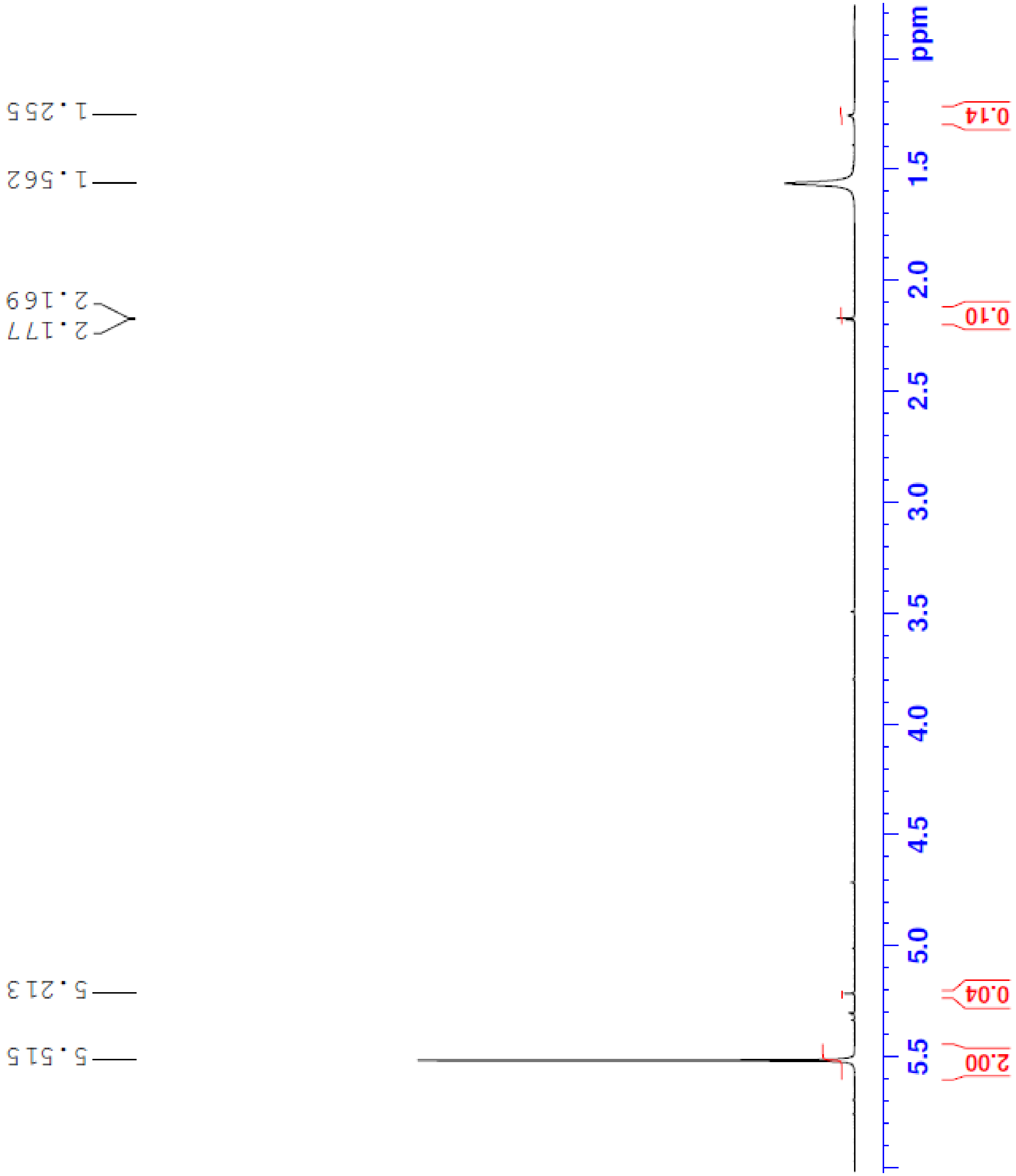
1.255
1.562
2.169
2.177
5.213
5.515
ppm
1.5
2.0
2.5
3.0
3.5
4.0
4.5
5.0
5.5
0.14
0.10
0.04
2.00

UPLC Method Conditions :

Column	: Acquity UPLC BEH C18 (2.1x100) mm, 1.7µm
Mobile Phase-A	: 0.05% TFA in Water
Mobile Phase-B	: 0.05% TFA in Acetonitrile
Gradient (T/% B)	: 0/30,4/90,6/90,6.1/30
Flow Rate	: 0.3 mL/min
Temparature	: 40℃
Diluent	: ACN+WATER

Auto-Scaled Chromatogram

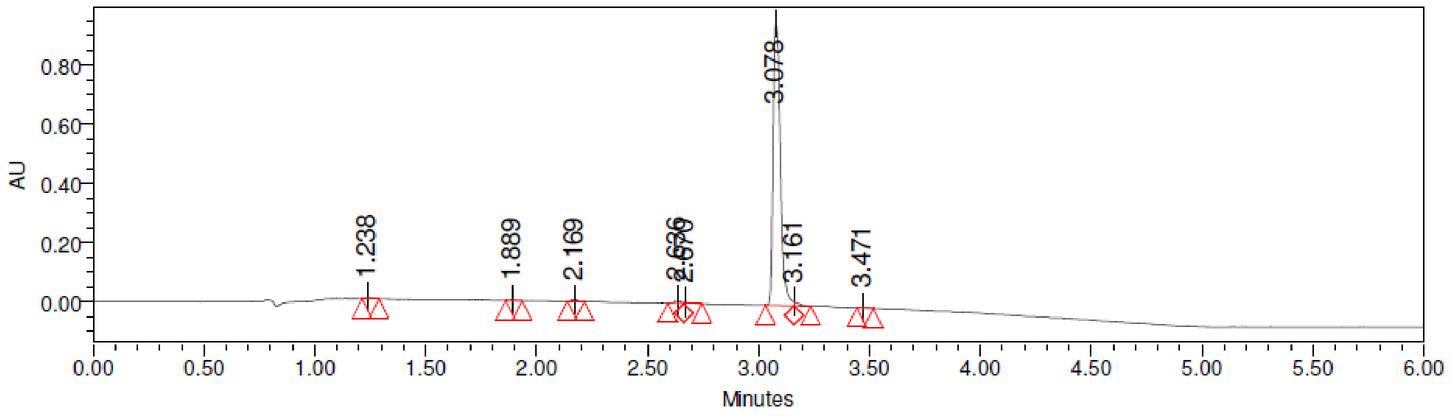

Peak Results

	RT	Area	Height	% Area
1	1.238	8707	4540	0.38
2	1.889	3379	1960	0.15
3	2.169	11334	5937	0.50
4	2.636	19357	9294	0.85
5	2.670	9812	5044	0.43
6	3.078	2200492	957112	96.48
7	3.161	20443	14158	0.90
8	3.471	7352	3469	0.32

GVK BIO Sciences Private Limited
Discovery Chemistry-Analytical Services
==

Sample ID :CPN CLUSTER Set-1-Target-2 (C4536-053-A1) **Date of analysis : 30-Sep-2019/23:02:28**
Acq Method :GVK-3 **Instrument ID: ANL-MCL2-LCMS-001**
2:F,2

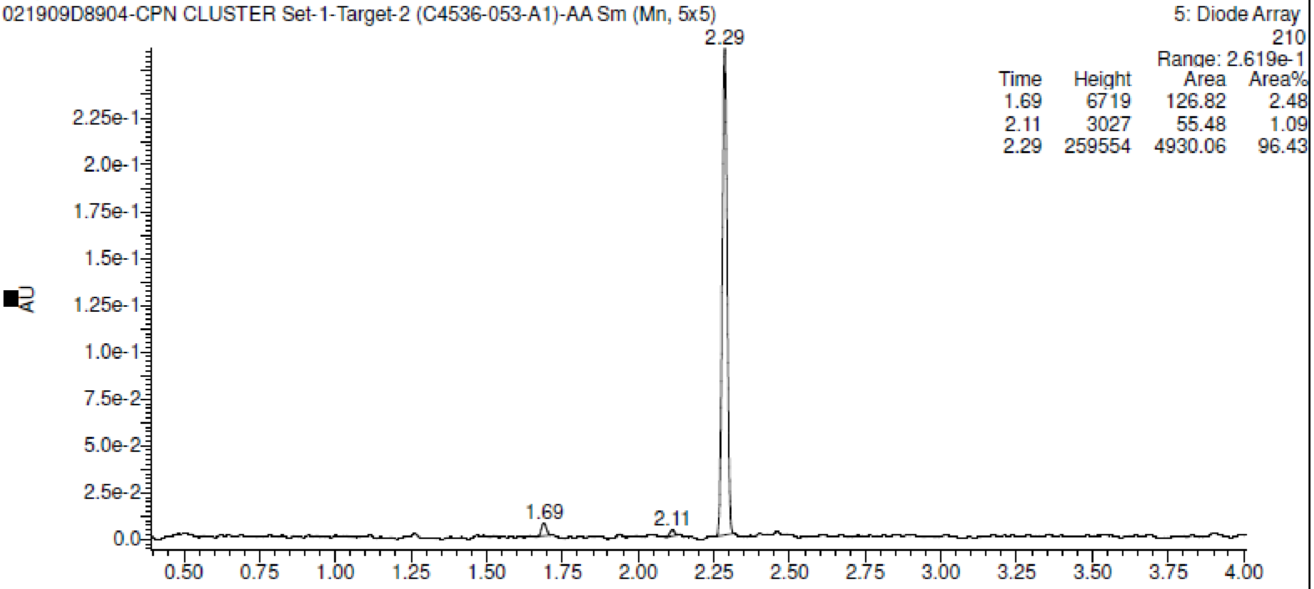

Time	Height	Area	Area%
1.69	6719	126.82	2.48
2.11	3027	55.48	1.09
2.29	259554	4930.06	96.43

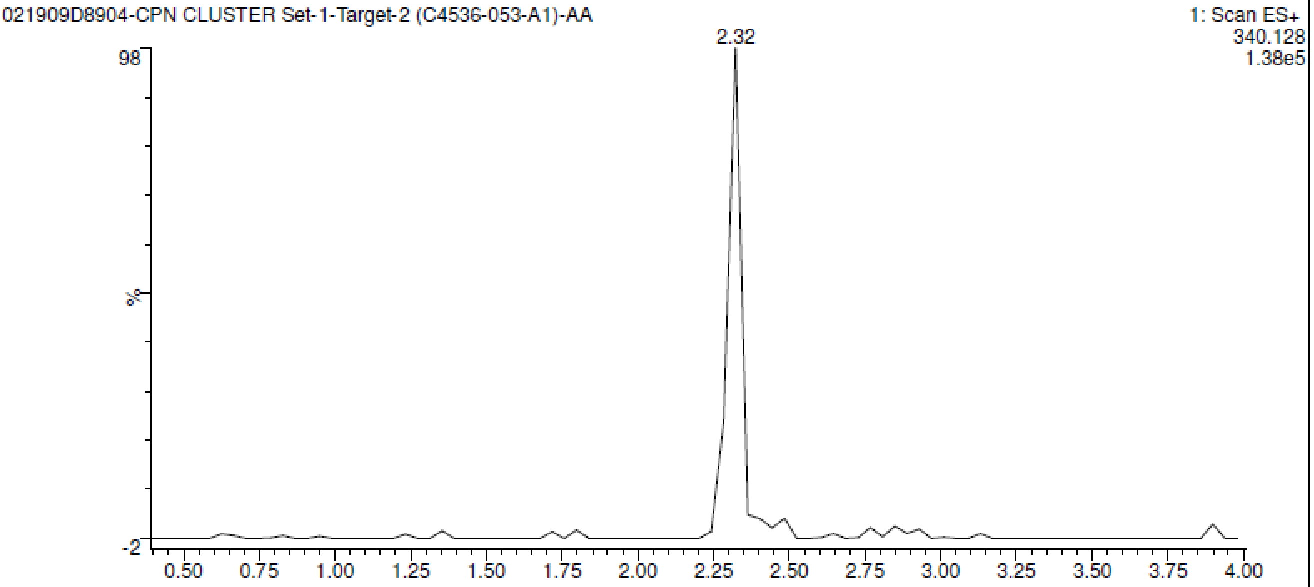

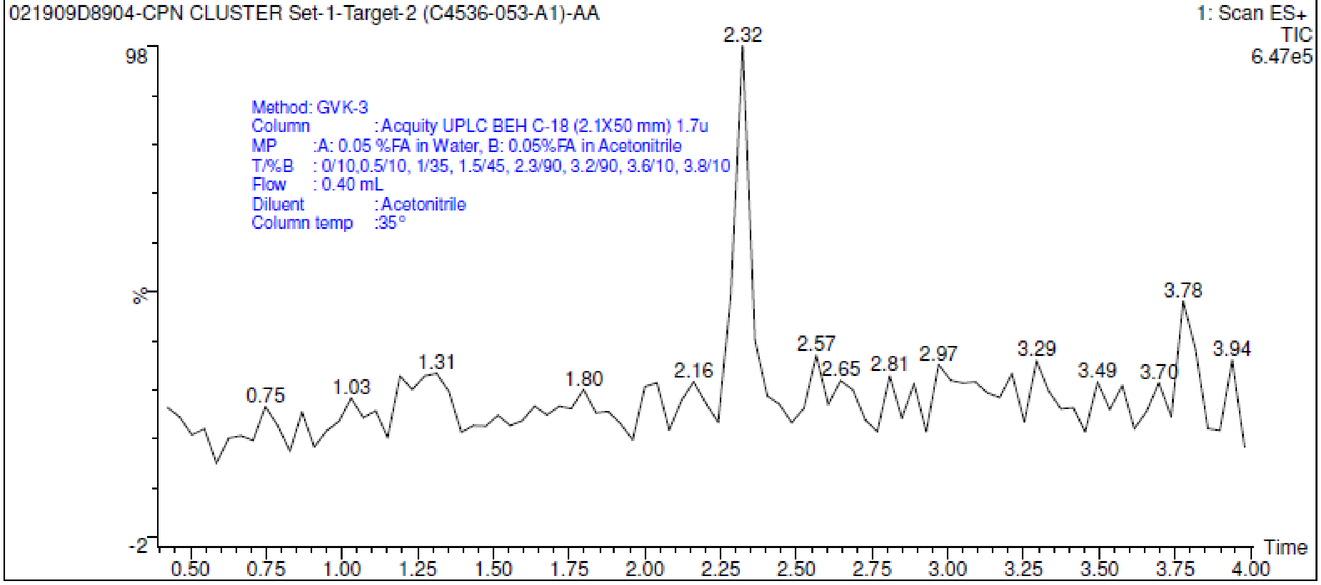

GVK Biosciences Private Limited
Discovery Chemistry-Analytical Services
================================

Sample ID: CPN CLUSTER Set-1-Target-2 (C4536-053-A1) **Date of analysis: 30-Sep-2019:23:02:28**
Acq Method :GVK-3 **Instrument ID:ANL-MCL2-LCMS-001**
2:F,2

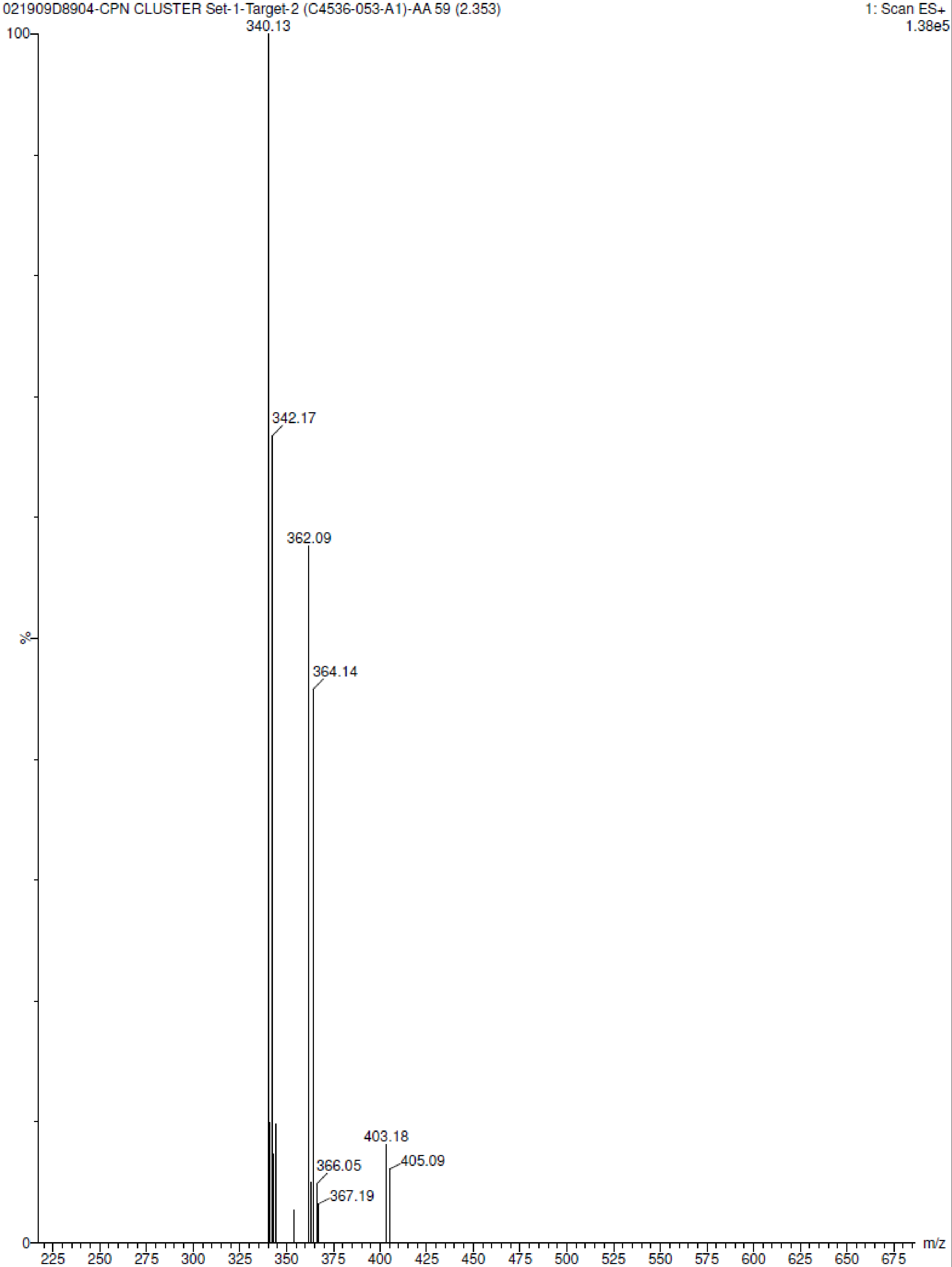

DCP_7 (16)

S.No	Test	Results
1	**Description**	Pale Yellow solid
2	**Identification**<br>(a) **NMR**<br>(b) **Mass by LC-MS**	 Complies to structure<br>281.23 $[M+H]^+$
3	**Chromatographic Purity by UPLC (Area %)**<br>Impurities>1.0%	99.66<br>Nil
4	**Chromatographic Purity by LCMS (Area %)**<br>Impurities>1.0%	98.82<br>Nil

Remarks:

1. Traces of Aliphatic impurities observed in NMR spectrum.
2. Traces of Acetonitrile observed in NMR spectrum (Approximately 0.34%).

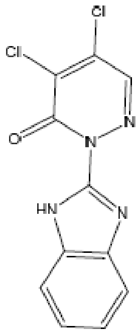

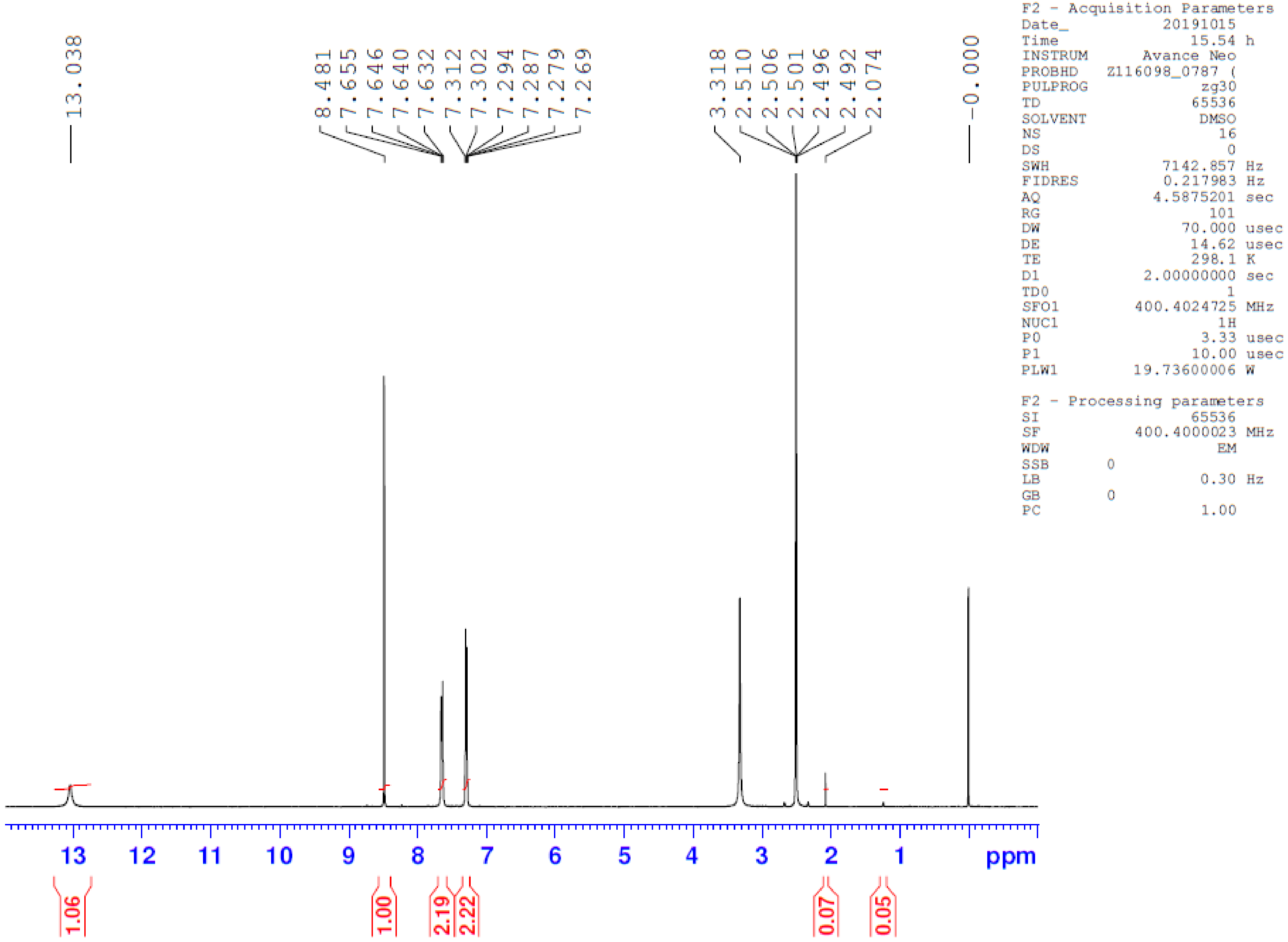
13.038
8.481
7.655
7.646
7.640
7.632
7.312
7.302
7.294
7.287
7.279
7.269
3.318
2.510
2.506
2.501
2.496
2.492
2.074
-0.000
F2 - Acquisition Parameters
Date_ 20191015
Time 15.54 h
INSTRUM Avance Neo
PROBHD Z116098_0787 (
PULPROG zg30
TD 65536
SOLVENT DMSO
NS 16
DS 0
SWH 7142.857 Hz
FIDRES 0.217983 Hz
AQ 4.5875201 sec
RG 101
DW 70.000 usec
DE 14.62 usec
TE 298.1 K
D1 2.00000000 sec
TD0 1
SFO1 400.4024725 MHz
NUC1 1H
P0 3.33 usec
P1 10.00 usec
PLW1 19.73600006 W
F2 - Processing parameters
SI 65536
SF 400.4000023 MHz
WDW EM
SSB 0
LB 0.30 Hz
GB 0
PC 1.00
13
12
11
10
9
8
7
6
5
4
3
2
1
ppm
1.06
1.00
2.19
2.22
0.07
0.05

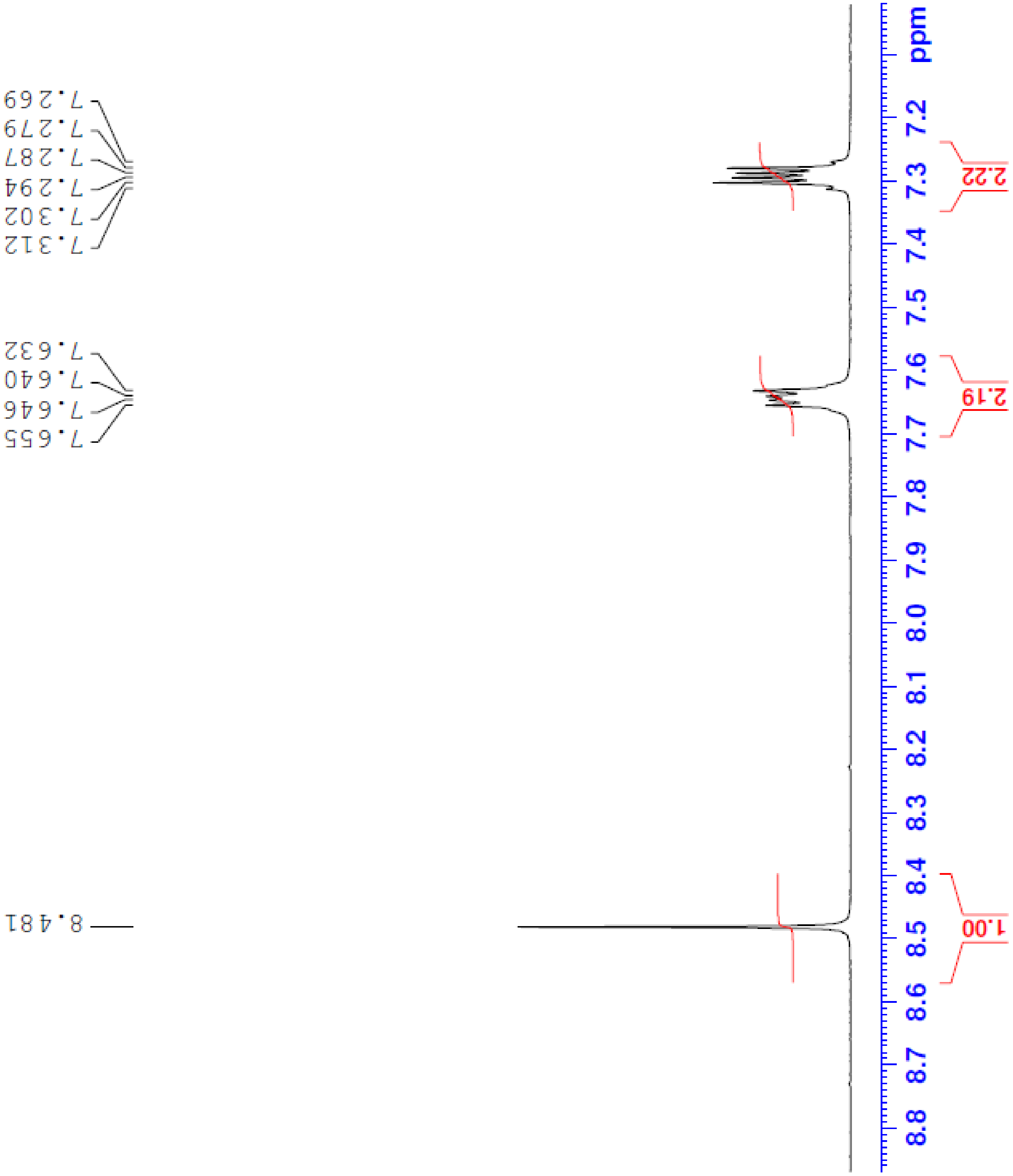
7.269
7.279
7.287
7.294
7.302
7.312
7.632
7.640
7.646
7.655
8.481
2.22
2.19
1.00
ppm
7.2
7.3
7.4
7.5
7.6
7.7
7.8
7.9
8.0
8.1
8.2
8.3
8.4
8.5
8.6
8.7
8.8

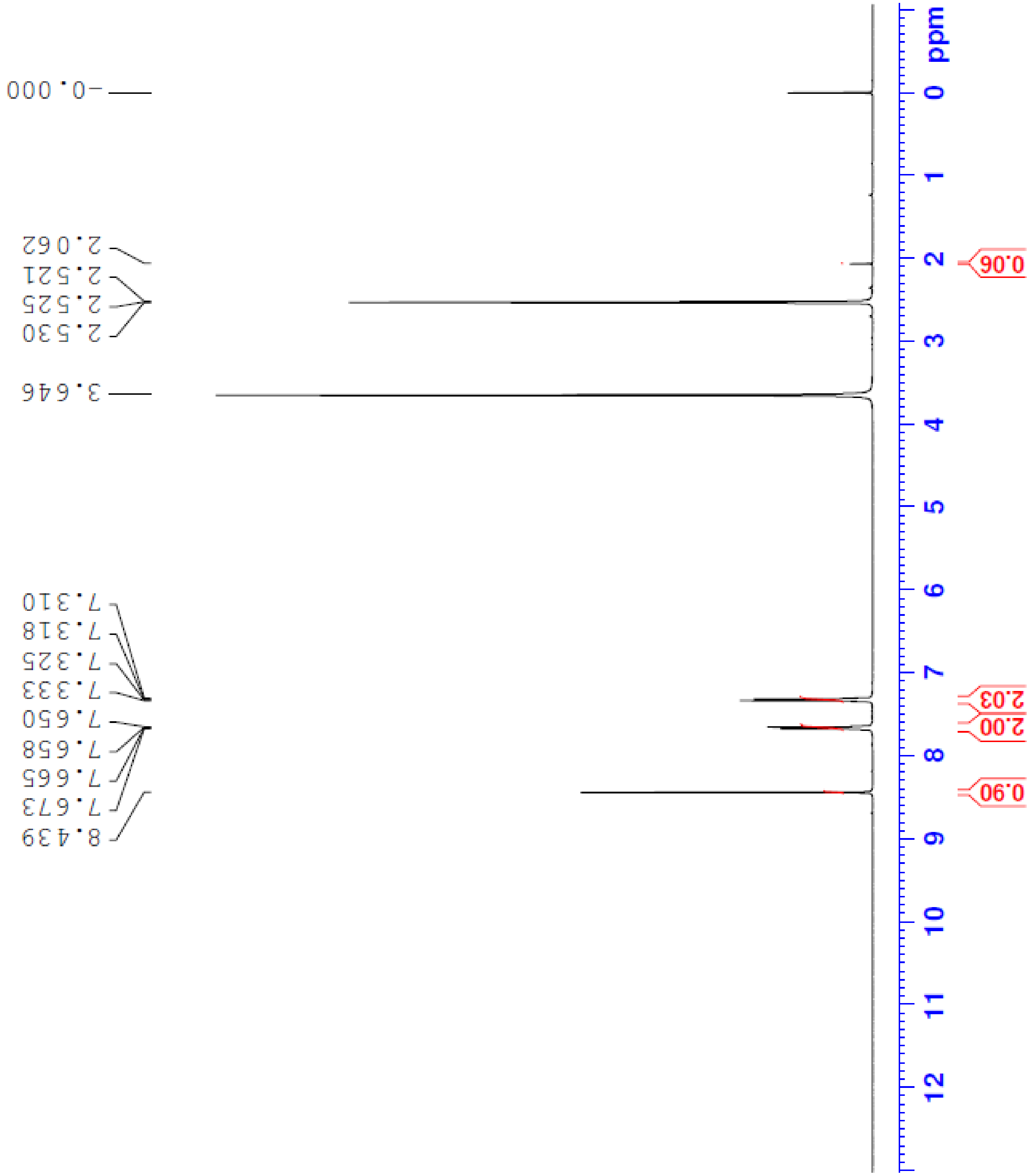

-0.000
2.062
2.521
2.525
2.530
3.646
7.310
7.318
7.325
7.333
7.650
7.658
7.665
7.673
8.439
0.06
2.03
2.00
0.90
ppm
0
1
2
3
4
5
6
7
8
9
10
11
12

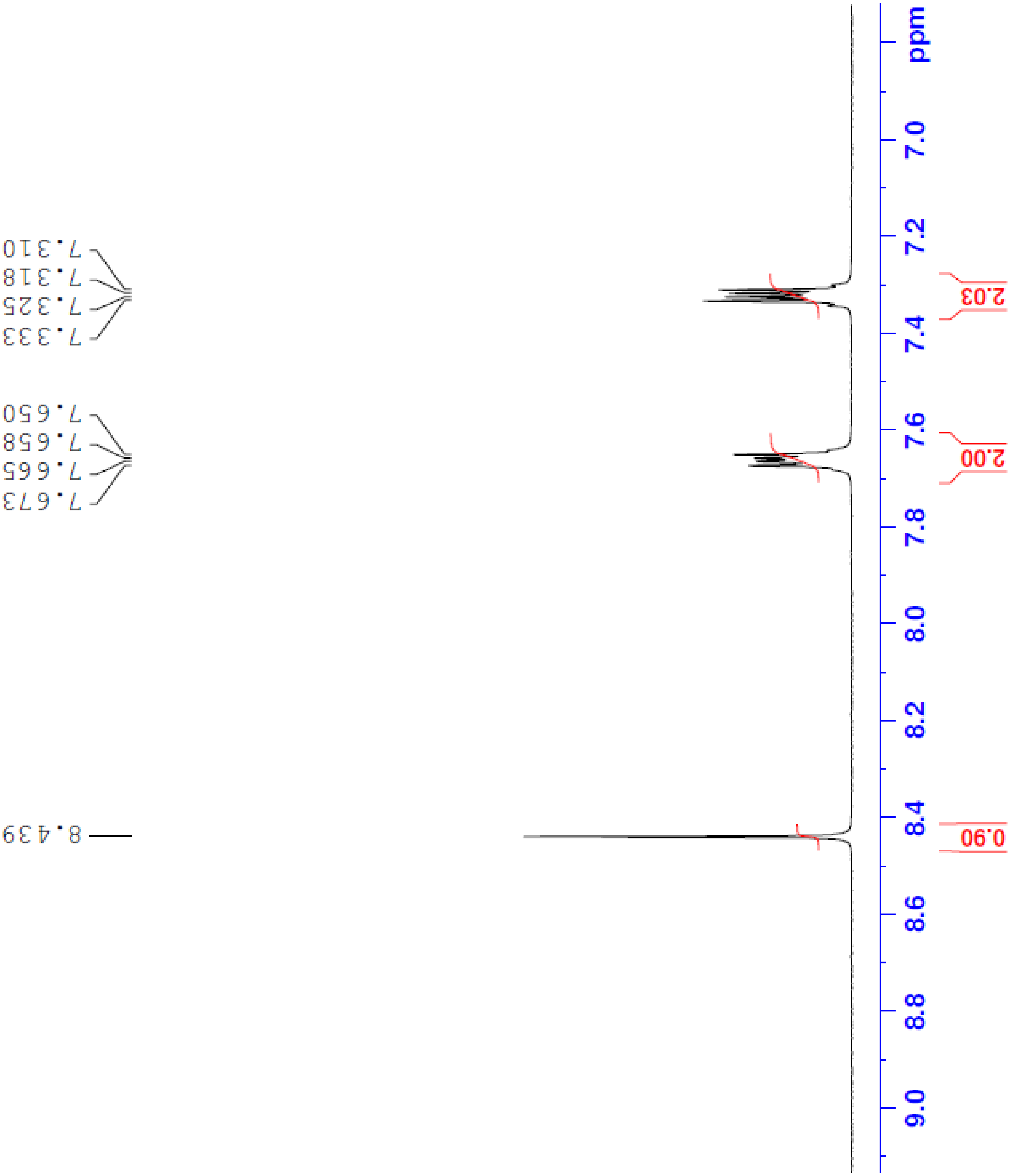

7.310
7.318
7.325
7.333
7.650
7.658
7.665
7.673
8.439
2.03
2.00
0.90
ppm
7.0
7.2
7.4
7.6
7.8
8.0
8.2
8.4
8.6
8.8
9.0

UPLC Method Conditions :

Column : Acquity UPLC BEH C18 (2.1x100) mm, 1.7µm
Mobile Phase-A : 0.05% TFA in Water
Mobile Phase-B : 0.05% TFA in Acetonitrile
Gradient (T/% B) : 0/10,4/90,6/90,6.1/10
Flow Rate : 0.3 mL/min
Temparature : 40℃
Diluent : ACN+WATER

Auto-Scaled Chromatogram

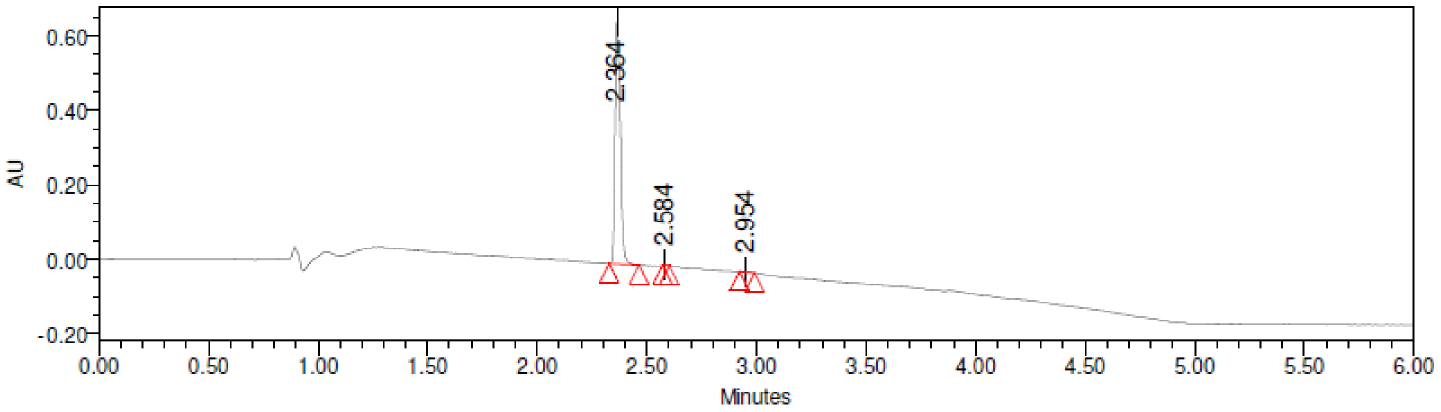

Peak Results

	RT	Area	Height	% Area
1	2.364	1096986	649988	99.66
2	2.584	1956	1630	0.18
3	2.954	1792	1063	0.16

GVK BIO Sciences Private Limited
Discovery Chemistry-Analytical Services
==

Sample ID :CPN CLUSTER Set-1-Target-4 (C4236-155-A1) **Date of analysis : 16-Oct-2019/16:15:50**
Acq Method :ATR-1 **Instrument ID: ANL-MCL2-LCMS-001**
1:E,1

021910B3532-CPN CLUSTER Set-1-Target-4 (C4236-155-A1)-C Sm (Mn, 5x5)

5: Diode Array
210
Range: 1.31

Time	Height	Area	Area%
1.98	1215410	49154.80	98.82
2.12	3293	136.79	0.28
2.49	6521	223.45	0.45
3.05	5313	224.68	0.45

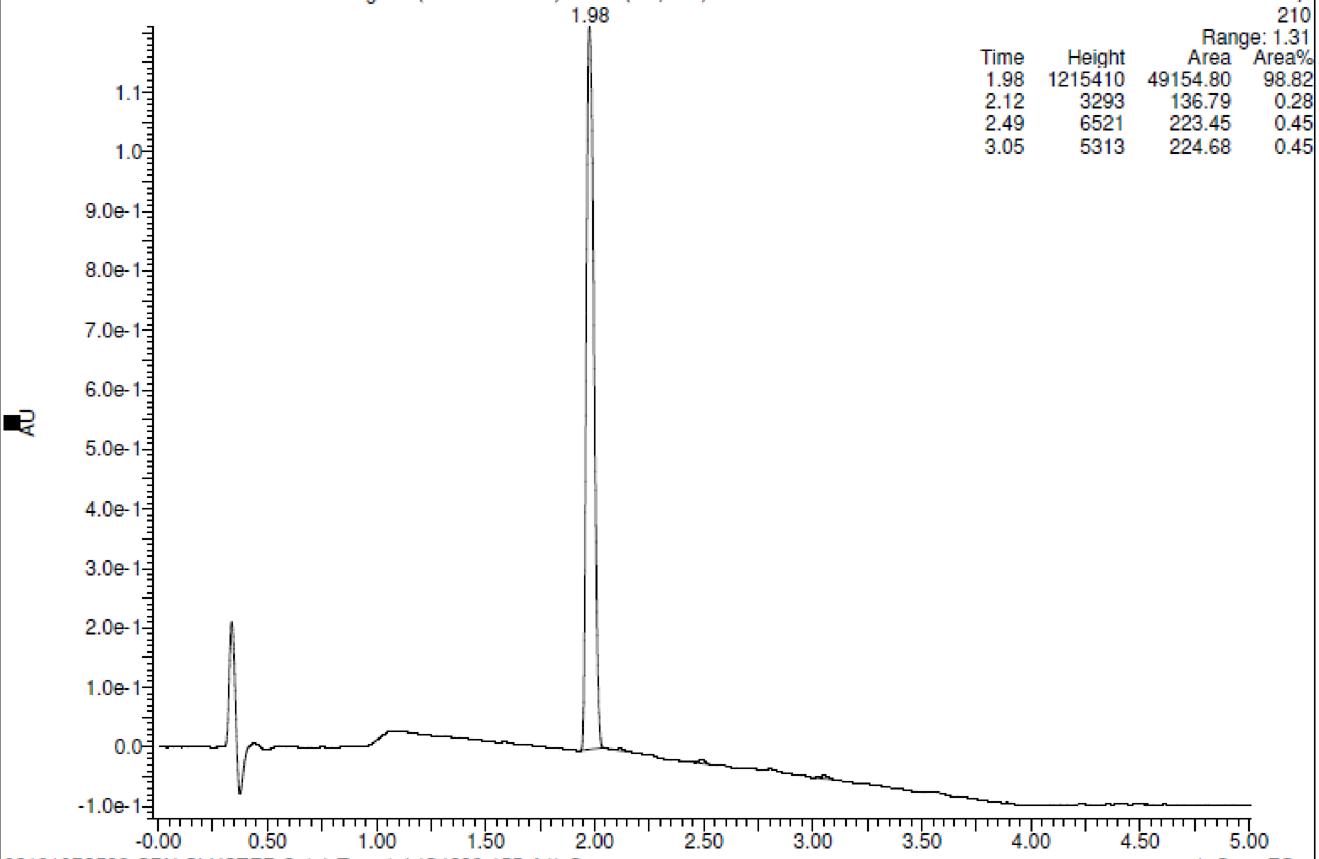

021910B3532-CPN CLUSTER Set-1-Target-4 (C4236-155-A1)-C

1: Scan ES+
TIC
2.48e7

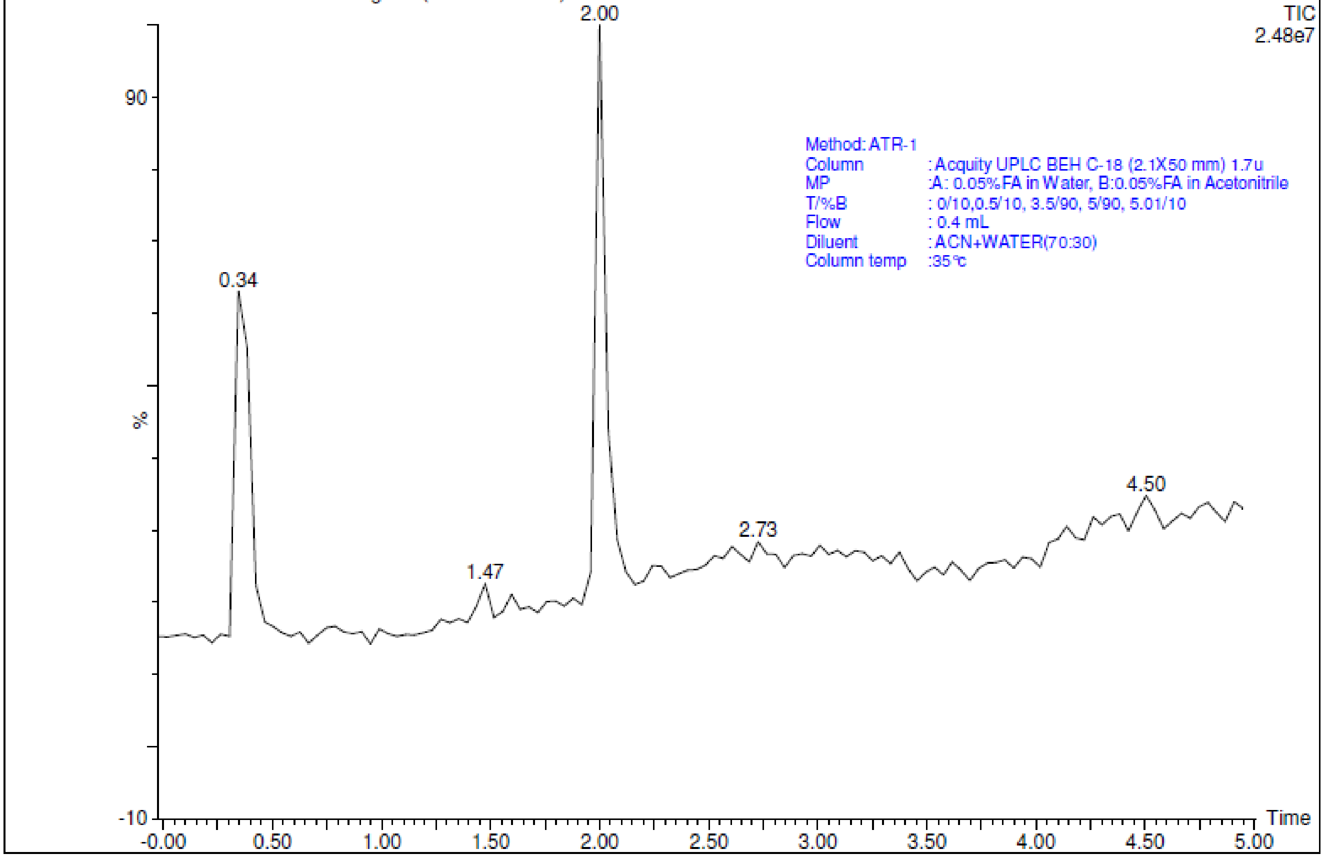

GVK Biosciences Private Limited
Discovery Chemistry-Analytical Services
================================

Sample ID: CPN CLUSTER Set-1-Target-4 (C4236-155-A1) **Date of analysis: 16-Oct-2019:16:15:50**
Acq Method :ATR-1 **Instrument ID:ANL-MCL2-LCMS-001**
1:E,1

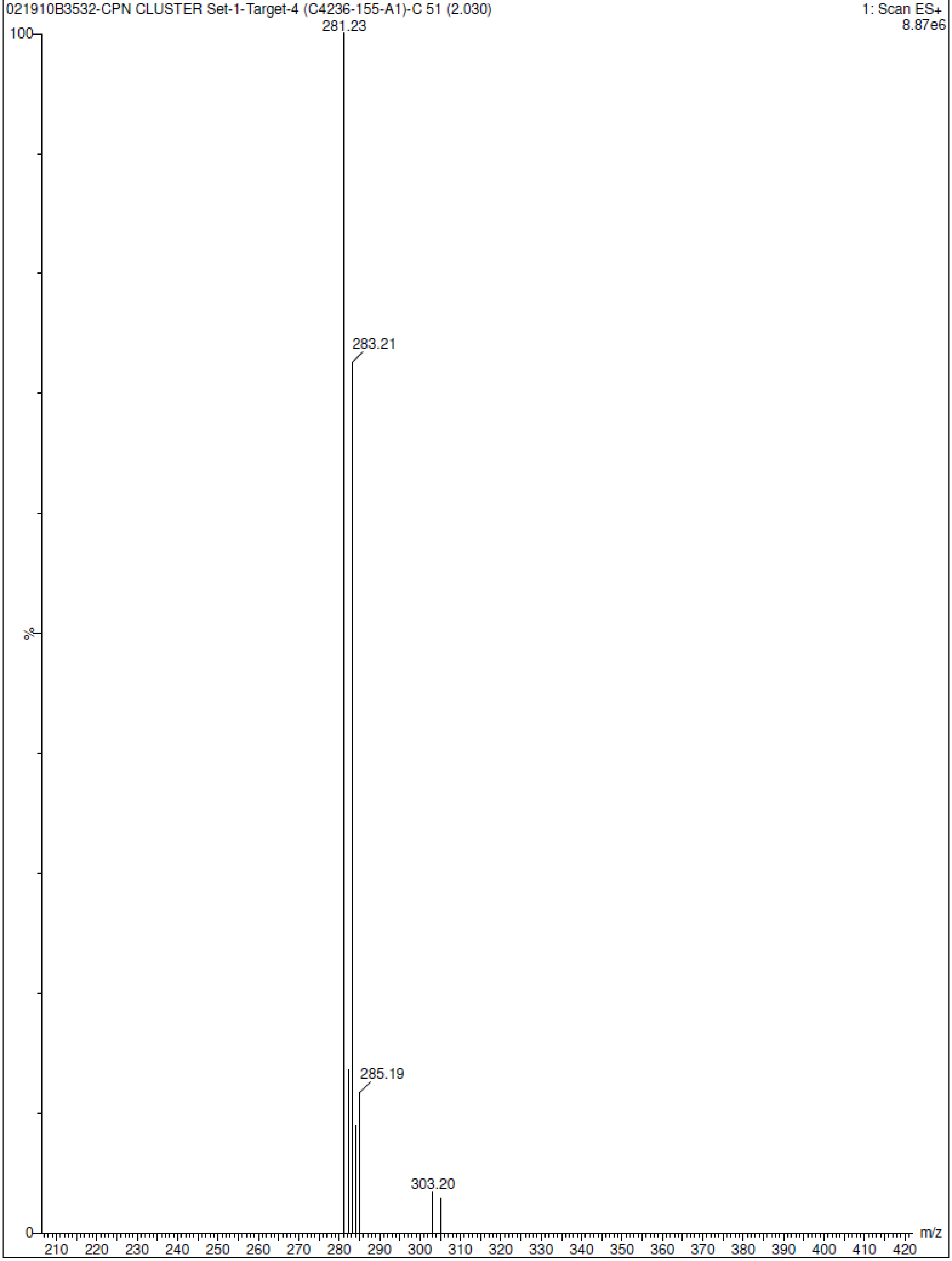

Supplement: Supplementary file 1 — Supplementary Information 1. [file 41598_2022_8209_MOESM1_ESM.pdf]
