# Supplementary material for: A combination screening to identify enhancers of para-aminosalicylic acid against Mycobacterium tuberculosis
Source: Sci Rep. 2022 Apr 4;12:5635. doi: 10.1038/s41598-022-08209-w (PMC8980009; doi:10.1038/s41598-022-08209-w)
Supplement: Supplementary file 2 — Supplementary Information 2. [file 41598_2022_8209_MOESM2_ESM.pdf]

A combination screening to identify enhancers of *para*-aminosalicylic acid against *Mycobacterium tuberculosis*

Supplementary materials

Jinyeong Heo[1], Dahae Koh[1], Minjeong Woo[2], Doyoon Kwon[2], Virginia Carla de Almeida Falcão[2], Connor Wood[2], Honggun Lee[1], Kideok Kim[1], Inhee Choi[3], Jichan Jang[4], Priscille Brodin[5], David Shum[1] and Vincent Delorme[2]#

1. Institut Pasteur Korea, Screening Discovery Platform, Seongnam, Gyeonggi, 13488 Republic of Korea

2. Institut Pasteur Korea, Tuberculosis Research Laboratory, Seongnam, Gyeonggi, 13488 Republic of Korea

3. Institut Pasteur Korea, Medicinal Chemistry, Seongnam, Gyeonggi, 13488 Republic of Korea

4. Molecular Mechanisms of Antibiotics, Division of Life Science, Research Institute of Life Science, Division of Applied Life Science (BK21plus Program), Gyeongsang National University, Jinju, 52828 Republic of Korea

5. University of Lille, CNRS, INSERM, CHU Lille, Institut Pasteur de Lille, U1019 - UMR 9017 - CIIL - Center for Infection and Immunity of Lille, Lille, France

# Address correspondence to: vincent.delorme@ip-korea.org.

Contents:

- Supplementary figures S1-S20

A

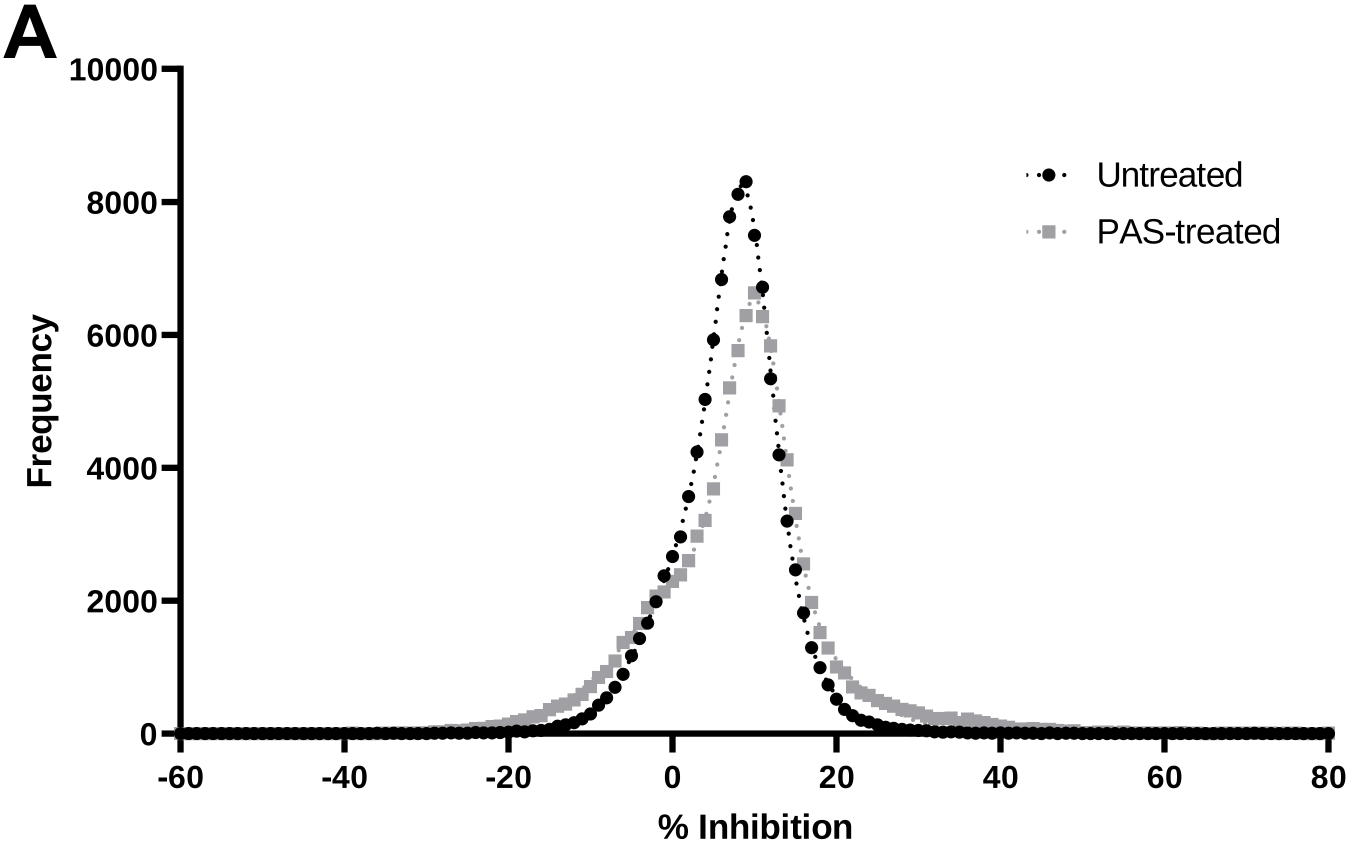

B

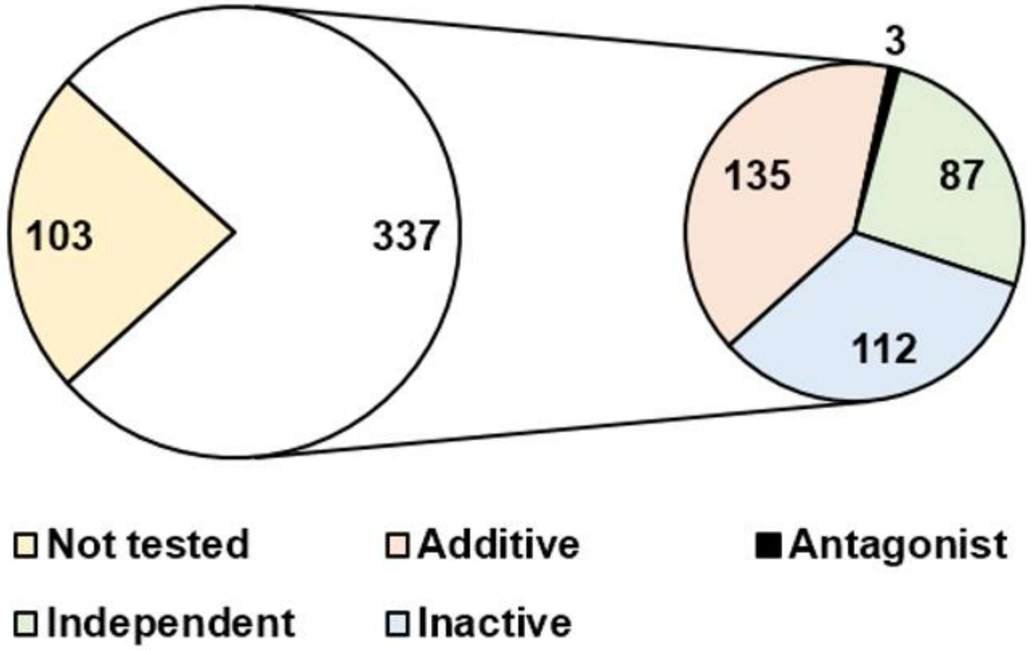

Figure S1. **Screening assay results**. **A**. Frequency distribution of the compound inhibition data for both sets (PAS-treated, untreated). The histogram was built from the data using a binning of 1, ranging from -100 to 100% inhibition. Best fits against a sum of two Gaussian distribution model, obtained by non-linear least square regression, are shown as dashed lines. Best fit parameters (Amplitude; Mean; SD) were [(3623; 6.025; 7.258), (4844; 8.776; 3.134)] for Untreated (R^2 = 0.9997) and [(2644; 6.081; 10.33), (4090; 10.26; 3.269)] for PAS-treated (R^2 = 0.9986). **B**. Number of hits falling in each category following activity confirmation by dose-response with cherry-picked compounds.

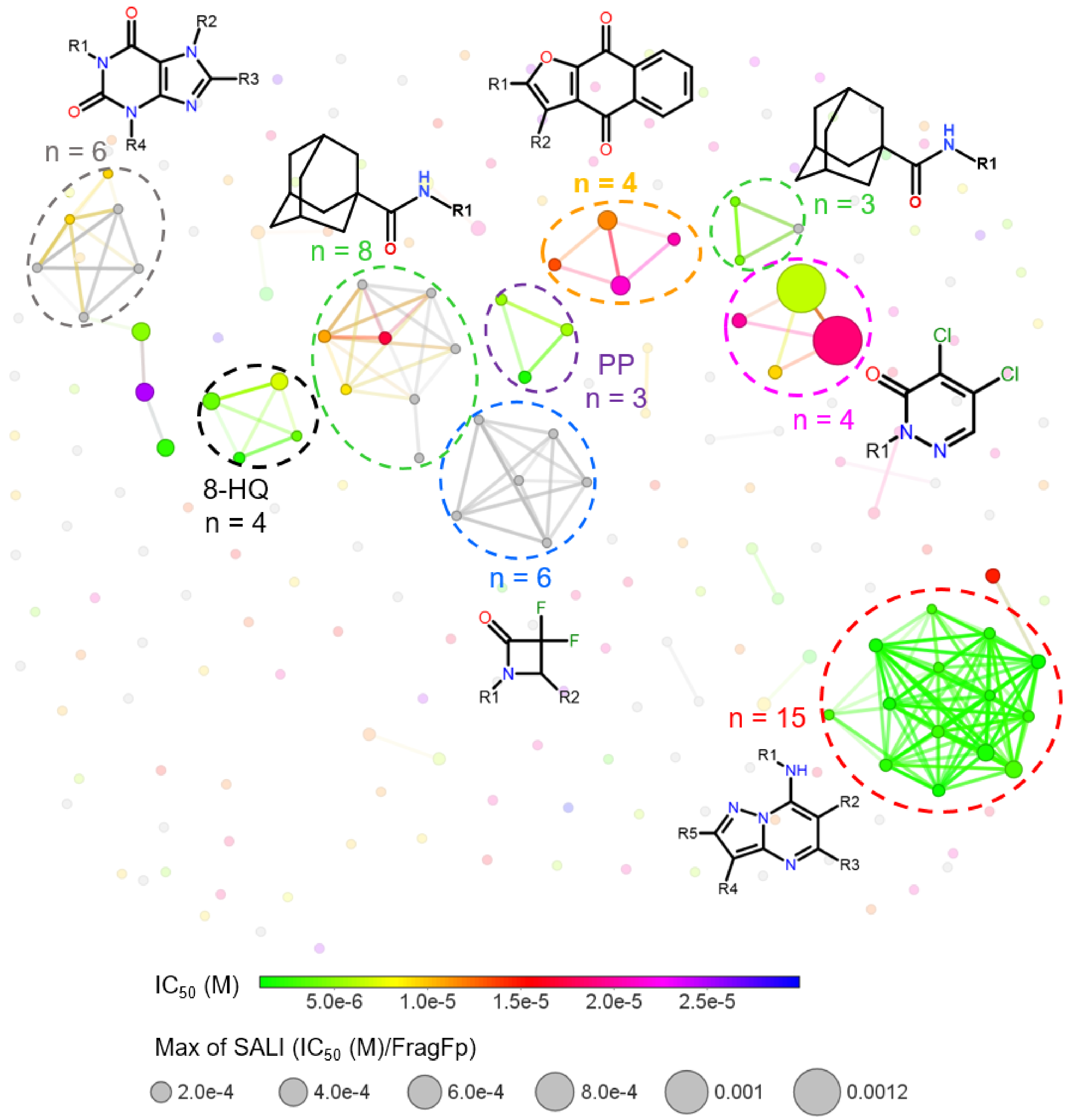

Figure S2. **Clustering of the hits active without PAS**. Similarity analysis of the 225 validated hits, using IC_{50} values in absence of PAS for the color coding (non-clustered compounds were faded for clarity). Structure Activity Landscape Index (SALI) plots were computed by DataWarrior v5.5.0, using the FragFp descriptor as similarity criterion. Clusters are circled in a dashed line with distinct colors (note that for the adamantyl amide cluster, 2 groups of 8 and 3 molecules respectively are present). Core chemical structure for each cluster is indicated, together with the number of molecules within the cluster (n). 8-HQ: 8-hydroxyquinoline. PP: pyrido[1,2-a]pyrimidin-4-one.

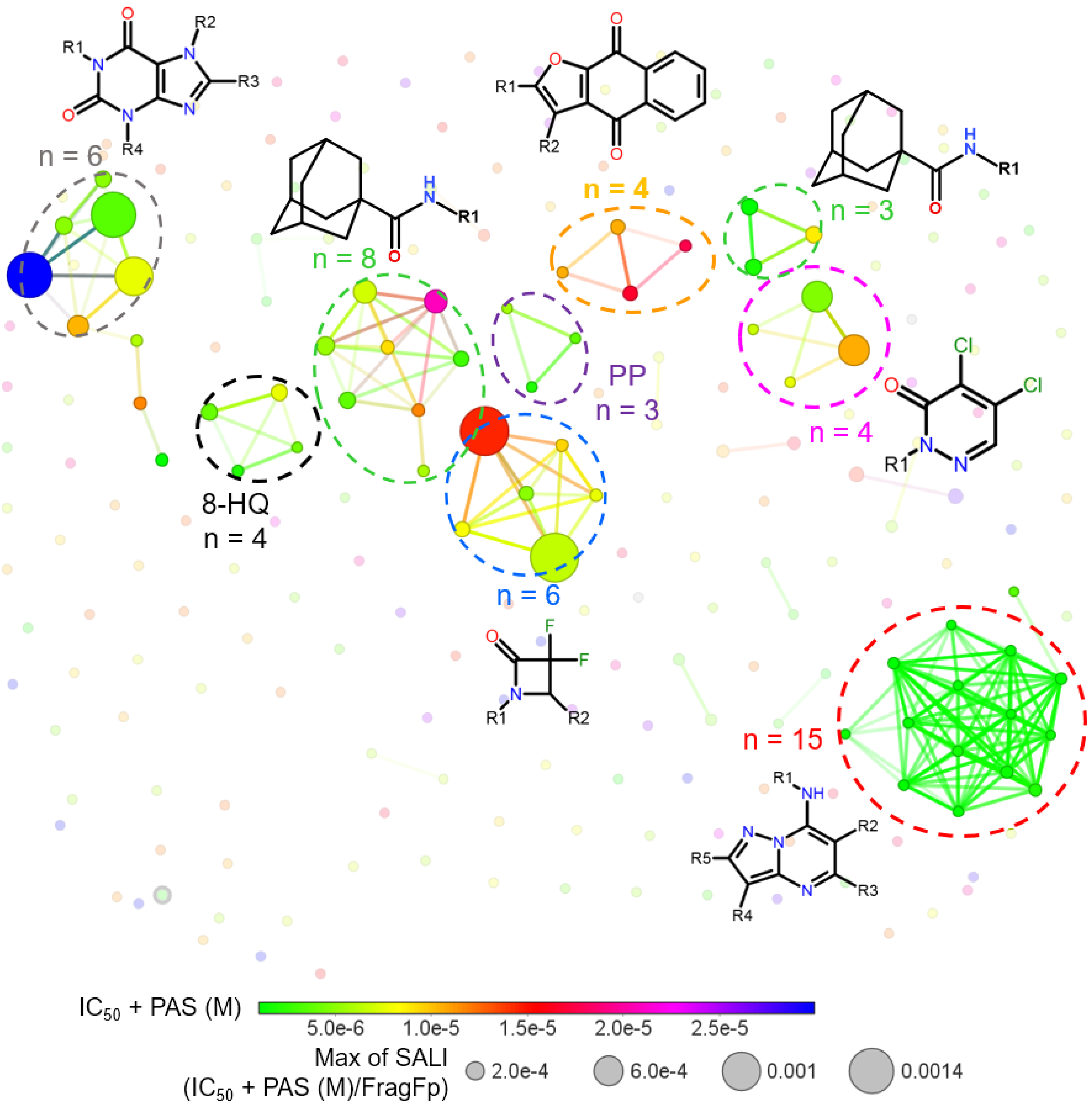

Figure S3. **Clustering of the hits active with PAS**. Similarity analysis of the 225 validated hits, using IC_{50} values in presence of PAS for the color coding (non-clustered compounds were faded for clarity). Structure Activity Landscape Index (SALI) plots were computed by DataWarrior v5.5.0, using the FragFp descriptor as similarity criterion. Clusters are circled in a dashed line with distinct colors (note that for the adamantyl amide cluster, 2 groups of 8 and 3 molecules respectively are present). Core chemical structure for each cluster is indicated, together with the number of molecules within the cluster (n). 8-HQ: 8-hydroxyquinoline. PP: pyrido[1,2-a]pyrimidin-4-one.

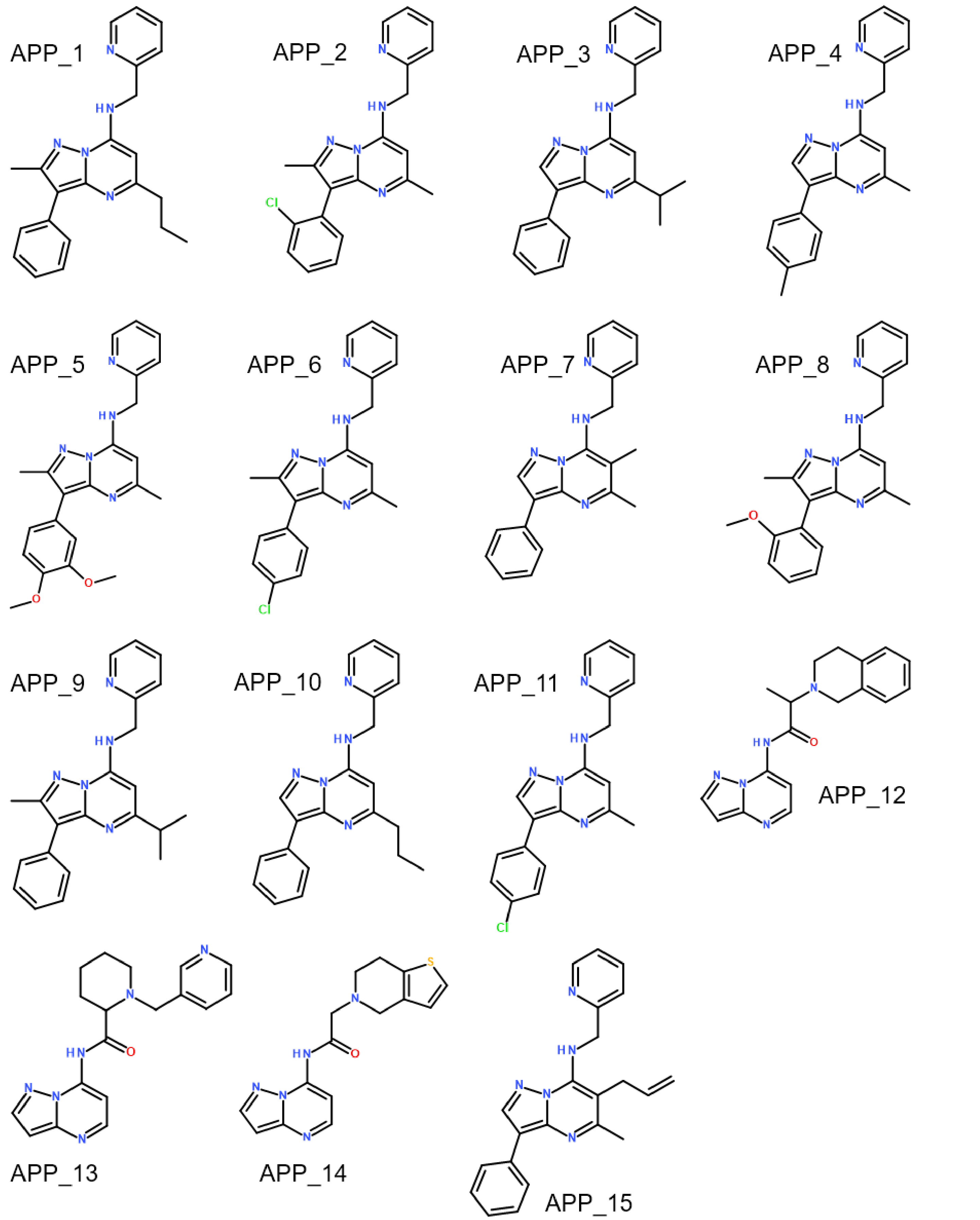

Figure S4. **Structure of the 15 aminopyrazolo[1,5-*a*]pyrimidine (APP) derivatives identified as hits in the screening**.

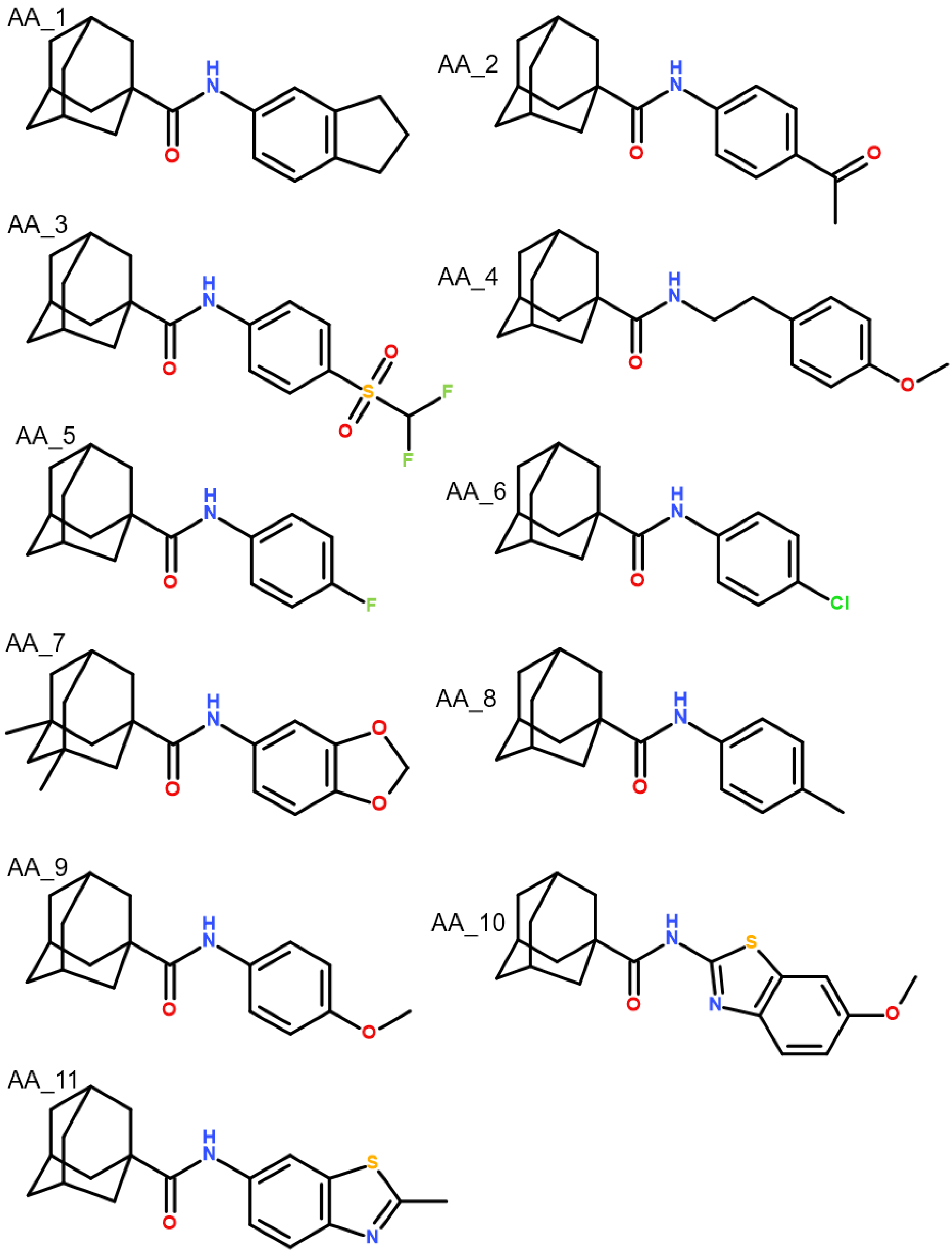

Figure S5. Structure of the 12 adamantyl amide (AA) derivatives identified as hits in the screening.

A

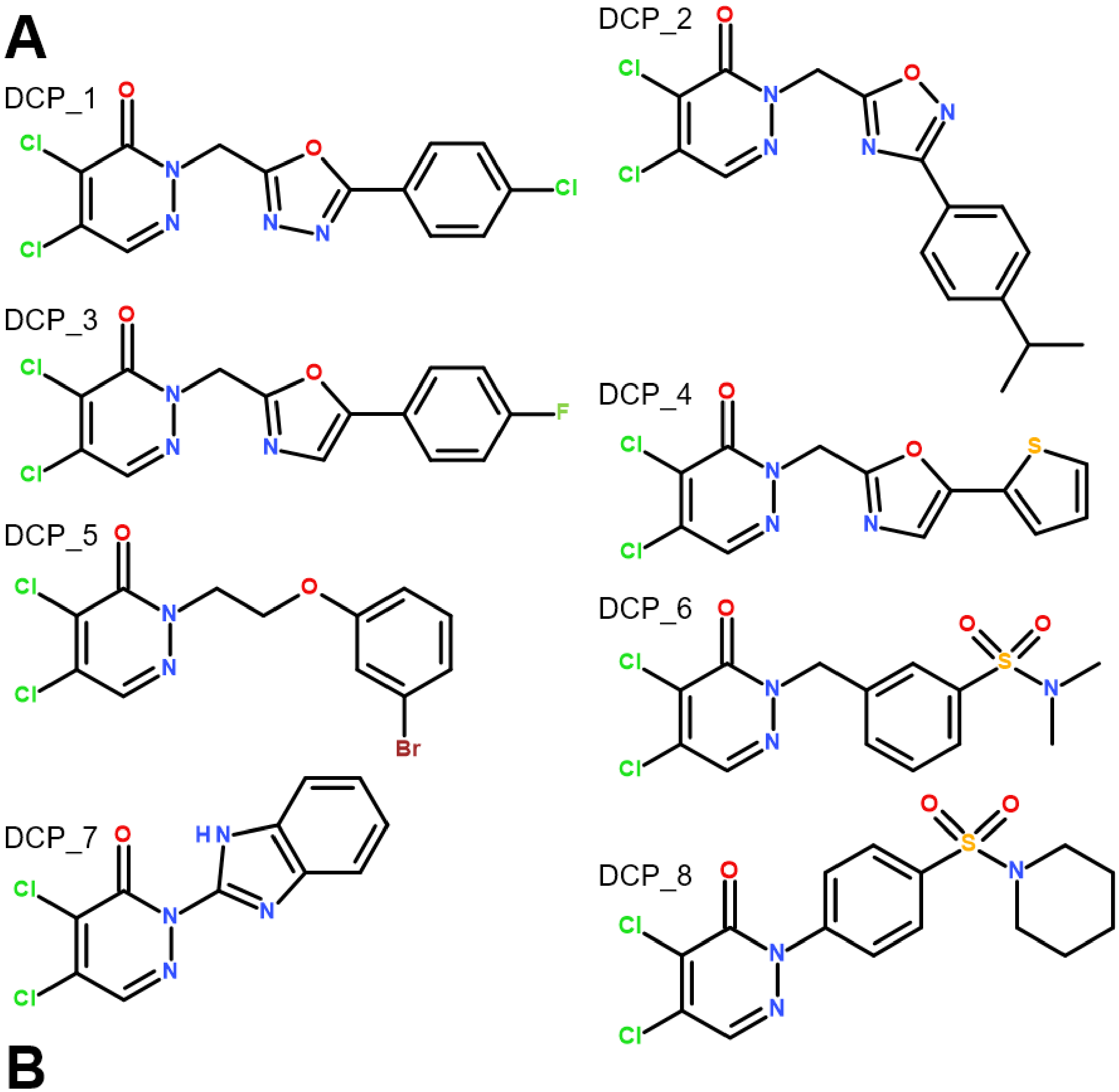

B

Compound	1ary screening (% Inhibition)		2ary screening IC_{50} (µM)		Re-synthesis IC_{50} (µM)		Ln(FC)	Category
	Untreated	*PAS-treated*	*Untreated*	*PAS-treated*	*Untreated*	*PAS-treated*		
**DCP_3**	94.2	95.8	6.4	4.6	4.5	3.7	0.20	independent
**DCP_7**	99.0	98.7	8.5	3.3	1.8	2.4	-0.29	independent

Figure S6. **Data for the DCP cluster**. **A.** Structure of the 4,5-dichloro-2*H*-pyridazin-3-one (DCP) derivatives identified as hits in the screening. **B.** Activity profile of DCP_3 and DCP_7. IC_{50} values given are average for a duplicated dose-response from a single, representative experiment. The fold-change (FC) was calculated using the IC_{50} values obtained from re-synthesized compounds.

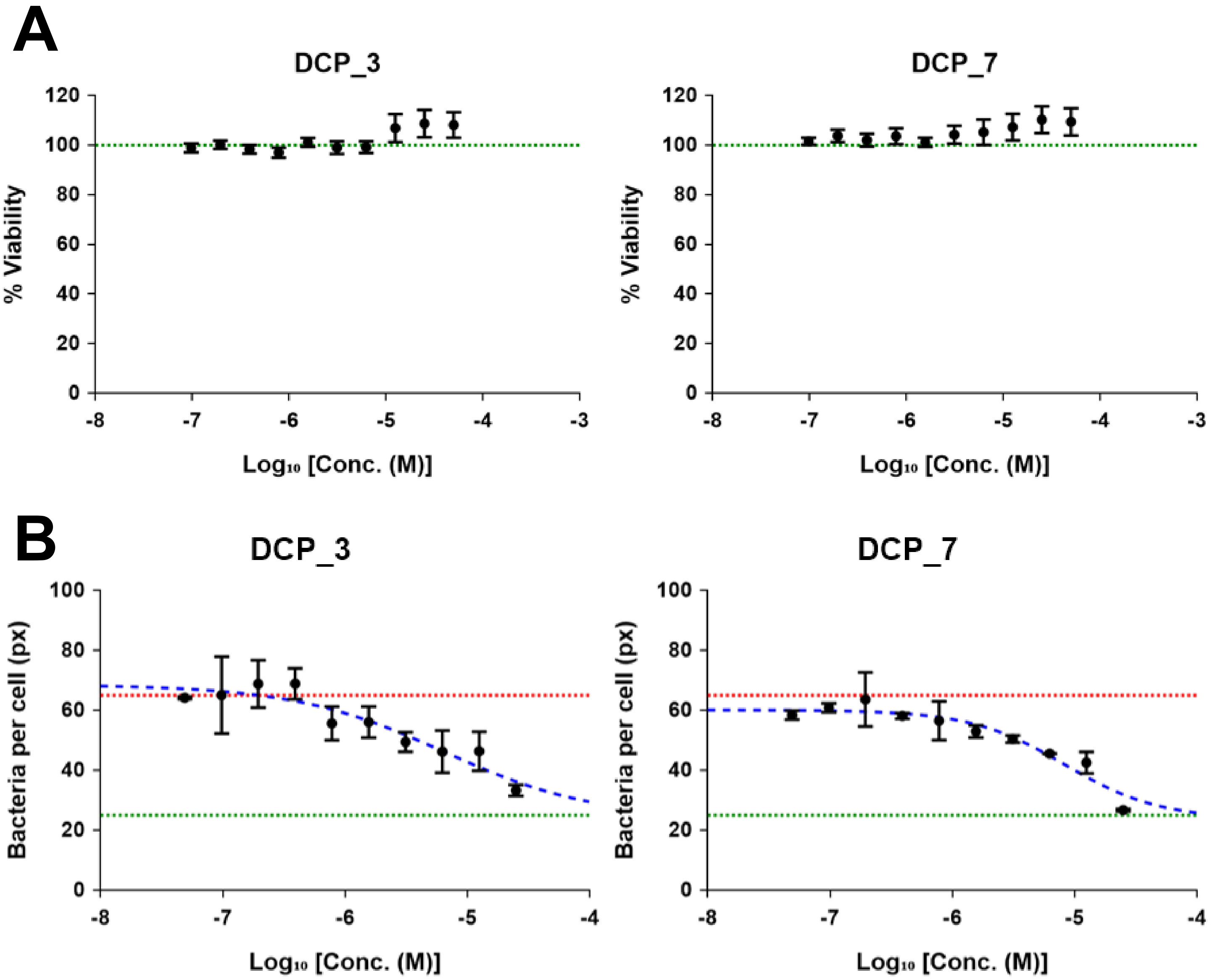

C

Compound	IC_{50} (µM)
**DCP_3**	9.2 ± 2.7
**DCP_7**	8.2 ± 3.6

Figure S7. Ability of DCP_3 and DCP_7 to restrict H37Rv-GFP growth in Raw264.7 macrophages. **A**. Cytotoxicity evaluation against Raw264.7 macrophages. See legend to Fig. S15 for experimental details. **B**. Dose-response curves showing the reduction in intracellular bacteria with increasing compound concentration. The red line indicates the average value obtained for the negative control (DMSO). The green line indicates the average value obtained for the positive control (rifampicin). Data shown are average and SD for a duplicated dose-response from a single, representative experiment. **C**. Summary of the IC_{50} values found for DCP_3 and DCP_7 using this intracellular assay. Values are average +/- SD for three biological replicates (each replicate was performed with a duplicated dose-response).

A

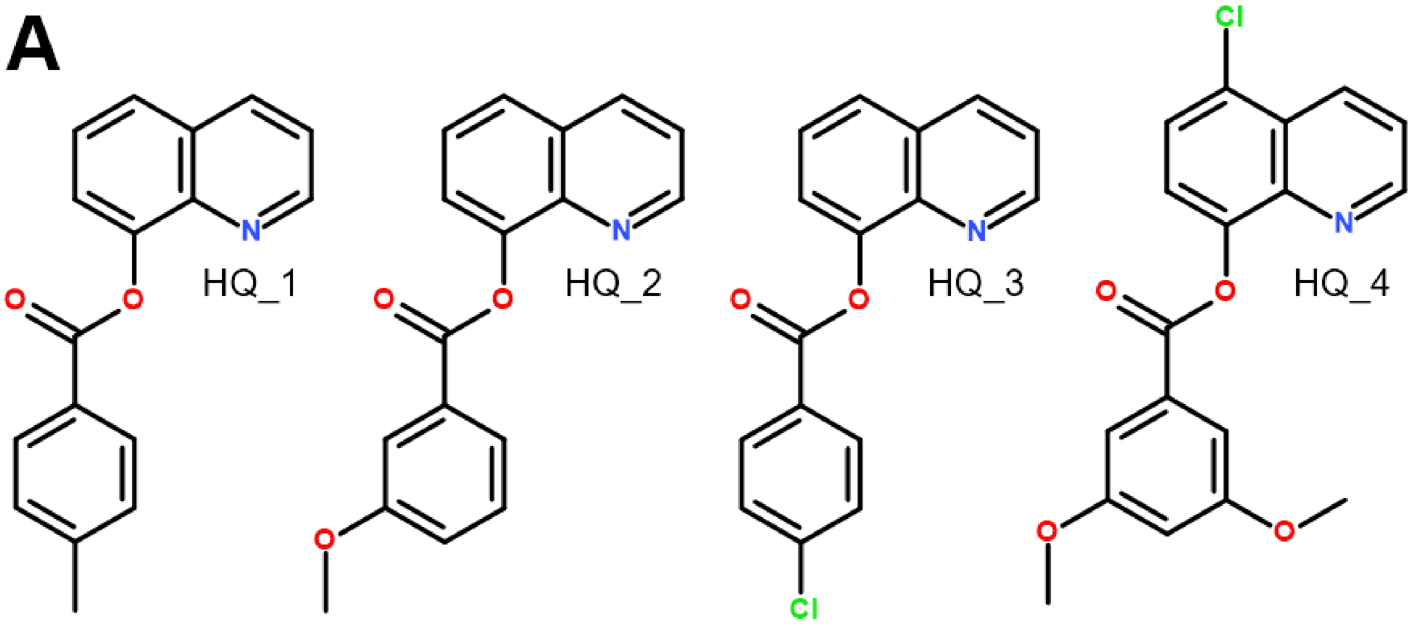

B

Compound	1^{ary} screening (% Inhibition)		2^{ary} screening IC_{50} (μM)		Ln(FC)	Category
	Untreated	*PAS-treated*	*Untreated*	*PAS-treated*		
**HQ_1**	104.9	99.1	4.0	3.9	0.03	independent
**HQ_2**	103.5	98.5	3.9	4.2	-0.07	Independent
**HQ_3**	102.1	97.5	7.1	7.7	-0.08	independent
**HQ_4**	100.4	100.7	2.0	2.0	0	independent

Figure S8. **Data for the 8-HQ cluster**. **A**. Chemical structure and **B**. activity profile of the 8-hydroxyquinoline (8-HQ) derivatives identified during the screening. IC_{50} values are from a single, duplicated dose-response.

A

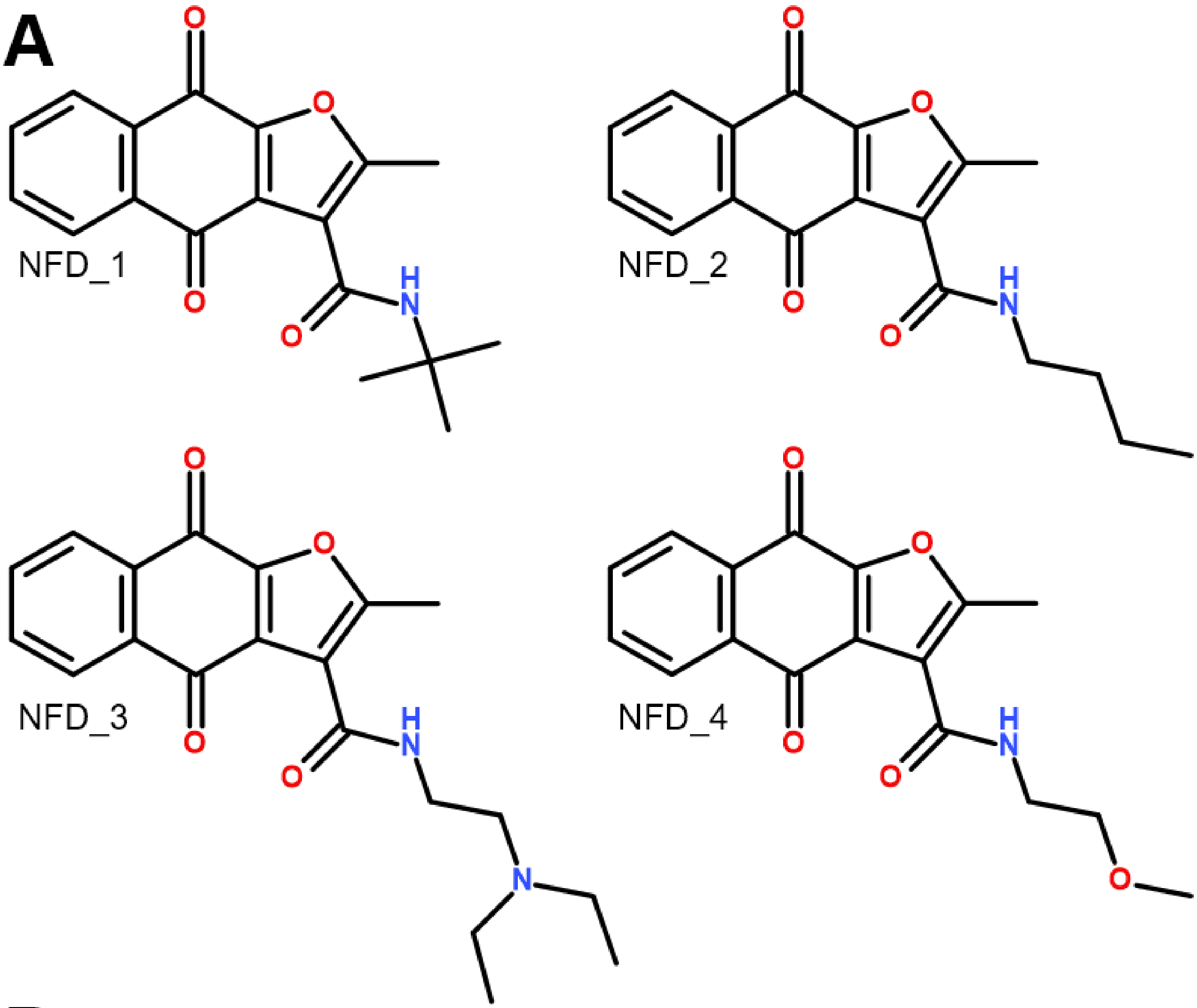

B

Compound	1ary screening (% Inhibition)		2ary screening IC_{50} (μM)		Ln(FC)	Category
	Untreated	*PAS-treated*	*Untreated*	*PAS-treated*		
**NFD_1**	29.3	58.8	20.3	17.7	0.14	independent
**NFD_2**	40.1	66.0	21.3	16.8	0.24	Independent
**NFD_3**	84.6	84.4	13.4	10.7	0.23	independent
**NFD_4**	76.4	77.9	11.8	10.5	0.12	independent

Figure S9. **Data for the NFD cluster**. **A**. Chemical structure and **B**. activity profile of the 4 naphtho[2,3-b]furan-4,9-dione (NFD) derivatives identified during the screening. IC_{50} values are from a single, duplicated dose-response.

A

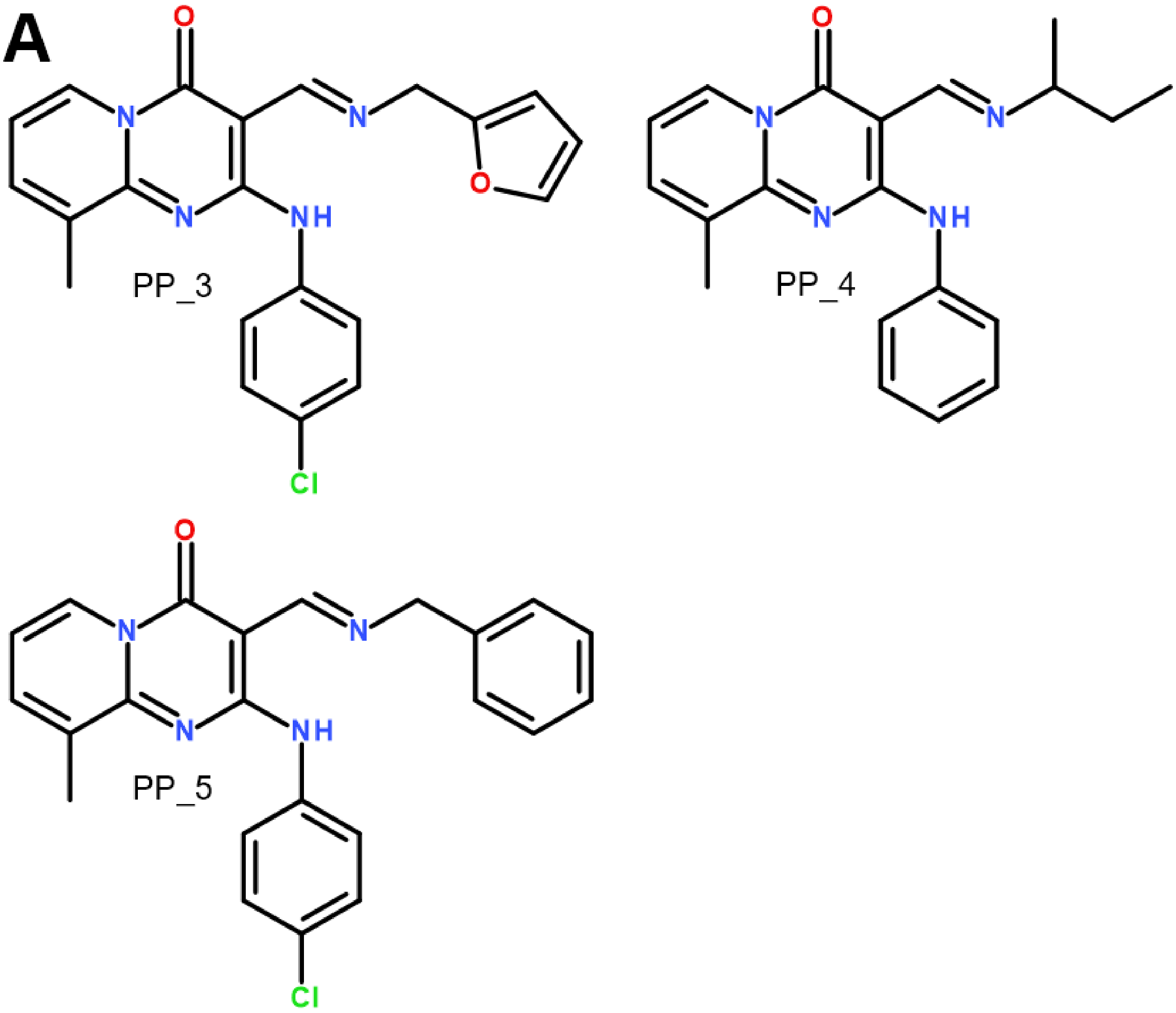

B

Compound	1ary screening (% Inhibition)		2ary screening IC_{50} (µM)		Ln(FC)	Category
	Untreated	*PAS-treated*	*Untreated*	*PAS-treated*		
**PP_3**	70.4	72.6	5.4	5.6	-0.04	independent
**PP_4**	89.2	88.7	1.8	1.9	-0.05	Independent
**PP_5**	72.0	88.4	5.7	3.7	0.43	independent

Figure S10. **Data for the PP cluster**. **A**. Chemical structure and **B**. activity profile of the 3 pyrido[1,2-a]pyrimidin-4-one (PP) derivatives identified during the screening. IC_{50} values are from a single, duplicated dose-response.

A

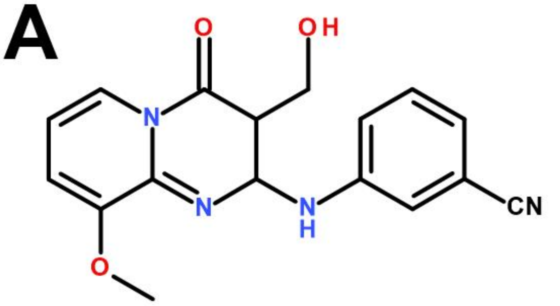

PP_2

IC_{50} (µM) = 0.23 ± 0.12

Mutant	IC_{50} (µM)	Mutation	
PP_2.1	> 25	DEL ($G^{830}T^{831}$)	Arg^{277}*shift*
PP_2.2	> 25	IN (T^{700})	Gln^{234}*shift*
PP_2.3	NT	SNP ($T^{350}C$)	$Leu^{117}Pro$
PP_2.4	NT	SNP ($C^{7}G$)	$Arg^{3}Gly$

B

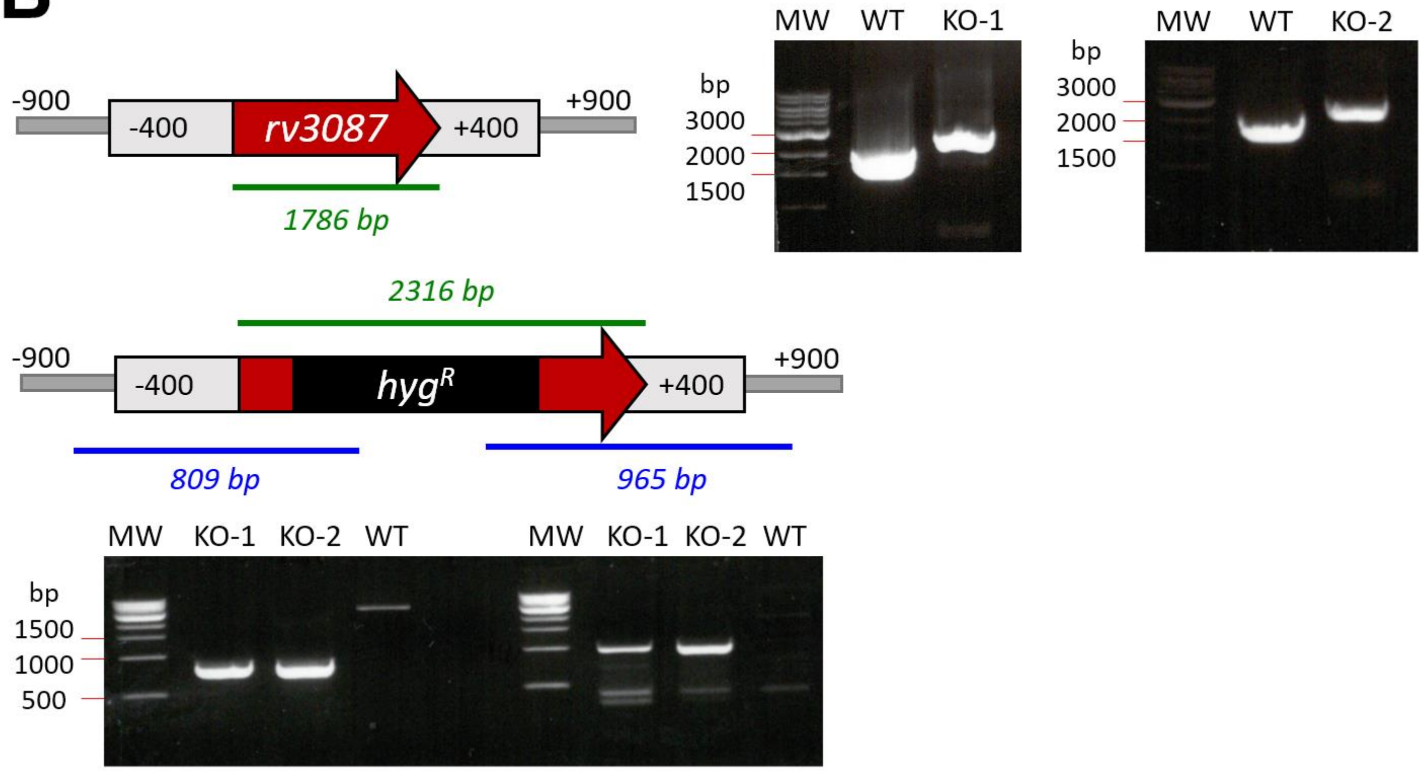

Figure S11. **Construction and validation of the rv3087 mutants**. **A**. *Left*, structure of the PP_2 derivative used for the generation of spontaneous resistant mutants. Activity value is the average +/- SD for three biological replicates (each replicate was performed with a duplicated dose-response). *Right*, summary of the mutations identified by whole genome sequencing and confirmed by Sanger sequencing. DEL, deletion; IN, insertion; SNP, single nucleotide polymorphism. Activities were determined by resazurin reduction assay. NT, activity not tested. **B**. Strategy used for the validation of *rv3087* gene disruption from the genome of two selected knock-out (KO) mutants (KO-1, KO-2), by PCR. A first set of primer was designed to amplify the whole *rv3087* gene (green), yielding a band of higher molecular weight (MW) in case of the KO, as compared to the wild-type (WT). Two sets of primers were also designed to amplify the 5' and 3' region of *rv3087* (blue), both with a hybridization site in the hygromycin resistance cassette (*hyg*^{R}), yielding bands of ~1 kb for both KO, but nothing for the WT. Full size and uncropped gels are shown on the next page.

Uncropped agarose gels of Figure S11

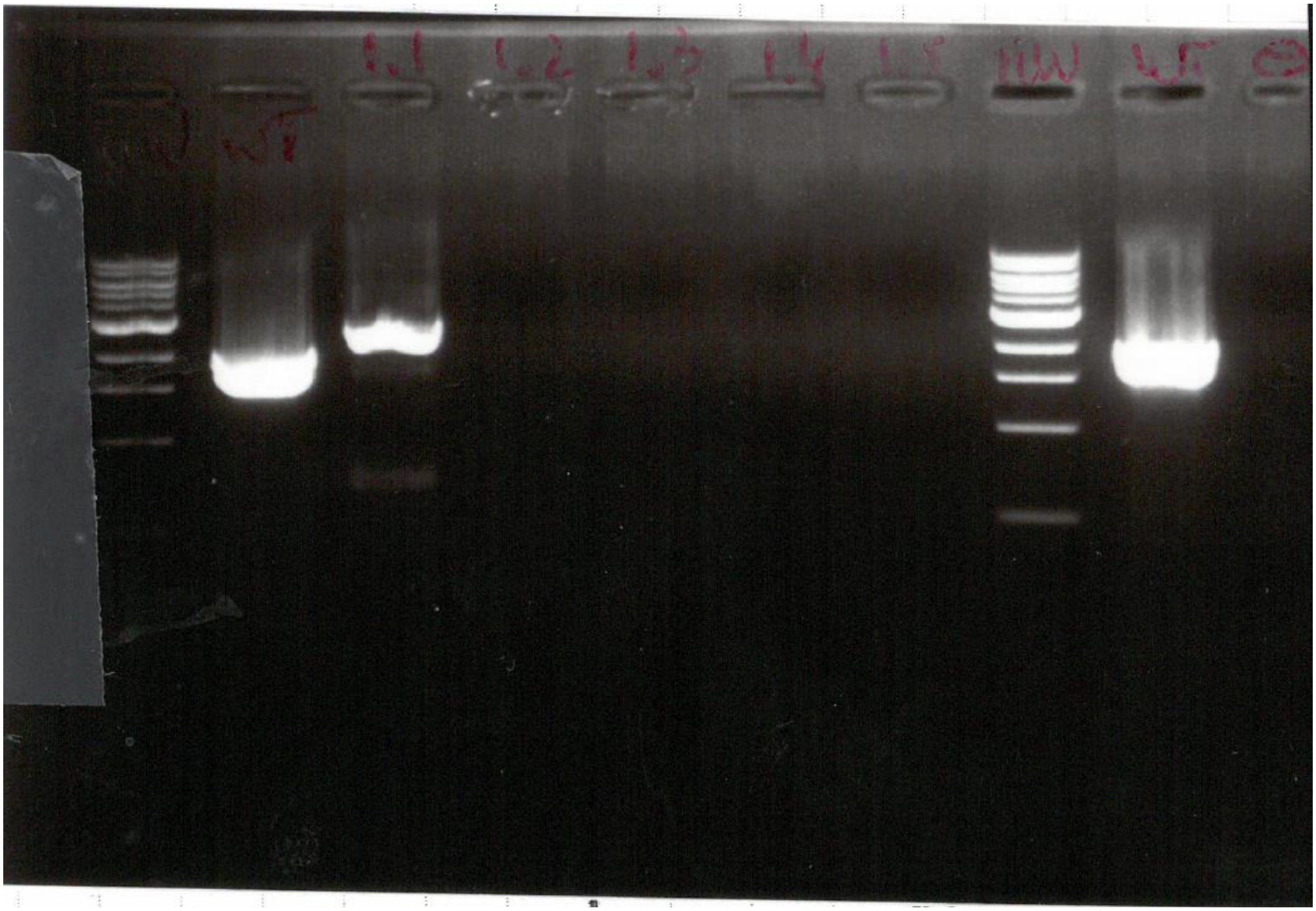

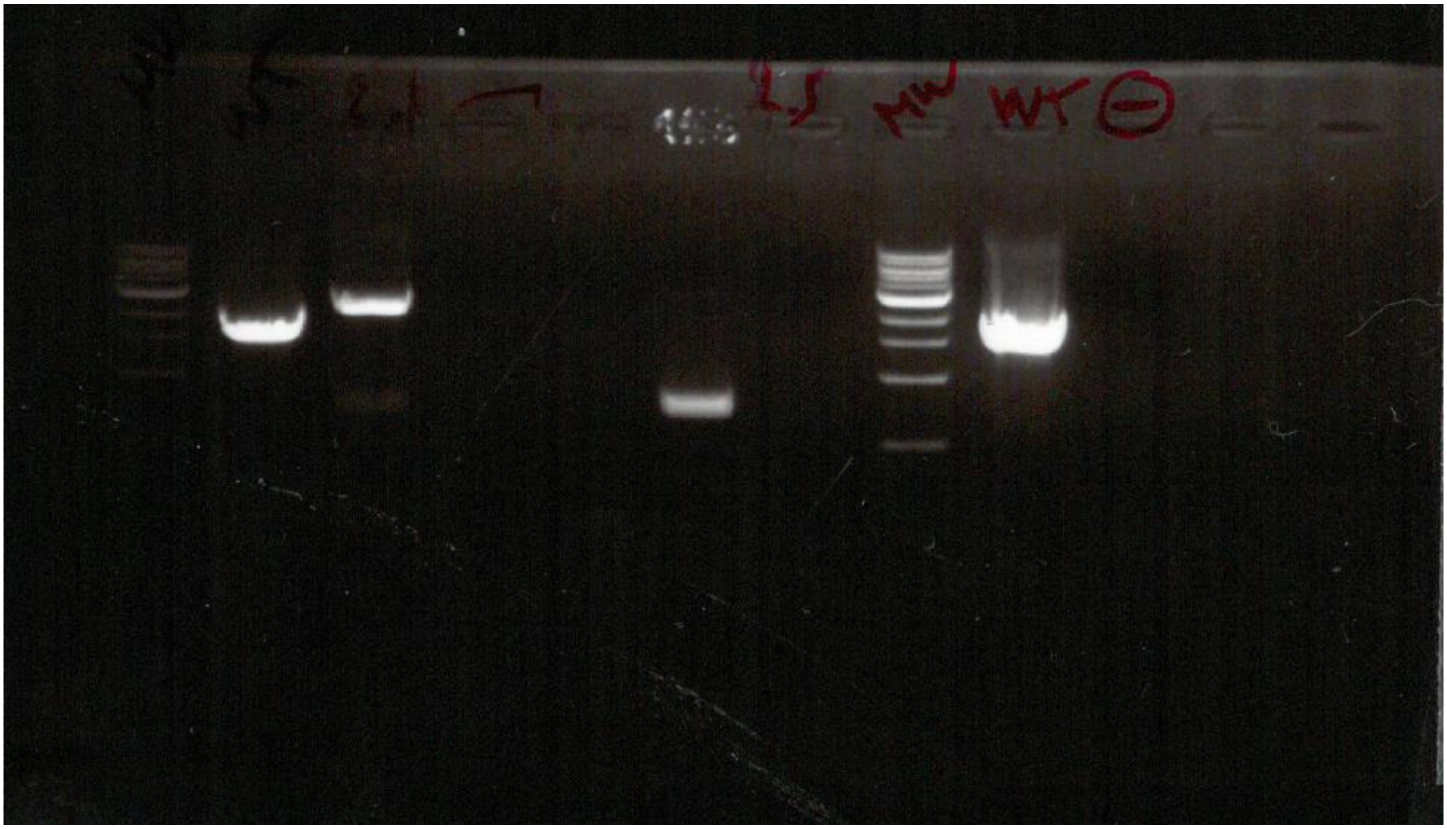

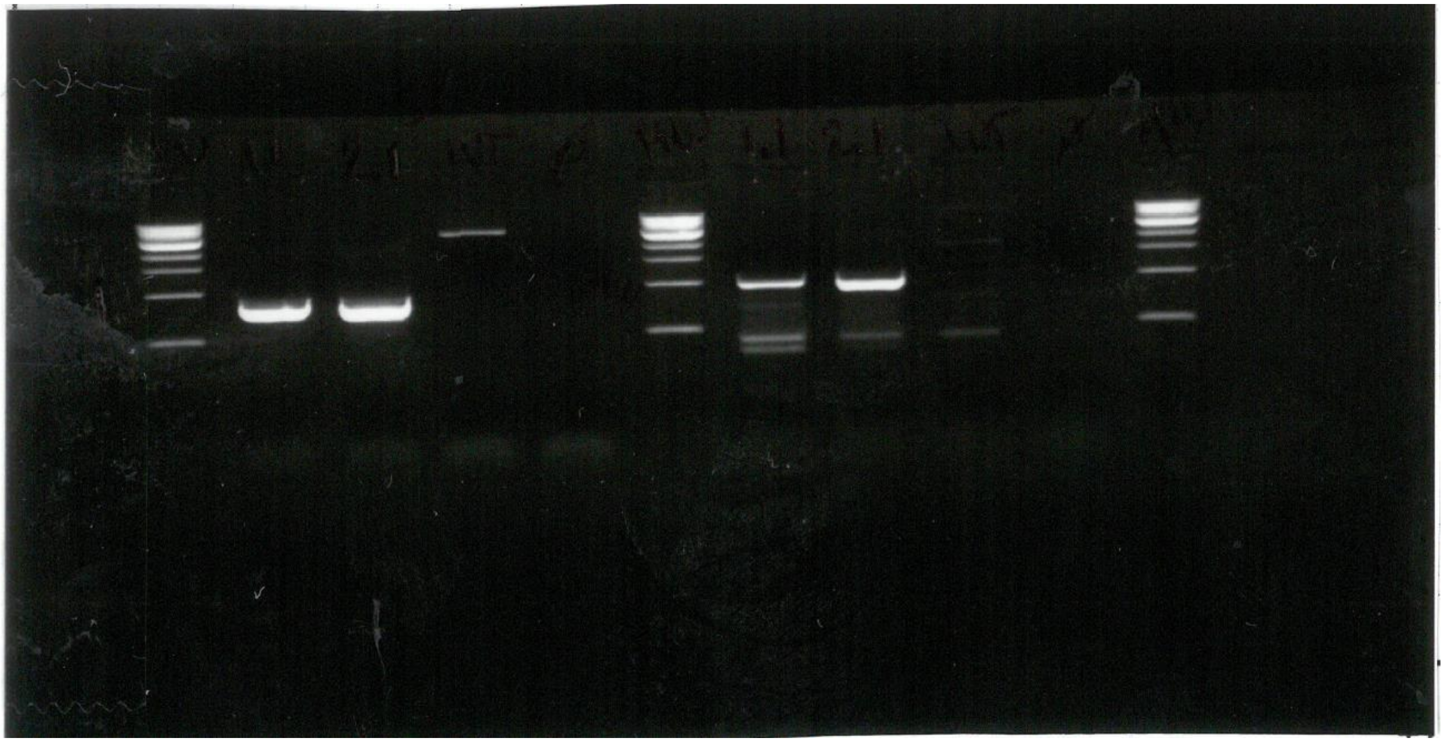

A

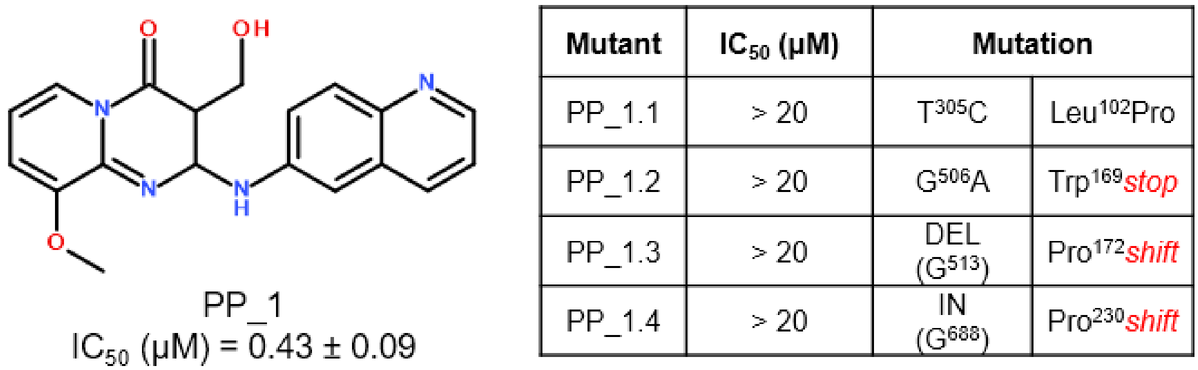

PP_1
IC_{50} (μM) = 0.43 ± 0.09

Mutant	IC_{50} (μM)	Mutation	
PP_1.1	> 20	$T^{305}C$	$Leu^{102}Pro$
PP_1.2	> 20	$G^{506}A$	Trp^{169}*stop*
PP_1.3	> 20	DEL (G^{513})	Pro^{172}*shift*
PP_1.4	> 20	IN (G^{688})	Pro^{230}*shift*

B

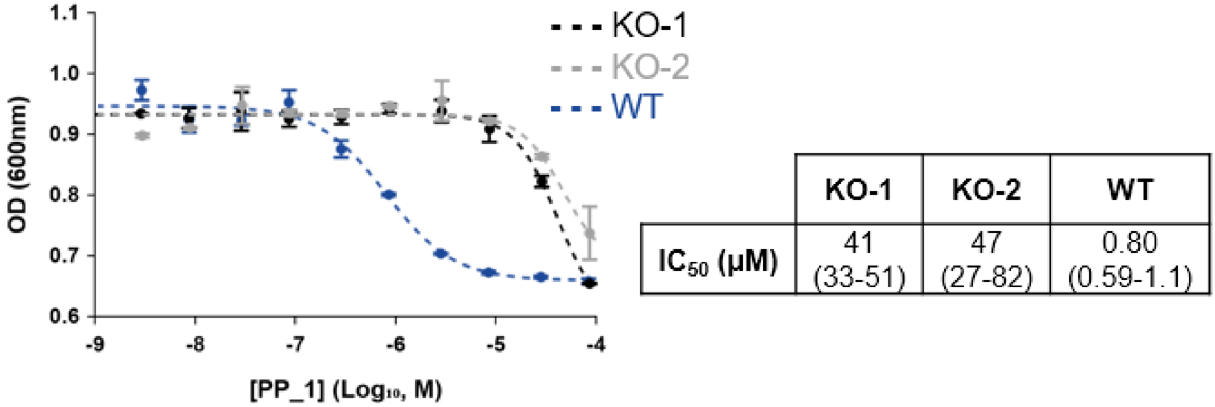

	KO-1	KO-2	WT
IC_{50} (μM)	41 (33-51)	47 (27-82)	0.80 (0.59-1.1)

C

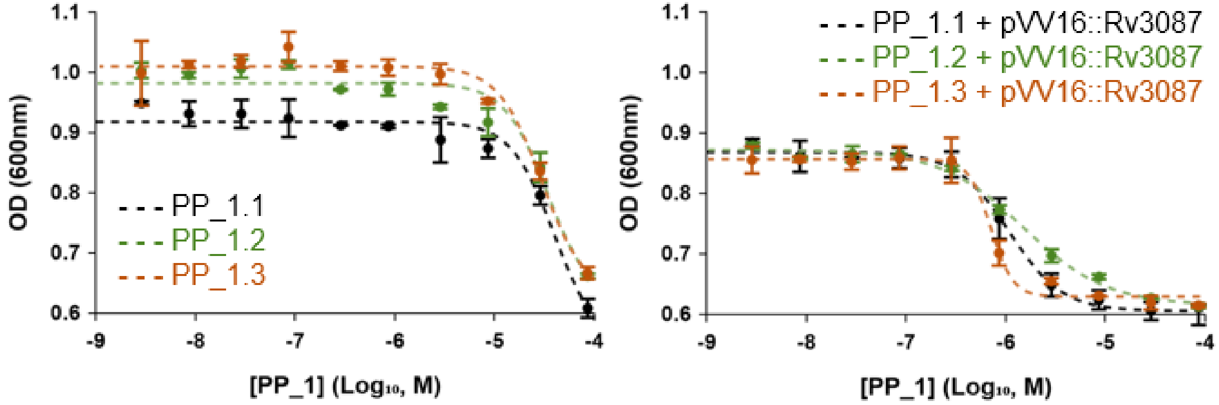

	PP_1.1	PP_1.2	PP_1.3	PP_1.1 + pVV16::Rv3087	PP_1.2 + pVV16::Rv3087	PP_1.3 + pVV16::Rv3087
IC_{50} (μM)	40 (29-55)	34 (24-48)	30 (23-39)	1.1 (0.84-1.4)	1.5 (1.2-1.9)	0.71 (0.56-0.91)

Figure S12. **Association of Rv3087 with resistance to PP_1. A**. *Left*, structure of the PP_1 derivative used for the generation of spontaneous resistant mutants. Activity value is average +/- SD for three biological replicates using H37Rv-GFP strain (each replicate was performed with a duplicated dose-response). *Right*, summary of the mutations identified by whole genome sequencing and confirmed by Sanger sequencing. DEL, deletion; IN, insertion; SNP, single nucleotide polymorphism. Activity values were determined using a resazurin reduction assay. **B**. Dose-response curves showing the activity of PP_1 on two confirmed Rv3087 knock-out (KO) mutants (KO-1, KO-2). **C**. Dose-response curves showing the activity of PP_1 on three resistant mutants, before (left) and after (right) transformation with pVV16::Rv3087 plasmid to constitutively overexpress Rv3087. For panels **B** and **C**, bacterial growth was monitored by optical density (OD) at 600 nm. Data are average and SD for a representative experiment performed in duplicates. IC_{50} values corresponding to the best fit are indicated, together with the 95% CI.

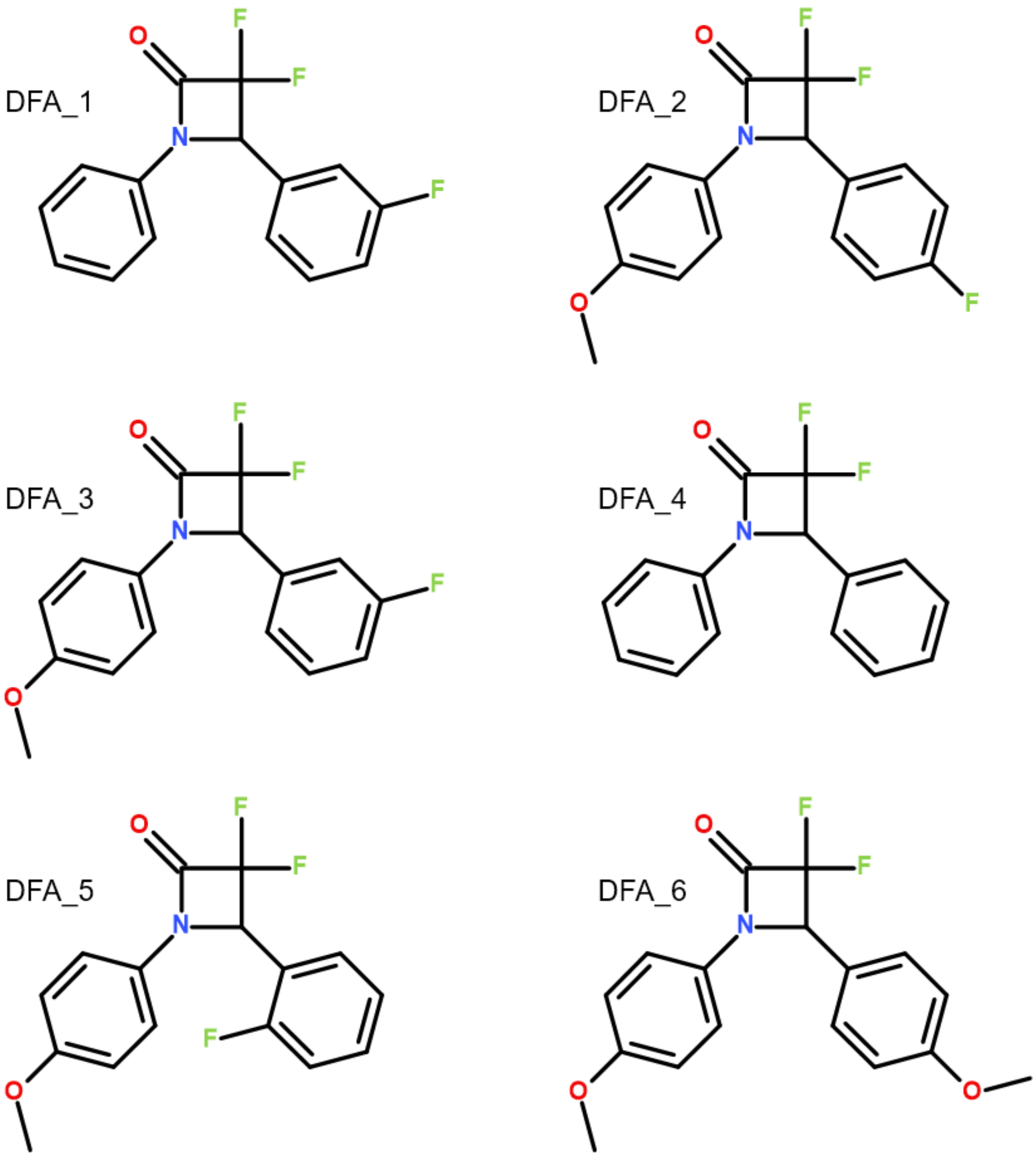

Figure S13. Structure of the 3,3-difluoro-2-azetidinone (DFA) derivatives identified as hits in the screening.

PL_1

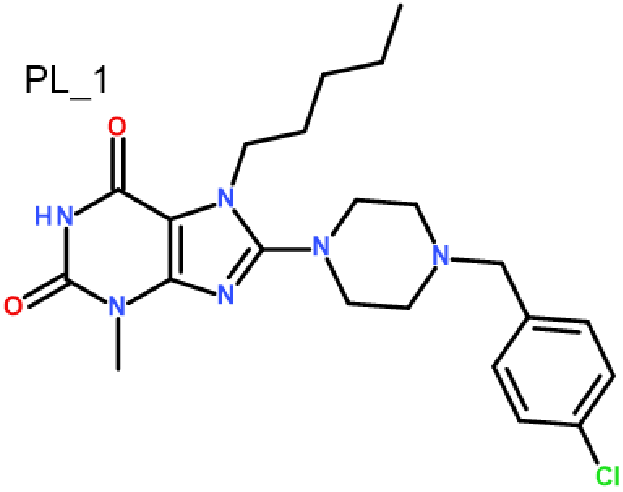

PL_2

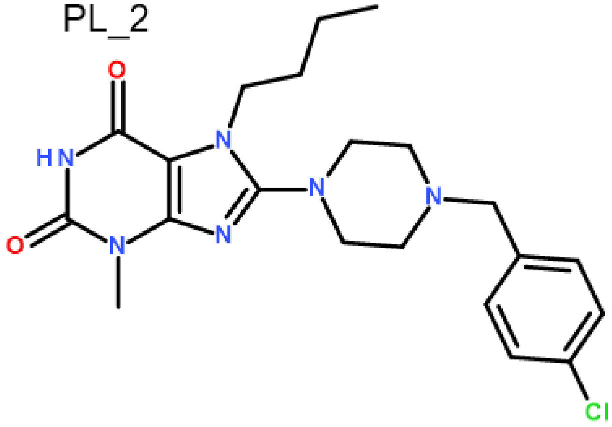

PL_3

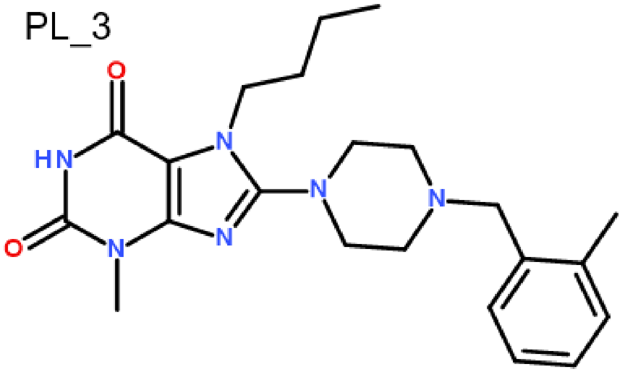

PL_4

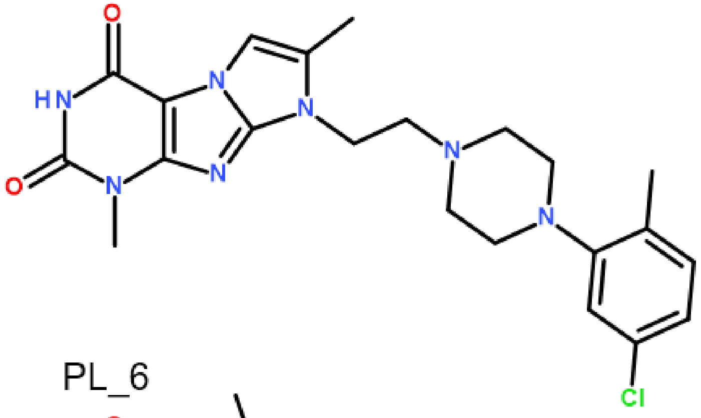

PL_5

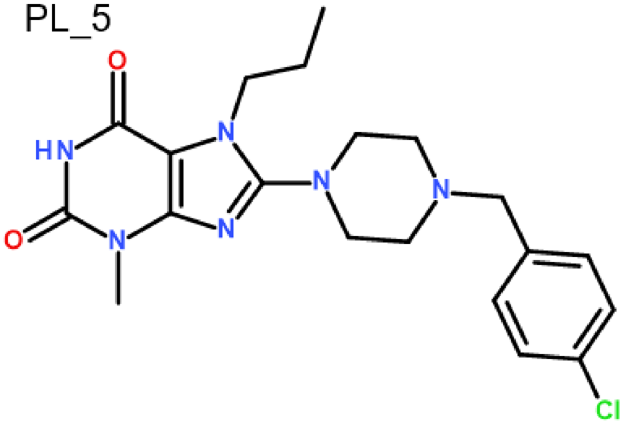

PL_6

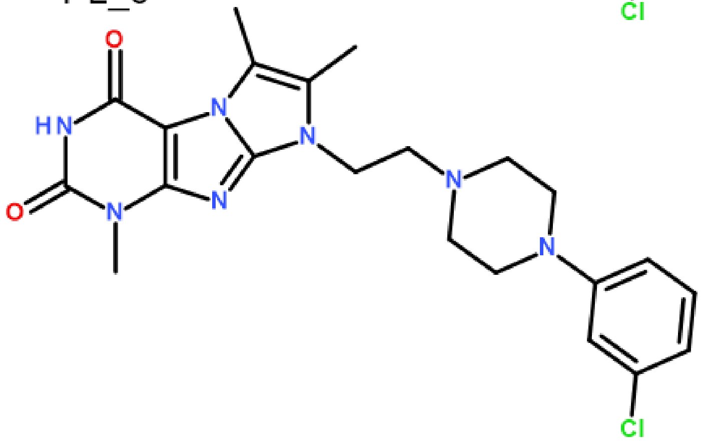

Figure S14. Structure of the 6 purine-like (PL) derivatives identified as hits in the screening.

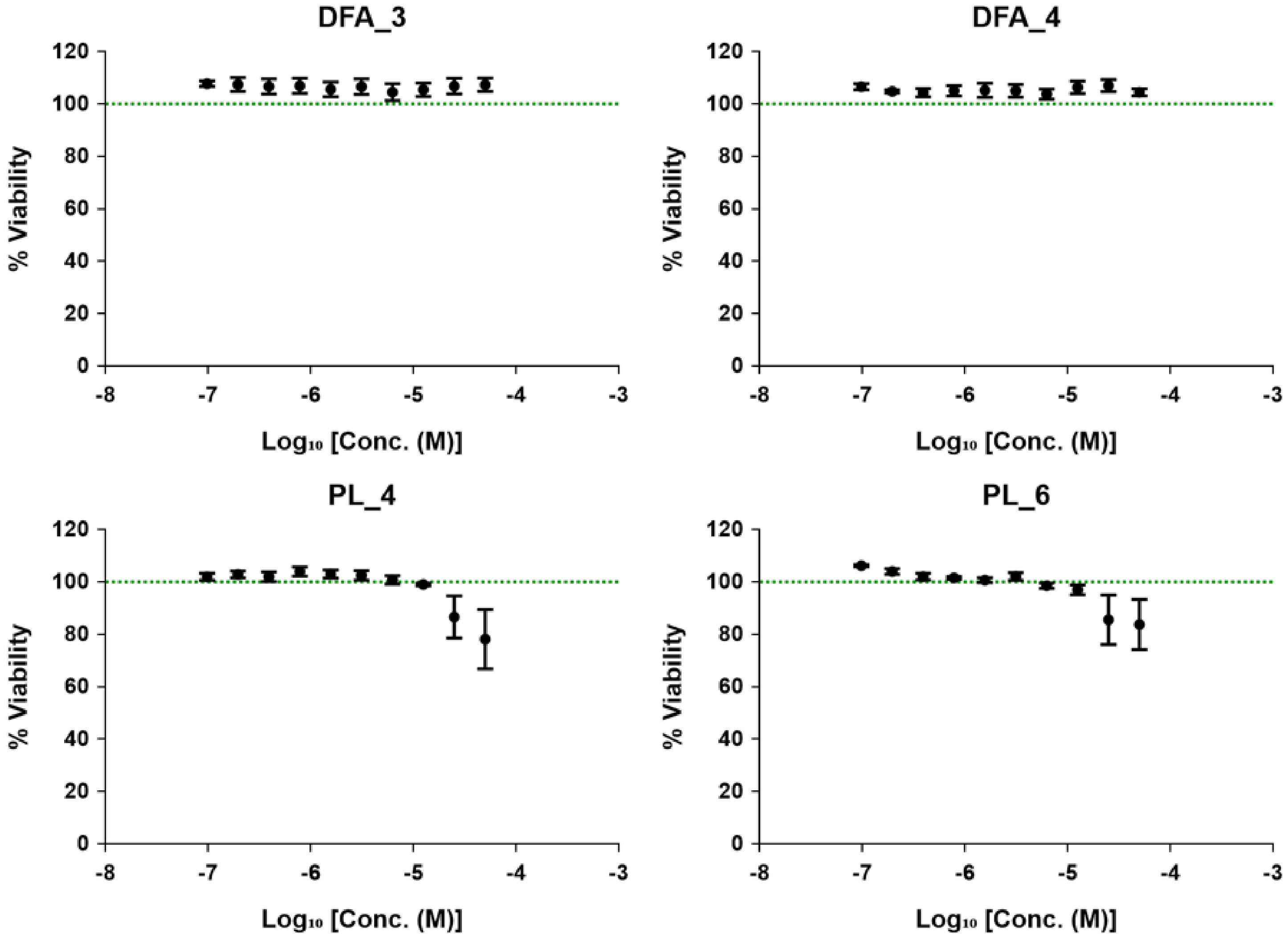

Figure S15. **Cytotoxicity profile of selected hit compounds**. Cytotoxicity was evaluated in 96-well plates, after 48h incubation of Raw264.7 macrophages (2×10^5 cells/mL) with the compounds, using the Alamar Blue viability assay as a read-out. Briefly, 50 µL of resazurin 0.01% in PBS was added in each well and the plate incubated for 4h at 37°C, 5% CO_2. Reading of the resorufin fluorescence was done at Ex. 535 nm and Em. 590 nm using a plate reader (Victor3, Perkin Elmer). The percentage of viability was calculated based on the average obtained for the DMSO control (n = 10, green line). Results are average +/- SD for a triplicated dose-response.

A

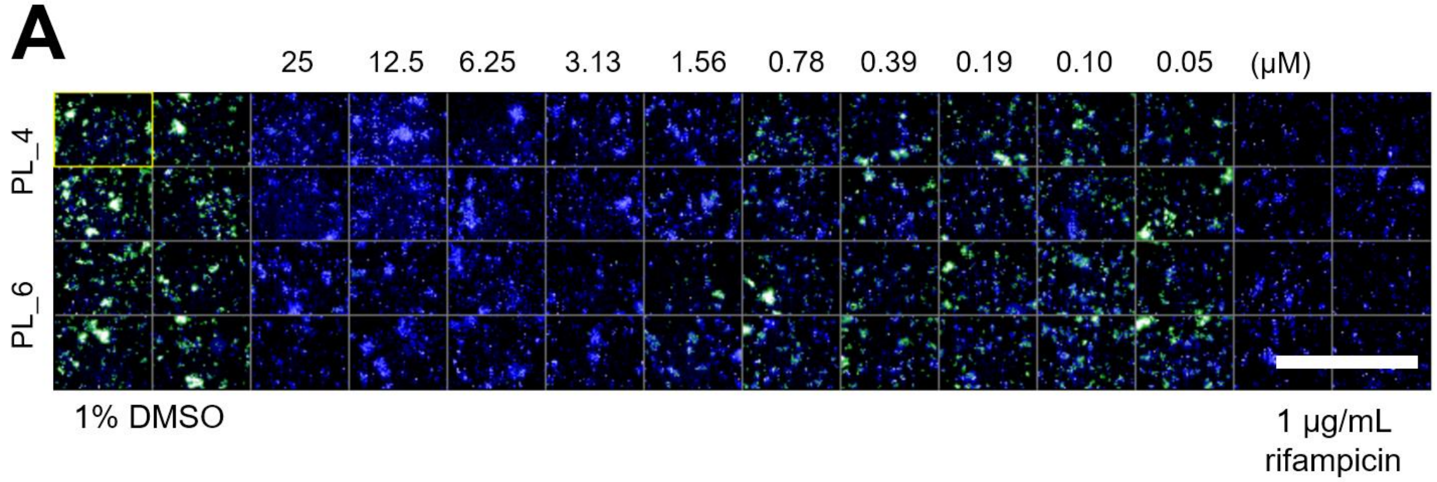

B

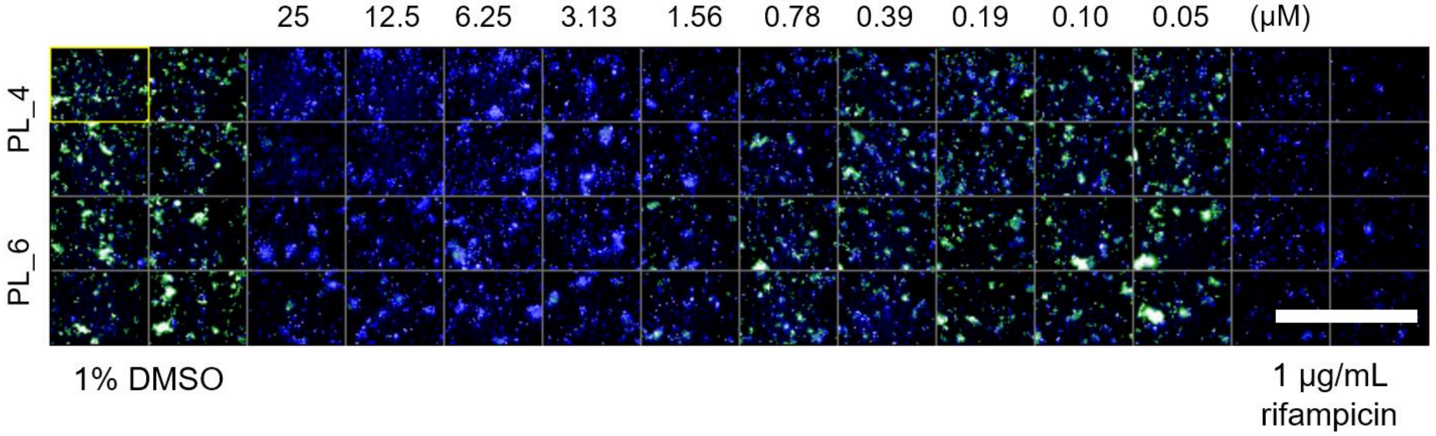

Figure S16. **Activity of PL_4 and PL_6 in the infected macrophage assay**. Fluorescence microscopy pictures of the dose-response curves showing the ability of PL_4 and PL_6 to restrict H37Rv-GFP growth in Raw264.7 macrophages, **A**. in absence or **B**. in presence of PAS (100 nM). GFP bacteria are visualized in green, macrophages were stained with the nuclear dye Hoechst 33342, shown in blue. Pictures for the negative (DMSO) and positive control (rifampicin) were also added (8 pictures each). Images shown are for a single field of view per well, for a representative experiment with a duplicated dose-response. Scale bar: 1 mm.

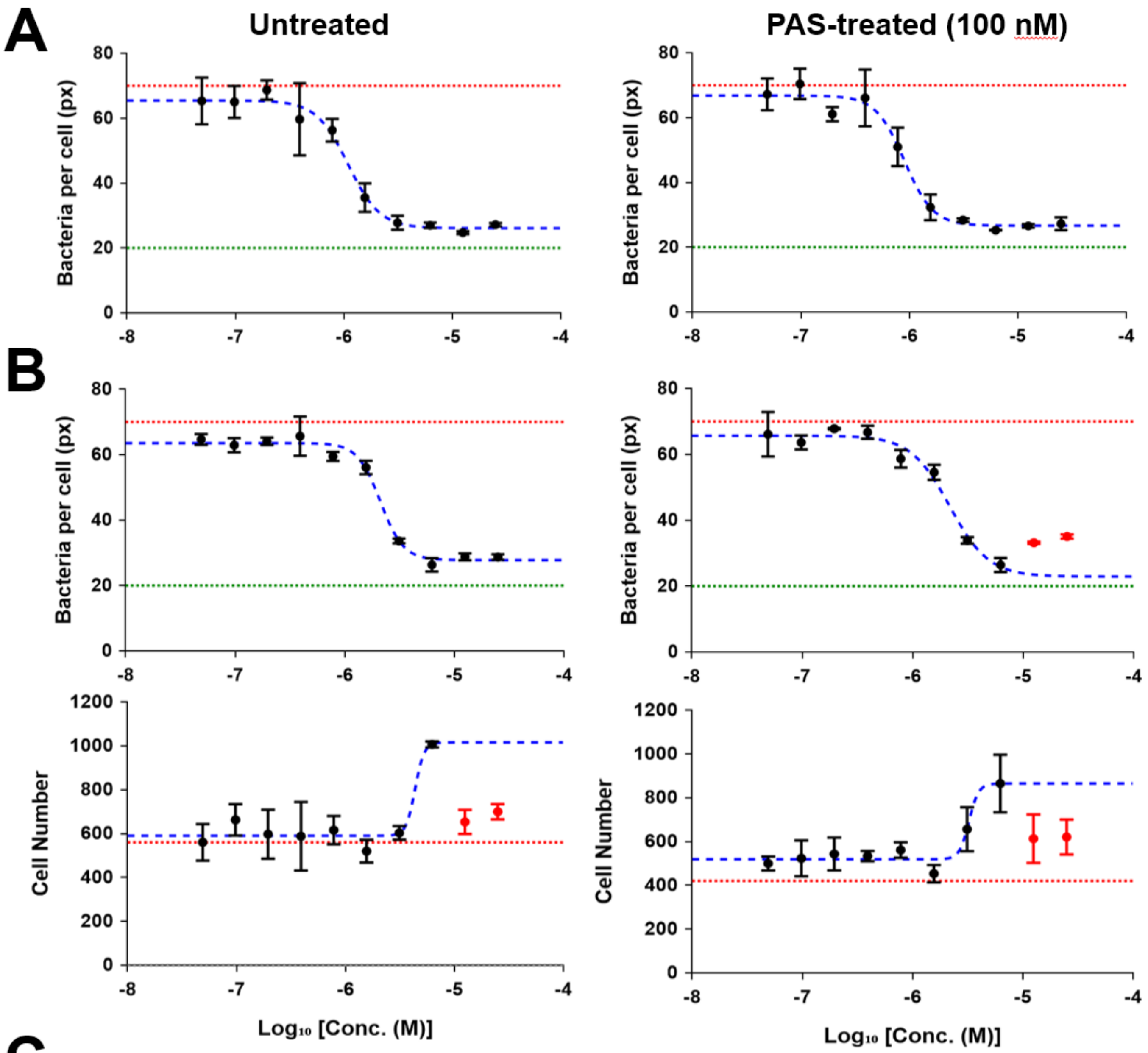

C

Compound	IC$_{50}$ (μM)	
	Untreated	*PAS-treated*
**PL_4**	1.2 ± 0.57	1.4 ± 0.92
**PL_6**	2.2 ± 0.96	1.9 ± 0.66

Figure S17. **Ability of PL_4 and PL_6 to restrict H37Rv-GFP growth in Raw264.7 macrophages**. Dose-response curves showing the reduction in intracellular bacteria with increasing concentration of **A**. PL_4 and **B**. PL_6. The red line indicates the average value obtained for the negative control (1% DMSO). The green line indicates the average value obtained for the positive control (1 μg/mL rifampicin). Points in red correspond to concentrations of PL_6 inducing a reduced viability in macrophages, as indicated in the bottom graph (*Cell Number* parameter), and were excluded from the fitting. Data shown are average and SD for a duplicated dose-response from a single, representative experiment. **C**. Summary of the IC$_{50}$ values found for PL_4 and PL_6 using this intracellular assay. Values are average +/- SD for three biological replicates (each replicate was performed with a duplicated dose-response).

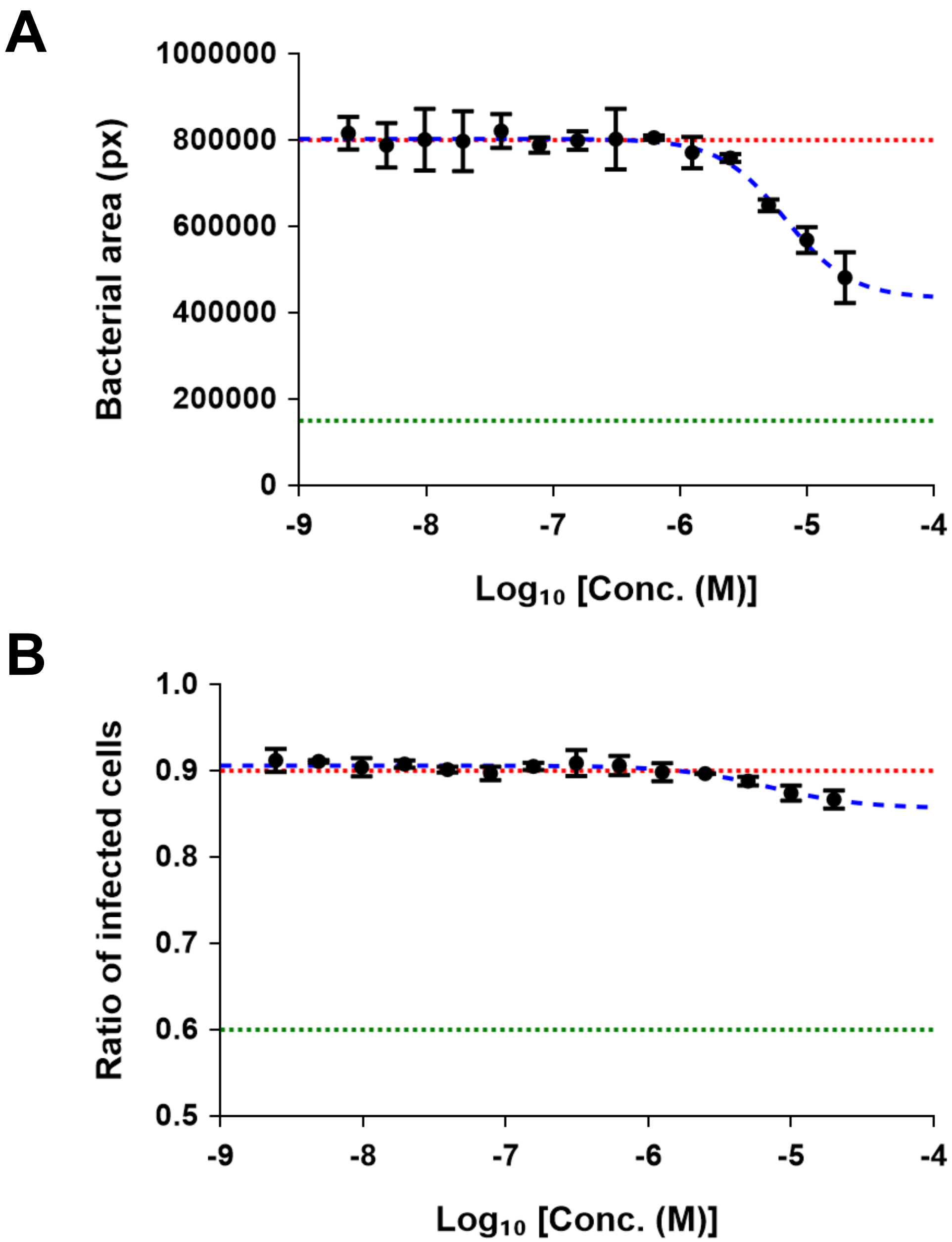

Figure S18. **PAS is unable to clear intracellular mycobacteria**. Dose-response curves showing **A**. a partial reduction in total bacteria area with increasing PAS concentration and **B**. an absence of effect for the ratio of infected cells, indicating inability of PAS to eliminate intracellular bacteria, while effectively preventing the growth of extracellular organisms freed after cell lysis. The red line indicates the average value obtained for the negative control (1% DMSO). The green line indicates the average value obtained for the positive control (1 µg/mL rifampicin). Data shown are average and SD for a duplicated dose-response from a single, representative experiment.

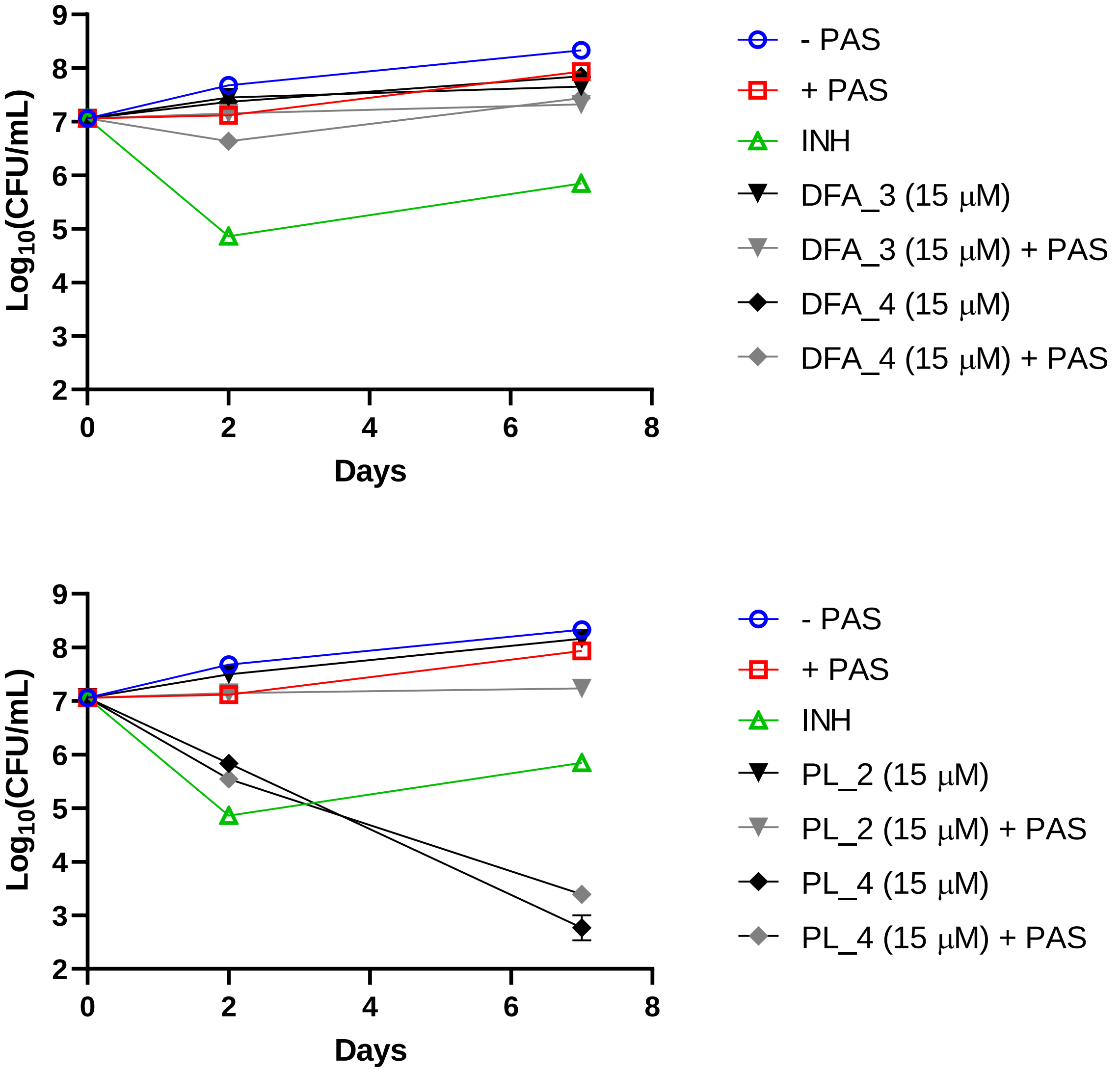

Figure S19. **Time-kill kinetics for DFA and PL compounds**. Bacteria were exposed to the compounds with or without PAS (100 nM) and the CFU enumerated after 2 and 7 days incubation. Untreated controls in absence (blue) or presence (red) of PAS were included to evaluate whether the different combinations were bacteriostatic or bactericidal. Isoniazid (INH, 10 μM) was included as a positive control for cidality. All counts were done in duplicates and the average +/- SD is shown.

	INH (IC_{50}, µM)	RIF (IC_{50}, nM)
**-**	0.20	1.27
**DFA_3 (10 µM)**	0.15	0.52
**DFA_4 (10 µM)**	0.15	0.70

Figure S20. **Benefits of DFA combination with other antibiotics**. H37Rv-GFP bacteria were grown as described in the main manuscript (secondary screening) and exposed to dose-responses of isoniazid (INH) or rifampicin (RIF) in presence or absence of DFA_3 or DFA_4 (10 µM). The RFU were measured after 5 days of incubation and IC_{50} values calculated by least-square regression. Values are from a single, triplicated dose-response.
